# *In Silico* Exploration of Potential Natural Inhibitors against SARS-Cov-2 nsp10

**DOI:** 10.3390/molecules26206151

**Published:** 2021-10-12

**Authors:** Ibrahim H. Eissa, Mohamed M. Khalifa, Eslam B. Elkaeed, Elsayed E. Hafez, Aisha A. Alsfouk, Ahmed M. Metwaly

**Affiliations:** 1 Pharmaceutical Medicinal Chemistry & Drug Design Department, Faculty of Pharmacy (Boys), Al-Azhar University, Cairo 11884, Egypt; mohamedKhalifa2321.el@azhar.edu.eg; 2 Department of Pharmaceutical Sciences, College of Pharmacy, Almaarefa University, Riyadh 13713, Saudi Arabia; ikaeed@mcst.edu.sa; 3 Department of Plant Protection and Biomolecular Diagnosis, ALCRI, City of Scientific Research and Technological Applications, New Borg El-Arab City 21934, Egypt; elsayed_hafez@yahoo.com; 4 Department of Pharmaceutical Sciences, College of Pharmacy, Princess Nourah bint Abdulrahman University, Riyadh 11564, Saudi Arabia; aaalsfouk@pnu.edu.sa; 5 Pharmacognosy and Medicinal Plants Department, Faculty of Pharmacy (Boys), Al-Azhar University, Cairo 11884, Egypt

**Keywords:** COVID-19, natural products, SARS-Cov-2 nsp10, structural similarity, fingerprint, molecular docking, ADMET, toxicity, DFT

## Abstract

In continuation of our previous effort, different *in silico* selection methods were applied to 310 naturally isolated metabolites that exhibited antiviral potentialities before. The applied selection methods aimed to pick the most relevant inhibitor of SARS-CoV-2 nsp10. At first, a structural similarity study against the co-crystallized ligand, S-Adenosyl Methionine (**SAM**), of SARS-CoV-2 nonstructural protein (nsp10) (PDB ID: 6W4H) was carried out. The similarity analysis culled 30 candidates. Secondly, a fingerprint study against **SAM** preferred compounds **44**, **48**, **85**, **102**, **105**, **182**, **220**, **221**, **282**, **284**, **285**, **301**, and **302**. The docking studies picked **48**, **182**, **220**, **221**, and **284**. While the ADMET analysis expected the likeness of the five candidates to be drugs, the toxicity study preferred compounds **48** and **182**. Finally, a density-functional theory (DFT) study suggested vidarabine (**182**) to be the most relevant SARS-Cov-2 nsp10 inhibitor.

## 1. Introduction

More than 217 million humans around the world were confirmed to be infected with COVID-19 and another 4.5 million families lost one of their beloveds as stated by the WHO on 2 September 2021 [1]. In response, all scientists in the field of drug discovery should work unceasingly to discover a cure against the notorious virus.

Computer-assisted (based or aided) drug design is a well-established branch of drug design that covers various *in silico* computational and theoretical approaches. These approaches are essential contributors to the development of new bioactive agents [2,3,4,5,6,7,8]. Computer-assisted drug design has been applied in drug discovery [9,10,11], computational chemistry [12,13], toxicity prediction [14,15,16], ADMET assessment [17,18,19], molecular modeling [20], molecular design [21,22], and rational drug design [23,24,25,26,27]. All these techniques have great popularity and have been used in both academic fields in addition to the pharmaceutical industries [28]. This approach has been introduced successfully and recurrently as a powerful weapon in the global fight against COVID-19 [29,30,31,32].

The relationship between humans and nature dates back to the prehistoric ages. The latter supplied the former with food, tools of beauty, and treatment [33,34]. Plants [35,36] and lately microorganisms [37,38] have been extensively screened to explore their healing power. Scientists isolated the secondary metabolites produced by these natural sources and labeled them as the key element in bioactivity. These candidates belonged to various classes as isochromenes [39], α-pyrones [40], diterpenes [41,42], sesquiterpenes [43,44], steroids [45], flavonoids [46,47], alkaloids [48], and saponins [49,50].

SARS-CoV-2 is an enveloped positive-sensed RNA virus. The replication of SARS-CoV-2 depends on a group of 16 non-structural proteins. These proteins have the codes of nsp1–nsp16. Between them, the two proteins nsp10 and nsp16 make an essential protein complex [51]. That complex is responsible for the vital methylation reaction at the ribose 2′-O position of the penultimate nucleotide of the viral RNA cap [52]. Accordingly, if a molecule could bind with that enzyme and inhibit this essential step, the replication process will be stopped.

The targeting of SARS-CoV-2 nsp-16 with a library of 10 [53] and 265 [54] FDA-approved compounds was studied before. Likely, a group set of 22 natural compounds from some Indian plants was computationally screened against six non-structural-proteins of SARS-CoV-2 [55].

**Figure 1:**
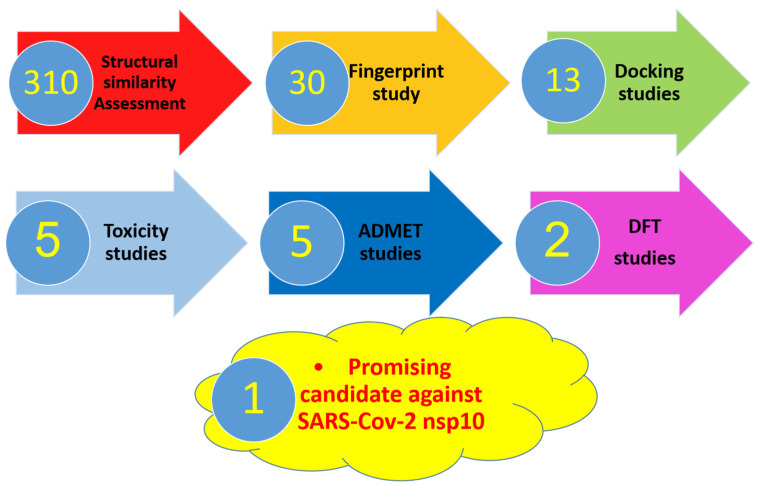
The applied *in silico* selection protocols.

In this study, different computational (*in silico*) selection methods were applied to 310 candidates. The examined candidates were chosen through a deep database search according to three parameters. The first parameter was to be naturally isolated. The second was having exhibited antiviral potentiality before. Lastly, we considered that the culled compounds belong to different chemical classes and accordingly have various chemical structures. The applied computational techniques were a structural similarity study against **SAM** followed by a fingerprint study against the same target. The selected candidates were docked against nsp10 (PDB ID: 6W4H) to prefer **44**, **48**, **85**, **102**, **105**, **182**, **220**, **221**, **282**, **284**, **285**, **301**, and **302**. Then ADMET and toxicity studies further picked two candidates. Finally, a DFT study suggested the most relevant inhibitor of SARS-Cov-2 nsp10 (Figure 1).

## 2. Results and Discussion

### 2.1. Molecular Similarity against ***SAM***

The basic principle of 2D Molecular similarity is that molecules with similar chemical structures are expected to have similar biological activities [56].

To measure the similarity of two objects, their general features have to be compared. On a molecular level, the molecular features or descriptors of any compound start from the general physicochemical properties and extend to more specific structural features such as partition coefficient (ALog p) [57], molecular weight (M. Wt) [58], hydrogen bond donors (HBA) [59], hydrogen bond acceptors (HBD) [60], number of rotatable bonds [61], number of rings, and also aromatic rings [62], in addition to molecular fractional polar surface area (MFPSA) [63].

**Figure 2:**
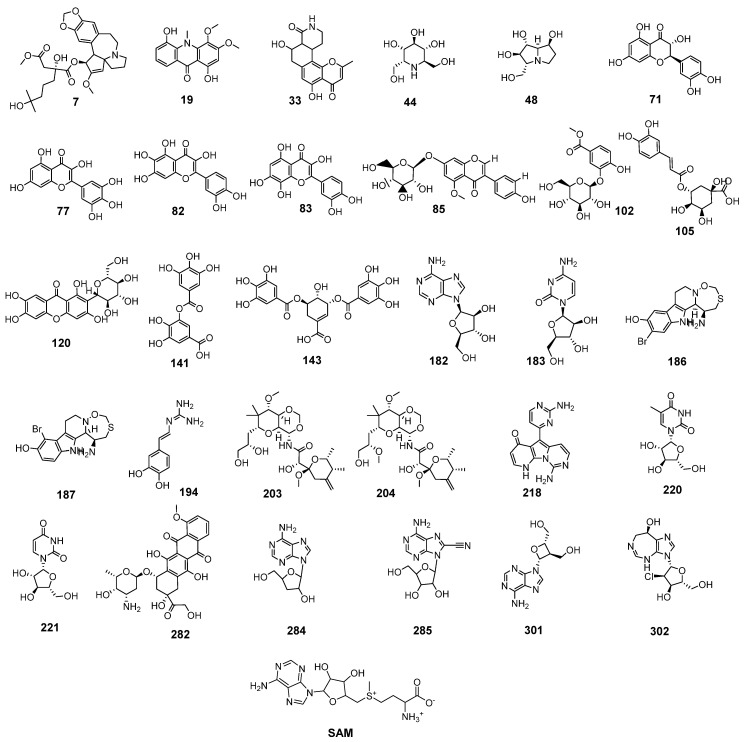
The most similar candidates with (**SAM**).

All mentioned molecular properties were used in the applied similarity study between the natural candidate’s set (Figure S1, Supplementary Materials) and the co-crystallized ligand (**SAM**) of SARS-CoV-2 nonstructural protein (nsp10) (PDB ID: 6W4H) using Discovery studio software. Thirty candidates (Figure 2) were chosen to be the most similar to **SAM**. 

As shown in Figure 2, the similar candidates showed a high degree of structural similarity with **SAM**. In detail, most candidates have a sugar-like moiety as that of **SAM** as candidates **85**, **102**, **105**, **120**, **182**, **183**, **203**, **204**, **220**, **221**, **282**, **284**, **285**, **301**, and **302**. These moieties may serve as a good center for hydrogen bonding interaction with the target receptor. Furthermore, most candidates have hetero bicyclic structures as present in **SAM**. Besides, xanthine-like structures were defined in many similar candidates such as **182**, **284**, **285**, and **301**.

**Figure 3:**
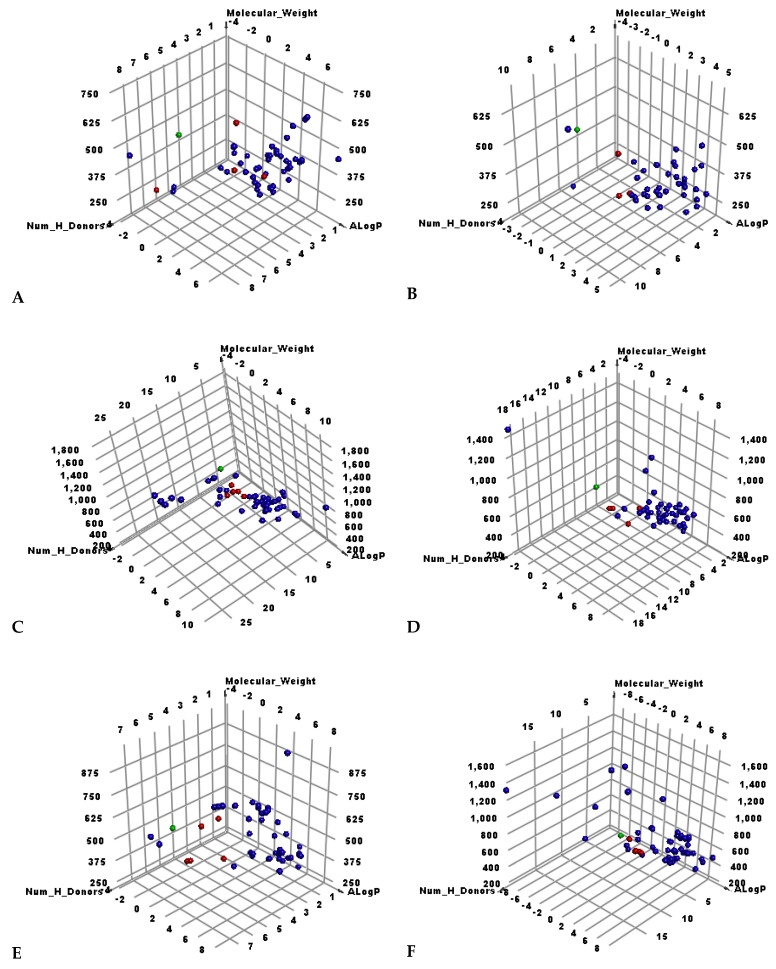
The results of similarity analysis of the test sets and **SAM**. Green = **SAM**, red = similar candidate, blue = not similar candidate. (**A**) Candidates 1–50, (**B**) 51–100, (**C**) 101–150, (**D**) 151–200, (**E**) 201–250, (**F**) 251–310.

As shown in Figure 3, the candidate’s set was divided into six smaller sets. From the first set to the fifth comprised 50 candidates while the sixth set was 60.

Table 1 demonstrates the molecular properties of the similar candidates as well as **SAM**.

### 2.2. Filter Using Fingerprints

The fingerprint is another similarity technique that depends on the 2D molecular structures of two different ligands in a binary format. This technique computes the presence and/or absence of several sub-structural fragments to calculate the degree of inter-molecular structural similarity. This technique is utilized as a tool to detect the degree of similarity between a hit candidate and a lead one [64] The fingerprint approach examines the following parameters: charges [65], hybridization [66], H-bond acceptors, and donors [67], positive and negative ionizable moieties [68], halogens, and aromatic rings beside the ALogP category of candidates. The experiment was carried out using Discovery Studio.

The fingerprint’s output depends on Tanimoto coefficient (SA/(SA + SB + SC)). SA is a symbol that represents the number of bits present in the reference molecule (**SAM**) and the examined candidate. On the other hand, SB and SC represent the number of bits in the examined candidate but not **SAM** and the number of bits in **SAM** but not the examined candidate, respectively. The Tanimoto coefficient gives values with a range of zero (no shared bits) to one (all bits the same).

The results revealed the significant fingerprint similarity of **44**, **48**, **85**, **102**, **105**, **182**, **220**, **221**, **282**, **284**, **285**, **301**, and **302** with **SAM** (Table 2).

The reported antiviral potentialities of the preferred metabolites were summarized in the Supplementary Materials.

### 2.3. Docking Studies

Molecular docking studies were achieved to study the binding modes, orientations, and affinities of the candidates **44**, **48**, **85**, **102**, **105**, **182**, **220**, **221**, **282**, **284**, **285**, **301**, and **302** inside the SARS-CoV-2 nonstructural protein (nsp10) (PDB ID: 6W4H, resolution: 1.80 Å) active site using MOE 14.0 software.

**Figure 4:**
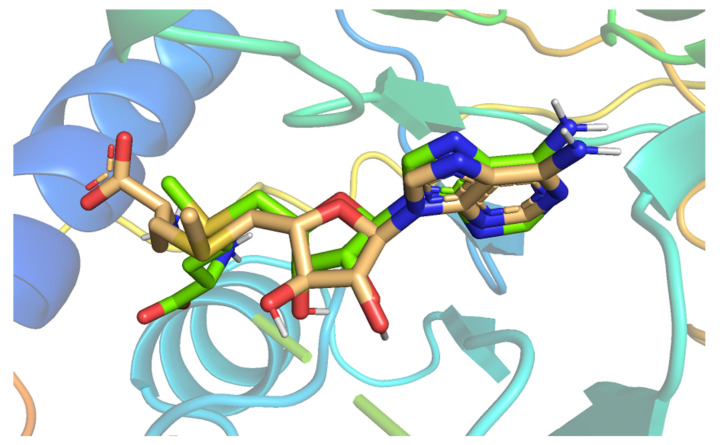
Superimposition of the co-crystallized ligand pose (**green**) and the docking pose (**wheat**).

The docking process was validated through a redocking step of **SAM** against active pockets of SARS-CoV-2 nonstructural protein (nsp10). The suitability of the performed protocol was demonstrated by the small RMSD (0.60 Å) that was found between the docked pose and **SAM** (Figure 4).

**Figure 5:**
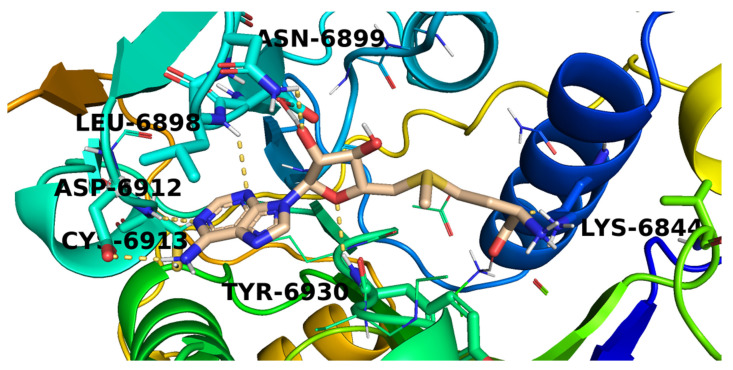
The proposed binding pattern of **SAM**.

The mode of binding of **SAM** inside COVID-19 nsp10 was illustrated in Figure 5. It was noticed that **SAM** interacted with the active site via the formation of six hydrogen bonds with Lys6844, Leu6898, Asn6899, Asp6912, Cys6913, and Tyr6930.

Among all studied metabolites, members **220**, **48**, **182**, **221**, and **284** exhibited the greatest binding free energies of docking (Table 3).

**Figure 6:**
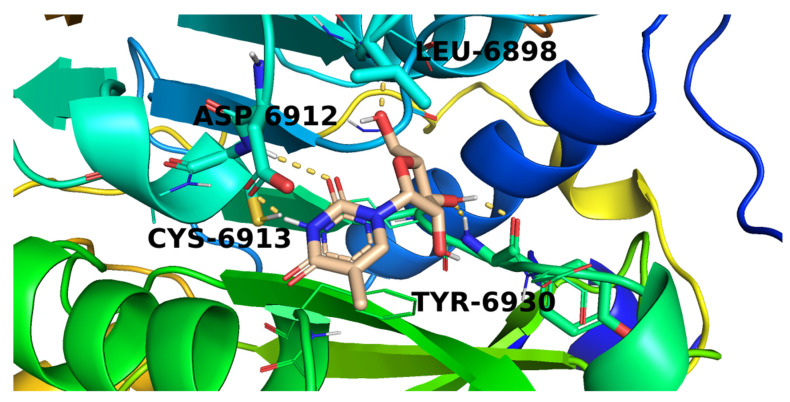
The proposed binding pattern of candidate **220**.

The methylpyrimidine-2,4-dione derivative (**220**) possessed a good potential affinity of −21.17 into the COVID-19 nsp10 active site. This high affinity is attributed to the formation of five hydrogen bond interactions. The pyrimidine moiety of candidate **220** was involved in two hydrogen-bonding interactions with Asp6912 and Cys6913. While the furan part interacted with the active site by three hydrogen bonds with Leu6898 and Tyr6930 (Figure 6).

**Figure 7:**
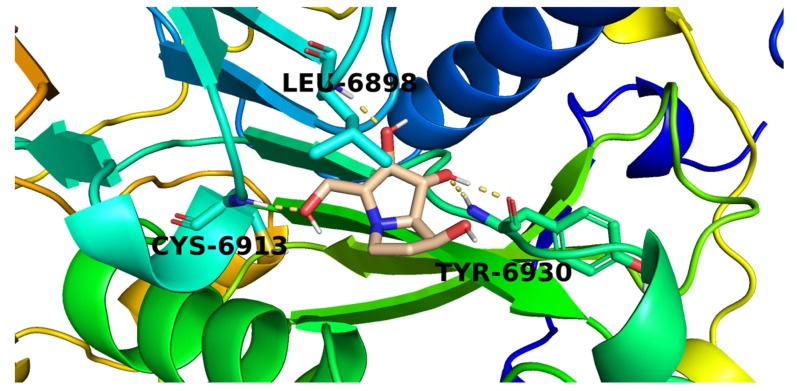
The proposed binding pattern of candidate **48**.

Candidate (**48**) exhibited a binding mode like that of **SAM** with the formation of four hydrogen bonds with Cys6913, Tyr6930, and Leu6898 (Figure 7).

**Figure 8:**
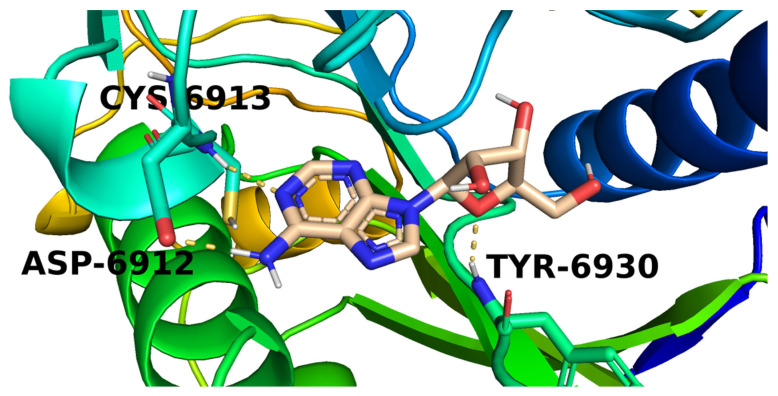
The proposed binding pattern of candidate **182**.

Investigation of the top docking poses of the 6-aminopurine member (**182**) showed that it interacted with the COVID-19 nsp10 active site through the formation of three hydrogen bond interactions. Its amino group was involved in a hydrogen bond with Asp6912 while one purine nitrogen atom formed a hydrogen bond with Cys6913. In addition, the furan oxygen interacted by a hydrogen bond with Tyr6930 (Figure 8).

**Figure 9:**
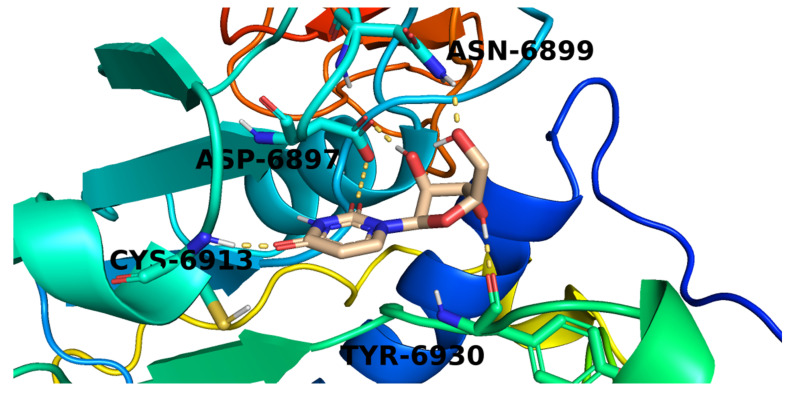
The proposed binding pattern of candidate **221**.

The proposed binding pattern of the pyrimidinedione derivative (**221**) was illustrated in Figure 9. It interacted with the active site via the formation of five hydrogen bonds with Asn6899, Asp6897, Cys6913, and Tyr6930.

**Figure 10:**
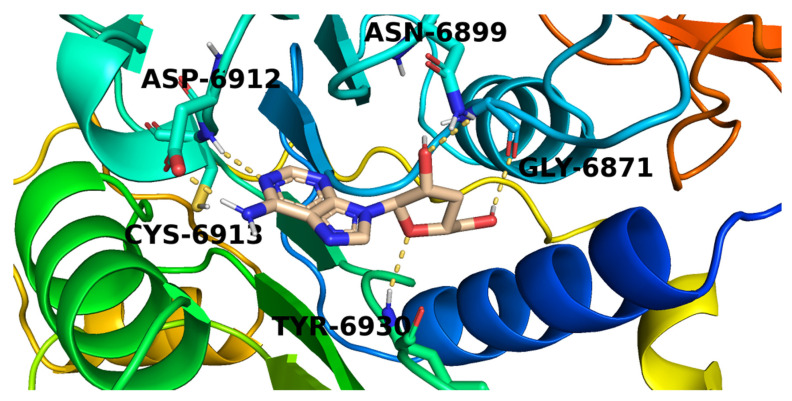
The proposed binding pattern of candidate **284**.

Figure 10 The proposed binding mode of candidate **284**. The purine moiety of **284** formed a hydrogen bond with Asp6912 while the attached amino group interacted with another hydrogen bond with Cys6913. The tetrahydrofuran-3-ol part formed two hydrogen bonds with Tyr6930 and Asn6899. Furthermore, the hydroxymethyl side chain was involved by a hydrogen bond with Gly6871.

### 2.4. In Silico ADMET Analysis

**Figure 11:**
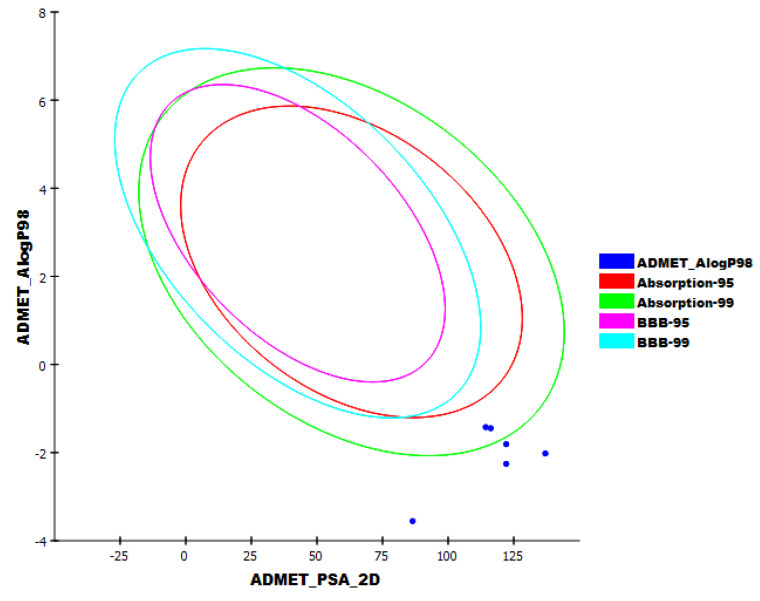
The expected ADMET study.

Five parameters were examined for candidates **48**, **182**, **220**, **221**, and **284** using Discovery studio software. Acyclovir, the potent anti-viral drug, was used as a reference candidate. The results are illustrated in Figure 11.

All the tested candidates have a very low chance to penetrate BBB. This indicates the high safety margin of such derivatives against the CNS. Additionally, all candidates exhibited an aqueous solubility character. For intestinal absorption, candidates **48**, **182**, **220**, and **221** were predicted to have poor to very poor levels, while candidate **284** was expected to have a moderate level. Furthermore, all candidates were predicted to be CYP2D6 non-inhibitors and can bind plasma protein by less than 90%. These results indicated that all the tested candidates have good pharmacokinetic properties and can be utilized for further investigations.

### 2.5. In Silico Toxicity Studies

Candidates **48**, **182**, **220**, **221**, and **284** were tested *in silico* for their proposed toxicity using Discovery studio software. In this test, seven toxicity models were utilized using ribavirin as a reference. The results are summarized in Table 4.

FDA rodent carcinogenicity in female mice indicated that candidates **48** and **182** were non-carcinogenic, while candidates **220**, **221**, and **284** had some sort of carcinogenicity. Besides, candidates **48**, **182**, and **284** showed TD_50_ values of 9.295, 4.245, and 6.402 mg/kg body weight/day, respectively. Candidates **220** and **221** showed high carcinogenic potency TD_50_ values of 67.851 and 55.437 mg/kg body weight/day, respectively. Furthermore, candidates **48** and **182** showed high rat maximum tolerated dose values of 0.191 and 0.175 g/kg body weight, respectively. On the other hand, candidates **220** and **221** showed low rate maximum tolerated dose values of 0.095 and 0.094 g/kg body weight, respectively. Candidate **284** showed a comparable rat maximum tolerated dose value (0.155 g/kg body weight) with ribavirin (0.154 g/kg body weight). The tested candidates showed rat oral LD_50_ values ranging from 0.778 to 6.173 g/kg body weight, which were higher than the reference drug LD_50_ = 0.750 g/kg body weight. For the rat chronic LOAEL model, candidates **48** and **182** showed high values of 0.018 and 0.010 g/kg body weight, while candidates **220**, **221**, and **284** showed low values of 0.009, 0.006, and 0.004 g/kg body weight, respectively. All candidates were predicted to have mild to moderate irritant effects against ocular irritancy and skin irritancy models. Accordingly, candidates **48** and **182** had low toxicity profiles and were preferred for further studies.

### 2.6. DFT Studies

**Figure 12:**
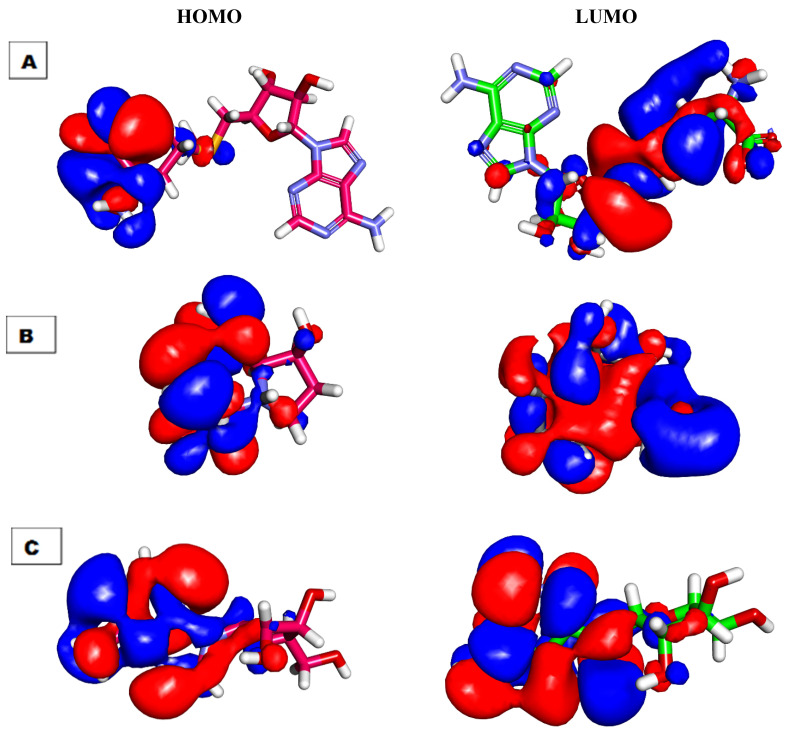
Spatial distribution of molecular orbitals for (**A**) **SAM**, (**B**) candidate **48**, and (**C**) candidate **182**.

**Figure 13:**
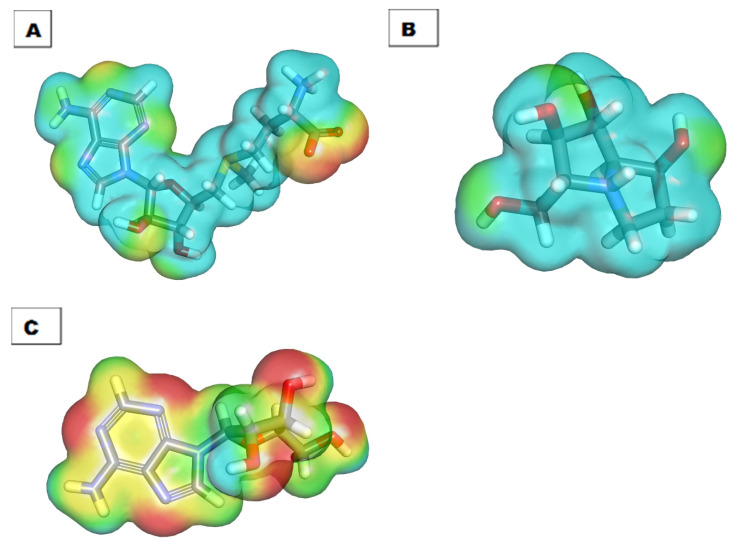
Molecular electrostatic potential map of (**A**) **SAM**, (**B**) candidate **48**, and (**C**) candidate **182**.

DFT parameters (Table 5) were studied for candidates 48 and 182 [69,70] against **SAM** as a reference using Discovery studio software (Table 5, Figure 12 and Figure 13).

#### 2.6.1. Molecular Orbital Analysis

Candidates **48**, **182**, and **SAM** exhibited total energy values of −664.379, −955.658, and −1675.931 kcal/mol, respectively. The higher total energy of candidate **182** indicates a higher reactivity against the biological target. The two tested candidates, **48** and **182**, showed almost equal dipole moment values of 1.391 and 1.396, respectively. The Molecular Orbital (MO) analysis of EHOMO represents the energy of the highest occupied molecular orbital. On the other side, ELUMO represents the lowest unoccupied molecular orbital energies. The MO analysis is one of the essential parameters that is linked to the chemical reactivity and stability of a molecule. The HOMO spatial distributions of **SAM** are mainly presented on the 2-aminobutanoic acid moiety (the electron transfer zones), while its LUMO spatial distributions are located on the tetrahydrofuran-3,4-diol moiety (the electron acceptor zones). For candidate **48**, the HOMO spatial distributions are mainly located on the (2*R*,3*R*,4*R*)-2-(hydroxymethyl)pyrrolidine-3,4-diol moiety, while its LUMO spatial distributions are found on the (*S*)-pyrrolidin-3-ol moiety. For candidate **182**, the HOMO spatial distributions are mainly presented on the 9*H*-purin-6-amine moiety, while its LUMO spatial distributions are located on the (2*R*,3*S*,4*R*)-2-(hydroxymethyl)tetrahydrofuran-3,4-diol moiety. Furthermore, the gap energy of candidate **182** (0.128 kcal/mol) was less than that of candidate **48** (0.210 kcal/mol), confirming the high reactivity of candidate **182**. Consequently, candidate **182** may serve as a promising candidate for further studies.

#### 2.6.2. Molecular Electrostatic Potential Maps (MEP)

MEP was used to specify the electrostatic potential of **48**, **182**, and **SAM** in a 3D form via the calculation of the partial charges, electronegativity, and chemical reactivity [71]. The electrostatic potential affects the binding of a drug with a specific protein and gives a deeper insight into drug–receptor interaction [72]. In MEP, the red color denotes the electronegative atoms, which can go through hydrogen bonding interactions as an acceptor. Additionally, the blue color denotes the electron-poor atoms that can form a donor in hydrogen bonding. The green to yellow color denotes the neutral atoms, which can form hydrophobic interactions [73].

The MEPs of **SAM**, **48**, and **182**, were illustrated in Figure 13A, B, and C, respectively. Investigating these figures indicated that **SAM** has eight red patches that are suitable for hydrogen bonding acceptors and are considered favorable sites for the electrophilic attack. Also, it comprises six blue patches that are suitable for hydrogen bond donors (the most favorable sites for the nucleophilic attack). Candidate **182** has six red patches and five blue patches. In addition, there is a yellow patch on the 9*H*-purine nucleus indicating a high possibility for hydrophobic interaction. These findings are highly like that of **SAM**. The MEP of candidate **48** is slightly different from **SAM**. In detail, it has four red patches and four blue patches. These results indicated that candidate **182** has a greater similarity with **SAM** than candidate **48**. Because of that, candidate **182** was singled out.

The antiviral activities of the preferred candidate, vidarabine (**182**), were reported against several viruses in different reports. It was active against herpes simplex encephalitis and neonatal herpes simplex infection [74,75], HBV [76], varicella-zoster virus [77], human polyomavirus [78], adenovirus [79], and Epstein–Barr virus infection [80].

## 3. Method

### 3.1. Molecular Similarity Detection

Achieved by Discovery studio software (see method part in Supplementary Materials).

### 3.2. Pharmacophoric Study

Achieved by Discovery studio software (see method part in Supplementary Materials).

### 3.3. Docking Studies

Docking studies were achieved by MOE.14 software (see method part in Supplementary Materials).

### 3.4. ADMET Analysis

Achieved by Discovery studio 4.0 (see method part in Supplementary Materials).

### 3.5. Toxicity Studies

Achieved by Discovery studio software [81,82,83] (see method part in Supplementary Materials).

### 3.6. DFT Studies

Achieved by Discovery studio software [84] (see method part in Supplementary Materials).

## 4. Conclusions

Vidarabine (**182**) was suggested to be the most relevant SARS-Cov-2 nsp10 inhibitor among 310 naturally isolated metabolites that exhibited antiviral potentialities before. This suggestion was based on different computational (*in silico*) selection methods that included molecular similarity assessment, molecular fingerprint, docking studies, toxicity, ADMET, and DFT. The selected candidate showed various antiviral activities before. Further in vitro and in vivo biological studies have to be conducted to confirm the effect of **182** against SARS-Cov-2 nsp10 and its potential as an anti-COVID-19 drug.

## Figures and Tables

**Table 1:** Structural properties of the similar candidates with **SAM**.

Candidate	ALog p ^1^	M. Wt ^2^	HBA ^3^	HBD ^4^	Rotatable Bonds	Rings	Aromatic Rings	MFPSA ^5^	Minimum Distance
**7**	0.857	546.629	9	3	11	5	1	0.223	1.272
**19**	2.643	301.294	6	2	2	3	2	0.261	1.441
**33**	0.674	315.321	5	3	0	4	1	0.333	1.472
**44**	−4.182	194.206	5	6	2	1	0	0.597	1.379
**48**	−3.556	190.217	4	5	1	2	0	0.466	1.454
**71**	1.479	304.252	7	5	1	3	2	0.467	1.491
**77**	1.388	318.235	8	6	1	3	2	0.526	1.477
**82**	1.388	318.235	8	6	1	3	2	0.526	1.477
**83**	1.388	318.235	8	6	1	3	2	0.526	1.477
**85**	0.436	446.404	10	5	5	4	2	0.373	1.093
**102**	−0.729	330.287	9	5	5	2	1	0.457	0.491
**105**	−1.814	353.301	9	5	5	2	1	0.489	0.375
**120**	−0.396	422.34	11	8	2	4	2	0.533	0.652
**141**	0.207	321.216	9	5	4	2	2	0.563	0.632
**143**	0.007	477.352	13	7	7	3	2	0.536	0.565
**182**	−1.881	267.241	8	4	2	3	2	0.539	0.489
**183**	−2.396	243.217	7	4	2	2	0	0.545	0.747
**186**	1.045	371.273	4	3	0	4	2	0.339	1.039
**187**	1.045	371.273	4	3	0	4	2	0.339	1.039
**194**	0.253	193.203	5	4	2	1	1	0.501	0.964
**203**	−0.499	503.583	10	4	8	3	0	0.268	1.113
**204**	−0.091	517.61	10	3	9	3	0	0.237	1.229
**218**	0.536	293.283	7	3	1	4	3	0.444	0.903
**220**	−2.005	258.228	6	4	2	2	0	0.479	0.877
**221**	−2.451	244.201	6	4	2	2	0	0.525	0.876
**282**	−1.049	544.527	11	6	5	5	2	0.403	0.534
**284**	−1.308	251.242	7	3	2	3	2	0.482	0.406
**285**	−1.595	292.251	9	4	2	3	2	0.57	0.432
**301**	−1.614	251.242	7	3	3	3	2	0.48	0.364
**302**	−1.526	302.714	7	4	2	3	1	0.401	0.510
**SAM**	−4.254	399.445	9	4	7	3	2	0.483	

^1^ Partition coefficient, ^2^ Molecular weight, ^3^ Hydrogen bond acceptors, ^4^ Hydrogen bond donors, ^5^ Molecular fractional polar surface area.

**Table 2:** Fingerprint similarity between the tested candidates and **SAM**.

Comp.	Similarity	SA	SB	SC
**SAM**	1	237	0	0
**44**	0.503	159	79	78
**48**	0.423	110	23	127
**85**	0.423	200	236	37
**102**	0.497	149	63	88
**105**	0.529	165	75	72
**182**	0.717	160	−14	77
**220**	0.475	135	47	102
**221**	0.458	125	36	112
**282**	0.443	250	327	−13
**284**	0.685	150	−18	87
**285**	0.671	159	0	78
**301**	0.642	145	−11	92
**302**	0.552	139	15	98

**Table 3:** The calculated binding free energies of the examined candidates and **SAM** inside COVID-19 nsp10.

Comp.	∆G [Kcal/mol]	Comp.	∆G [Kcal/mol]
**44**	−18.65	**221**	−20.09
**48**	−21.15	**282**	−19.85
**85**	−19.32	**284**	−20.07
**102**	−18.98	**285**	−19.02
**105**	−20.01	**301**	−18.72
**182**	−21.10	**302**	−16.96
**220**	−21.17	**SAM**	−22.05

**Table 4:** Toxicity properties of candidates.

Comp.	FDA Rodent Carcinogenicity (Mouse-Female)	Carcinogenic Potency TD_50_ (Mouse) mg/kg Body Weight/Day	Rat Maximum Tolerated Dose (Feed) ^a^	Rat Oral LD_50_ ^a^	Rat Chronic LOAEL ^a^	Ocular Irritancy	Skin Irritancy
**48**	Non-Carcinogen	9.295	0.191	0.778	0.018	Severe	Mild
**182**	Non-Carcinogen	4.245	0.175	1.119	0.010	Moderate	Mild
**220**	Single-Carcinogen	67.851	0.095	6.173	0.009	Moderate	Mild
**221**	Single-Carcinogen	55.437	0.094	4.343	0.006	Moderate	Mild
**284**	Multi-Carcinogen	6.402	0.155	1.213	0.004	Moderate	Mild
Ribavirin	Non-Carcinogen	13.111	0.154	0.750	0.013	Mild	Mild

^a^ Unit = g/kg body weight.

**Table 5:** Spatial distribution of molecular orbitals for candidates **48** and **182**.

Name	Total Energy *	Binding Energy *	HOMO Energy *	LUMO Energy *	Dipole Mag	Band Gap Energy *
**48**	−664.379	−4.841	−0.366	−0.156	1.391	0.210
**182**	−955.658	−6.102	−0.195	−0.068	1.396	0.128
**SAM**	−1675.931	−8.815	−0.270	−0.174	3.631	0.097

* Unit = kcal/mol for all descriptors except Dipole Mag.

## Supplementary Materials

The following are available online, Figure S1: Chemical structures of the examined natural antiviral compounds, Table S1: Detailed toxicity report, in addition to the method (Molecular Similarity, Pharmacophore, Docking studies, ADMET studies, Toxicity studies and DFT studies).

## Sample Availability

Samples of the compounds are not available from the authors.

## References

[1] WHO WHO Coronavirus (COVID-19) Dashboard. https://covid19.who.int/.

[2] Prieto-Martínez F.D., López-López E., Juárez-Mercado K.E., Medina-Franco J.L. (2019). Computational drug design methods—Current and future perspectives. In Silico Drug Design.

[3] Nasser A.A., Eissa I.H., Oun M.R., El-Zahabi M.A., Taghour M.S., Belal A., Saleh A.M., Mehany A.B., Luesch H., Mostafa A.E. (2020). Discovery of new pyrimidine-5-carbonitrile derivatives as anticancer agents targeting EGFR WT and EGFR T790M. Org. Biomol. Chem..

[4] Abbass E.M., Khalil A.K., Mohamed M.M., Eissa I.H., El-Naggar A.M. (2020). Design, efficient synthesis, docking studies, and anticancer evaluation of new quinoxalines as potential intercalative Topo II inhibitors and apoptosis inducers. Bioorg. Chem..

[5] Alanazi M.M., Mahdy H.A., Alsaif N.A., Obaidullah A.J., Alkahtani H.M., Al-Mehizia A.A., Alsubaie S.M., Dahab M.A., Eissa I.H. (2021). New bis ([1,2,4][1,2,4][1,2,4][1,2,4][1,2,4][1,2,4][1,2,4][1,2,4][1,2,4][1,2,4][1,2,4][1,2,4][1,2,4][1,2,4][1,2,4][1,2,4][1,2,4][1,2,4][1,2,4] triazolo)[4,3-*a*:3′,4′-*c*] quinoxaline derivatives as VEGFR-2 inhibitors and apoptosis inducers: Design, synthesis, in silico studies, and anticancer evaluation. Bioorg. Chem..

[6] El-Helby A.-G.A., Sakr H., Ayyad R.R., Mahdy H.A., Khalifa M.M., Belal A., Rashed M., El-Sharkawy A., Metwaly A.M., Elhendawy M.A. (2020). Design, synthesis, molecular modeling, in vivo studies and anticancer activity evaluation of new phthalazine derivatives as potential DNA intercalators and topoisomerase II inhibitors. Bioorg. Chem..

[7] Eissa I.H., Ibrahim M.K., Metwaly A.M., Belal A., Mehany A.B., Abdelhady A.A., Elhendawy M.A., Radwan M.M., ElSohly M.A., Mahdy H.A. (2021). Design, molecular docking, in vitro, and in vivo studies of new quinazolin-4 (3H)-ones as VEGFR-2 inhibitors with potential activity against hepatocellular carcinoma. Bioorg. Chem..

[8] Abo-Ashour M.F., Eldehna W.M., Nocentini A., Bonardi A., Bua S., Ibrahim H.S., Elaasser M.M., Kryštof V., Jorda R., Gratteri P. (2019). 3-Hydrazinoisatin-based benzenesulfonamides as novel carbonic anhydrase inhibitors endowed with anticancer activity: Synthesis, in vitro biological evaluation and in silico insights. Eur. J. Med. Chem..

[9] Marrone T.J., Briggs A., James M., McCammon J.A. (1997). Structure-based drug design: Computational advances. Annu. Rev. Pharmacol. Toxicol..

[10] Li N., Wang Y., Li W., Li H., Yang L., Wang J., Mahdy H.A., Mehany A., Jaiash D.A., Santali E.Y. (2020). Screening of Some Sulfonamide and Sulfonylurea Derivatives as Anti-Alzheimer’s Agents Targeting BACE1 and PPARγ. J. Chem..

[11] Abdel-Aziz H.A., Eldehna W.M., Fares M., Al-Rashood S.T., Al-Rashood K.A., Abdel-Aziz M.M., Soliman D.H. (2015). Synthesis, biological evaluation and 2D-QSAR study of halophenyl bis-hydrazones as antimicrobial and antitubercular agents. Int. J. Mol. Sci..

[12] Kairys V., Baranauskiene L., Kazlauskiene M., Matulis D., Kazlauskas E. (2019). Binding affinity in drug design: Experimental and computational techniques. Expert Opin. Drug Discov..

[13] Al-Warhi T., El Kerdawy A.M., Aljaeed N., Ismael O.E., Ayyad R.R., Eldehna W.M., Abdel-Aziz H.A., Al-Ansary G.H. (2020). Synthesis, biological evaluation and in silico studies of certain oxindole–indole conjugates as anticancer CDK inhibitors. Molecules.

[14] El-Metwally S.A., Abou-El-Regal M.M., Eissa I.H., Mehany A.B., Mahdy H.A., Elkady H., Elwan A., Elkaeed E.B. (2021). Discovery of thieno [2,3-d] pyrimidine-based derivatives as potent VEGFR-2 kinase inhibitors and anti-cancer agents. Bioorg. Chem..

[15] Alanazi M.M., Eissa I.H., Alsaif N.A., Obaidullah A.J., Alanazi W.A., Alasmari A.F., Albassam H., Elkady H., Elwan A. (2021). Design, synthesis, docking, ADMET studies, and anticancer evaluation of new 3-methylquinoxaline derivatives as VEGFR-2 inhibitors and apoptosis inducers. J. Enzym. Inhib. Med. Chem..

[16] Alanazi M.M., Alaa E., Alsaif N.A., Obaidullah A.J., Alkahtani H.M., Al-Mehizia A.A., Alsubaie S.M., Taghour M.S., Eissa I.H. (2021). Discovery of new 3-methylquinoxalines as potential anti-cancer agents and apoptosis inducers targeting VEGFR-2: Design, synthesis, and in silico studies. J. Enzym. Inhib. Med. Chem..

[17] Alsaif N.A., Taghour M.S., Alanazi M.M., Obaidullah A.J., Al-Mehizia A.A., Alanazi M.M., Aldawas S., Elwan A., Elkady H. (2021). Discovery of new VEGFR-2 inhibitors based on bis ([1,2,4]triazolo)[4,3-*a*: 3′,4′-*c*] quinoxaline derivatives as anticancer agents and apoptosis inducers. J. Enzym. Inhib. Med. Chem..

[18] Alsaif N.A., Dahab M.A., Alanazi M.M., Obaidullah A.J., Al-Mehizia A.A., Alanazi M.M., Aldawas S., Mahdy H.A., Elkady H. (2021). New quinoxaline derivatives as VEGFR-2 inhibitors with anticancer and apoptotic activity: Design, molecular modeling, and synthesis. Bioorg. Chem..

[19] El-Adl K., Ibrahim M.-K., Alesawy M.S., Eissa I.H. (2021). [1,2,4]Triazolo [4,3-*c*]quinazoline and bis([1,2,4][1,2,4][1,2,4][1,2,4][1,2,4][1,2,4][1,2,4][1,2,4][1,2,4][1,2,4][1,2,4][1,2,4][1,2,4][1,2,4]triazolo)[4,3-*a*:4′,3′-*c*] quinazoline derived DNA intercalators: Design, synthesis, in silico ADMET profile, molecular docking and anti-proliferative evaluation studies. Bioorg. Med. Chem..

[20] March-Vila E., Pinzi L., Sturm N., Tinivella A., Engkvist O., Chen H., Rastelli G. (2017). On the integration of in silico drug design methods for drug repurposing. Front. Pharmacol..

[21] Zhang W., Pei J., Lai L. (2017). Computational multitarget drug design. J. Chem. Inf. Model..

[22] Youssef M.I., Zhou Y., Eissa I.H., Wang Y., Zhang J., Jiang L., Hu W., Qi J., Chen Z. (2020). Tetradecyl 2,3-dihydroxybenzoate alleviates oligodendrocyte damage following chronic cerebral hypoperfusion through IGF-1 receptor. Neurochem. Int..

[23] Zhong F., Xing J., Li X., Liu X., Fu Z., Xiong Z., Lu D., Wu X., Zhao J., Tan X. (2018). Artificial intelligence in drug design. Sci. China Life Sci..

[24] Hagras M., El Deeb M.A., Elzahabi H.S., Elkaeed E.B., Mehany A.B., Eissa I.H. (2021). Discovery of new quinolines as potent colchicine binding site inhibitors: Design, synthesis, docking studies, and anti-proliferative evaluation. J. Enzym. Inhib. Med. Chem..

[25] Eissa I.H., Dahab M.A., Ibrahim M.K., Alsaif N.A., Alanazi A., Eissa S.I., Mehany A.B., Beauchemin A.M. (2021). Design and discovery of new antiproliferative 1,2,4-triazin-3 (2H)-ones as tubulin polymerization inhibitors targeting colchicine binding site. Bioorg. Chem..

[26] Eissa I.H., El-Helby A.-G.A., Mahdy H.A., Khalifa M.M., Elnagar H.A., Mehany A.B., Metwaly A.M., Elhendawy M.A., Radwan M.M., ElSohly M.A. (2020). Discovery of new quinazolin-4 (3H)-ones as VEGFR-2 inhibitors: Design, synthesis, and anti-proliferative evaluation. Bioorg. Chem..

[27] El-Adl K., El-Helby A.-G.A., Ayyad R.R., Mahdy H.A., Khalifa M.M., Elnagar H.A., Mehany A.B., Metwaly A.M., Elhendawy M.A., Radwan M.M. (2021). Design, synthesis, and anti-proliferative evaluation of new quinazolin-4 (3H)-ones as potential VEGFR-2 inhibitors. Bioorg. Med. Chem..

[28] Hopfinger A. (1985). Computer-assisted drug design. J. Med. Chem..

[29] Jalmakhanbetova R.I., Suleimen Y.M., Oyama M., Elkaeed E.B., Eissa I., Suleimen R.N., Metwaly A.M., Ishmuratova M.Y. (2021). Isolation and In Silico Anti-COVID-19 Main Protease (Mpro) Activities of Flavonoids and a Sesquiterpene Lactone from Artemisia sublessingiana. J. Chem..

[30] Al-Karmalawy A.A., Dahab M.A., Metwaly A.M., Elhady S.S., Elkaeed E.B., Eissa I.H., Darwish K.M. (2021). Molecular Docking and Dynamics Simulation Revealed the Potential Inhibitory Activity of ACEIs Against SARS-CoV-2 Targeting the hACE2 Receptor. Front. Chem..

[31] Alesawy M.S., Abdallah A.E., Taghour M.S., Elkaeed E.B., Eissa I.H., Metwaly A.M. (2021). In Silico Studies of Some Isoflavonoids as Potential Candidates against COVID-19 Targeting Human ACE2 (hACE2) and Viral Main Protease (Mpro). Molecules.

[32] El-Demerdash A., Metwaly A.M., Hassan A., El-Aziz A., Mohamed T., Elkaeed E.B., Eissa I.H., Arafa R.K., Stockand J.D. (2021). Comprehensive virtual screening of the antiviral potentialities of marine polycyclic guanidine alkaloids against SARS-CoV-2 (COVID-19). Biomolecules.

[33] Metwaly A.M., Ghoneim M.M., Eissa I.H., Elsehemy I.A., Mostafa A.E., Hegazy M.M., Afifi W.M., Dou D. (2021). Traditional ancient Egyptian medicine: A review. Saudi J. Biol. Sci..

[34] Han X., Yang Y., Metwaly A.M., Xue Y., Shi Y., Dou D. (2019). The Chinese herbal formulae (Yitangkang) exerts an antidiabetic effect through the regulation of substance metabolism and energy metabolism in type 2 diabetic rats. J. Ethnopharmacol..

[35] Metwaly A.M., Zhu L., Huang L., Dou D. (2019). Black ginseng and its saponins: Preparation, phytochemistry and pharmacological effects. Molecules.

[36] Wang Y.-M., Ran X.-K., Riaz M., Yu M., Cai Q., Dou D.-Q., Metwaly A.M., Kang T.-G., Cai D.-C. (2019). Chemical constituents of stems and leaves of *Tagetespatula* L. and its fingerprint. Molecules.

[37] Metwaly A. (2019). Comparative biological evaluation of four endophytic fungi isolated from nigella sativa seeds. Al-Azhar J. Pharm. Sci..

[38] Metwaly A.M., Wanas A.S., Radwan M.M., Ross S.A., ElSohly M.A. (2017). New α-Pyrone derivatives from the endophytic fungus *Embellisia* sp.. Med. Chem. Res..

[39] Metwaly A.M., Kadry H.A., Atef A., Mohammad A.-E.I., Ma G., Cutler S.J., Ross S.A. (2014). Nigrosphaerin A a new isochromene derivative from the endophytic fungus *Nigrospora sphaerica*. Phytochem. Lett..

[40] Metwaly A.M., Fronczek F.R., Ma G., Kadry H.A., Atef A., Mohammad A.-E.I., Cutler S.J., Ross S.A. (2014). Antileukemic α-pyrone derivatives from the endophytic fungus *Alternaria phragmospora*. Tetrahedron Lett..

[41] Imieje V.O., Zaki A.A., Metwaly A.M., Mostafa A.E., Elkaeed E.B., Falodun A. (2021). Comprehensive In Silico Screening of the Antiviral Potentialities of a New Humulene Glucoside from *Asteriscus hierochunticus* against SARS-CoV-2. J. Chem..

[42] Zhanzhaxina A., Suleimen Y., Metwaly A.M., Eissa I.H., Elkaeed E.B., Suleimen R., Ishmuratova M., Akatan K., Luyten W. (2021). In Vitro and In Silico Cytotoxic and Antibacterial Activities of a Diterpene from *Cousinia alata* Schrenk. J. Chem..

[43] Imieje V.O., Zaki A.A., Metwaly A.M., Eissa I.H., Elkaeed E.B., Ali Z., Khan I.A., Falodun A. (2021). Antileishmanial Derivatives of Humulene from *Asteriscus hierochunticus* with in silico Tubulin Inhibition Potential. Rec. Nat. Prod..

[44] Jalmakhanbetova R., Elkaeed E.B., Eissa I.H., Metwaly A.M., Suleimen Y.M. (2021). Synthesis and Molecular Docking of Some Grossgemin Amino Derivatives as Tubulin Inhibitors Targeting Colchicine Binding Site. J. Chem..

[45] Suleimen Y.M., Metwaly A.M., Mostafa A.E., Elkaeed E.B., Liu H.-W., Basnet B.B., Suleimen R.N., Ishmuratova M.Y., Turdybekov K.M., Van Heсke K. (2021). Isolation, Crystal Structure, and In Silico Aromatase Inhibition Activity of Ergosta-5,22-dien-3β-ol from the Fungus *Gyromitra esculenta*. J. Chem..

[46] Ghoneim M.M., Afifi W.M., Ibrahim M., Elagawany M., Khayat M.T., Aboutaleb M.H., Metwaly A.M. (2019). Biological evaluation and molecular docking study of metabolites from *Salvadora persica* L. Growing in Egypt. Pharmacogn. Mag..

[47] Liu L., Luo S., Yu M., Metwaly A.M., Ran X., Ma C., Dou D., Cai D. (2020). Chemical Constituents of Tagetes patula and Their Neuroprotecting Action. Nat. Prod. Commun..

[48] Metwaly A.M., Ghoneim M.M., Musa A. (2015). Two new antileishmanial diketopiperazine alkaloids from the endophytic fungus *Trichosporum* sp.. Derpharmachemica.

[49] Yassin A.M., El-Deeb N.M., Metwaly A.M., El Fawal G.F., Radwan M.M., Hafez E.E. (2017). Induction of apoptosis in human cancer cells through extrinsic and intrinsic pathways by *Balanites aegyptiaca* furostanol saponins and saponin-coated silvernanoparticles. Appl. Biochem. Biotechnol..

[50] Sharaf M.H., El-Sherbiny G.M., Moghannem S.A., Abdelmonem M., Elsehemy I.A., Metwaly A.M., Kalaba M.H. (2021). New combination approaches to combat methicillin-resistant *Staphylococcus aureus* (MRSA). Sci. Rep..

[51] Lin S., Chen H., Chen Z., Yang F., Ye F., Zheng Y., Yang J., Lin X., Sun H., Wang L. (2021). Crystal structure of SARS-CoV-2 nsp10 bound to nsp14-ExoN domain reveals an exoribonuclease with both structural and functional integrity. Nucleic Acids Res..

[52] Lin S., Chen H., Ye F., Chen Z., Yang F., Zheng Y., Cao Y., Qiao J., Yang S., Lu G. (2020). Crystal structure of SARS-CoV-2 nsp10/nsp16 2′-*O*-methylase and its implication on antiviral drug design. Signal Transduct. Target. Ther..

[53] Tazikeh-Lemeski E., Moradi S., Raoufi R., Shahlaei M., Janlou M.A.M., Zolghadri S. (2020). Targeting SARS-COV-2 non-structural protein 16: A virtual drug repurposing study. J. Biomol. Struct. Dyn..

[54] Yadav R., Parihar R.D., Dhiman U., Dhamija P., Kumar S. (2020). Docking of fda approved drugs targeting nsp-16, n-protein and main protease of sars-cov-2 as dual inhibitors. Biointerface Res. Appl. Chem..

[55] Parida P.K., Paul D., Chakravorty D. (2021). Nature’s therapy for COVID-19: Targeting the vital non-structural proteins (NSP) from SARS-CoV-2 with phytochemicals from Indian medicinal plants. Phytomed. Plus.

[56] Nasser M., Salim N., Hamza H., Saeed F., Rabiu I. (2021). Improved deep learning based method for molecular similarity searching using stack of deep belief networks. Molecules.

[57] Turchi M., Cai Q., Lian G. (2019). An evaluation of in-silico methods for predicting solute partition in multiphase complex fluids–A case study of octanol/water partition coefficient. Chem. Eng. Sci..

[58] Sullivan K.M., Enoch S.J., Ezendam J., Sewald K., Roggen E.L., Cochrane S. (2017). An adverse outcome pathway for sensitization of the respiratory tract by low-molecular-weight chemicals: Building evidence to support the utility of in vitro and in silico methods in a regulatory context. Applied In Vitro Toxicology.

[59] Altamash T., Amhamed A., Aparicio S., Atilhan M. (2020). Effect of hydrogen bond donors and acceptors on CO_2_ absorption by deep eutectic solvents. Processes.

[60] Wan Y., Tian Y., Wang W., Gu S., Ju X., Liu G. (2018). In silico studies of diarylpyridine derivatives as novel HIV-1 NNRTIs using docking-based 3D-QSAR, molecular dynamics, and pharmacophore modeling approaches. RSC Adv..

[61] Escamilla-Gutiérrez A., Ribas-Aparicio R.M., Córdova-Espinoza M.G., Castelán-Vega J.A. (2021). In silico strategies for modeling RNA aptamers and predicting binding sites of their molecular targets. Nucleosides Nucleotides Nucleic Acids.

[62] Kaushik A.C., Kumar A., Bharadwaj S., Chaudhary R., Sahi S. (2018). Ligand-Based Approach for In-silico Drug Designing. Bioinformatics Techniques for Drug Discovery.

[63] Zhang H., Ren J.-X., Ma J.-X., Ding L. (2019). Development of an in silico prediction model for chemical-induced urinary tract toxicity by using naïve Bayes classifier. Mol. Divers..

[64] Willett P. (2006). Similarity-based virtual screening using 2D fingerprints. Drug Discov. Today.

[65] Ieritano C., Campbell J.L., Hopkins W.S. (2021). Predicting differential ion mobility behaviour in silico using machine learning. Analyst.

[66] Taha M., Ismail N.H., Ali M., Rashid U., Imran S., Uddin N., Khan K.M. (2017). Molecular hybridization conceded exceptionally potent quinolinyl-oxadiazole hybrids through phenyl linked thiosemicarbazide antileishmanial scaffolds: In silico validation and SAR studies. Bioorg. Chem..

[67] Chu H., He Q.-X., Wang J., Hu Y., Wang Y.-Q., Lin Z.-H. (2020). In silico design of novel benzohydroxamate-based compounds as inhibitors of histone deacetylase 6 based on 3D-QSAR, molecular docking, and molecular dynamics simulations. New J. Chem..

[68] Opo F.A.D.M., Rahman M.M., Ahammad F., Ahmed I., Bhuiyan M.A., Asiri A.M. (2021). Structure based pharmacophore modeling, virtual screening, molecular docking and ADMET approaches for identification of natural anti-cancer agents targeting XIAP protein. Sci. Rep..

[69] Fleming I. (1977). Frontier Orbitals and Organic Chemical Reactions.

[70] El-Nahass M., Kamel M., El-Deeb A., Atta A., Huthaily S. (2011). Ab initio HF, DFT and experimental (FT-IR) investigation of vibrational spectroscopy of PN, N-dimethylaminobenzylidenemalononitrile (DBM). Spectrochim. Acta Part. A Mol. Biomol. Spectrosc..

[71] Suhasini M., Sailatha E., Gunasekaran S., Ramkumaar G. (2015). Vibrational and electronic investigations, thermodynamic parameters, HOMO and LUMO analysis on Lornoxicam by density functional theory. J. Mol. Struct..

[72] Bitencourt-Ferreira G., de Azevedo Junior W.F. (2021). Electrostatic Potential Energy in Protein-Drug Complexes. Curr. Med. Chem..

[73] Matin M.M., Hasan M.S., Uzzaman M., Bhuiyan M.M.H., Kibria S.M., Hossain M.E., Roshid M.H. (2020). Synthesis, spectroscopic characterization, molecular docking, and ADMET studies of mannopyranoside esters as antimicrobial agents. J. Mol. Struct..

[74] Whitley R.J., Alford C.A., Hirsch M.S., Schooley R.T., Luby J.P., Aoki F.Y., Hanley D., Nahmias A.J., Soong S.-J., NIAID Collaborative Antiviral Study Group (1986). Vidarabine versus acyclovir therapy in herpes simplex encephalitis. N. Engl. J. Med..

[75] Whitley R.J., Nahmias A.J., Soong S.-J., Galasso G.G., Fleming C.L., Alford C.A., Connor J., Bryson Y., Linnemann C. (1980). Vidarabine therapy of neonatal herpes simplex virus infection. Pediatrics.

[76] Pollard R.B., Smith J.L., Neal E.A., Gregory P.B., Merigan T.C., Robinson W.S. (1978). Effect of vidarabine on chronic hepatitis B virus infection. JAMA.

[77] Miwa N., Kurosaki K., Yoshida Y., Kurokawa M., Saito S., Shiraki K. (2005). Comparative efficacy of acyclovir and vidarabine on the replication of varicella-zoster virus. Antivir. Res..

[78] Chapman C., Flower A., Durrant S. (1991). The use of vidarabine in the treatment of human polyomavirus associated acute haemorrhagic cystitis. Bone Marrow Transplant..

[79] Kurosaki K., Miwa N., Yoshida Y., Kurokawa M., Kurimoto M., Endo S., Shiraki K. (2004). Therapeutic basis of vidarabine on adenovirus-induced haemorrhagic cystitis. Antivir. Chem. Chemother..

[80] Kimura H., Morita M., Tsuge I., Hoshino Y., Tanaka N., Ito Y., Morishima T. (2001). Vidarabine therapy for severe chronic active Epstein–Barr virus infection. J. Pediatric Hematol. Oncol..

[81] Yousef R., Sakr H., Eissa I., Mehany A., Metwaly A., Elhendawy M.A., Radwan M., ElSohly M.A., Abulkhair H.S., El-Adl K. (2021). New quinoxaline-2 (1H)-ones as potential VEGFR-2 inhibitors: Design, synthesis, molecular docking, ADMET profile and anti-proliferative evaluations. New J. Chem..

[82] Amer H.H., Alotaibi S.H., Trawneh A.H., Metwaly A.M., Eissa I.H. (2021). Anticancer activity, spectroscopic and molecular docking of some new synthesized sugar hydrazones, Arylidene and α-Aminophosphonate derivatives. Arab. J. Chem..

[83] Alesawy M.S., Al-Karmalawy A.A., Elkaeed E.B., Alswah M., Belal A., Taghour M.S., Eissa I.H. (2021). Design and discovery of new 1,2,4-triazolo [4,3-*c*] quinazolines as potential DNA intercalators and topoisomerase II inhibitors. Arch. Pharm..

[84] Parmar D.R., Soni J.Y., Guduru R., Rayani R.H., Kusurkar R.V., Vala A.G., Talukdar S.N., Eissa I.H., Metwaly A.M., Khalil A. (2021). Discovery of new anticancer thiourea-azetidine hybrids: Design, synthesis, in vitro antiproliferative, SAR, in silico molecular docking against VEGFR-2, ADMET, toxicity, and DFT studies. Bioorg. Chem..

