# Supplementary material for: In Silico Exploration of Potential Natural Inhibitors against SARS-Cov-2 nsp10
Source: Molecules. 2021 Oct 12;26(20):6151. doi: 10.3390/molecules26206151 (PMC8539059; doi:10.3390/molecules26206151)
Supplement: Supplementary file 1 [file molecules-26-06151-s001.zip › molecules-1394222-SI.pdf]

In silico Exploration of Potential Natural Inhibitors Against SARS-Cov-2 nsp10

Ibrahim. H. Eissa[a]*, Mohamed M. Khalifa [a], Eslam B. Elkaeed[b], Elsayed E. Hafez[c], Aisha A. Alsfouk[d], Ahmed M. Metwaly[e]*

[a] Pharmaceutical Medicinal Chemistry & Drug Design Department, Faculty of Pharmacy (Boys), Al-Azhar University, Cairo11884, Egypt.

[b] Department of Pharmaceutical Sciences, College of Pharmacy, Almaarefa University, Ad Diriyah 13713, Riyadh, Saudi Arabia.

[C] Department of Plant Protection and Biomolecular Diagnosis, ALCRI, City of Scientific Research and Technological Applications, New Borg El-Arab City, Alexandria, 21934, Egypt

[d] Department of Pharmaceutical Sciences, College of Pharmacy, Princess Nourah bint Abdulrahman University, Riyadh, Saudi Arabia.

[e] Pharmacognosy and Medicinal Plants Department, Faculty of Pharmacy (Boys), Al-Azhar University, Cairo 11884, Egypt.

Figure S1. Chemical structures of the examined natural antiviral compounds	
The most similar compounds with the co-crystallized ligand (SAM)	
Method	Molecular Similarity
	Pharmacophore
	Docking studies
	ADMET studies
	Toxicity studies
	DFT studies
Toxicity report	

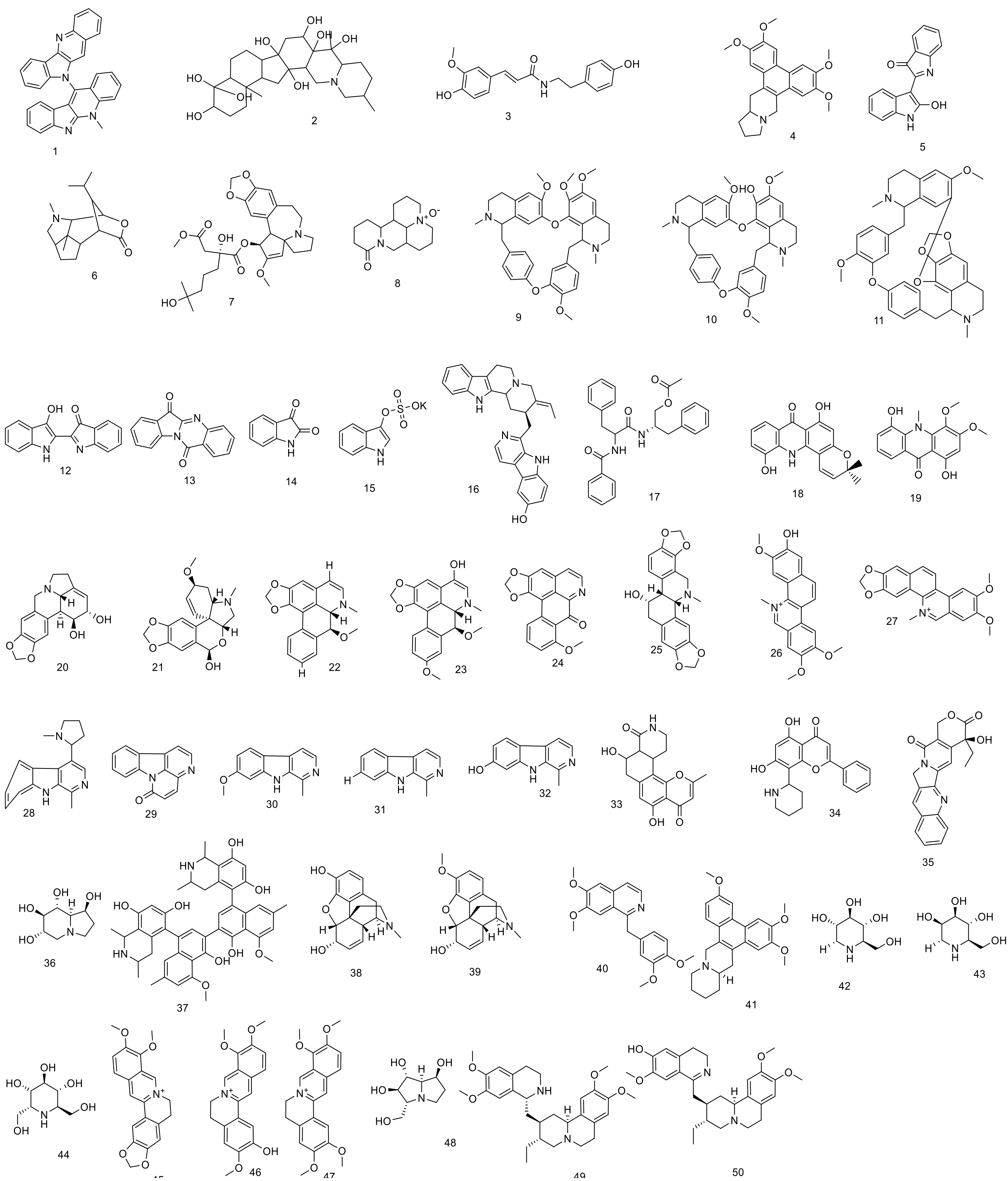

Figure S1. Chemical structures of the examined 310 natural antiviral compounds

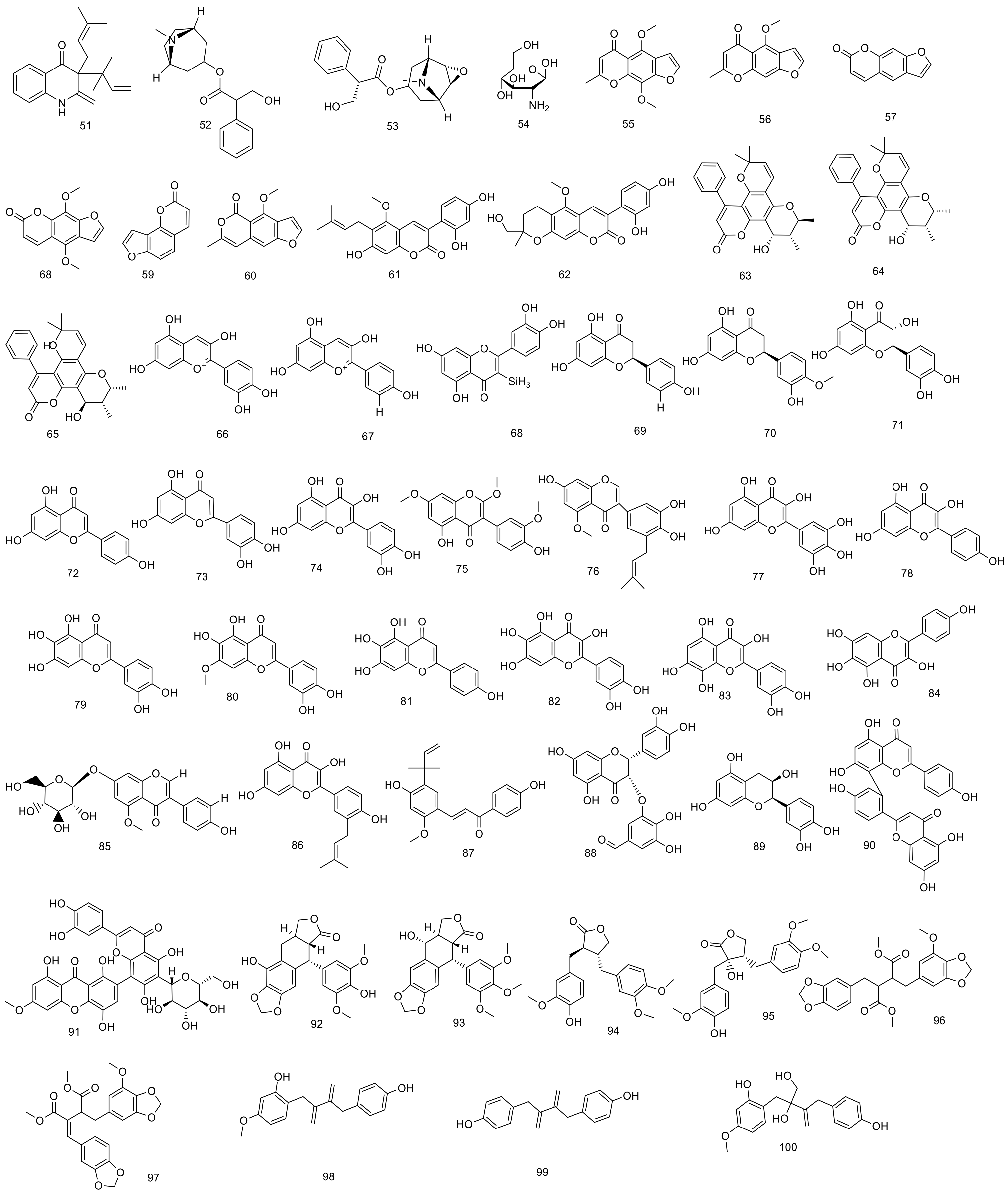

Figure S1. Chemical structures of the examined 310 natural antiviral compounds

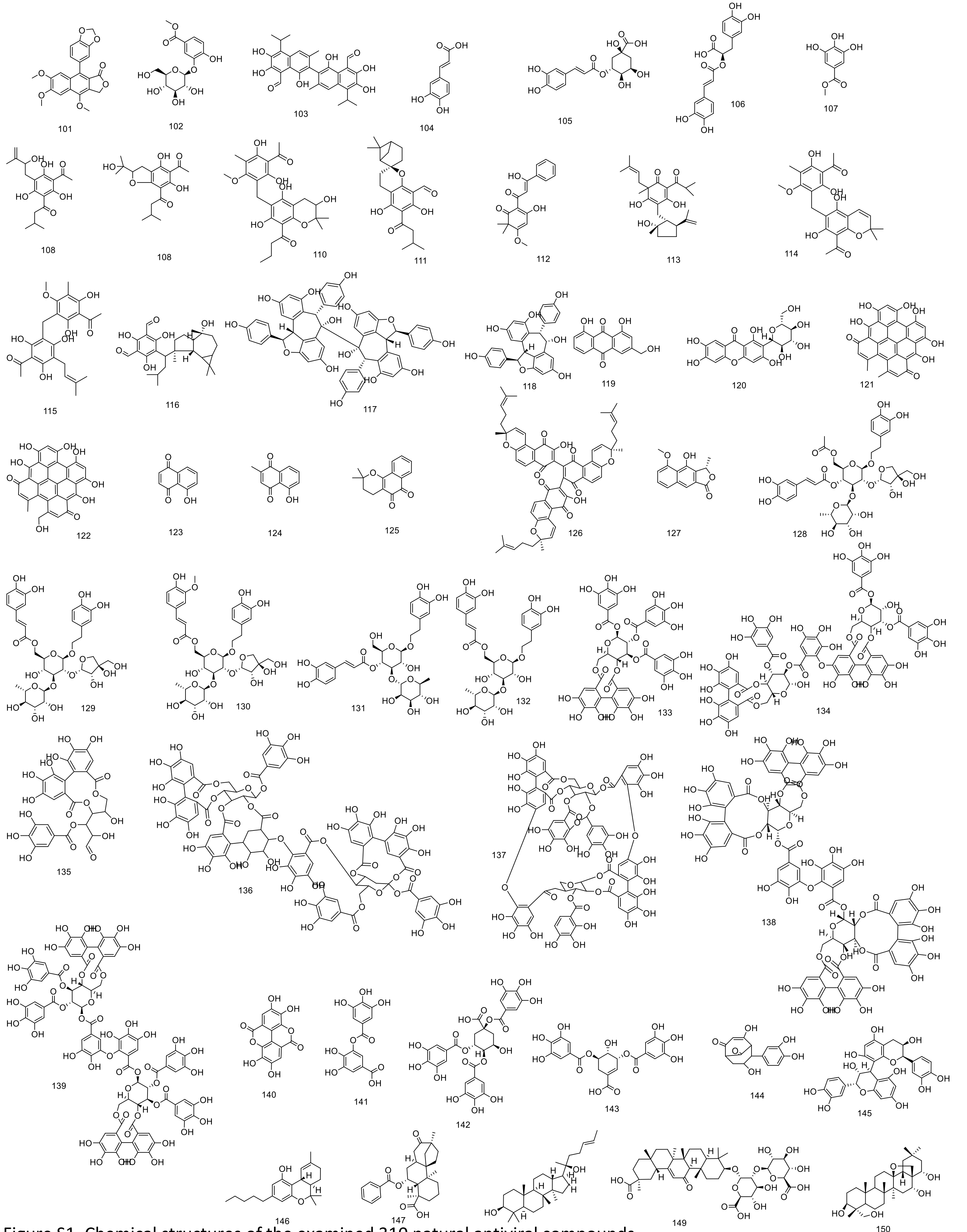

Figure S1. Chemical structures of the examined 310 natural antiviral compounds

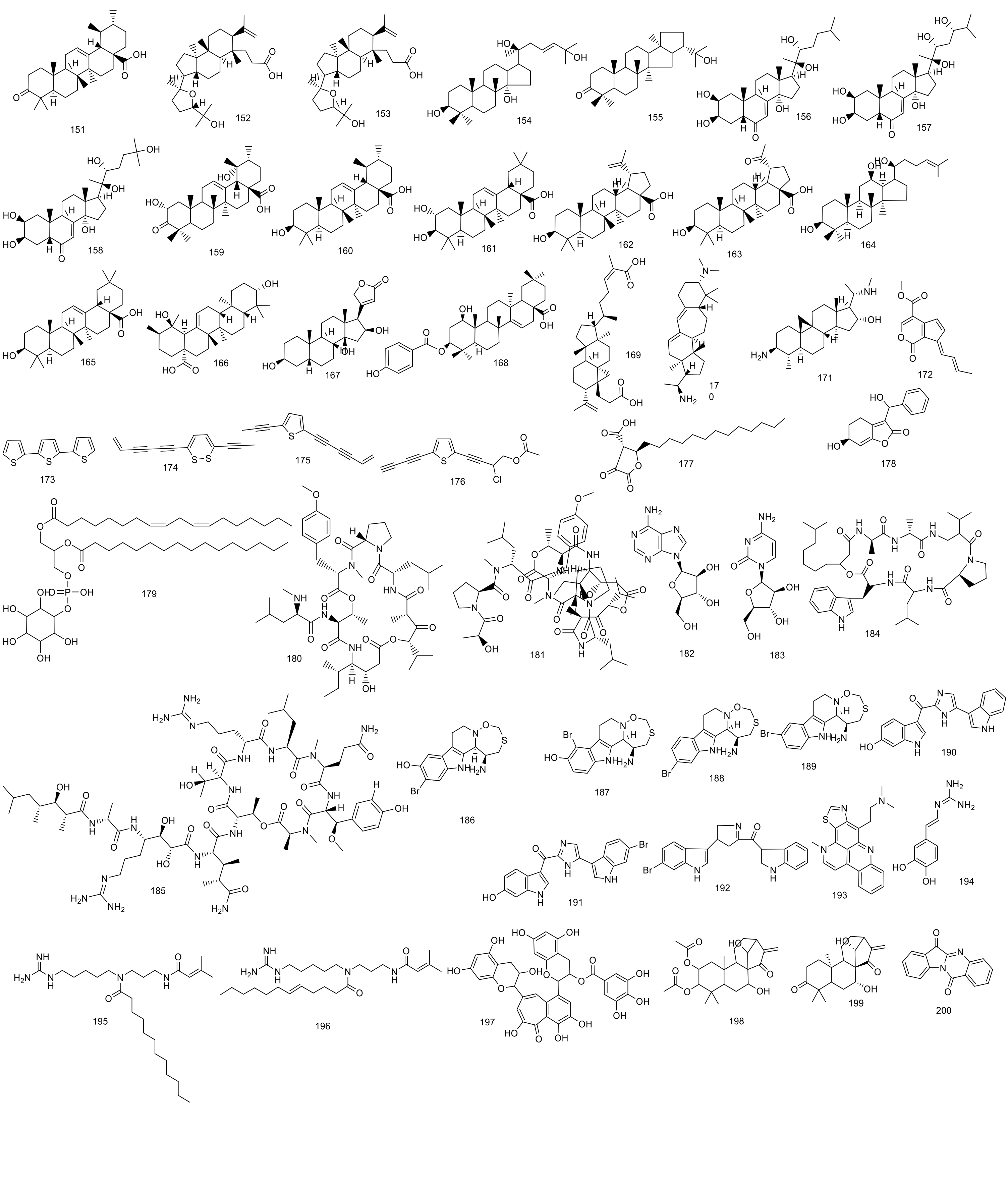

Figure S1. Chemical structures of the examined 310 natural antiviral compounds

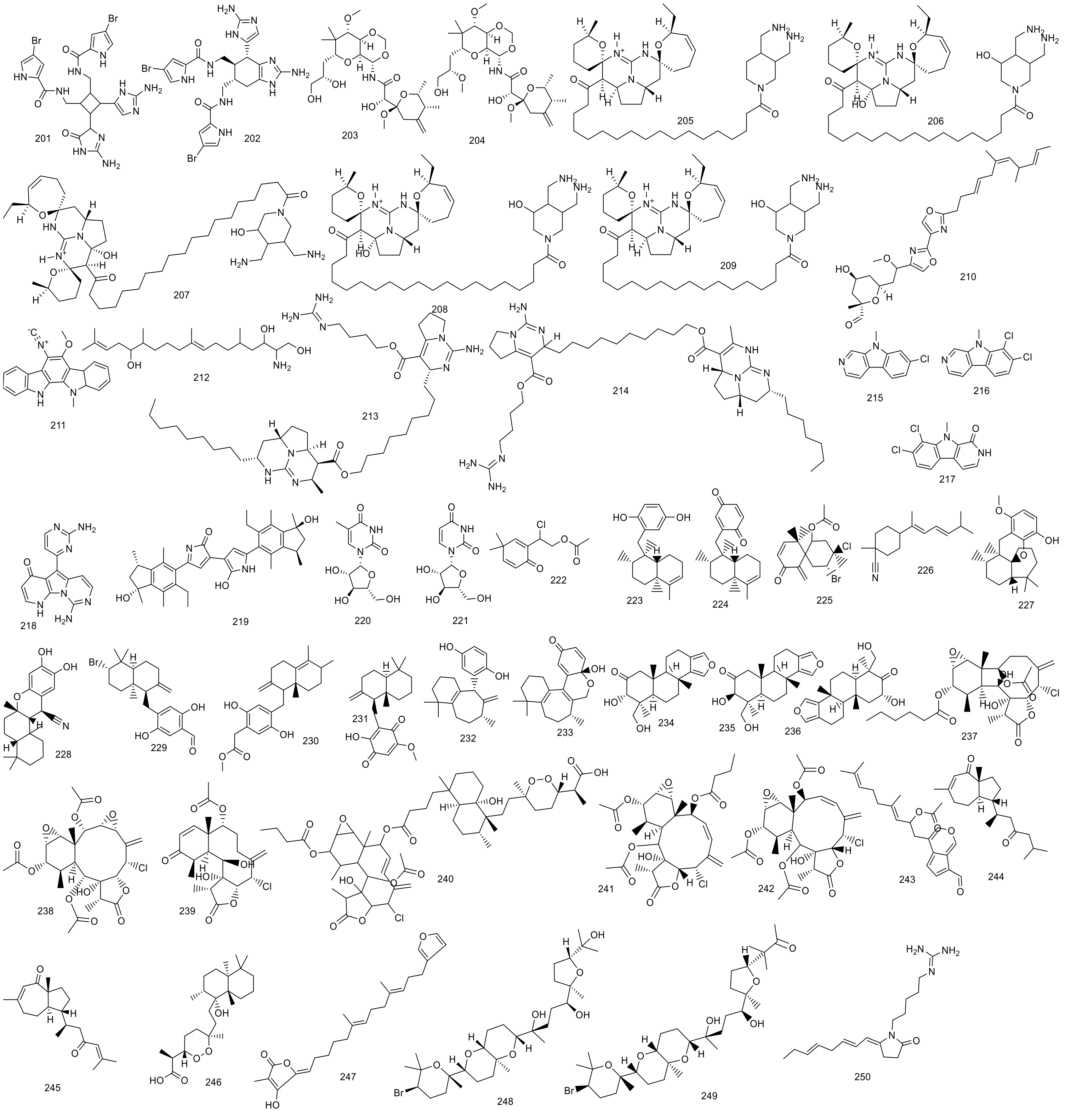

Figure S1. Chemical structures of the examined 310 natural antiviral compounds

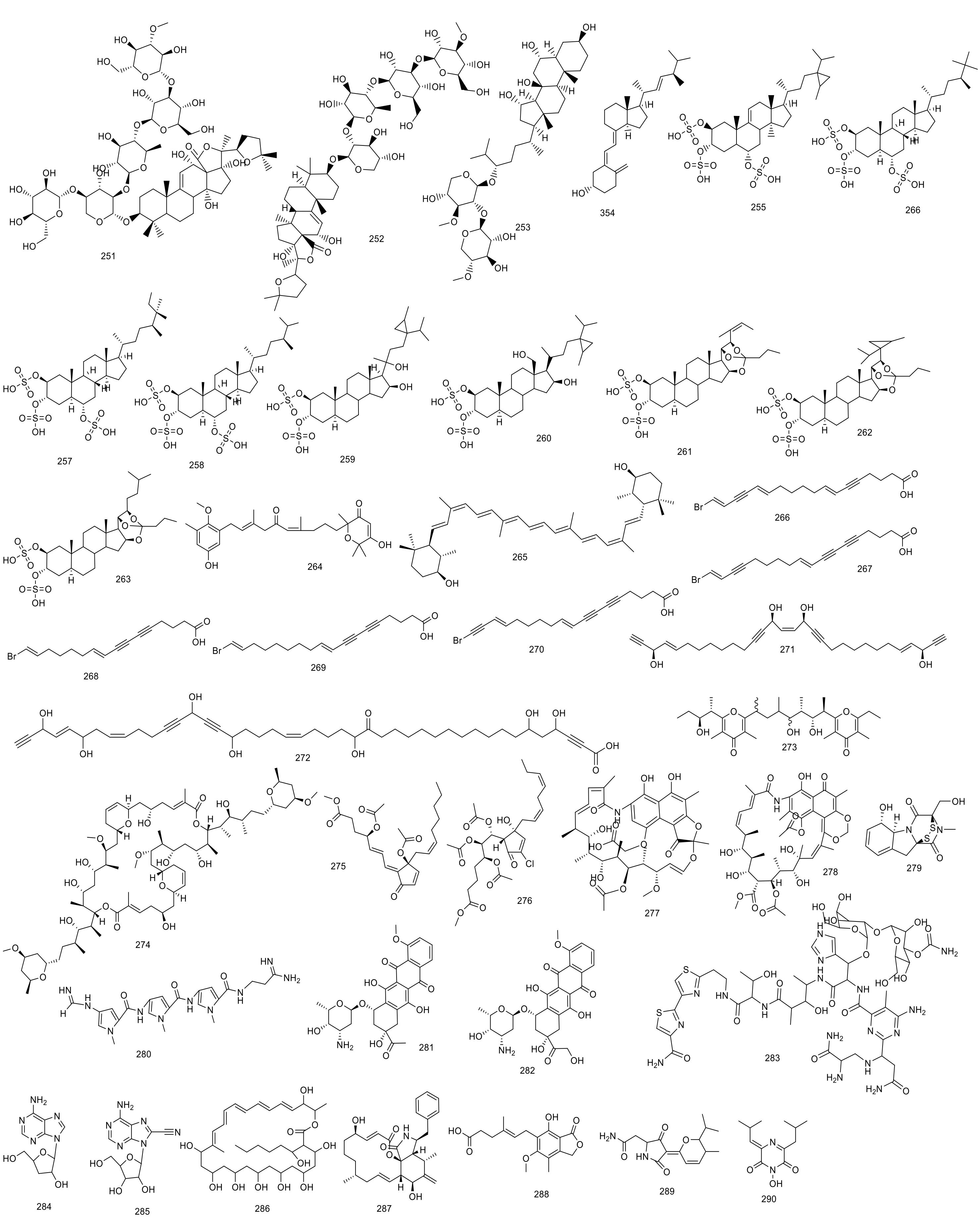

Figure S1. Chemical structures of the examined 310 natural antiviral compounds

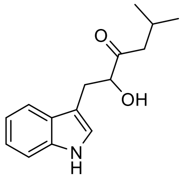

291

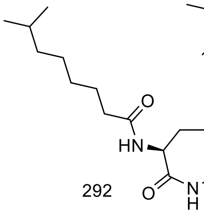

292

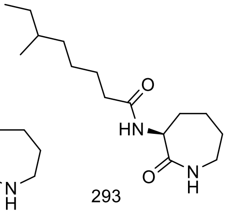

293

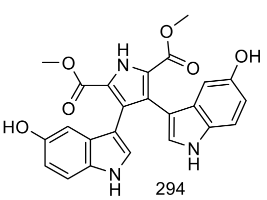

294

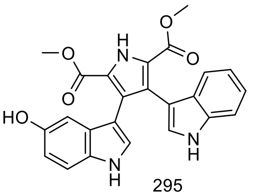

295

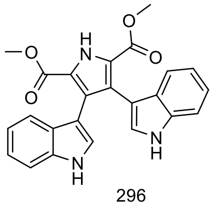

296

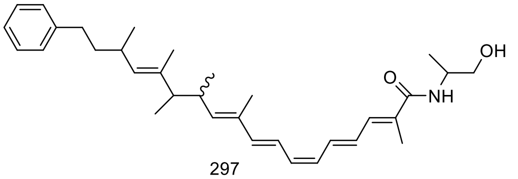

297

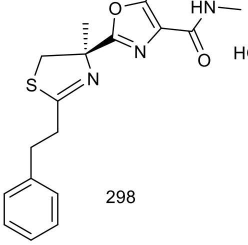

298

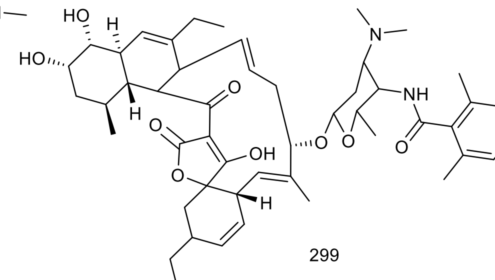

299

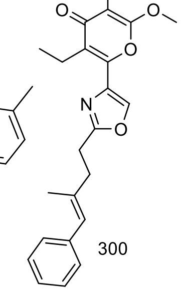

300

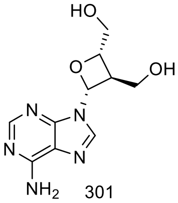

301

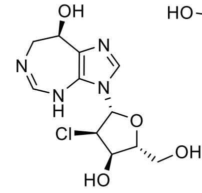

302

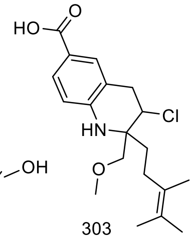

303

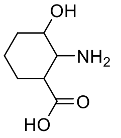

304

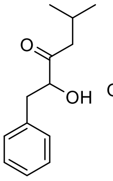

305

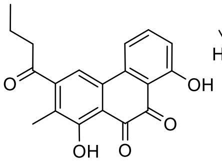

306

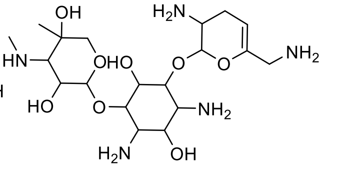

307

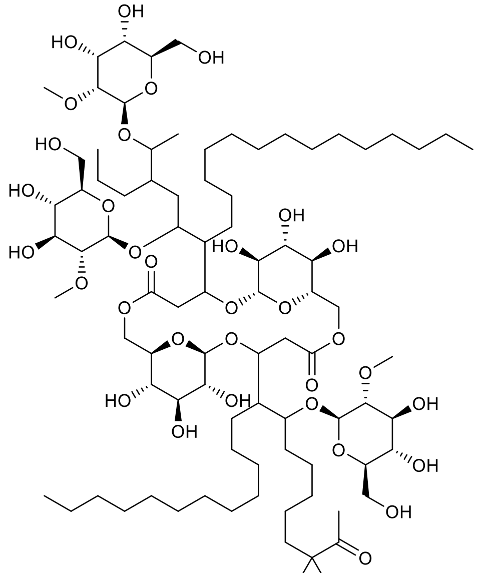

308

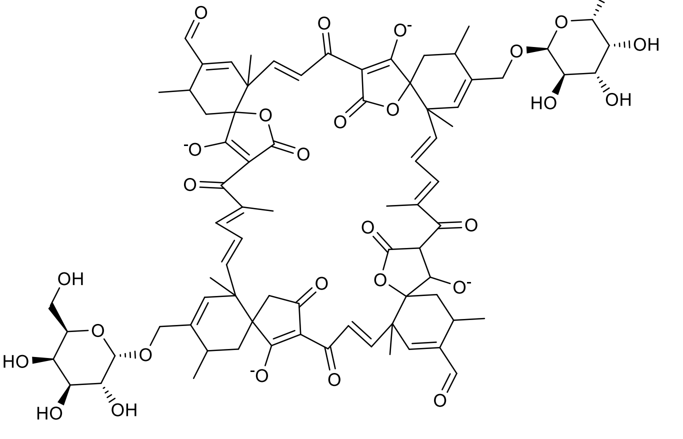

309

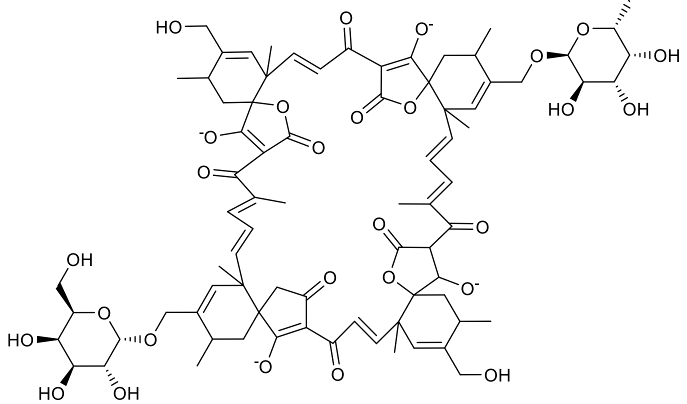

310

Figure S1. Chemical structures of the examined 310 natural antiviral compounds

The most similar compounds with the co-crystallized ligand (SAM) of SARS-CoV-2 nonstructural protein (nsp10) (PDB ID: 6W4H)

**Comp. No**	**Name**	**Type**	**Source**	**Antiviral activity**
**44**	α-Homonojirimycin	Azasugar	*Omphalea diandra* [1]	Anti-HIV-1 activity via being α-glucosidase inhibitors [2,3]
**48**	australine	Pyrrolizidine alkaloid	*Castanospermum australe* [4]	aAnti-HIV-1 activity via being α-glucosidase inhibitors [2,3]
**85**	5-O-Methylgenistein-7-glucoside	isoflavonoid	*Ulex europaeus* [5]	Exhibited inhibition potential against HSV-1 [5]
**102**	woodorien	Benzoic acid derivative	*Woodwardia orientalis* [6]	Exhibited inhibition potential against HSV-1 [6]
**105**	chlorogenic acid	Phenolic compound	Several plants including coffee beans[7]	Inhibited effectively HBV (*in vivo* and *in vitro*) [8], influenza and parainfluenza viruses [9], enterovirus [10]. Surprisingly, it regulated the innate immune against Gama coronavirus through the positive regulation of the signaling pathways of MDA5, TLR7, and NF-κB [11]
**220**	Spongothymidine	Nucleosides	*Cryptotethya crypta* [12]	The modification of 220 resulted in the sugar-modified nucleoside analog, vidarabine(182) [13].<br>Vidarabine was very active against HSV [14,15], HBV [16], varicella-zoster [17], human polyomavirus [18], adenovirus [19], and Epstein–Barr [20] virus infections
**221**	Spongouridine			
**182**	Vidarabine			
**282**	Doxorubicin	Anthracycline glycosides	*Streptomyces peucetius* [21]	Was very active against avian myeloblastosis in addition to murine and rauscher leukemia viruses [22,23]
**284**	Cordyceptin	adenosine derivative	*Cordyceps militaris*[24].	Cordyceptin showed promising activities against influenza virus (*in vivo*) [25], HIV via inhibition of reverse transcriptase [26] Rotavirus through the induction of type I interferon [27], and Epstein-Barr virus [28]
**285**	Toyocamycin	adenosine derivative	*Streptomyces toyocaensis* [29]	Stopped the replication of vesicular stomatitis virus [30] and stabilized the adenovirus nuclear RNA [31]
**301**	Oxetanocin A		*Bacillus megaterium* [32]	Was active against HIV [33] HBV [34] varicella-zoster virus [35]
**302**	Adechlorin		*Actinomadura* sp[36]	Was active against various viruses through the inhibition of adenosine deaminase [36].

1. Kite, G.C.; Fellows, L.E.; Fleet, G.W.; Liu, P.S.; Scofield, A.M.; Smith, N.G. α-Homonojirimycin [2, 6-dideoxy-2, 6-imino-d-glycero-l-gulo-heptitol] from Omphalea diandra L.: isolation and glucosidase inhibtion. *Tetrahedron letters* **1988**, *29*, 6483-6485.
2. Cos, P.; Maes, L.; Vlietinck, A.; Pieters, L. Plant-derived leading compounds for chemotherapy of human immunodefiency virus (HIV) infection–an update (1998–2007). *Planta medica* **2008**, *74*, 1323-1337.
3. Vlietinck, A.; De Bruyne, T.; Apers, S.; Pieters, L. Plant-derived leading compounds for chemotherapy of human immunodeficiency virus (HIV) infection. *Planta medica* **1998**, *64*, 97-109.
4. Molyneux, R.J.; Benson, M.; Wong, R.Y.; Tropea, J.E.; Elbein, A.D. Australine, a novel pyrrolizidine alkaloid glucosidase inhibitor from Castanospermum australe. *Journal of Natural Products* **1988**, *51*, 1198-1206.
5. El Sayed, K.A. Natural products as antiviral agents. In *Studies in Natural Products Chemistry*, Elsevier: 2000; Vol. 24, pp. 473-572.
6. XU, H.-X.; KADOTA, S.; KUROKAWA, M.; SHIRAKI, K.; MATSUMOTO, T.; NAMBA, T. Isolation and structure of woodorien, a new glucoside having antiviral activity, from Woodwardia orientalis. *Chemical and pharmaceutical bulletin* **1993**, *41*, 1803-1806.
7. Moores, R.; McDermott, D.L.; Wood, T. Determination of chlorogenic acid in coffee. *Analytical Chemistry* **1948**, *20*, 620-624.
8. Wang, G.-F.; Shi, L.-P.; Ren, Y.-D.; Liu, Q.-F.; Liu, H.-F.; Zhang, R.-J.; Li, Z.; Zhu, F.-H.; He, P.-L.; Tang, W. Anti-hepatitis B virus activity of chlorogenic acid, quinic acid and caffeic acid in vivo and in vitro. *Antiviral research* **2009**, *83*, 186-190.
9. Ding, Y.; Cao, Z.; Cao, L.; Ding, G.; Wang, Z.; Xiao, W. Antiviral activity of chlorogenic acid against influenza A (H1N1/H3N2) virus and its inhibition of neuraminidase. *Scientific reports* **2017**, *7*, 1-11.
10. Li, X.; Liu, Y.; Hou, X.; Peng, H.; Zhang, L.; Jiang, Q.; Shi, M.; Ji, Y.; Wang, Y.; Shi, W. Chlorogenic acid inhibits the replication and viability of enterovirus 71 in vitro. *PloS one* **2013**, *8*, e76007.
11. Abaidullah, M.; Peng, S.; Song, X.; Zou, Y.; Li, L.; Jia, R.; Yin, Z. Chlorogenic acid is a positive regulator of MDA5, TLR7 and NF-κB signaling pathways mediated antiviral responses against Gammacoronavirus infection. *International immunopharmacology* **2021**, *96*, 107671.
12. Stempien, M.; Nigrelli, R.; Pulitzer, G. Further observations on the Caribbean sponge Cryptotethya crypta (de Laubenfels). *nature* **1965**, *207*, 217-217.
13. Hong, W.-H.; Chang, T.; Daly, R.E. Vidarabine. In *Analytical Profiles of Drug Substances*, Elsevier: 1986; Vol. 15, pp. 647-672.
14. Whitley, R.J.; Alford, C.A.; Hirsch, M.S.; Schooley, R.T.; Luby, J.P.; Aoki, F.Y.; Hanley, D.; Nahmias, A.J.; Soong, S.-J.; Group*, N.C.A.S. Vidarabine versus acyclovir therapy in herpes simplex encephalitis. *New England Journal of Medicine* **1986**, *314*, 144-149.
15. Whitley, R.J.; Nahmias, A.J.; Soong, S.-J.; Galasso, G.G.; Fleming, C.L.; Alford, C.A.; Connor, J.; Bryson, Y.; Linnemann, C. Vidarabine therapy of neonatal herpes simplex virus infection. *Pediatrics* **1980**, *66*, 495-501.
16. Pollard, R.B.; Smith, J.L.; Neal, E.A.; Gregory, P.B.; Merigan, T.C.; Robinson, W.S. Effect of vidarabine on chronic hepatitis B virus infection. *Jama* **1978**, *239*, 1648-1650.
17. Miwa, N.; Kurosaki, K.; Yoshida, Y.; Kurokawa, M.; Saito, S.; Shiraki, K. Comparative efficacy of acyclovir and vidarabine on the replication of varicella-zoster virus. *Antiviral research* **2005**, *65*, 49-55.
18. Chapman, C.; Flower, A.; Durrant, S. The use of vidarabine in the treatment of human polyomavirus associated acute haemorrhagic cystitis. *Bone marrow transplantation* **1991**, *7*, 481-483.

19. Kurosaki, K.; Miwa, N.; Yoshida, Y.; Kurokawa, M.; Kurimoto, M.; Endo, S.; Shiraki, K. Therapeutic basis of vidarabine on adenovirus-induced haemorrhagic cystitis. *Antiviral Chemistry and Chemotherapy* **2004**, *15*, 281-285.
20. Kimura, H.; Morita, M.; Tsuge, I.; Hoshino, Y.; Tanaka, N.; Ito, Y.; Morishima, T. Vidarabine therapy for severe chronic active Epstein–Barr virus infection. *Journal of pediatric hematology/oncology* **2001**, *23*, 294-299.
21. Hutchinson, C.; Colombo, A. Genetic engineering of doxorubicin production in Streptomyces peucetius: a review. *Journal of Industrial Microbiology and Biotechnology* **1999**, *23*, 647-652.
22. Tomita, Y.; Kuwata, T. Effects of adriamycin on the reverse transcriptase and the production of murine leukemia virus. *Cancer research* **1976**, *36*, 3016-3019.
23. Papas, T.S.; Schafer, M.P. The inhibition of Rauscher leukemia virus and avian myeloblastosis virus DNA polymerases by anthracycline compounds. *Annals of the New York Academy of Sciences* **1977**, *284*, 566-575.
24. Cunningham, K.; Manson, W.; Spring, F.; Hutchinson, S. Cordycepin, a metabolic product isolated from cultures of Cordyceps militaris (Linn.) Link. *nature* **1950**, *166*, 949-949.
25. Ohta, Y.; Lee, J.-B.; Hayashi, K.; Fujita, A.; Park, D.K.; Hayashi, T. In vivo anti-influenza virus activity of an immunomodulatory acidic polysaccharide isolated from Cordyceps militaris grown on germinated soybeans. *Journal of Agricultural and Food Chemistry* **2007**, *55*, 10194-10199.
26. Mueller, W.E.; Weiler, B.E.; Charubala, R.; Pfleiderer, W.; Leserman, L.; Sobol, R.W.; Suhadolnik, R.J.; Schroeder, H.C. Cordycepin analogs of 2', 5'-oligoadenylate inhibit human immunodeficiency virus infection via inhibition of reverse transcriptase. *Biochemistry* **1991**, *30*, 2027-2033.
27. Chanda, S.; Banerjee, A.; Nandi, S.; Chakrabarti, S.; Sarkar, M. Cordycepin an adenosine analogue executes anti rotaviral effect by stimulating induction of type I interferon. *J Virol Antivir Res* **2015**, *4*, 2.
28. Ryu, E.; Son, M.; Lee, M.; Lee, K.; Cho, J.Y.; Cho, S.; Lee, S.K.; Lee, Y.M.; Cho, H.; Sung, G.-H. Cordycepin is a novel chemical suppressor of Epstein-Barr virus replication. *Oncoscience* **2014**, *1*, 866.
29. Nishimura, H.; Katagiri, K.; Satō, K.; Mayama, M.; Shimaoka, N. Toyocamycin, a new anti-candida antibiotics. *The Journal of Antibiotics, Series A* **1956**, *9*, 60-62.
30. Moyer, S.A.; Holmes, K.S. The specific inhibition of vesicular stomatitis virus replication by toyocamycin. *Virology* **1979**, *98*, 99-107.
31. Madore, H.P.; Bello, L.J. Stabilization of adenovirus nuclear RNA by intercalating drugs. *Biochimica et Biophysica Acta (BBA)-Nucleic Acids and Protein Synthesis* **1978**, *520*, 404-410.
32. SHIMADA, N.; HASEGAWA, S.; HARADA, T.; TOMISAWA, T.; FUJII, A.; TAKITA, T. Oxetanocin, a novel nucleoside from bacteria. *The Journal of antibiotics* **1986**, *39*, 1623-1625.
33. HOSHINO, H.; SHIMIZU, N.; SHIMADA, N.; TAKITA, T.; TAKEUCHI, T. Inhibition of infectivity of human immunodeficiency virus by oxetanocin. *The Journal of antibiotics* **1987**, *40*, 1077-1078.
34. NAGAHATA, T.; UEDA, K.; TSURIMOTO, T.; CHISAKA, O.; MATSUBARA, K. Anti-hepatitis B virus activities of purine derivatives of oxetanocin A. *The Journal of antibiotics* **1989**, *42*, 644-646.
35. Sakuma, T.; Saijo, M.; Suzutani, T.; Yoshida, I.; Saito, S.; Kitagawa, M.; Hasegawa, S.; Azuma, M. Antiviral activity of oxetanocins against varicella-zoster virus. *Antimicrobial agents and chemotherapy* **1991**, *35*, 1512-1514.
36. OMURA, S.; IMAMURA, N.; KUGA, H.; ISHIKAWA, H.; YAMAZAKI, Y.; OKANO, K.; KIMURA, K.; TAKAHASHI, Y.; TANAKA, H. Adechlorin, a new adenosine deaminase inhibitor containing chlorine production, isolation and properties. *The Journal of antibiotics* **1985**, *38*, 1008-1015.

Method

1- Molecular Similarity

Molecular Similarity of the 310 natural compounds against the co-crystallized ligand (SAM) of SARS-Cov-2 nsp16-nsp10 (**PDB ID: 6W4H**) was carried out calculated using Discovery studio 4.0. At first, the CHARMM force field was applied then the compounds were prepared using prepare ligand protocol. Then, the 310 compounds were used as a test set while the co-crystallized ligand (SAM) was used as a reference compounds. The protocol was adjusted to give 10% output. The default molecular properties were applied. The molecular properties include number of rotatable bonds, number of rings, number of aromatic rings, number of hydrogen bond donors (HBA), number of hydrogen bond acceptors (HBD), partition coefficient (ALog p), molecular weight (M. Wt), and molecular fractional polar surface area (MFPSA).

2- Fingerprint study

Fingerprint study of the 30 selected compounds against the co-crystallized ligand (SAM) of SARS-Cov-2 nsp16-nsp10 (**PDB ID: 6W4H**) was carried out calculated using Discovery studio 4.0. At first, the CHARMM force field was applied then the compounds were prepared using prepare ligand protocol. Then, the 30 compounds were used as a test set while the co-crystallized ligand (SAM) was used as a reference compounds. The protocol was adjusted to give the most related compounds to the co-crystallized ligand (40-50%). The default molecular properties were applied. The used fingerprints were based on some parameters related to type of atoms which may be one of the following: charge, hybridization, H-bond acceptor, H-bond donor, Positive ionizable, Negative ionizable, Halogen, Aromatic, or None of the above. In addition, it includes the ALogP category of atoms.

3- Docking studies

Crystal structure of SARS-Cov-2 nsp16-nsp10 (**PDB ID: 6W4H**), resolution: 1.80 Å] was obtained from Protein Data Bank. The docking investigation was accomplished using MOE2014 software. At first, the crystal structure of SARS-Cov-2 nsp16-nsp10 was prepared by removing water molecules. Only one chain was retained beside the co-crystallized ligand (SAM). Then, the selected chain was protonated and subjected to minimization of energy process. Next, the active site of the target protein was defined.

Structures of the tested compounds and the co-crystallized ligand were drawn using ChemBioDraw Ultra 14.0 and saved as MDL-SD format. Such file was opened using MOE to display the 3D structures which were protonated and subjected to energy minimization. Formerly, validation of the docking process was performed by docking the co-crystallized ligand against the isolated pocket of active site. The produced RMSD value indicated the validity of process. Finally, docking of the tested compounds was done through the dock option inserted in compute window. For each docked molecule, 30 docked poses were

produced using ASE for scoring function and force field for refinement. The results of the docking process were then visualized using Discovery Studio 4.0 software.

4- ADMET studies

ADMET descriptors (absorption, distribution, metabolism, excretion and toxicity) of the compounds were determined using Discovery studio 4.0. At first, the CHARMM force field was applied then the tested compounds were prepared and minimized according to the preparation of small molecule protocol. Then ADMET descriptors protocol was applied to carry out these studies.

5- Toxicity studies

The toxicity parameters of the tested compounds were calculated using Discovery studio 4.0. Ribavirin was used as a reference drug. At first, the CHARMM force field was applied then the compounds were prepared and minimized according to the preparation of small molecule protocol. Then different parameters were calculated from toxicity prediction (extensible) protocol.

6- DFT studies:

The DFT parameters (total energy, binding energy, HOMO, LUMO, gap energy, dipole moment, and electrostatic potential) were calculated using Discovery studio software. the tested compounds were prepared using prepare ligand protocol. Then, the prepared compounds were subjected to DFT calculation protocol using the default option.

Toxicity report -48

TOPKAT_Aerobic_Biodegradability

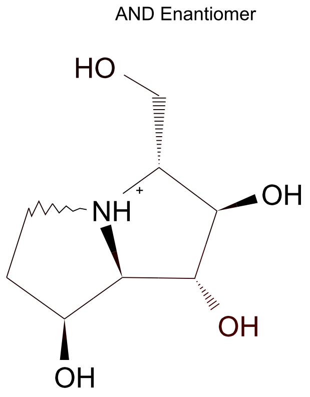

$C_8H_{16}NO_4$

Molecular Weight: 190.21693

ALogP: -3.556

Rotatable Bonds: 1

Acceptors: 4

Donors: 5

Structural Similar Compounds

Name	1,3-Propanediol,_2,2-bis(hydroxymethyl)-	1,2,3-Propanetriol	1,3,5-Pentanetriol,_3-methyl-
Structure	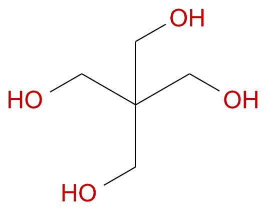	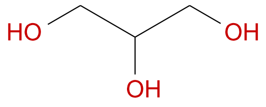	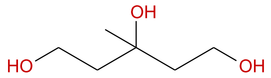
Actual Endpoint	Non-Degradable	Degradable	Non-Degradable
Predicted Endpoint	Degradable	Degradable	Degradable
Distance	0.726	0.972	0.987
Reference	Environmental Toxicology & Chemistry 18(9), 1763-1768, 1999.	Environmental Toxicology & Chemistry 18(9), 1763-1768, 1999.	Environmental Toxicology & Chemistry 18(9), 1763-1768, 1999.

Model Prediction

Prediction: Degradable

Probability: 0.551

Enrichment: 1.262

Bayesian Score: 1.265

Mahalanobis Distance: 12.042

Mahalanobis Distance p-value: 0.000264

Prediction: Positive if the Bayesian score is above the estimated best cutoff value from minimizing the false positive and false negative rate.
Probability: The esimated probability that the sample is in the positive category. This assumes that the Bayesian score follows a normal distribution and is different from the prediction using a cutoff.
Enrichment: An estimate of enrichment, that is, the increased likelihood (versus random) of this sample being in the category.
Bayesian Score: The standard Laplacian-modified Bayesian score.
Mahalanobis Distance: The Mahalanobis distance (MD) is the distance to the center of the training data. The larger the MD, the less trustworthy the prediction.
Mahalanobis Distance p-value: The p-value gives the fraction of training data with an MD greater than or equal to the one for the given sample, assuming normally distributed data. The smaller the p-value, the less trustworthy the prediciton. For highly non-normal X properties (e.g., fingerprints), the MD p-value is wildly inaccurate.

Model Applicability

Unknown features are fingerprint features in the query molecule, but not found in the training set.

1. All properties and OPS components are within expected ranges.

Feature Contribution

Top features for positive contribution

Fingerprint	Bit/Smiles	Feature Structure	Score	Degradable in training set
SCFP_12	-424515134	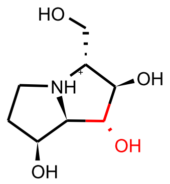 [*]C([*])O	0.648	87 out of 111

SCFP_12	-1272798659	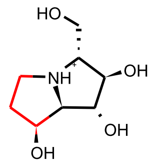 [*][C@@H]1[*][*]CC1	0.518	160 out of 234
SCFP_12	-711686199	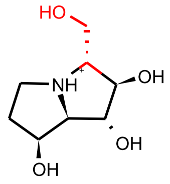 [*]C([*])CO	0.490	106 out of 159

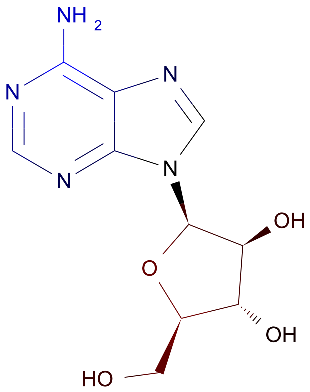

$C_{10}H_{13}N_5O_4$
Molecular Weight: 267.24132
ALogP: -1.881
Rotatable Bonds: 2
Acceptors: 8
Donors: 4

Structural Similar Compounds

Name	H_Acid	Benzenesulfonic_acid,_4,4'-oxybis-,_dihydrazide	Glycine,_N,N-bis(carboxymethyl)-
Structure	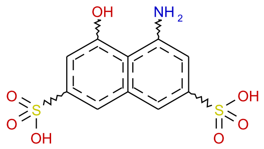	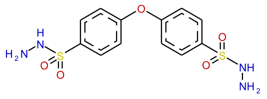	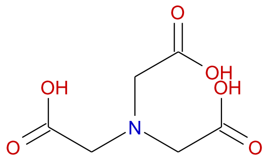
Actual Endpoint	Non-Degradable	Non-Degradable	Non-Degradable
Predicted Endpoint	Non-Degradable	Non-Degradable	Non-Degradable
Distance	0.700	0.751	0.852
Reference	Environmental Toxicology & Chemistry 18(9), 1763-1768, 1999.	Environmental Toxicology & Chemistry 18(9), 1763-1768, 1999.	Environmental Toxicology & Chemistry 18(9), 1763-1768, 1999.

Model Prediction

Prediction: Non-Degradable
Probability: 0.241
Enrichment: 0.553
Bayesian Score: -5.853
Mahalanobis Distance: 16.343
Mahalanobis Distance p-value: 1.39e-016

Prediction: Positive if the Bayesian score is above the estimated best cutoff value from minimizing the false positive and false negative rate.
Probability: The esimated probability that the sample is in the positive category. This assumes that the Bayesian score follows a normal distribution and is different from the prediction using a cutoff.
Enrichment: An estimate of enrichment, that is, the increased likelihood (versus random) of this sample being in the category.
Bayesian Score: The standard Laplacian-modified Bayesian score.
Mahalanobis Distance: The Mahalanobis distance (MD) is the distance to the center of the training data. The larger the MD, the less trustworthy the prediction.
Mahalanobis Distance p-value: The p-value gives the fraction of training data with an MD greater than or equal to the one for the given sample, assuming normally distributed data. The smaller the p-value, the less trustworthy the prediciton. For highly non-normal X properties (e.g., fingerprints), the MD p-value is wildly inaccurate.

Model Applicability

Unknown features are fingerprint features in the query molecule, but not found in the training set.

1. OPS PC13 out of range. Value: -4.9079. Training min, max, SD, explained variance: -3.586, 5.704, 1.413, 0.0229.

Feature Contribution

Top features for positive contribution

Fingerprint	Bit/Smiles	Feature Structure	Score	Degradable in training set
SCFP_12	-424515134	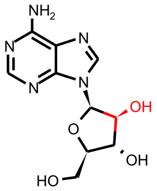 [*]C([*])O	0.648	87 out of 111

SCFP_12	-188384666	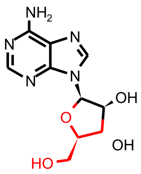 [*][C@H]1[*][*]O[C@@H]1CO	0.590	3 out of 3
SCFP_12	-711686199	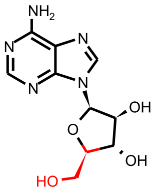 [*]C([*])CO	0.490	106 out of 159

Top Features for negative contribution

Fingerprint	Bit/Smiles	Feature Structure	Score	Degradable in training set
SCFP_12	10	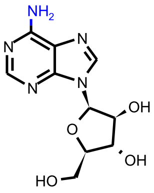 [*]N	-1.607	11 out of 145
SCFP_12	384920865	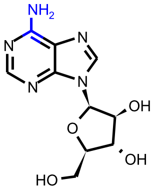 [*]:[c](:[*])N	-1.364	6 out of 65
SCFP_12	112554633	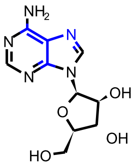 [*][c](:[*]):[c]1:n:[*]:[*]:[c]:1:[*]	-1.077	1 out of 12

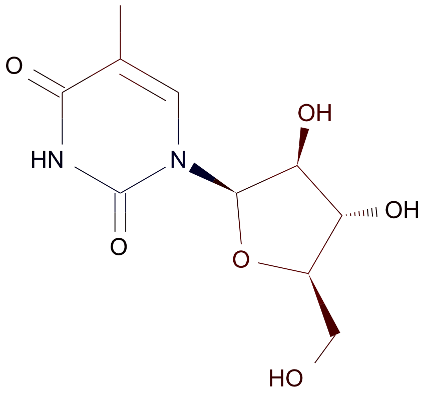

$C_{10}H_{14}N_2O_6$
Molecular Weight: 258.22795
ALogP: -2.005
Rotatable Bonds: 2
Acceptors: 6
Donors: 4

Structural Similar Compounds

Name	1,3,5-Triazine-2,4,6(1H,3H,5H)-trione,_1,3,5-tris(2-hydroxyethyl)-	Benzenesulfonic_acid,_4,4'-oxybis-,_dihydrazide	1,3-Propanediol,_2,2-bis(hydroxymethyl)-
Structure	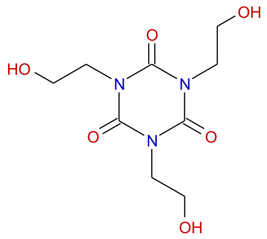	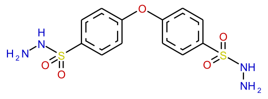	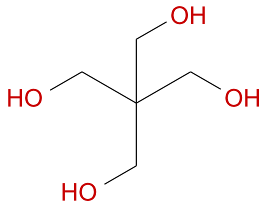
Actual Endpoint	Non-Degradable	Non-Degradable	Non-Degradable
Predicted Endpoint	Non-Degradable	Non-Degradable	Degradable
Distance	0.690	0.766	0.784
Reference	Environmental Toxicology & Chemistry 18(9), 1763-1768, 1999.	Environmental Toxicology & Chemistry 18(9), 1763-1768, 1999.	Environmental Toxicology & Chemistry 18(9), 1763-1768, 1999.

Model Prediction

Prediction: Degradable
Probability: 0.626
Enrichment: 1.435
Bayesian Score: 2.880
Mahalanobis Distance: 11.416
Mahalanobis Distance p-value: 0.00333

Prediction: Positive if the Bayesian score is above the estimated best cutoff value from minimizing the false positive and false negative rate.
Probability: The esimated probability that the sample is in the positive category. This assumes that the Bayesian score follows a normal distribution and is different from the prediction using a cutoff.
Enrichment: An estimate of enrichment, that is, the increased likelihood (versus random) of this sample being in the category.
Bayesian Score: The standard Laplacian-modified Bayesian score.
Mahalanobis Distance: The Mahalanobis distance (MD) is the distance to the center of the training data. The larger the MD, the less trustworthy the prediction.
Mahalanobis Distance p-value: The p-value gives the fraction of training data with an MD greater than or equal to the one for the given sample, assuming normally distributed data. The smaller the p-value, the less trustworthy the prediciton. For highly non-normal X properties (e.g., fingerprints), the MD p-value is wildly inaccurate.

Model Applicability

Unknown features are fingerprint features in the query molecule, but not found in the training set.

1. All properties and OPS components are within expected ranges.

Feature Contribution

Top features for positive contribution				
**Fingerprint**	**Bit/Smiles**	**Feature Structure**	**Score**	**Degradable in training set**
SCFP_12	136627117	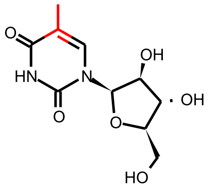 [*]C(=[*])C	0.662	56 out of 70

SCFP_12	-424515134	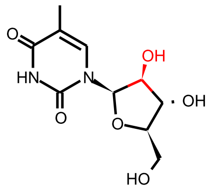<br>[*]C([*])O	0.648	87 out of 111
SCFP_12	-188384666	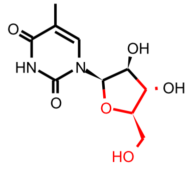<br>[*][C@H]1[*][*]O[C@@H]1CO	0.590	3 out of 3

Top Features for negative contribution

Fingerprint	Bit/Smiles	Feature Structure	Score	Degradable in training set
SCFP_12	1256786467	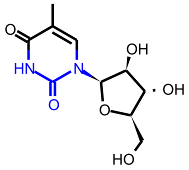<br>[*]NC(=O)N([*])[*]	-0.541	1 out of 6
SCFP_12	399659969	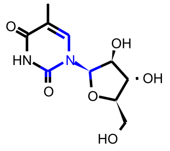<br>[*]C([*])N(C=[*])C(=[*])[*]	-0.341	0 out of 1
SCFP_12	1631785938	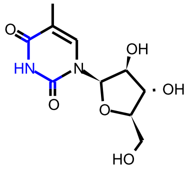<br>[*]C(=[*])NC(=[*])[*]	-0.271	1 out of 4

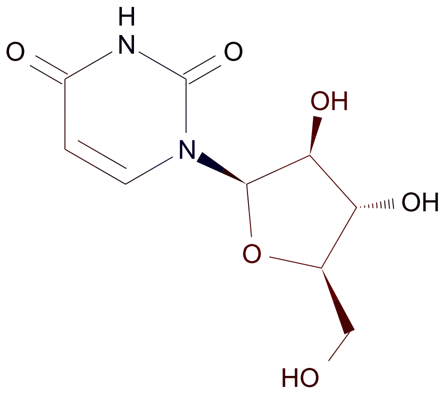

$C_9H_{12}N_2O_6$
Molecular Weight: 244.20137
ALogP: -2.451
Rotatable Bonds: 2
Acceptors: 6
Donors: 4

Structural Similar Compounds

Name	1,3,5-Triazine-2,4,6(1H,3H,5H)-trione,_1,3,5-tris(2-hydroxyethyl)-	1,3-Propanediol,_2,2-bis(hydroxymethyl)-	Benzenesulfonic_acid,_4,4'-oxybis-,_dihydrazide
Structure	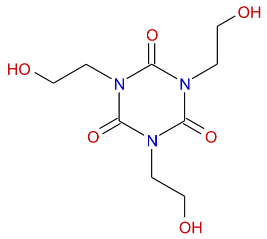	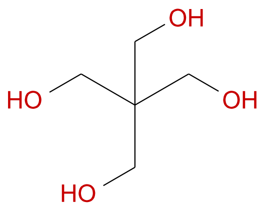	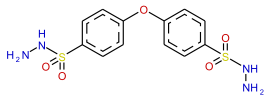
Actual Endpoint	Non-Degradable	Non-Degradable	Non-Degradable
Predicted Endpoint	Non-Degradable	Degradable	Non-Degradable
Distance	0.708	0.772	0.787
Reference	Environmental Toxicology & Chemistry 18(9), 1763-1768, 1999.	Environmental Toxicology & Chemistry 18(9), 1763-1768, 1999.	Environmental Toxicology & Chemistry 18(9), 1763-1768, 1999.

Model Prediction

Prediction: Degradable
Probability: 0.598
Enrichment: 1.370
Bayesian Score: 2.263
Mahalanobis Distance: 10.792
Mahalanobis Distance p-value: 0.0261

Prediction: Positive if the Bayesian score is above the estimated best cutoff value from minimizing the false positive and false negative rate.
Probability: The esimated probability that the sample is in the positive category. This assumes that the Bayesian score follows a normal distribution and is different from the prediction using a cutoff.
Enrichment: An estimate of enrichment, that is, the increased likelihood (versus random) of this sample being in the category.
Bayesian Score: The standard Laplacian-modified Bayesian score.
Mahalanobis Distance: The Mahalanobis distance (MD) is the distance to the center of the training data. The larger the MD, the less trustworthy the prediction.
Mahalanobis Distance p-value: The p-value gives the fraction of training data with an MD greater than or equal to the one for the given sample, assuming normally distributed data. The smaller the p-value, the less trustworthy the prediciton. For highly non-normal X properties (e.g., fingerprints), the MD p-value is wildly inaccurate.

Model Applicability

Unknown features are fingerprint features in the query molecule, but not found in the training set.

1. All properties and OPS components are within expected ranges.

Feature Contribution

Top features for positive contribution				
**Fingerprint**	**Bit/Smiles**	**Feature Structure**	**Score**	**Degradable in training set**
SCFP_12	-424515134	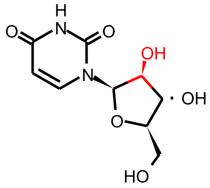 [*]C([*])O	0.648	87 out of 111

SCFP_12	-1971196727	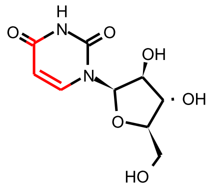 [*]\C=C/C(=[*])[*]	0.621	22 out of 28
SCFP_12	-188384666	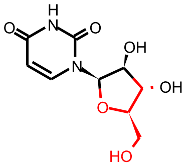 [*][C@H]1[*][*]O[C@@H]1CO	0.590	3 out of 3

Top Features for negative contribution

Fingerprint	Bit/Smiles	Feature Structure	Score	Degradable in training set
SCFP_12	1256786467	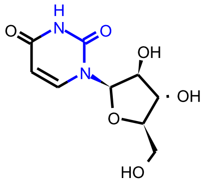 [*]NC(=O)N([*])[*]	-0.541	1 out of 6
SCFP_12	399659969	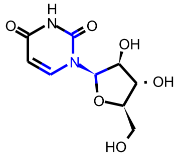 [*]C([*])N(C=[*])C(=[*])[*]	-0.341	0 out of 1
SCFP_12	1631785938	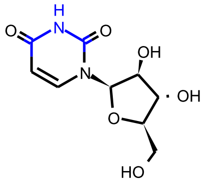 [*]C(=[*])NC(=[*])[*]	-0.271	1 out of 4

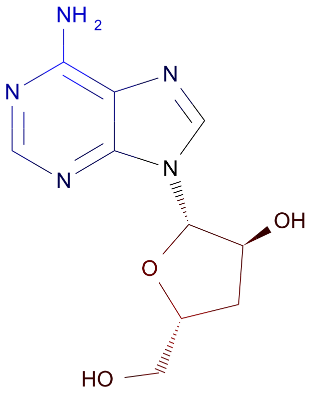

$C_{10}H_{13}N_5O_3$
Molecular Weight: 251.24191
ALogP: -1.308
Rotatable Bonds: 2
Acceptors: 7
Donors: 3

Model Prediction

Prediction: Non-Degradable
Probability: 0.250
Enrichment: 0.572
Bayesian Score: -5.613
Mahalanobis Distance: 16.522
Mahalanobis Distance p-value: 3.06e-017

Prediction: Positive if the Bayesian score is above the estimated best cutoff value from minimizing the false positive and false negative rate.
Probability: The esimated probability that the sample is in the positive category. This assumes that the Bayesian score follows a normal distribution and is different from the prediction using a cutoff.
Enrichment: An estimate of enrichment, that is, the increased likelihood (versus random) of this sample being in the category.
Bayesian Score: The standard Laplacian-modified Bayesian score.
Mahalanobis Distance: The Mahalanobis distance (MD) is the distance to the center of the training data. The larger the MD, the less trustworthy the prediction.
Mahalanobis Distance p-value: The p-value gives the fraction of training data with an MD greater than or equal to the one for the given sample, assuming normally distributed data. The smaller the p-value, the less trustworthy the prediciton. For highly non-normal X properties (e.g., fingerprints), the MD p-value is wildly inaccurate.

Structural Similar Compounds

Name	1,3,5-Triazine-2,4,6(1H,3H,5H)-trione,_1,3,5-tris(2-hydroxyethyl)-	Glycine,_N,N-bis(carboxymethyl)-	2-Naphthalenesulfonic_acid,_6-amino-4-hydroxy-
Structure	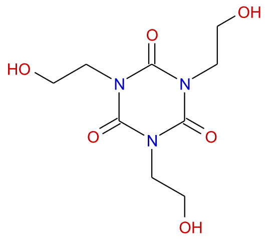	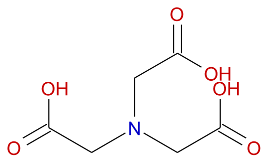	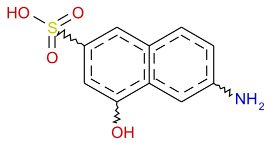
Actual Endpoint	Non-Degradable	Non-Degradable	Non-Degradable
Predicted Endpoint	Non-Degradable	Non-Degradable	Non-Degradable
Distance	0.656	0.769	0.773
Reference	Environmental Toxicology & Chemistry 18(9), 1763-1768, 1999.	Environmental Toxicology & Chemistry 18(9), 1763-1768, 1999.	Environmental Toxicology & Chemistry 18(9), 1763-1768, 1999.

Model Applicability

Unknown features are fingerprint features in the query molecule, but not found in the training set.

1. OPS PC13 out of range. Value: -4.9259. Training min, max, SD, explained variance: -3.586, 5.704, 1.413, 0.0229.

Feature Contribution

Top features for positive contribution

Fingerprint	Bit/Smiles	Feature Structure	Score	Degradable in training set
SCFP_12	-424515134	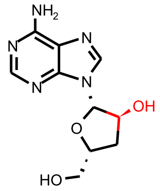 [*]C([*])O	0.648	87 out of 111

SCFP_12	-188384666	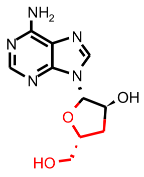<br>[*][C@H]1[*][*]O[C@@H<br>]1CO	0.590	3 out of 3
SCFP_12	-1272798659	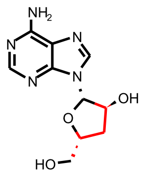<br>[*][C@@H]1[*][*]CC1	0.518	160 out of 234
**Top Features for negative contribution**				
**Fingerprint**	**Bit/Smiles**	**Feature Structure**	**Score**	**Degradable in training set**
SCFP_12	10	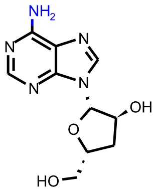<br>[*]N	-1.607	11 out of 145
SCFP_12	384920865	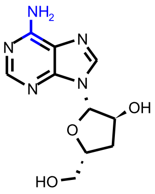<br>[*]:[c](:[*])N	-1.364	6 out of 65
SCFP_12	112554633	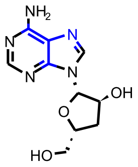<br>[*][c](:[*]):[c]1:n:[<br>*]:[*]:[c]:1:[*]	-1.077	1 out of 12

Ribavirin

TOPKAT_Aerobic_Biodegradability

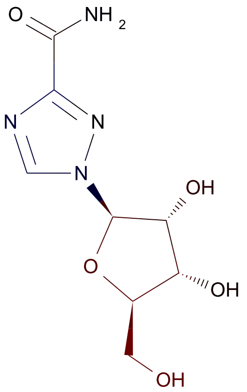

$C_8H_{12}N_4O_5$

Molecular Weight: 244.20467

ALogP: -2.745

Rotatable Bonds: 3

Acceptors: 7

Donors: 4

Model Prediction

Prediction: Degradable

Probability: 0.511

Enrichment: 1.171

Bayesian Score: 0.442

Mahalanobis Distance: 14.890

Mahalanobis Distance p-value: 1.26e-011

Prediction: Positive if the Bayesian score is above the estimated best cutoff value from minimizing the false positive and false negative rate.
Probability: The esimated probability that the sample is in the positive category. This assumes that the Bayesian score follows a normal distribution and is different from the prediction using a cutoff.
Enrichment: An estimate of enrichment, that is, the increased likelihood (versus random) of this sample being in the category.
Bayesian Score: The standard Laplacian-modified Bayesian score.
Mahalanobis Distance: The Mahalanobis distance (MD) is the distance to the center of the training data. The larger the MD, the less trustworthy the prediction.
Mahalanobis Distance p-value: The p-value gives the fraction of training data with an MD greater than or equal to the one for the given sample, assuming normally distributed data. The smaller the p-value, the less trustworthy the prediciton. For highly non-normal X properties (e.g., fingerprints), the MD p-value is wildly inaccurate.

Structural Similar Compounds

Name	Glycine,_N,N-bis(carboxymethyl)-	H_Acid	Benzenesulfonic_acid,_4,4'-oxybis-,_dihydrazide
Structure	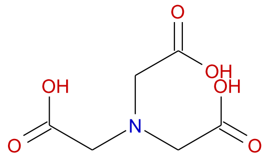	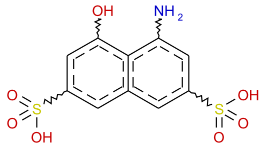	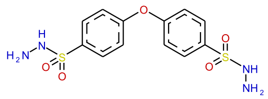
Actual Endpoint	Non-Degradable	Non-Degradable	Non-Degradable
Predicted Endpoint	Non-Degradable	Non-Degradable	Non-Degradable
Distance	0.752	0.768	0.770
Reference	Environmental Toxicology & Chemistry 18(9), 1763-1768, 1999.	Environmental Toxicology & Chemistry 18(9), 1763-1768, 1999.	Environmental Toxicology & Chemistry 18(9), 1763-1768, 1999.

Model Applicability

Unknown features are fingerprint features in the query molecule, but not found in the training set.

1. OPS PC13 out of range. Value: -4.125. Training min, max, SD, explained variance: -3.586, 5.704, 1.413, 0.0229.

Feature Contribution

Top features for positive contribution				
**Fingerprint**	**Bit/Smiles**	**Feature Structure**	**Score**	**Degradable in training set**
SCFP_12	-424515134	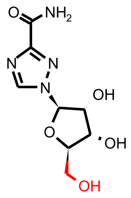 [*]C([*])O	0.648	87 out of 111

SCFP_12	-188384666	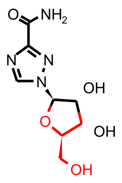 [*][C@H]1[*][*]O[C@@H]1CO	0.590	3 out of 3
SCFP_12	-711686199	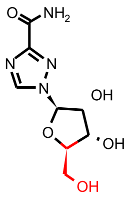 [*]C([*])CO	0.490	106 out of 159
**Top Features for negative contribution**				
**Fingerprint**	**Bit/Smiles**	**Feature Structure**	**Score**	**Degradable in training set**
SCFP_12	-1181430618	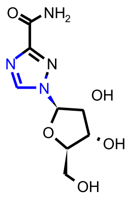 [*]:n:[cH]:n:[*]	-0.594	0 out of 2
SCFP_12	1257084377	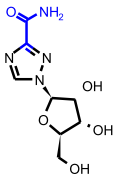 [*]:[c](:[*])C(=O)N	-0.541	1 out of 6
SCFP_12	149003983	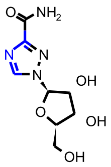 [*][c](:[*]):n:[cH]:[*]	-0.497	8 out of 34

AND Enantiomer

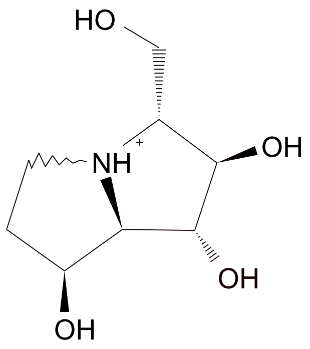

$C_8H_{16}NO_4$

Molecular Weight: 190.21693

ALogP: -3.556

Rotatable Bonds: 1

Acceptors: 4

Donors: 5

Model Prediction

Prediction: Toxic

Probability: 0.584

Enrichment: 1.110

Bayesian Score: 0.944

Mahalanobis Distance: 7.814

Mahalanobis Distance p-value: 0.693

Prediction: Positive if the Bayesian score is above the estimated best cutoff value from minimizing the false positive and false negative rate.
Probability: The esimated probability that the sample is in the positive category. This assumes that the Bayesian score follows a normal distribution and is different from the prediction using a cutoff.
Enrichment: An estimate of enrichment, that is, the increased likelihood (versus random) of this sample being in the category.
Bayesian Score: The standard Laplacian-modified Bayesian score.
Mahalanobis Distance: The Mahalanobis distance (MD) is the distance to the center of the training data. The larger the MD, the less trustworthy the prediction.
Mahalanobis Distance p-value: The p-value gives the fraction of training data with an MD greater than or equal to the one for the given sample, assuming normally distributed data. The smaller the p-value, the less trustworthy the prediciton. For highly non-normal X properties (e.g., fingerprints), the MD p-value is wildly inaccurate.

Structural Similar Compounds

Name	Bredinin	Ribavirin	Vidarabine
Structure	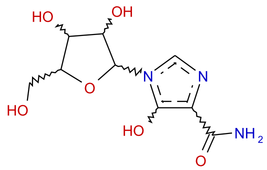	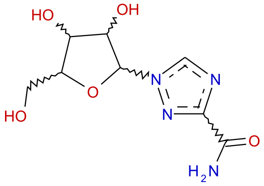	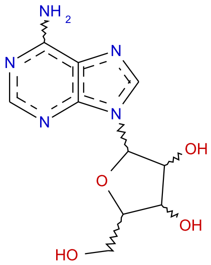
Actual Endpoint	Toxic	Toxic	Non-Toxic
Predicted Endpoint	Toxic	Toxic	Non-Toxic
Distance	0.784	0.798	0.827
Reference	Oyo Yakuri 26(3):377-381; 1983	Teratology 17(1):93-101; 1978	Teratology 15(3):231-41; 1977

Model Applicability

Unknown features are fingerprint features in the query molecule, but not found in the training set.

1. All properties and OPS components are within expected ranges.

Feature Contribution

Top features for positive contribution				
**Fingerprint**	**Bit/Smiles**	**Feature Structure**	**Score**	**Toxic in training set**
SCFP_6	-424515134	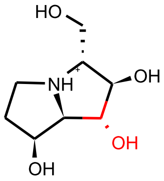 [*]C([*])O	0.275	39 out of 56

SCFP_6	1702634808	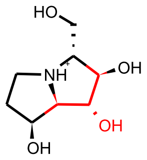 [*][C@@H]1[*][*][C@H]([*])[C@H]1O	0.159	26 out of 42
SCFP_6	-1272798659	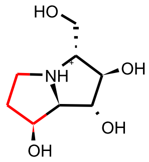 [*][C@@H]1[*][*]CC1	0.071	44 out of 78
**Top Features for negative contribution**				
**Fingerprint**	**Bit/Smiles**	**Feature Structure**	**Score**	**Toxic in training set**
SCFP_6	12	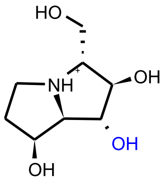 [*]O	0.000	97 out of 178
SCFP_6	0	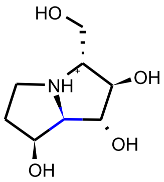 [*]C([*])[*]	0.000	122 out of 230

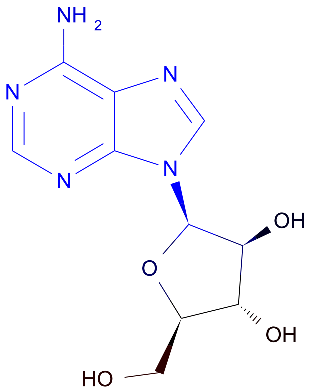

$C_{10}H_{13}N_5O_4$
Molecular Weight: 267.24132
ALogP: -1.881
Rotatable Bonds: 2
Acceptors: 8
Donors: 4

Model Prediction

Prediction: Non-Toxic
Probability: 0.281
Enrichment: 0.534
Bayesian Score: -9.108
Mahalanobis Distance: 7.347
Mahalanobis Distance p-value: 0.866

Prediction: Positive if the Bayesian score is above the estimated best cutoff value from minimizing the false positive and false negative rate.
Probability: The esimated probability that the sample is in the positive category. This assumes that the Bayesian score follows a normal distribution and is different from the prediction using a cutoff.
Enrichment: An estimate of enrichment, that is, the increased likelihood (versus random) of this sample being in the category.
Bayesian Score: The standard Laplacian-modified Bayesian score.
Mahalanobis Distance: The Mahalanobis distance (MD) is the distance to the center of the training data. The larger the MD, the less trustworthy the prediction.
Mahalanobis Distance p-value: The p-value gives the fraction of training data with an MD greater than or equal to the one for the given sample, assuming normally distributed data. The smaller the p-value, the less trustworthy the prediciton. For highly non-normal X properties (e.g., fingerprints), the MD p-value is wildly inaccurate.

Structural Similar Compounds

Name	Vidarabine	Bredinin	Ribavirin
Structure	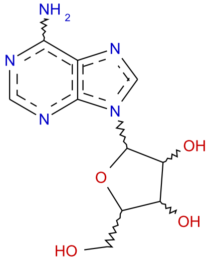	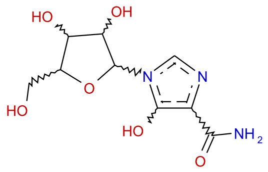	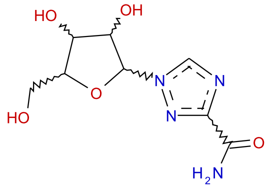
Actual Endpoint	Non-Toxic	Toxic	Toxic
Predicted Endpoint	Non-Toxic	Toxic	Toxic
Distance	0.000	0.513	0.527
Reference	Teratology 15(3):231-41; 1977	Oyo Yakuri 26(3):377-381; 1983	Teratology 17(1):93-101; 1978

Model Applicability

Unknown features are fingerprint features in the query molecule, but not found in the training set.

1. All properties and OPS components are within expected ranges.

Feature Contribution

Top features for positive contribution				
**Fingerprint**	**Bit/Smiles**	**Feature Structure**	**Score**	**Toxic in training set**
SCFP_6	-1486266146	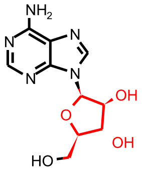 [*]C[C@H]1O[C@@H]([*])[C@@H](O)[C@@H]1O	0.431	7 out of 8

SCFP_6	-188384666	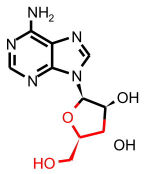 [*][C@H]1[*][*]O[C@@H]1CO	0.431	7 out of 8
SCFP_6	-1181430618	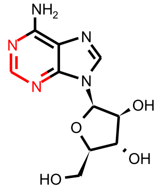 [*]:n:[cH]:n:[*]	0.298	6 out of 8
**Top Features for negative contribution**				
**Fingerprint**	**Bit/Smiles**	**Feature Structure**	**Score**	**Toxic in training set**
SCFP_6	-572674910	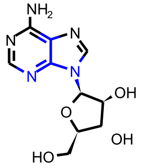 [*]n1:[*]:[*]:[c](:[*]):[c]:1:n:[*]	-0.945	0 out of 3
SCFP_6	41215856	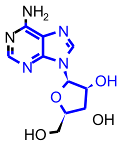 [*][C@H]1O[C@H]([C@@H](O)[C@@H]1[*])n2:[cH]:n:[c]3:[c]([*]):[*]:[cH]:n:[c]:2:3	-0.718	0 out of 2
SCFP_6	1607737603	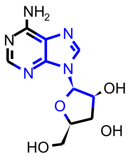 [*][C@H]1[*][*]O[C@H]1n2:[cH]:n:[c](:[*]):[c]:2:n:[*]	-0.718	0 out of 2

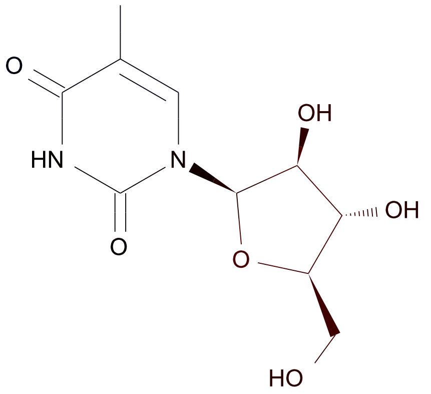

$C_{10}H_{14}N_2O_6$
Molecular Weight: 258.22795
ALogP: -2.005
Rotatable Bonds: 2
Acceptors: 6
Donors: 4

Model Prediction

Prediction: Toxic
Probability: 0.610
Enrichment: 1.160
Bayesian Score: 1.621
Mahalanobis Distance: 7.635
Mahalanobis Distance p-value: 0.768

Prediction: Positive if the Bayesian score is above the estimated best cutoff value from minimizing the false positive and false negative rate.
Probability: The esimated probability that the sample is in the positive category. This assumes that the Bayesian score follows a normal distribution and is different from the prediction using a cutoff.
Enrichment: An estimate of enrichment, that is, the increased likelihood (versus random) of this sample being in the category.
Bayesian Score: The standard Laplacian-modified Bayesian score.
Mahalanobis Distance: The Mahalanobis distance (MD) is the distance to the center of the training data. The larger the MD, the less trustworthy the prediction.
Mahalanobis Distance p-value: The p-value gives the fraction of training data with an MD greater than or equal to the one for the given sample, assuming normally distributed data. The smaller the p-value, the less trustworthy the prediciton. For highly non-normal X properties (e.g., fingerprints), the MD p-value is wildly inaccurate.

Structural Similar Compounds

Name	Vidarabine	Ribavirin	Bredinin
Structure	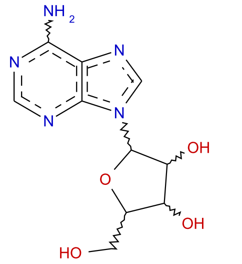	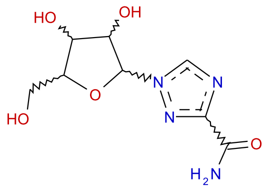	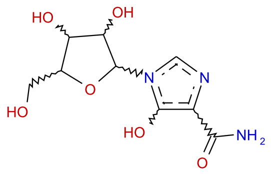
Actual Endpoint	Non-Toxic	Toxic	Toxic
Predicted Endpoint	Non-Toxic	Toxic	Toxic
Distance	0.596	0.607	0.634
Reference	Teratology 15(3):231-41; 1977	Teratology 17(1):93-101; 1978	Oyo Yakuri 26(3):377-381; 1983

Model Applicability

Unknown features are fingerprint features in the query molecule, but not found in the training set.

1. All properties and OPS components are within expected ranges.

Feature Contribution

Top features for positive contribution

Fingerprint	Bit/Smiles	Feature Structure	Score	Toxic in training set
SCFP_6	-188384666	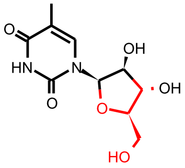 [*][C@H]1[*][*]O[C@@H]1CO	0.431	7 out of 8

SCFP_6	-1486266146	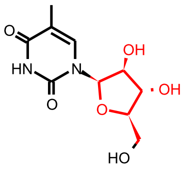 [*]C[C@H]1O[C@@H]([*]<br>)[C@@H](O)[C@@H]1O	0.431	7 out of 8
SCFP_6	-1971435055	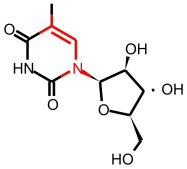 [*]N([*])C=C([*])[*]	0.381	2 out of 2

Top Features for negative contribution

Fingerprint	Bit/Smiles	Feature Structure	Score	Toxic in training set
SCFP_6	399659969	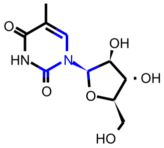 [*]C([*])N(C=[*])C(=[<br>*])[*]	-0.526	3 out of 11
SCFP_6	1257024795	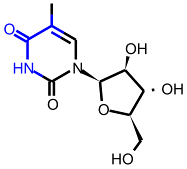 [*]NC(=O)C(=[*])[*]	-0.303	4 out of 11
SCFP_6	55464376	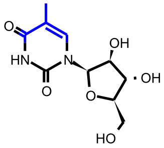 [*]\C=C(\C)/C(=[*])[*<br>]	-0.252	1 out of 3

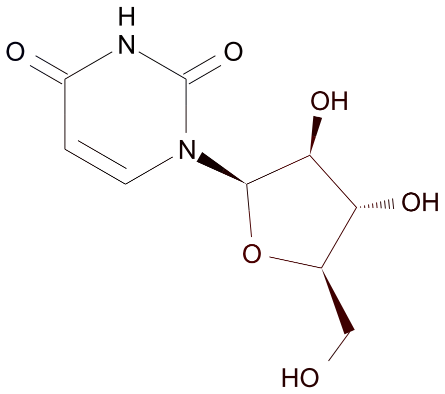

$C_9H_{12}N_2O_6$
Molecular Weight: 244.20137
ALogP: -2.451
Rotatable Bonds: 2
Acceptors: 6
Donors: 4

Model Prediction

Prediction: Toxic
Probability: 0.641
Enrichment: 1.219
Bayesian Score: 2.411
Mahalanobis Distance: 7.919
Mahalanobis Distance p-value: 0.645

Prediction: Positive if the Bayesian score is above the estimated best cutoff value from minimizing the false positive and false negative rate.
Probability: The esimated probability that the sample is in the positive category. This assumes that the Bayesian score follows a normal distribution and is different from the prediction using a cutoff.
Enrichment: An estimate of enrichment, that is, the increased likelihood (versus random) of this sample being in the category.
Bayesian Score: The standard Laplacian-modified Bayesian score.
Mahalanobis Distance: The Mahalanobis distance (MD) is the distance to the center of the training data. The larger the MD, the less trustworthy the prediction.
Mahalanobis Distance p-value: The p-value gives the fraction of training data with an MD greater than or equal to the one for the given sample, assuming normally distributed data. The smaller the p-value, the less trustworthy the prediciton. For highly non-normal X properties (e.g., fingerprints), the MD p-value is wildly inaccurate.

Structural Similar Compounds

Name	Ribavirin	Vidarabine	Bredinin
Structure	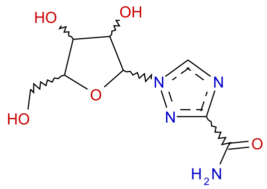	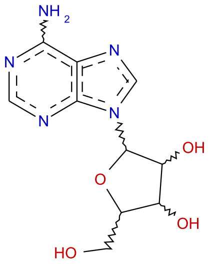	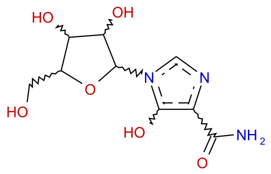
Actual Endpoint	Toxic	Non-Toxic	Toxic
Predicted Endpoint	Toxic	Non-Toxic	Toxic
Distance	0.543	0.586	0.590
Reference	Teratology 17(1):93-101; 1978	Teratology 15(3):231-41; 1977	Oyo Yakuri 26(3):377-381; 1983

Model Applicability

Unknown features are fingerprint features in the query molecule, but not found in the training set.

1. All properties and OPS components are within expected ranges.

Feature Contribution

Top features for positive contribution				
**Fingerprint**	**Bit/Smiles**	**Feature Structure**	**Score**	**Toxic in training set**
SCFP_6	-188384666	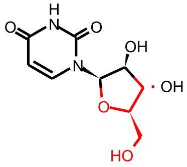 [*][C@H]1[*][*]O[C@@H]1CO	0.431	7 out of 8

SCFP_6	-1486266146	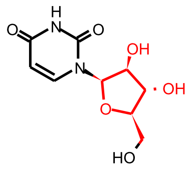<br>[*]C[C@H]1O[C@@H]([*]<br>)[C@@H](O)[C@@H]1O	0.431	7 out of 8
SCFP_6	-1971435055	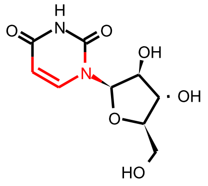<br>[*]N([*])C=C([*])[*]	0.381	2 out of 2
**Top Features for negative contribution**				
**Fingerprint**	**Bit/Smiles**	**Feature Structure**	**Score**	**Toxic in training set**
SCFP_6	399659969	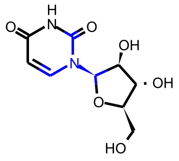<br>[*]C([*])N(C=[*])C(=[<br>*])[*]	-0.526	3 out of 11
SCFP_6	1257024795	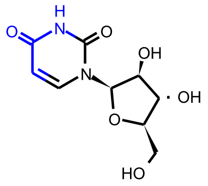<br>[*]NC(=O)C(=[*])[*]	-0.303	4 out of 11
SCFP_6	0	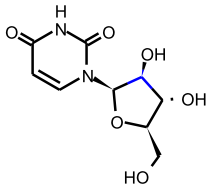<br>[*]C([*])[*]	0.000	122 out of 230

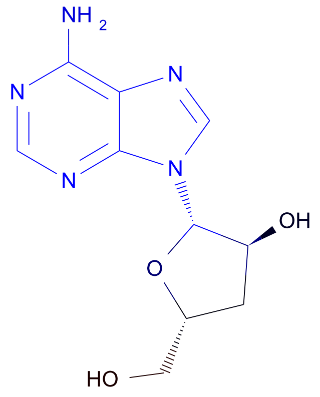

$C_{10}H_{13}N_5O_3$
Molecular Weight: 251.24191
ALogP: -1.308
Rotatable Bonds: 2
Acceptors: 7
Donors: 3

Model Prediction

Prediction: Non-Toxic
Probability: 0.283
Enrichment: 0.538
Bayesian Score: -9.029
Mahalanobis Distance: 7.929
Mahalanobis Distance p-value: 0.64

Prediction: Positive if the Bayesian score is above the estimated best cutoff value from minimizing the false positive and false negative rate.
Probability: The esimated probability that the sample is in the positive category. This assumes that the Bayesian score follows a normal distribution and is different from the prediction using a cutoff.
Enrichment: An estimate of enrichment, that is, the increased likelihood (versus random) of this sample being in the category.
Bayesian Score: The standard Laplacian-modified Bayesian score.
Mahalanobis Distance: The Mahalanobis distance (MD) is the distance to the center of the training data. The larger the MD, the less trustworthy the prediction.
Mahalanobis Distance p-value: The p-value gives the fraction of training data with an MD greater than or equal to the one for the given sample, assuming normally distributed data. The smaller the p-value, the less trustworthy the prediciton. For highly non-normal X properties (e.g., fingerprints), the MD p-value is wildly inaccurate.

Structural Similar Compounds

Name	Vidarabine	Ribavirin	Bredinin
Structure	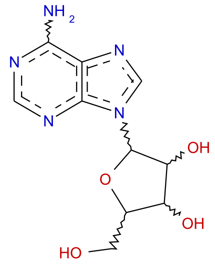	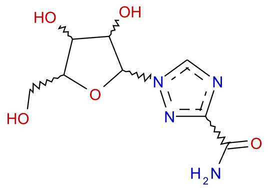	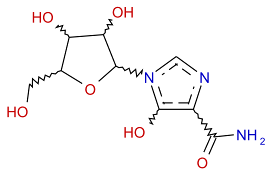
Actual Endpoint	Non-Toxic	Toxic	Toxic
Predicted Endpoint	Non-Toxic	Toxic	Toxic
Distance	0.314	0.666	0.713
Reference	Teratology 15(3):231-41; 1977	Teratology 17(1):93-101; 1978	Oyo Yakuri 26(3):377-381; 1983

Model Applicability

Unknown features are fingerprint features in the query molecule, but not found in the training set.

1. All properties and OPS components are within expected ranges.

Feature Contribution

Top features for positive contribution

Fingerprint	Bit/Smiles	Feature Structure	Score	Toxic in training set
SCFP_6	-188384666	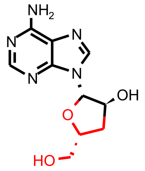 [*][C@H]1[*][*]O[C@@H]1CO	0.431	7 out of 8

SCFP_6	-1181430618	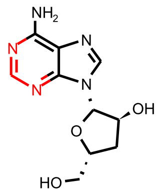 [*]:n:[cH]:n:[*]	0.298	6 out of 8
SCFP_6	-424515134	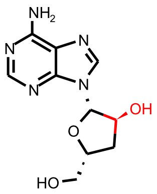 [*]C([*])O	0.275	39 out of 56

Top Features for negative contribution

Fingerprint	Bit/Smiles	Feature Structure	Score	Toxic in training set
SCFP_6	-572674910	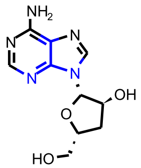 [*]n1:[*]:[*]:[c](:[*]):[c]:1:n:[*]	-0.945	0 out of 3
SCFP_6	41215856	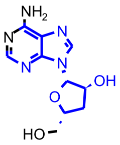 [*][C@H]1O[C@H]([C@@H](O)[C@@H]1[*])n2:[cH]:n:[c]3:[c]([*]):[*]:[cH]:n:[c]:2:3	-0.718	0 out of 2
SCFP_6	2142015375	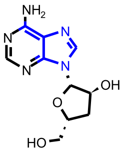 [*]n1:[cH]:n:[c](:[c]([*]):[*]):[c]:1:[*]	-0.718	0 out of 2

Ribavirin

TOPKAT_Developmental_Toxicity_Potential

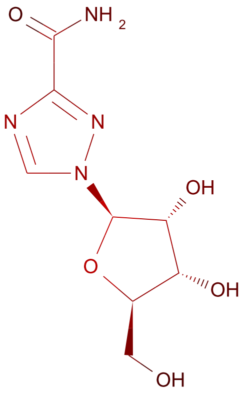

$C_8H_{12}N_4O_5$
Molecular Weight: 244.20467
ALogP: -2.745
Rotatable Bonds: 3
Acceptors: 7
Donors: 4

Structural Similar Compounds

Name	Ribavirin	Bredinin	Vidarabine
Structure	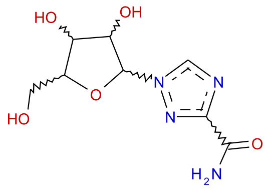	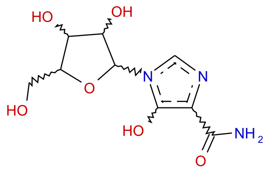	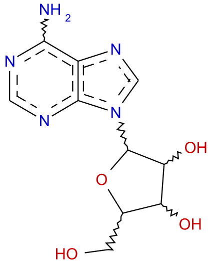
Actual Endpoint	Toxic	Toxic	Non-Toxic
Predicted Endpoint	Toxic	Toxic	Non-Toxic
Distance	0.000	0.436	0.527
Reference	Teratology 17(1):93-101; 1978	Oyo Yakuri 26(3):377-381; 1983	Teratology 15(3):231-41; 1977

Model Prediction

Prediction: Toxic
Probability: 0.791
Enrichment: 1.503
Bayesian Score: 6.415
Mahalanobis Distance: 6.567
Mahalanobis Distance p-value: 0.985

Prediction: Positive if the Bayesian score is above the estimated best cutoff value from minimizing the false positive and false negative rate.
Probability: The esimated probability that the sample is in the positive category. This assumes that the Bayesian score follows a normal distribution and is different from the prediction using a cutoff.
Enrichment: An estimate of enrichment, that is, the increased likelihood (versus random) of this sample being in the category.
Bayesian Score: The standard Laplacian-modified Bayesian score.
Mahalanobis Distance: The Mahalanobis distance (MD) is the distance to the center of the training data. The larger the MD, the less trustworthy the prediction.
Mahalanobis Distance p-value: The p-value gives the fraction of training data with an MD greater than or equal to the one for the given sample, assuming normally distributed data. The smaller the p-value, the less trustworthy the prediciton. For highly non-normal X properties (e.g., fingerprints), the MD p-value is wildly inaccurate.

Model Applicability

Unknown features are fingerprint features in the query molecule, but not found in the training set.

1. All properties and OPS components are within expected ranges.

Feature Contribution

Top features for positive contribution

Fingerprint	Bit/Smiles	Feature Structure	Score	Toxic in training set
SCFP_6	-1357949052	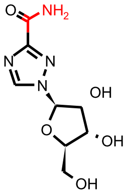 [*]C(=[*])N	0.453	8 out of 9

SCFP_6	-1486266146	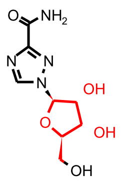 [*]C[C@H]1O[C@@H]([*])[C@@H](O)[C@@H]1O	0.431	7 out of 8
SCFP_6	-188384666	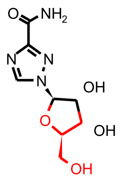 [*][C@H]1[*][*]O[C@@H]1CO	0.431	7 out of 8

Top Features for negative contribution

Fingerprint	Bit/Smiles	Feature Structure	Score	Toxic in training set
SCFP_6	149212520	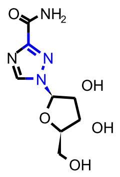 [*]n1:[*]:[*]:[c]([*]):n:1	-0.448	5 out of 16
SCFP_6	-443296553	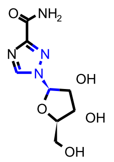 [*]C([*])n1:[cH]:[*]:[*]:n:1	-0.324	2 out of 6
SCFP_6	8	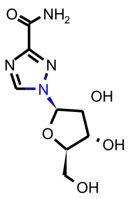 [*]:n:[*]	-0.278	24 out of 61

AND Enantiomer

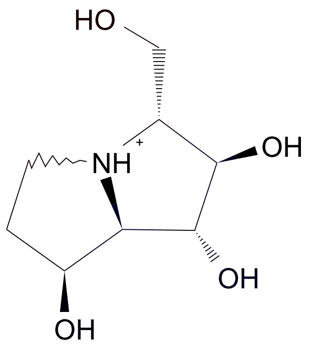

$C_8H_{16}NO_4$
Molecular Weight: 190.21693
ALogP: -3.556
Rotatable Bonds: 1
Acceptors: 4
Donors: 5

Model Prediction

Prediction: Non-Carcinogen
Probability: 0.245
Enrichment: 0.765
Bayesian Score: -0.985
Mahalanobis Distance: 9.503
Mahalanobis Distance p-value: 0.674

Prediction: Positive if the Bayesian score is above the estimated best cutoff value from minimizing the false positive and false negative rate.
Probability: The esimated probability that the sample is in the positive category. This assumes that the Bayesian score follows a normal distribution and is different from the prediction using a cutoff.
Enrichment: An estimate of enrichment, that is, the increased likelihood (versus random) of this sample being in the category.
Bayesian Score: The standard Laplacian-modified Bayesian score.
Mahalanobis Distance: The Mahalanobis distance (MD) is the distance to the center of the training data. The larger the MD, the less trustworthy the prediction.
Mahalanobis Distance p-value: The p-value gives the fraction of training data with an MD greater than or equal to the one for the given sample, assuming normally distributed data. The smaller the p-value, the less trustworthy the prediciton. For highly non-normal X properties (e.g., fingerprints), the MD p-value is wildly inaccurate.

Structural Similar Compounds

Name	Methyldopa	Ribavirin	Ascorbic acid
Structure	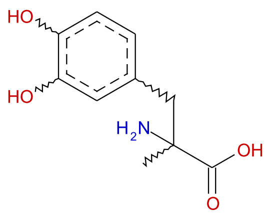	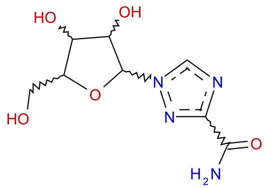	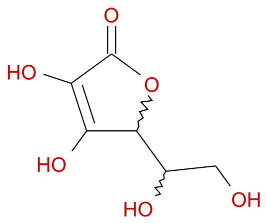
Actual Endpoint	Non-Carcinogen	Non-Carcinogen	Non-Carcinogen
Predicted Endpoint	Non-Carcinogen	Non-Carcinogen	Non-Carcinogen
Distance	0.749	0.831	0.832
Reference	US FDA (Centre for Drug Eval.& Res./Off. Testing & Res.) Sept. 1997	US FDA (Centre for Drug Eval.& Res./Off. Testing & Res.) Sept. 1997	US FDA (Centre for Drug Eval.& Res./Off. Testing & Res.) Sept. 1997

Model Applicability

Unknown features are fingerprint features in the query molecule, but not found in the training set.

1. All properties and OPS components are within expected ranges.
2. Unknown ECFP_2 feature: 1976330679: [*][NH+]([*])[*]
3. Unknown ECFP_2 feature: -1693770146: [*][C@@H]1[*][*][C@H]2[*][*]C[NH+]12
4. Unknown ECFP_2 feature: -1395316370: [*][C@H]1[*][*][NH+]2[*][*][C@H]([*])C12
5. Unknown ECFP_2 feature: -705668329: [*]C[C@@H]1[C@@H]([*])[*][*][NH+]1[*]
6. Unknown ECFP_2 feature: -244159614: [*][NH+]1[*][*]CC1

Feature Contribution

Top features for positive contribution				
**Fingerprint**	**Bit/Smiles**	**Feature Structure**	**Score**	**Carcinogen in training set**
ECFP_6	-85480422	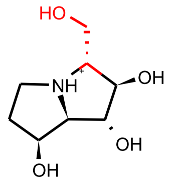 [*]C([*])CO	0.337	3 out of 6

ECFP_6	-1331450522	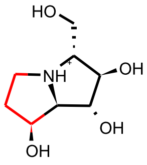<br>[*][C@@H]1[*][*]CC1	0.238	25 out of 63
ECFP_6	2022454958	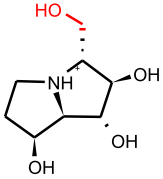<br>[*]CO	0.135	9 out of 25
**Top Features for negative contribution**				
**Fingerprint**	**Bit/Smiles**	**Feature Structure**	**Score**	**Carcinogen in training set**
ECFP_6	305695353	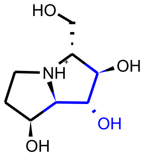<br>[*][C@@H]1[*][*][C@H]([*])[C@H]1O	-0.638	1 out of 9
ECFP_6	-329826665	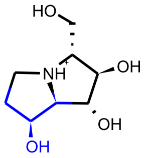<br>[*][C@@H]1[*][*]C[C@@H]1O	-0.232	2 out of 9
ECFP_6	1559650422	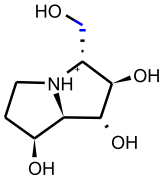<br>[*]C[*]	-0.164	50 out of 191

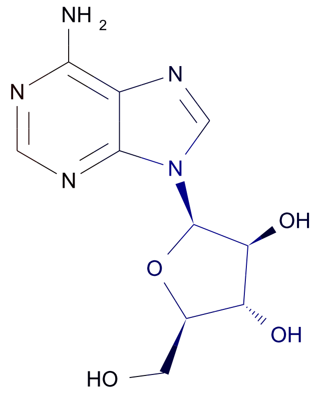

$C_{10}H_{13}N_5O_4$
Molecular Weight: 267.24132
ALogP: -1.881
Rotatable Bonds: 2
Acceptors: 8
Donors: 4

Model Prediction

Prediction: Non-Carcinogen
Probability: 0.210
Enrichment: 0.654
Bayesian Score: -4.417
Mahalanobis Distance: 9.636
Mahalanobis Distance p-value: 0.614

Prediction: Positive if the Bayesian score is above the estimated best cutoff value from minimizing the false positive and false negative rate.
Probability: The esimated probability that the sample is in the positive category. This assumes that the Bayesian score follows a normal distribution and is different from the prediction using a cutoff.
Enrichment: An estimate of enrichment, that is, the increased likelihood (versus random) of this sample being in the category.
Bayesian Score: The standard Laplacian-modified Bayesian score.
Mahalanobis Distance: The Mahalanobis distance (MD) is the distance to the center of the training data. The larger the MD, the less trustworthy the prediction.
Mahalanobis Distance p-value: The p-value gives the fraction of training data with an MD greater than or equal to the one for the given sample, assuming normally distributed data. The smaller the p-value, the less trustworthy the prediciton. For highly non-normal X properties (e.g., fingerprints), the MD p-value is wildly inaccurate.

Structural Similar Compounds

Name	Ribavirin	Ganciclovir	Acyclovir
Structure	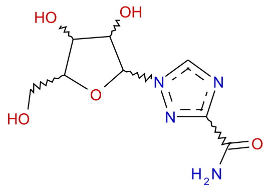	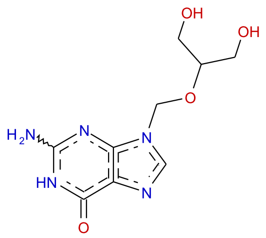	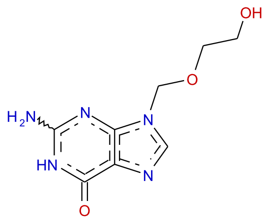
Actual Endpoint	Non-Carcinogen	Carcinogen	Non-Carcinogen
Predicted Endpoint	Non-Carcinogen	Carcinogen	Carcinogen
Distance	0.500	0.645	0.710
Reference	US FDA (Centre for Drug Eval.& Res./Off. Testing & Res.) Sept. 1997	US FDA (Centre for Drug Eval.& Res./Off. Testing & Res.) Sept. 1997	US FDA (Centre for Drug Eval.& Res./Off. Testing & Res.) Sept. 1997

Model Applicability

Unknown features are fingerprint features in the query molecule, but not found in the training set.

1. All properties and OPS components are within expected ranges.

Feature Contribution

Top features for positive contribution

Fingerprint	Bit/Smiles	Feature Structure	Score	Carcinogen in training set
ECFP_6	-1448786963	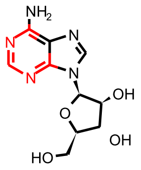 [*][c]1:[*]:[c](:[*]):n:[cH]:n:1	0.424	1 out of 1

ECFP_6	-85480422	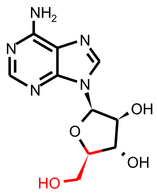 [*]C([*])CO	0.337	3 out of 6
ECFP_6	683445015	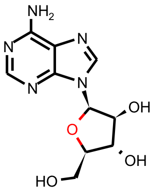 [*]O[*]	0.181	18 out of 48

Top Features for negative contribution

**Fingerprint**	**Bit/Smiles**	**Feature Structure**	**Score**	**Carcinogen in training set**
ECFP_6	305695353	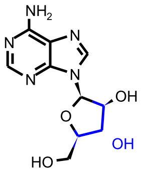 [*][C@@H]1[*][*][C@H]([*])[C@H]1O	-0.638	1 out of 9
ECFP_6	1811812564	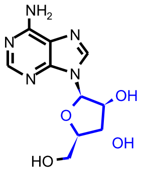 [*]C[C@H]1O[C@@H]([*])[C@@H](O)[C@@H]1O	-0.482	0 out of 2
ECFP_6	-763075316	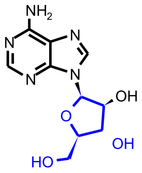 [*][C@@H]1O[C@H](CO)[C@@H](O)[C@@H]1[*]	-0.482	0 out of 2

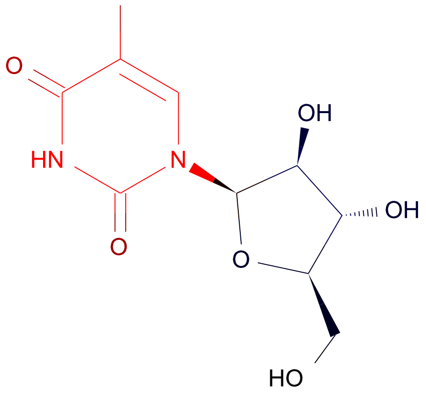

$C_{10}H_{14}N_2O_6$
Molecular Weight: 258.22795
ALogP: -2.005
Rotatable Bonds: 2
Acceptors: 6
Donors: 4

Model Prediction

Prediction: Carcinogen
Probability: 0.458
Enrichment: 1.429
Bayesian Score: 5.731
Mahalanobis Distance: 10.179
Mahalanobis Distance p-value: 0.365

Prediction: Positive if the Bayesian score is above the estimated best cutoff value from minimizing the false positive and false negative rate.
Probability: The esimated probability that the sample is in the positive category. This assumes that the Bayesian score follows a normal distribution and is different from the prediction using a cutoff.
Enrichment: An estimate of enrichment, that is, the increased likelihood (versus random) of this sample being in the category.
Bayesian Score: The standard Laplacian-modified Bayesian score.
Mahalanobis Distance: The Mahalanobis distance (MD) is the distance to the center of the training data. The larger the MD, the less trustworthy the prediction.
Mahalanobis Distance p-value: The p-value gives the fraction of training data with an MD greater than or equal to the one for the given sample, assuming normally distributed data. The smaller the p-value, the less trustworthy the prediciton. For highly non-normal X properties (e.g., fingerprints), the MD p-value is wildly inaccurate.

Structural Similar Compounds

Name	Zidovudine	Methyldopa	Ribavirin
Structure	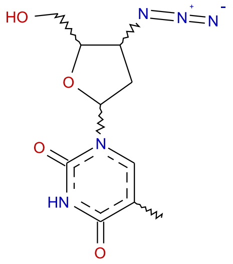	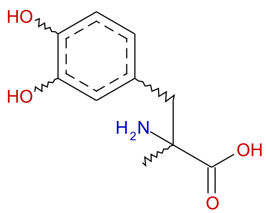	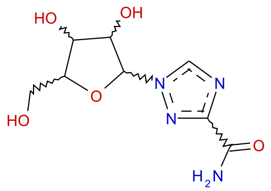
Actual Endpoint	Carcinogen	Non-Carcinogen	Non-Carcinogen
Predicted Endpoint	Carcinogen	Non-Carcinogen	Non-Carcinogen
Distance	0.528	0.597	0.603
Reference	US FDA (Centre for Drug Eval.& Res./Off. Testing & Res.) Sept. 1997	US FDA (Centre for Drug Eval.& Res./Off. Testing & Res.) Sept. 1997	US FDA (Centre for Drug Eval.& Res./Off. Testing & Res.) Sept. 1997

Model Applicability

Unknown features are fingerprint features in the query molecule, but not found in the training set.

1. All properties and OPS components are within expected ranges.
2. Unknown ECFP_2 feature: -627157165: [*][C@H]1[*][*]O[C@H]1N([*])[*]

Feature Contribution

Top features for positive contribution

Fingerprint	Bit/Smiles	Feature Structure	Score	Carcinogen in training set
ECFP_6	1526392165	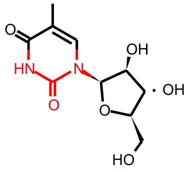 [*]NC(=O)N([*])[*]	0.742	5 out of 6

ECFP_6	-3067141	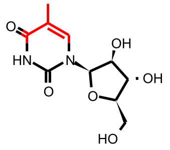 [*]\C=C(\C)/C(=[*])[*]	0.675	4 out of 5
ECFP_6	1231005866	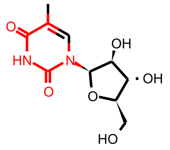 [*]N1[*]=C([*])C(=O)N C1=O	0.617	2 out of 2

Top Features for negative contribution

Fingerprint	Bit/Smiles	Feature Structure	Score	Carcinogen in training set
ECFP_6	305695353	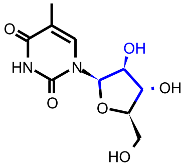 [*][C@@H]1[*][*][C@H] ([*])[C@H]1O	-0.638	1 out of 9
ECFP_6	-763075316	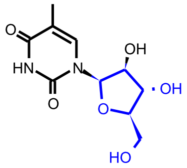 [*][C@@H]1O[C@H](CO)[C@@H](O)[C@@H]1[*]	-0.482	0 out of 2
ECFP_6	1811812564	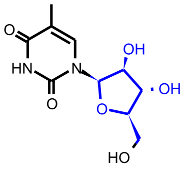 [*]C[C@H]1O[C@@H]([*])[C@@H](O)[C@@H]1O	-0.482	0 out of 2

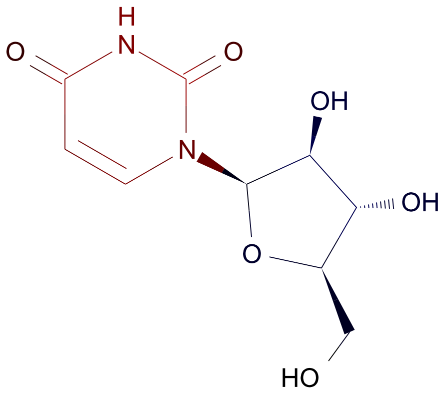

$C_9H_{12}N_2O_6$
Molecular Weight: 244.20137
ALogP: -2.451
Rotatable Bonds: 2
Acceptors: 6
Donors: 4

Model Prediction

Prediction: Carcinogen
Probability: 0.335
Enrichment: 1.045
Bayesian Score: 2.736
Mahalanobis Distance: 9.889
Mahalanobis Distance p-value: 0.497

Prediction: Positive if the Bayesian score is above the estimated best cutoff value from minimizing the false positive and false negative rate.
Probability: The esimated probability that the sample is in the positive category. This assumes that the Bayesian score follows a normal distribution and is different from the prediction using a cutoff.
Enrichment: An estimate of enrichment, that is, the increased likelihood (versus random) of this sample being in the category.
Bayesian Score: The standard Laplacian-modified Bayesian score.
Mahalanobis Distance: The Mahalanobis distance (MD) is the distance to the center of the training data. The larger the MD, the less trustworthy the prediction.
Mahalanobis Distance p-value: The p-value gives the fraction of training data with an MD greater than or equal to the one for the given sample, assuming normally distributed data. The smaller the p-value, the less trustworthy the prediciton. For highly non-normal X properties (e.g., fingerprints), the MD p-value is wildly inaccurate.

Structural Similar Compounds

Name	Ribavirin	Methyldopa	Ascorbic acid
Structure	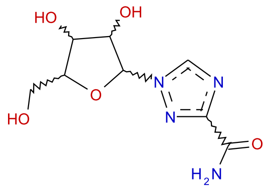	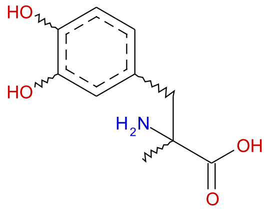	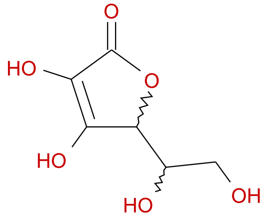
Actual Endpoint	Non-Carcinogen	Non-Carcinogen	Non-Carcinogen
Predicted Endpoint	Non-Carcinogen	Non-Carcinogen	Non-Carcinogen
Distance	0.542	0.626	0.645
Reference	US FDA (Centre for Drug Eval.& Res./Off. Testing & Res.) Sept. 1997	US FDA (Centre for Drug Eval.& Res./Off. Testing & Res.) Sept. 1997	US FDA (Centre for Drug Eval.& Res./Off. Testing & Res.) Sept. 1997

Model Applicability

Unknown features are fingerprint features in the query molecule, but not found in the training set.

1. All properties and OPS components are within expected ranges.
2. Unknown ECFP_2 feature: -627157165: [*][C@H]1[*][*]O[C@H]1N([*])[*]

Feature Contribution

Top features for positive contribution				
**Fingerprint**	**Bit/Smiles**	**Feature Structure**	**Score**	**Carcinogen in training set**
ECFP_6	1526392165	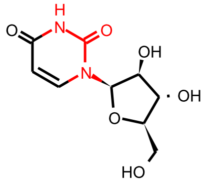 [*]NC(=O)N([*])[*]	0.742	5 out of 6

ECFP_6	-867415197	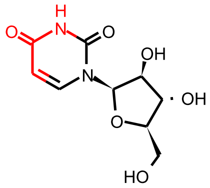 [*]NC(=O)C=[*]	0.581	3 out of 4
ECFP_6	1745066357	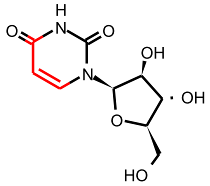 [*]\C=C/C(=[*])[*]	0.529	7 out of 12
**Top Features for negative contribution**				
**Fingerprint**	**Bit/Smiles**	**Feature Structure**	**Score**	**Carcinogen in training set**
ECFP_6	305695353	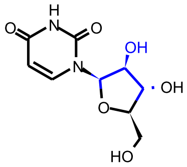 [*][C@@H]1[*][*][C@H]([*])[C@H]1O	-0.638	1 out of 9
ECFP_6	1811812564	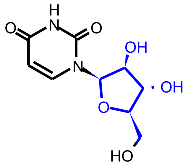 [*]C[C@H]1O[C@@H]([*])[C@@H](O)[C@@H]1O	-0.482	0 out of 2
ECFP_6	-763075316	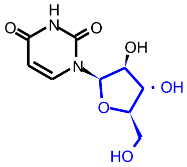 [*][C@@H]1O[C@H](CO)[C@@H](O)[C@@H]1[*]	-0.482	0 out of 2

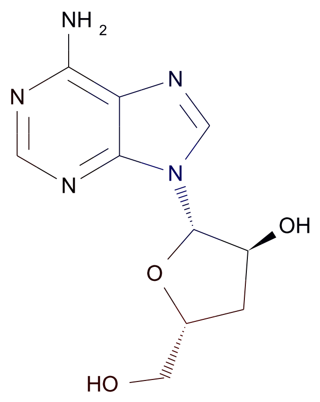

$C_{10}H_{13}N_5O_3$
Molecular Weight: 251.24191
ALogP: -1.308
Rotatable Bonds: 2
Acceptors: 7
Donors: 3

Model Prediction

Prediction: Carcinogen
Probability: 0.252
Enrichment: 0.787
Bayesian Score: -0.575
Mahalanobis Distance: 10.233
Mahalanobis Distance p-value: 0.342

Prediction: Positive if the Bayesian score is above the estimated best cutoff value from minimizing the false positive and false negative rate.
Probability: The esimated probability that the sample is in the positive category. This assumes that the Bayesian score follows a normal distribution and is different from the prediction using a cutoff.
Enrichment: An estimate of enrichment, that is, the increased likelihood (versus random) of this sample being in the category.
Bayesian Score: The standard Laplacian-modified Bayesian score.
Mahalanobis Distance: The Mahalanobis distance (MD) is the distance to the center of the training data. The larger the MD, the less trustworthy the prediction.
Mahalanobis Distance p-value: The p-value gives the fraction of training data with an MD greater than or equal to the one for the given sample, assuming normally distributed data. The smaller the p-value, the less trustworthy the prediciton. For highly non-normal X properties (e.g., fingerprints), the MD p-value is wildly inaccurate.

Structural Similar Compounds

Name	Acyclovir	Zidovudine	Didanosine
Structure	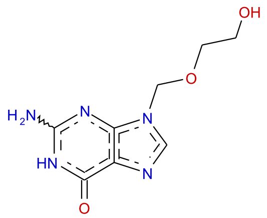	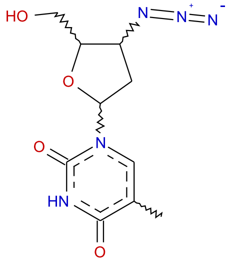	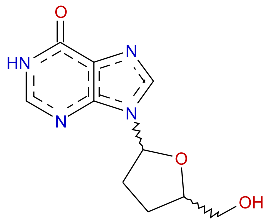
Actual Endpoint	Non-Carcinogen	Carcinogen	Non-Carcinogen
Predicted Endpoint	Carcinogen	Carcinogen	Non-Carcinogen
Distance	0.597	0.604	0.654
Reference	US FDA (Centre for Drug Eval.& Res./Off. Testing & Res.) Sept. 1997	US FDA (Centre for Drug Eval.& Res./Off. Testing & Res.) Sept. 1997	US FDA (Centre for Drug Eval.& Res./Off. Testing & Res.) Sept. 1997

Model Applicability

Unknown features are fingerprint features in the query molecule, but not found in the training set.

1. All properties and OPS components are within expected ranges.

Feature Contribution

Top features for positive contribution				
Fingerprint	Bit/Smiles	Feature Structure	Score	Carcinogen in training set
ECFP_6	376664744	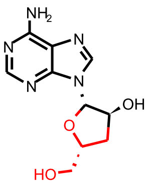 OC[C@H]1C[*][*]O1	0.442	2 out of 3

ECFP_6	-1448786963	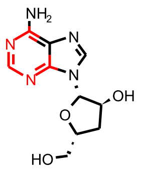 [*][c]1:[*]:[c](:[*]) :n:[cH]:n:1	0.424	1 out of 1
ECFP_6	-554480104	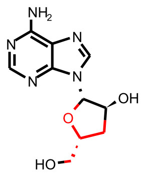 [*]C[C@H]1C[*][*]O1	0.364	4 out of 8

Top Features for negative contribution

Fingerprint	Bit/Smiles	Feature Structure	Score	Carcinogen in training set
ECFP_6	672362763	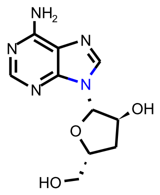 [*]n(:[*]):[*]	-0.410	5 out of 26
ECFP_6	-708878603	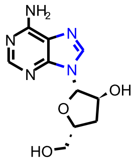 [*]n1:[*]:[*]:n:[cH]: 1	-0.384	2 out of 11
ECFP_6	1048320787	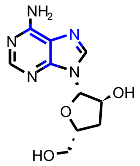 [*][c](:[*]):[c]1:n:[*]:[*]:[c]:1:[*]	-0.356	1 out of 6

Ribavirin

TOPKAT_Mouse_Female_FDA_None_vs_Carcinogen

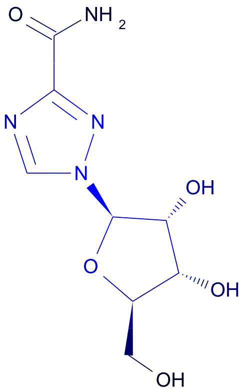

$C_8H_{12}N_4O_5$
Molecular Weight: 244.20467
ALogP: -2.745
Rotatable Bonds: 3
Acceptors: 7
Donors: 4

Structural Similar Compounds

Name	Ribavirin	Ganciclovir	Ascorbic acid
Structure	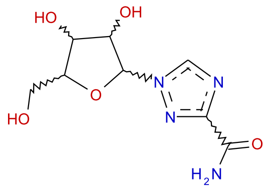	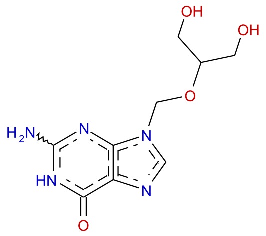	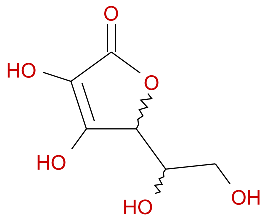
Actual Endpoint	Non-Carcinogen	Carcinogen	Non-Carcinogen
Predicted Endpoint	Non-Carcinogen	Carcinogen	Non-Carcinogen
Distance	0.000	0.621	0.639
Reference	US FDA (Centre for Drug Eval.& Res./Off. Testing & Res.) Sept. 1997	US FDA (Centre for Drug Eval.& Res./Off. Testing & Res.) Sept. 1997	US FDA (Centre for Drug Eval.& Res./Off. Testing & Res.) Sept. 1997

Model Prediction

Prediction: Non-Carcinogen
Probability: 0.212
Enrichment: 0.662
Bayesian Score: -8.673
Mahalanobis Distance: 9.227
Mahalanobis Distance p-value: 0.784

Prediction: Positive if the Bayesian score is above the estimated best cutoff value from minimizing the false positive and false negative rate.
Probability: The esimated probability that the sample is in the positive category. This assumes that the Bayesian score follows a normal distribution and is different from the prediction using a cutoff.
Enrichment: An estimate of enrichment, that is, the increased likelihood (versus random) of this sample being in the category.
Bayesian Score: The standard Laplacian-modified Bayesian score.
Mahalanobis Distance: The Mahalanobis distance (MD) is the distance to the center of the training data. The larger the MD, the less trustworthy the prediction.
Mahalanobis Distance p-value: The p-value gives the fraction of training data with an MD greater than or equal to the one for the given sample, assuming normally distributed data. The smaller the p-value, the less trustworthy the prediciton. For highly non-normal X properties (e.g., fingerprints), the MD p-value is wildly inaccurate.

Model Applicability

Unknown features are fingerprint features in the query molecule, but not found in the training set.

1. All properties and OPS components are within expected ranges.

Feature Contribution

Top features for positive contribution				
**Fingerprint**	**Bit/Smiles**	**Feature Structure**	**Score**	**Carcinogen in training set**
ECFP_6	-85480422	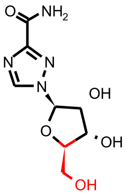 [*]C([*])CO	0.337	3 out of 6

ECFP_6	852414842	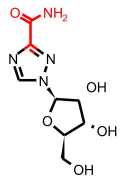<br>[*]:[c](:[*])C(=O)N	0.234	3 out of 7
ECFP_6	683445015	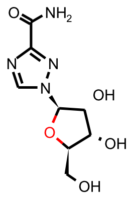<br>[*]O[*]	0.181	18 out of 48
**Top Features for negative contribution**				
**Fingerprint**	**Bit/Smiles**	**Feature Structure**	**Score**	**Carcinogen in training set**
ECFP_6	835630791	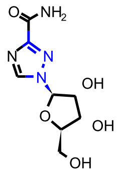<br>[*]n1:[*]:[*]:[c]([*])):n:1	-0.657	0 out of 3
ECFP_6	305695353	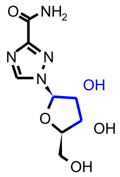<br>[*][C@@H]1[*][*][C@H]([*])[C@H]1O	-0.638	1 out of 9
ECFP_6	-763075316	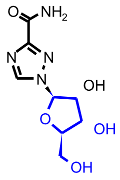<br>[*][C@@H]1O[C@H](CO)[C@@H](O)[C@@H]1[*]	-0.482	0 out of 2

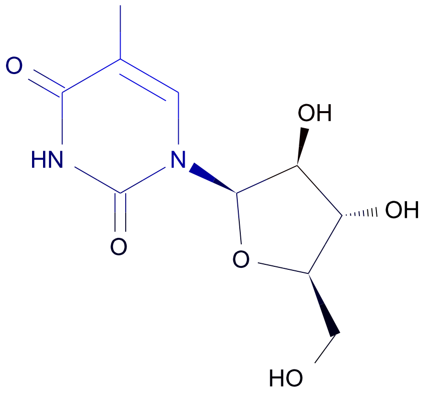

$C_{10}H_{14}N_2O_6$
Molecular Weight: 258.22795
ALogP: -2.005
Rotatable Bonds: 2
Acceptors: 6
Donors: 4

Model Prediction

Prediction: Single-Carcinogen
Probability: 0.258
Enrichment: 0.630
Bayesian Score: -5.223
Mahalanobis Distance: 11.059
Mahalanobis Distance p-value: 0.00245

Prediction: Positive if the Bayesian score is above the estimated best cutoff value from minimizing the false positive and false negative rate.
Probability: The esimated probability that the sample is in the positive category. This assumes that the Bayesian score follows a normal distribution and is different from the prediction using a cutoff.
Enrichment: An estimate of enrichment, that is, the increased likelihood (versus random) of this sample being in the category.
Bayesian Score: The standard Laplacian-modified Bayesian score.
Mahalanobis Distance: The Mahalanobis distance (MD) is the distance to the center of the training data. The larger the MD, the less trustworthy the prediction.
Mahalanobis Distance p-value: The p-value gives the fraction of training data with an MD greater than or equal to the one for the given sample, assuming normally distributed data. The smaller the p-value, the less trustworthy the prediciton. For highly non-normal X properties (e.g., fingerprints), the MD p-value is wildly inaccurate.

Structural Similar Compounds

Name	Zidovudine	Ganciclovir	Zalcitabine
Structure	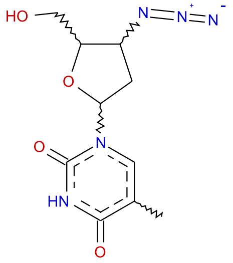	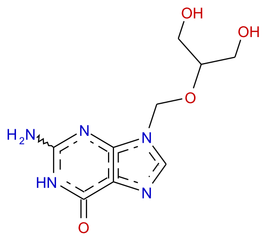	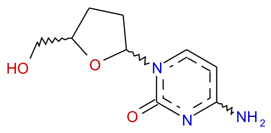
Actual Endpoint	Single-Carcinogen	Multiple-Carcinogen	Single-Carcinogen
Predicted Endpoint	Single-Carcinogen	Multiple-Carcinogen	Single-Carcinogen
Distance	0.502	0.634	0.787
Reference	US FDA (Centre for Drug Eval.& Res./Off. Testing & Res.) Sept. 1997	US FDA (Centre for Drug Eval.& Res./Off. Testing & Res.) Sept. 1997	US FDA (Centre for Drug Eval.& Res./Off. Testing & Res.) Sept. 1997

Model Applicability

Unknown features are fingerprint features in the query molecule, but not found in the training set.

1. ALogP out of range. Value: -2.005. Training min, max, mean, SD: -1.934, 10.955, 2.4263, 2.278.
2. Unknown ECFP_2 feature: -627157165: [*][C@H]1[*][*]O[C@H]1N([*])[*]

Feature Contribution

Top features for positive contribution

Fingerprint	Bit/Smiles	Feature Structure	Score	Multiple-Carcinogen in training set
ECFP_4	2024749573	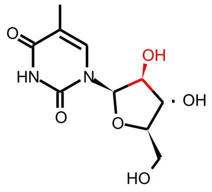 [*]C([*])O	0.494	8 out of 11

ECFP_4	2022454958	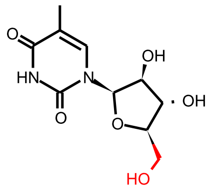 [*]CO	0.249	5 out of 9
ECFP_4	-154530762	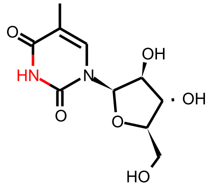 [*]N[*]	0.175	7 out of 14
**Top Features for negative contribution**				
**Fingerprint**	**Bit/Smiles**	**Feature Structure**	**Score**	**Multiple-Carcinogen in training set**
ECFP_4	-3067141	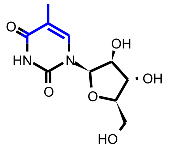 [*]\C=C(\C)/C(=[*])[*]	-0.968	0 out of 4
ECFP_4	-812320335	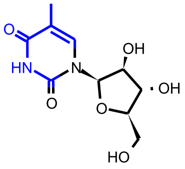 [*]=C1[*]C=C(C)C(=O)N 1	-0.800	0 out of 3
ECFP_4	596401564	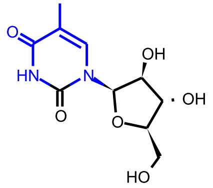 [*]N1[*]NC(=O)C(=C1)C	-0.597	0 out of 2

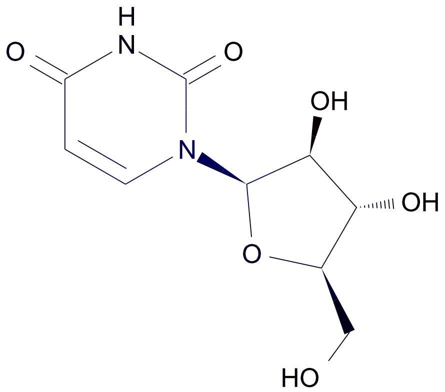

$C_9H_{12}N_2O_6$
Molecular Weight: 244.20137
ALogP: -2.451
Rotatable Bonds: 2
Acceptors: 6
Donors: 4

Model Prediction

Prediction: Single-Carcinogen
Probability: 0.332
Enrichment: 0.810
Bayesian Score: -1.983
Mahalanobis Distance: 11.480
Mahalanobis Distance p-value: 0.0011

Prediction: Positive if the Bayesian score is above the estimated best cutoff value from minimizing the false positive and false negative rate.
Probability: The esimated probability that the sample is in the positive category. This assumes that the Bayesian score follows a normal distribution and is different from the prediction using a cutoff.
Enrichment: An estimate of enrichment, that is, the increased likelihood (versus random) of this sample being in the category.
Bayesian Score: The standard Laplacian-modified Bayesian score.
Mahalanobis Distance: The Mahalanobis distance (MD) is the distance to the center of the training data. The larger the MD, the less trustworthy the prediction.
Mahalanobis Distance p-value: The p-value gives the fraction of training data with an MD greater than or equal to the one for the given sample, assuming normally distributed data. The smaller the p-value, the less trustworthy the prediciton. For highly non-normal X properties (e.g., fingerprints), the MD p-value is wildly inaccurate.

Structural Similar Compounds

Name	Ganciclovir	Zidovudine	Zalcitabine
Structure	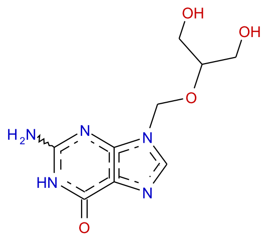	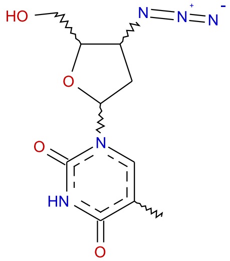	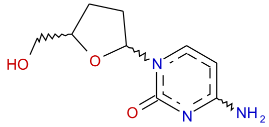
Actual Endpoint	Multiple-Carcinogen	Single-Carcinogen	Single-Carcinogen
Predicted Endpoint	Multiple-Carcinogen	Single-Carcinogen	Single-Carcinogen
Distance	0.640	0.641	0.776
Reference	US FDA (Centre for Drug Eval.& Res./Off. Testing & Res.) Sept. 1997	US FDA (Centre for Drug Eval.& Res./Off. Testing & Res.) Sept. 1997	US FDA (Centre for Drug Eval.& Res./Off. Testing & Res.) Sept. 1997

Model Applicability

Unknown features are fingerprint features in the query molecule, but not found in the training set.

1. ALogP out of range. Value: -2.451. Training min, max, mean, SD: -1.934, 10.955, 2.4263, 2.278.
2. Unknown ECFP_2 feature: -627157165: [*][C@H]1[*][*]O[C@H]1N([*])[*]

Feature Contribution

Top features for positive contribution

Fingerprint	Bit/Smiles	Feature Structure	Score	Multiple-Carcinogen in training set
ECFP_4	2024749573	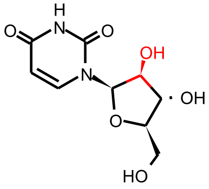 [*]C([*])O	0.494	8 out of 11

ECFP_4	1745066357	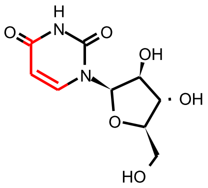 [*]\C=C/C(=[*])[*]	0.259	4 out of 7
ECFP_4	2022454958	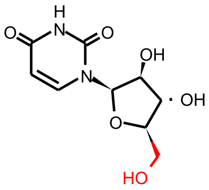 [*]CO	0.249	5 out of 9

Top Features for negative contribution

Fingerprint	Bit/Smiles	Feature Structure	Score	Multiple-Carcinogen in training set
ECFP_4	-1811420270	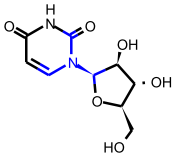 [*]C([*])N(C=[*])C(=[*])[*]	-0.597	0 out of 2
ECFP_4	305695353	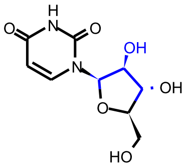 [*][C@@H]1[*][*][C@@H]([*])C1O	-0.342	0 out of 1
ECFP_4	-553149446	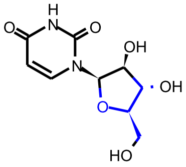 [*]C[C@H]1O[*][*][C@@H]1[*]	-0.342	0 out of 1

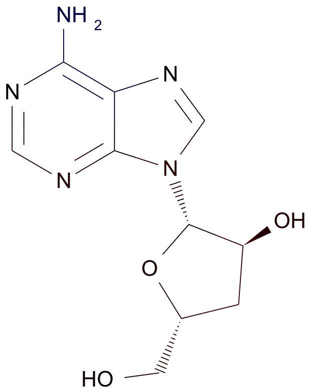

$C_{10}H_{13}N_5O_3$
Molecular Weight: 251.24191
ALogP: -1.308
Rotatable Bonds: 2
Acceptors: 7
Donors: 3

Structural Similar Compounds

Name	Zidovudine	Triamterene	Zalcitabine
Structure	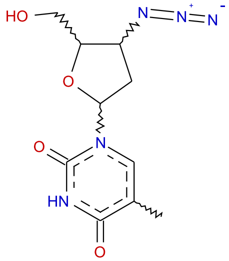	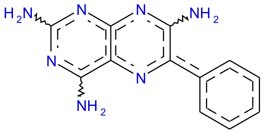	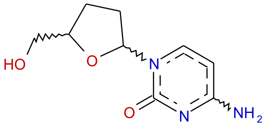
Actual Endpoint	Single-Carcinogen	Single-Carcinogen	Single-Carcinogen
Predicted Endpoint	Single-Carcinogen	Single-Carcinogen	Single-Carcinogen
Distance	0.590	0.663	0.664
Reference	US FDA (Centre for Drug Eval.& Res./Off. Testing & Res.) Sept. 1997	US FDA (Centre for Drug Eval.& Res./Off. Testing & Res.) Sept. 1997	US FDA (Centre for Drug Eval.& Res./Off. Testing & Res.) Sept. 1997

Model Prediction

Prediction: Multiple-Carcinogen
Probability: 0.456
Enrichment: 1.112
Bayesian Score: 1.266
Mahalanobis Distance: 9.452
Mahalanobis Distance p-value: 0.04

Prediction: Positive if the Bayesian score is above the estimated best cutoff value from minimizing the false positive and false negative rate.
Probability: The esimated probability that the sample is in the positive category. This assumes that the Bayesian score follows a normal distribution and is different from the prediction using a cutoff.
Enrichment: An estimate of enrichment, that is, the increased likelihood (versus random) of this sample being in the category.
Bayesian Score: The standard Laplacian-modified Bayesian score.
Mahalanobis Distance: The Mahalanobis distance (MD) is the distance to the center of the training data. The larger the MD, the less trustworthy the prediction.
Mahalanobis Distance p-value: The p-value gives the fraction of training data with an MD greater than or equal to the one for the given sample, assuming normally distributed data. The smaller the p-value, the less trustworthy the prediciton. For highly non-normal X properties (e.g., fingerprints), the MD p-value is wildly inaccurate.

Model Applicability

Unknown features are fingerprint features in the query molecule, but not found in the training set.

1. All properties and OPS components are within expected ranges.
2. Unknown ECFP_2 feature: 1049075205: [*]n1:[*]:[*]:[c](:[*]):[c]:1:n:[*]
3. Unknown ECFP_2 feature: -1219098860: [*]C([*])n1:[c](:[*]):[*]:[*]:c:1
4. Unknown ECFP_2 feature: 125442029: [*][C@H]1[*][*]O[C@@H]1n(:[*]):[*]

Feature Contribution

Top features for positive contribution

Fingerprint	Bit/Smiles	Feature Structure	Score	Multiple-Carcinogen in training set
ECFP_4	-708878603	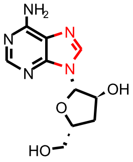 [*]n1:[*]:[*]:n:[cH]:1	0.501	2 out of 2

ECFP_4	2024749573	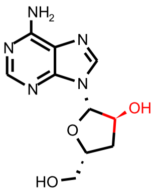 [*]C([*])O	0.494	8 out of 11
ECFP_4	-801490360	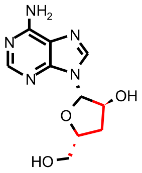 [*][C@@H]1[*][*][C@@H]([*])C1	0.422	8 out of 12

Top Features for negative contribution

Fingerprint	Bit/Smiles	Feature Structure	Score	Multiple-Carcinogen in training set
ECFP_4	-1734834311	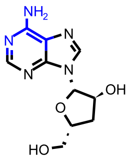 [*]:n:[c](N):[c](:[*]):[*]	-0.597	0 out of 2
ECFP_4	-938530932	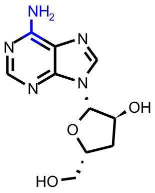 [*]:[c](:[*])N	-0.352	2 out of 8
ECFP_4	1048320787	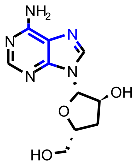 [*][c](:[*]):[c]1:n:[*]:[*]:[c]:1:[*]	-0.342	0 out of 1

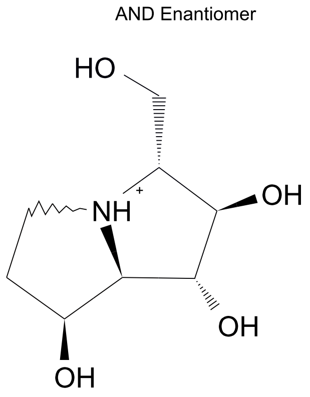

$C_8H_{16}NO_4$
Molecular Weight: 190.21693
ALogP: -3.556
Rotatable Bonds: 1
Acceptors: 4
Donors: 5

Model Prediction

Prediction: Carcinogen
Probability: 0.285
Enrichment: 0.969
Bayesian Score: -0.781
Mahalanobis Distance: 7.806
Mahalanobis Distance p-value: 0.993

Prediction: Positive if the Bayesian score is above the estimated best cutoff value from minimizing the false positive and false negative rate.
Probability: The esimated probability that the sample is in the positive category. This assumes that the Bayesian score follows a normal distribution and is different from the prediction using a cutoff.
Enrichment: An estimate of enrichment, that is, the increased likelihood (versus random) of this sample being in the category.
Bayesian Score: The standard Laplacian-modified Bayesian score.
Mahalanobis Distance: The Mahalanobis distance (MD) is the distance to the center of the training data. The larger the MD, the less trustworthy the prediction.
Mahalanobis Distance p-value: The p-value gives the fraction of training data with an MD greater than or equal to the one for the given sample, assuming normally distributed data. The smaller the p-value, the less trustworthy the prediciton. For highly non-normal X properties (e.g., fingerprints), the MD p-value is wildly inaccurate.

Structural Similar Compounds

Name	Methyldopa	Mannitol	Ascorbic acid
Structure	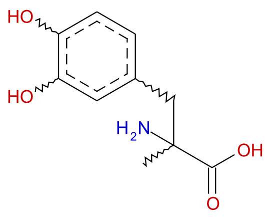	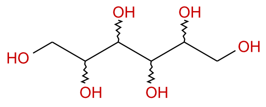	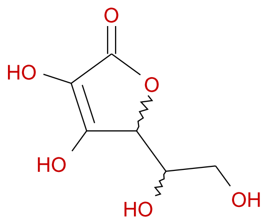
Actual Endpoint	Non-Carcinogen	Non-Carcinogen	Non-Carcinogen
Predicted Endpoint	Non-Carcinogen	Non-Carcinogen	Non-Carcinogen
Distance	0.738	0.810	0.813
Reference	US FDA (Centre for Drug Eval.& Res./Off. Testing & Res.) Sept. 1997	US FDA (Centre for Drug Eval.& Res./Off. Testing & Res.) Sept. 1997	US FDA (Centre for Drug Eval.& Res./Off. Testing & Res.) Sept. 1997

Model Applicability

Unknown features are fingerprint features in the query molecule, but not found in the training set.

1. All properties and OPS components are within expected ranges.
2. Unknown FCFP_2 feature: 10: [*][NH+]([*])[*]
3. Unknown FCFP_2 feature: -1853714334: [*][C@@H]1[*][*][C@H]2[*][*]C[NH+]12
4. Unknown FCFP_2 feature: -1817836174: [*][C@H]1[*][*][NH+]2[*][*][C@H]([*])C12
5. Unknown FCFP_2 feature: 1155241219: [*][NH+]1[*][*]CC1

Feature Contribution

Top features for positive contribution				
Fingerprint	Bit/Smiles	Feature Structure	Score	Carcinogen in training set
FCFP_6	0	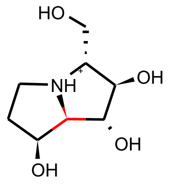 [*]C([*])[*]	0.114	90 out of 305

FCFP_6	3	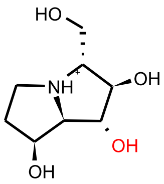 [*]O	0.105	62 out of 212
FCFP_6	-1272798659	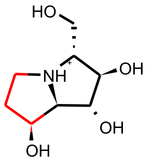 [*][C@@H]1[*][*]CC1	0.063	39 out of 139

Top Features for negative contribution

**Fingerprint**	**Bit/Smiles**	**Feature Structure**	**Score**	**Carcinogen in training set**
FCFP_6	-1272709286	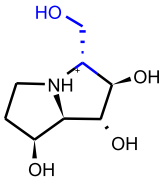 [*]C([*])CO	-0.185	16 out of 74
FCFP_6	-1043250487	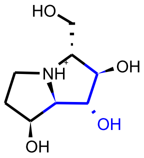 [*][C@@H]1[*][*][C@H]([*])[C@H]1O	-0.073	19 out of 78
FCFP_6	1070061035	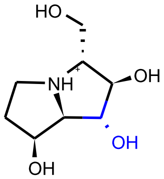 [*]C([*])O	0.000	33 out of 129

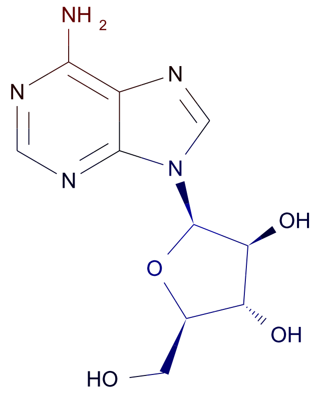

$C_{10}H_{13}N_5O_4$
Molecular Weight: 267.24132
ALogP: -1.881
Rotatable Bonds: 2
Acceptors: 8
Donors: 4

Model Prediction

Prediction: Non-Carcinogen
Probability: 0.228
Enrichment: 0.775
Bayesian Score: -3.366
Mahalanobis Distance: 11.464
Mahalanobis Distance p-value: 0.0268

Prediction: Positive if the Bayesian score is above the estimated best cutoff value from minimizing the false positive and false negative rate.
Probability: The esimated probability that the sample is in the positive category. This assumes that the Bayesian score follows a normal distribution and is different from the prediction using a cutoff.
Enrichment: An estimate of enrichment, that is, the increased likelihood (versus random) of this sample being in the category.
Bayesian Score: The standard Laplacian-modified Bayesian score.
Mahalanobis Distance: The Mahalanobis distance (MD) is the distance to the center of the training data. The larger the MD, the less trustworthy the prediction.
Mahalanobis Distance p-value: The p-value gives the fraction of training data with an MD greater than or equal to the one for the given sample, assuming normally distributed data. The smaller the p-value, the less trustworthy the prediciton. For highly non-normal X properties (e.g., fingerprints), the MD p-value is wildly inaccurate.

Structural Similar Compounds

Name	Ribavirin	Ganciclovir	Acyclovir
Structure	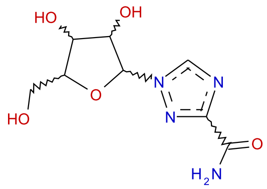	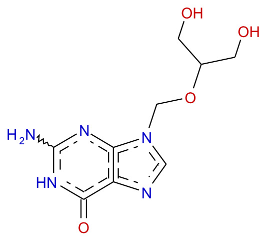	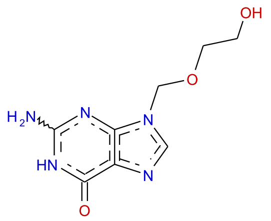
Actual Endpoint	Non-Carcinogen	Carcinogen	Non-Carcinogen
Predicted Endpoint	Non-Carcinogen	Carcinogen	Carcinogen
Distance	0.516	0.614	0.695
Reference	US FDA (Centre for Drug Eval.& Res./Off. Testing & Res.) Sept. 1997	US FDA (Centre for Drug Eval.& Res./Off. Testing & Res.) Sept. 1997	US FDA (Centre for Drug Eval.& Res./Off. Testing & Res.) Sept. 1997

Model Applicability

Unknown features are fingerprint features in the query molecule, but not found in the training set.

1. All properties and OPS components are within expected ranges.

Feature Contribution

Top features for positive contribution

Fingerprint	Bit/Smiles	Feature Structure	Score	Carcinogen in training set
FCFP_6	-576794779	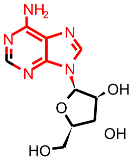 [*]n1:[cH]:n:[c]2:[c](N):n:[*]:n:[c]:1:2	0.676	2 out of 2

FCFP_6	-450797925	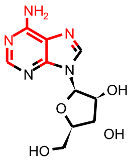 N[c]1:n:[cH]:[*]:[c]2 :[*]:[*]:n:[c]:1:2	0.676	2 out of 2
FCFP_6	-1151884458	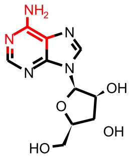 [*]:n:[c](N):[c](:[*]):[*]	0.348	6 out of 15

Top Features for negative contribution

Fingerprint	Bit/Smiles	Feature Structure	Score	Carcinogen in training set
FCFP_6	-124685461	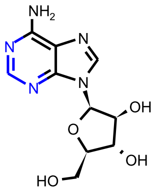 [*]:n:[cH]:n:[*]	-0.731	1 out of 12
FCFP_6	422052003	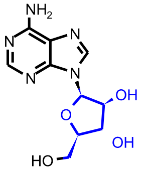 [*]C[C@H]1O[C@@H]([*])[C@@H](O)[C@@H]1O	-0.582	0 out of 3
FCFP_6	1315416442	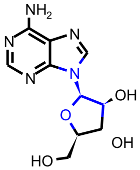 [*][C@H]1[*][*]O[C@H] 1n(:[*]):[*]	-0.423	0 out of 2

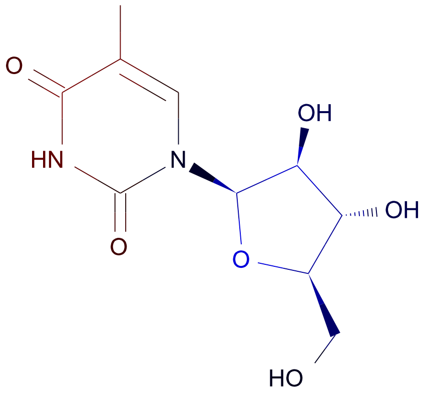

$C_{10}H_{14}N_2O_6$
Molecular Weight: 258.22795
ALogP: -2.005
Rotatable Bonds: 2
Acceptors: 6
Donors: 4

Model Prediction

Prediction: Carcinogen
Probability: 0.267
Enrichment: 0.907
Bayesian Score: -1.535
Mahalanobis Distance: 9.169
Mahalanobis Distance p-value: 0.75

Prediction: Positive if the Bayesian score is above the estimated best cutoff value from minimizing the false positive and false negative rate.
Probability: The esimated probability that the sample is in the positive category. This assumes that the Bayesian score follows a normal distribution and is different from the prediction using a cutoff.
Enrichment: An estimate of enrichment, that is, the increased likelihood (versus random) of this sample being in the category.
Bayesian Score: The standard Laplacian-modified Bayesian score.
Mahalanobis Distance: The Mahalanobis distance (MD) is the distance to the center of the training data. The larger the MD, the less trustworthy the prediction.
Mahalanobis Distance p-value: The p-value gives the fraction of training data with an MD greater than or equal to the one for the given sample, assuming normally distributed data. The smaller the p-value, the less trustworthy the prediciton. For highly non-normal X properties (e.g., fingerprints), the MD p-value is wildly inaccurate.

Structural Similar Compounds

Name	Zidovudine	Methyldopa	Ribavirin
Structure	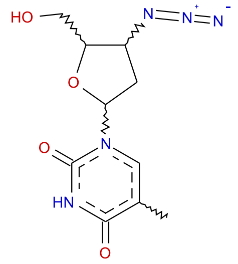	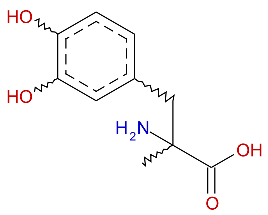	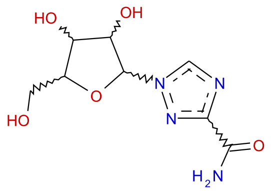
Actual Endpoint	Non-Carcinogen	Non-Carcinogen	Non-Carcinogen
Predicted Endpoint	Non-Carcinogen	Non-Carcinogen	Non-Carcinogen
Distance	0.489	0.589	0.615
Reference	US FDA (Centre for Drug Eval.& Res./Off. Testing & Res.) Sept. 1997	US FDA (Centre for Drug Eval.& Res./Off. Testing & Res.) Sept. 1997	US FDA (Centre for Drug Eval.& Res./Off. Testing & Res.) Sept. 1997

Model Applicability

Unknown features are fingerprint features in the query molecule, but not found in the training set.

1. All properties and OPS components are within expected ranges.

Feature Contribution

Top features for positive contribution

Fingerprint	Bit/Smiles	Feature Structure	Score	Carcinogen in training set
FCFP_6	201733902	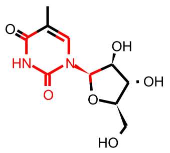 [*]C([*])N1C=[*]C(=[*])NC1=O	0.517	2 out of 3

FCFP_6	566058135	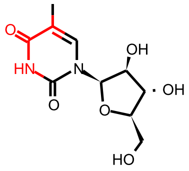 [*]NC(=O)C(=[*])[*]	0.447	17 out of 40
FCFP_6	-548679521	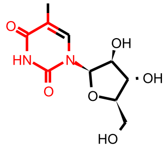 [*]N1[*]=C([*])C(=O)N C1=O	0.380	2 out of 4

Top Features for negative contribution

Fingerprint	Bit/Smiles	Feature Structure	Score	Carcinogen in training set
FCFP_6	699500266	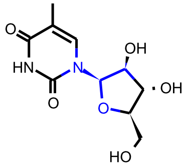 [*][C@H]1[*][*]O[C@H] 1N([*])[*]	-0.850	1 out of 14
FCFP_6	422052003	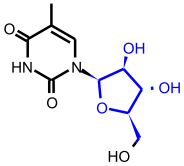 [*]C[C@H]1O[C@@H]([*])[C@@H](O)[C@@H]1O	-0.582	0 out of 3
FCFP_6	-332692398	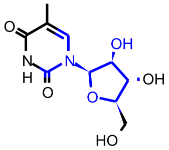 [*][C@H]1O[C@H]([C@@H](O)[C@@H]1[*])N(C=[*])C(=[*])[*]	-0.582	0 out of 3

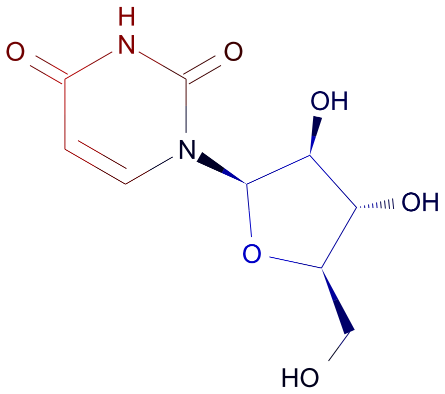

$C_9H_{12}N_2O_6$
Molecular Weight: 244.20137
ALogP: -2.451
Rotatable Bonds: 2
Acceptors: 6
Donors: 4

Model Prediction

Prediction: Carcinogen
Probability: 0.290
Enrichment: 0.986
Bayesian Score: -0.584
Mahalanobis Distance: 8.318
Mahalanobis Distance p-value: 0.961

Prediction: Positive if the Bayesian score is above the estimated best cutoff value from minimizing the false positive and false negative rate.
Probability: The esimated probability that the sample is in the positive category. This assumes that the Bayesian score follows a normal distribution and is different from the prediction using a cutoff.
Enrichment: An estimate of enrichment, that is, the increased likelihood (versus random) of this sample being in the category.
Bayesian Score: The standard Laplacian-modified Bayesian score.
Mahalanobis Distance: The Mahalanobis distance (MD) is the distance to the center of the training data. The larger the MD, the less trustworthy the prediction.
Mahalanobis Distance p-value: The p-value gives the fraction of training data with an MD greater than or equal to the one for the given sample, assuming normally distributed data. The smaller the p-value, the less trustworthy the prediciton. For highly non-normal X properties (e.g., fingerprints), the MD p-value is wildly inaccurate.

Structural Similar Compounds

Name	Ribavirin	Methyldopa	Ascorbic acid
Structure	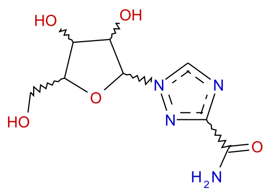	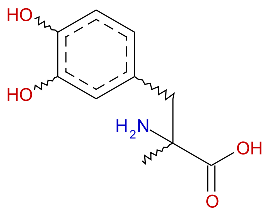	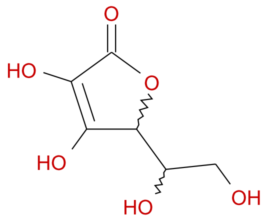
Actual Endpoint	Non-Carcinogen	Non-Carcinogen	Non-Carcinogen
Predicted Endpoint	Non-Carcinogen	Non-Carcinogen	Non-Carcinogen
Distance	0.555	0.620	0.621
Reference	US FDA (Centre for Drug Eval.& Res./Off. Testing & Res.) Sept. 1997	US FDA (Centre for Drug Eval.& Res./Off. Testing & Res.) Sept. 1997	US FDA (Centre for Drug Eval.& Res./Off. Testing & Res.) Sept. 1997

Model Applicability

Unknown features are fingerprint features in the query molecule, but not found in the training set.

1. All properties and OPS components are within expected ranges.

Feature Contribution

Top features for positive contribution

Fingerprint	Bit/Smiles	Feature Structure	Score	Carcinogen in training set
FCFP_6	1561767715	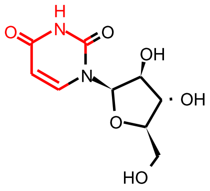 [*]=C1[*]C=CC(=O)N1	0.891	4 out of 4

FCFP_6	201733902	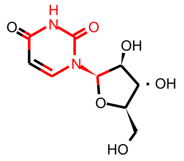 [*]C([*])N1C=[*]C(=[*])NC1=O	0.517	2 out of 3
FCFP_6	451847724	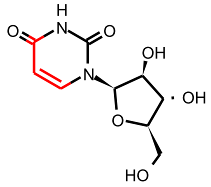 [*]\C=C/C(=[*])[*]	0.479	21 out of 48

Top Features for negative contribution

Fingerprint	Bit/Smiles	Feature Structure	Score	Carcinogen in training set
FCFP_6	699500266	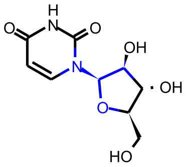 [*][C@H]1[*][*]O[C@H] 1N([*])[*]	-0.850	1 out of 14
FCFP_6	422052003	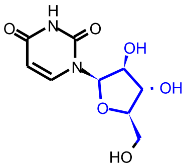 [*]C[C@H]1O[C@@H]([*])[C@@H](O)[C@@H]1O	-0.582	0 out of 3
FCFP_6	-332692398	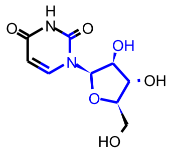 [*][C@H]1O[C@H]([C@@H](O)[C@@H]1[*])N(C=[*])C(=[*])[*]	-0.582	0 out of 3

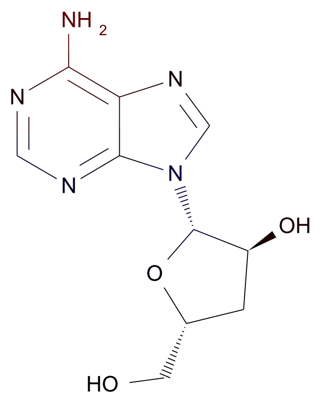

$C_{10}H_{13}N_5O_3$
Molecular Weight: 251.24191
ALogP: -1.308
Rotatable Bonds: 2
Acceptors: 7
Donors: 3

Model Prediction

Prediction: Carcinogen
Probability: 0.287
Enrichment: 0.975
Bayesian Score: -0.722
Mahalanobis Distance: 11.853
Mahalanobis Distance p-value: 0.00991

Prediction: Positive if the Bayesian score is above the estimated best cutoff value from minimizing the false positive and false negative rate.
Probability: The esimated probability that the sample is in the positive category. This assumes that the Bayesian score follows a normal distribution and is different from the prediction using a cutoff.
Enrichment: An estimate of enrichment, that is, the increased likelihood (versus random) of this sample being in the category.
Bayesian Score: The standard Laplacian-modified Bayesian score.
Mahalanobis Distance: The Mahalanobis distance (MD) is the distance to the center of the training data. The larger the MD, the less trustworthy the prediction.
Mahalanobis Distance p-value: The p-value gives the fraction of training data with an MD greater than or equal to the one for the given sample, assuming normally distributed data. The smaller the p-value, the less trustworthy the prediciton. For highly non-normal X properties (e.g., fingerprints), the MD p-value is wildly inaccurate.

Structural Similar Compounds

Name	Acyclovir	Zidovudine	Didanosine
Structure	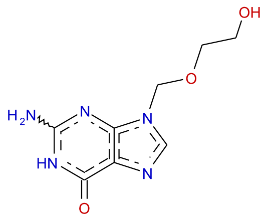	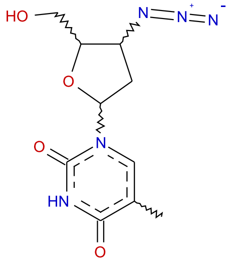	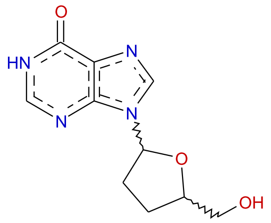
Actual Endpoint	Non-Carcinogen	Non-Carcinogen	Non-Carcinogen
Predicted Endpoint	Carcinogen	Non-Carcinogen	Non-Carcinogen
Distance	0.581	0.607	0.639
Reference	US FDA (Centre for Drug Eval.& Res./Off. Testing & Res.) Sept. 1997	US FDA (Centre for Drug Eval.& Res./Off. Testing & Res.) Sept. 1997	US FDA (Centre for Drug Eval.& Res./Off. Testing & Res.) Sept. 1997

Model Applicability

Unknown features are fingerprint features in the query molecule, but not found in the training set.

1. All properties and OPS components are within expected ranges.

Feature Contribution

Top features for positive contribution

Fingerprint	Bit/Smiles	Feature Structure	Score	Carcinogen in training set
FCFP_6	-450797925	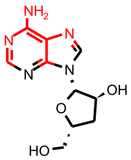 N[c]1:n:[cH]:[*]:[c]2:[*]:[*]:n:[c]:1:2	0.676	2 out of 2

FCFP_6	-576794779	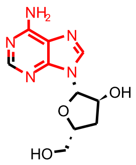 [*]n1:[cH]:n:[c]2:[c](N):n:[*]:n:[c]:1:2	0.676	2 out of 2
FCFP_6	355654354	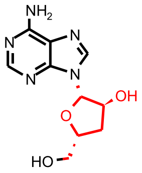 [*]C[C@H]1C[C@H](O)[C@@H]([*])O1	0.517	2 out of 3

Top Features for negative contribution

Fingerprint	Bit/Smiles	Feature Structure	Score	Carcinogen in training set
FCFP_6	-124685461	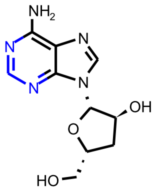 [*]:n:[cH]:n:[*]	-0.731	1 out of 12
FCFP_6	1315416442	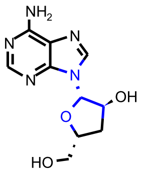 [*][C@H]1[*][*]O[C@H]1n(:[*]):[*]	-0.423	0 out of 2
FCFP_6	1864608766	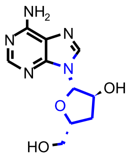 [*]C[C@H]1O[C@H]([C@@H]([*])[C@@H]1[*])n(:[*]):[*]	-0.423	0 out of 2

Ribavirin

TOPKAT_Mouse_Male_FDA_None_vs_Carcinogen

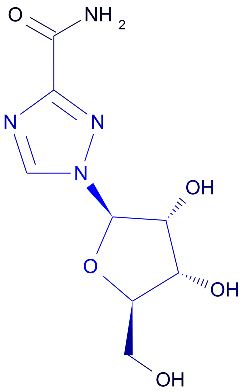

$C_8H_{12}N_4O_5$
Molecular Weight: 244.20467
ALogP: -2.745
Rotatable Bonds: 3
Acceptors: 7
Donors: 4

Model Prediction

Prediction: Non-Carcinogen
Probability: 0.165
Enrichment: 0.561
Bayesian Score: -7.533
Mahalanobis Distance: 9.864
Mahalanobis Distance p-value: 0.436

Prediction: Positive if the Bayesian score is above the estimated best cutoff value from minimizing the false positive and false negative rate.
Probability: The esimated probability that the sample is in the positive category. This assumes that the Bayesian score follows a normal distribution and is different from the prediction using a cutoff.
Enrichment: An estimate of enrichment, that is, the increased likelihood (versus random) of this sample being in the category.
Bayesian Score: The standard Laplacian-modified Bayesian score.
Mahalanobis Distance: The Mahalanobis distance (MD) is the distance to the center of the training data. The larger the MD, the less trustworthy the prediction.
Mahalanobis Distance p-value: The p-value gives the fraction of training data with an MD greater than or equal to the one for the given sample, assuming normally distributed data. The smaller the p-value, the less trustworthy the prediciton. For highly non-normal X properties (e.g., fingerprints), the MD p-value is wildly inaccurate.

Structural Similar Compounds

Name	Ribavirin	Ganciclovir	Ascorbic acid
Structure	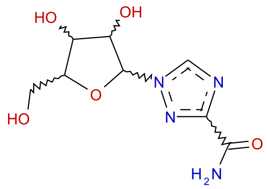	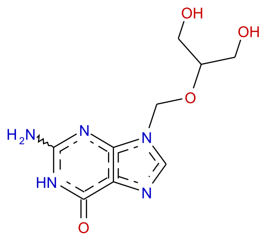	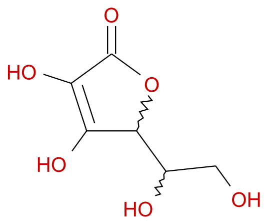
Actual Endpoint	Non-Carcinogen	Carcinogen	Non-Carcinogen
Predicted Endpoint	Non-Carcinogen	Carcinogen	Non-Carcinogen
Distance	0.000	0.583	0.606
Reference	US FDA (Centre for Drug Eval.& Res./Off. Testing & Res.) Sept. 1997	US FDA (Centre for Drug Eval.& Res./Off. Testing & Res.) Sept. 1997	US FDA (Centre for Drug Eval.& Res./Off. Testing & Res.) Sept. 1997

Model Applicability

Unknown features are fingerprint features in the query molecule, but not found in the training set.

1. All properties and OPS components are within expected ranges.

Feature Contribution

Top features for positive contribution

Fingerprint	Bit/Smiles	Feature Structure	Score	Carcinogen in training set
FCFP_6	-2049666792	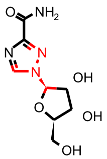 [*]C([*])n1:[cH]:[*]:[*]:n:1	0.259	2 out of 5

FCFP_6	1872154524	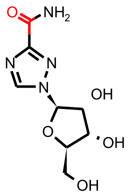 [*]C(=O)[*]	0.205	69 out of 213
FCFP_6	-1549103449	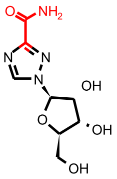 [*]:[c](:[*])C(=O)N	0.204	7 out of 21
**Top Features for negative contribution**				
**Fingerprint**	**Bit/Smiles**	**Feature Structure**	**Score**	**Carcinogen in training set**
FCFP_6	-124685461	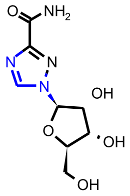 [*]:n:[cH]:n:[*]	-0.731	1 out of 12
FCFP_6	422052003	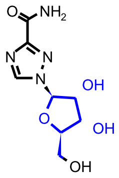 [*]C[C@H]1O[C@@H]([*])[C@@H](O)[C@@H]1O	-0.582	0 out of 3
FCFP_6	659252319	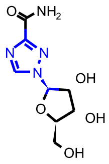 [*]C([*])n1:[cH]:n:[c]([*]):n:1	-0.582	0 out of 3

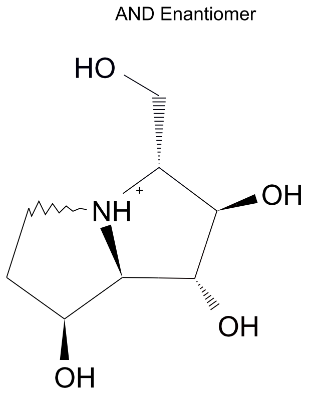

$C_8H_{16}NO_4$
Molecular Weight: 190.21693
ALogP: -3.556
Rotatable Bonds: 1
Acceptors: 4
Donors: 5

Model Prediction

Prediction: Multiple-Carcinogen
Probability: 0.261
Enrichment: 0.868
Bayesian Score: 0.047
Mahalanobis Distance: 13.070
Mahalanobis Distance p-value: 8.66e-005

Prediction: Positive if the Bayesian score is above the estimated best cutoff value from minimizing the false positive and false negative rate.
Probability: The esimated probability that the sample is in the positive category. This assumes that the Bayesian score follows a normal distribution and is different from the prediction using a cutoff.
Enrichment: An estimate of enrichment, that is, the increased likelihood (versus random) of this sample being in the category.
Bayesian Score: The standard Laplacian-modified Bayesian score.
Mahalanobis Distance: The Mahalanobis distance (MD) is the distance to the center of the training data. The larger the MD, the less trustworthy the prediction.
Mahalanobis Distance p-value: The p-value gives the fraction of training data with an MD greater than or equal to the one for the given sample, assuming normally distributed data. The smaller the p-value, the less trustworthy the prediciton. For highly non-normal X properties (e.g., fingerprints), the MD p-value is wildly inaccurate.

Structural Similar Compounds

Name	Ganciclovir	Metaproterenol	Hydrochlorothiazide
Structure	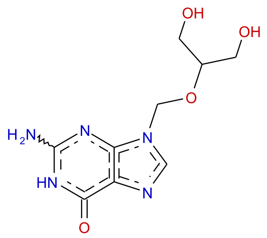	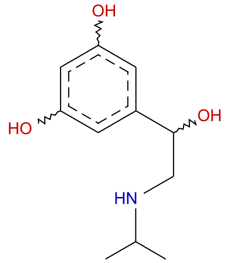	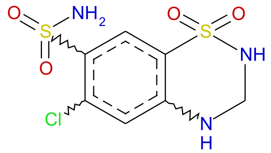
Actual Endpoint	Multiple-Carcinogen	Single-Carcinogen	Single-Carcinogen
Predicted Endpoint	Multiple-Carcinogen	Single-Carcinogen	Single-Carcinogen
Distance	0.958	0.970	1.003
Reference	US FDA (Centre for Drug Eval.& Res./Off. Testing & Res.) Sept. 1997	US FDA (Centre for Drug Eval.& Res./Off. Testing & Res.) Sept. 1997	US FDA (Centre for Drug Eval.& Res./Off. Testing & Res.) Sept. 1997

Model Applicability

Unknown features are fingerprint features in the query molecule, but not found in the training set.

1. ALogP out of range. Value: -3.556. Training min, max, mean, SD: -3.329, 10.955, 2.3788, 2.44.
2. Unknown FCFP_2 feature: 10: [*][NH+]([*])[*]
3. Unknown FCFP_2 feature: -1853714334: [*][C@@H]1[*][*][C@H]2[*][*]C[NH+]12
4. Unknown FCFP_2 feature: -1817836174: [*][C@H]1[*][*][NH+]2[*][*][C@H]([*])C12
5. Unknown FCFP_2 feature: 1155241219: [*][NH+]1[*][*]CC1

Feature Contribution

Top features for positive contribution

Fingerprint	Bit/Smiles	Feature Structure	Score	Multiple-Carcinogen in training set
FCFP_12	1070061035	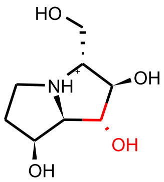 [*]C([*])O	0.135	13 out of 33

FCFP_12	-1043250487	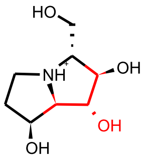<br>[*][C@@H]1[*][*][C@H]([*])[C@H]1O	0.069	7 out of 19
**Top Features for negative contribution**				
**Fingerprint**	**Bit/Smiles**	**Feature Structure**	**Score**	**Multiple-Carcinogen in training set**
FCFP_12	-1272709286	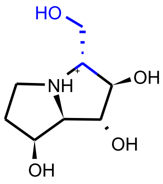<br>[*]C([*])CO	-0.254	4 out of 16
FCFP_12	3	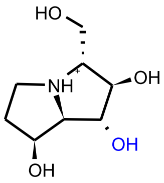<br>[*]O	-0.100	19 out of 62
FCFP_12	0	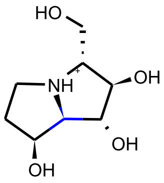<br>[*]C([*])[*]	-0.087	28 out of 90

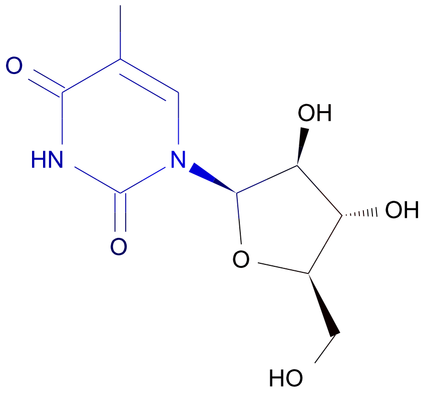

$C_{10}H_{14}N_2O_6$
Molecular Weight: 258.22795
ALogP: -2.005
Rotatable Bonds: 2
Acceptors: 6
Donors: 4

Model Prediction

Prediction: Single-Carcinogen
Probability: 0.160
Enrichment: 0.532
Bayesian Score: -5.736
Mahalanobis Distance: 10.034
Mahalanobis Distance p-value: 0.0174

Prediction: Positive if the Bayesian score is above the estimated best cutoff value from minimizing the false positive and false negative rate.
Probability: The esimated probability that the sample is in the positive category. This assumes that the Bayesian score follows a normal distribution and is different from the prediction using a cutoff.
Enrichment: An estimate of enrichment, that is, the increased likelihood (versus random) of this sample being in the category.
Bayesian Score: The standard Laplacian-modified Bayesian score.
Mahalanobis Distance: The Mahalanobis distance (MD) is the distance to the center of the training data. The larger the MD, the less trustworthy the prediction.
Mahalanobis Distance p-value: The p-value gives the fraction of training data with an MD greater than or equal to the one for the given sample, assuming normally distributed data. The smaller the p-value, the less trustworthy the prediciton. For highly non-normal X properties (e.g., fingerprints), the MD p-value is wildly inaccurate.

Structural Similar Compounds

Name	Ganciclovir	Hydrochlorothiazide	Zalcitabine
Structure	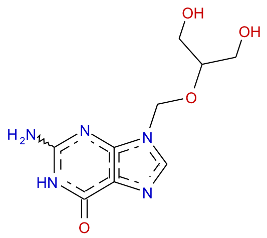	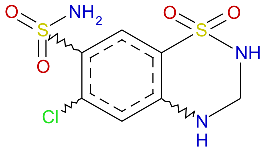	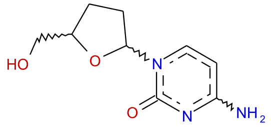
Actual Endpoint	Multiple-Carcinogen	Single-Carcinogen	Single-Carcinogen
Predicted Endpoint	Multiple-Carcinogen	Single-Carcinogen	Single-Carcinogen
Distance	0.672	0.754	0.817
Reference	US FDA (Centre for Drug Eval.& Res./Off. Testing & Res.) Sept. 1997	US FDA (Centre for Drug Eval.& Res./Off. Testing & Res.) Sept. 1997	US FDA (Centre for Drug Eval.& Res./Off. Testing & Res.) Sept. 1997

Model Applicability

Unknown features are fingerprint features in the query molecule, but not found in the training set.

1. All properties and OPS components are within expected ranges.

Feature Contribution

Top features for positive contribution

Fingerprint	Bit/Smiles	Feature Structure	Score	Multiple-Carcinogen in training set
FCFP_12	-1043310069	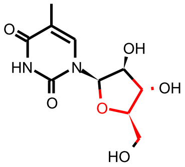 [*]C[C@H]1O[*][*][C@@H]1[*]	0.597	7 out of 10

FCFP_12	436886043	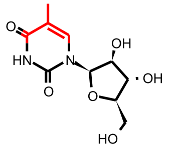[*]\C=C(\C)/C(=[*])[*<br>]	0.270	7 out of 15
FCFP_12	-2091721556	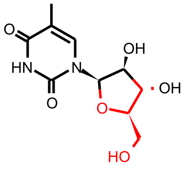[*][C@H]1[*][*]O[C@@H<br>]1CO	0.174	1 out of 2
**Top Features for negative contribution**				
**Fingerprint**	**Bit/Smiles**	**Feature Structure**	**Score**	**Multiple-Carcinogen in training set**
FCFP_12	451877515	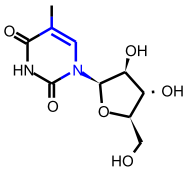[*]N([*])C=C([*])[*]	-0.704	0 out of 3
FCFP_12	566058135	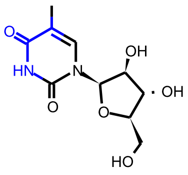[*]NC(=O)C(=[*])[*]	-0.528	3 out of 17
FCFP_12	-548679521	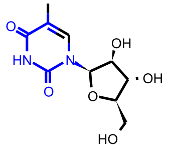[*]N1[*]=C([*])C(=O)N<br>C1=O	-0.519	0 out of 2

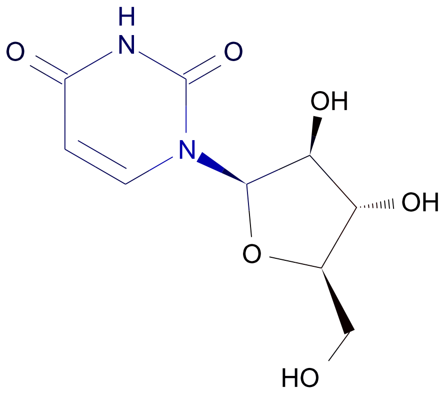

$C_9H_{12}N_2O_6$
Molecular Weight: 244.20137
ALogP: -2.451
Rotatable Bonds: 2
Acceptors: 6
Donors: 4

Model Prediction

Prediction: Multiple-Carcinogen
Probability: 0.174
Enrichment: 0.577
Bayesian Score: -4.368
Mahalanobis Distance: 8.448
Mahalanobis Distance p-value: 0.162

Prediction: Positive if the Bayesian score is above the estimated best cutoff value from minimizing the false positive and false negative rate.
Probability: The esimated probability that the sample is in the positive category. This assumes that the Bayesian score follows a normal distribution and is different from the prediction using a cutoff.
Enrichment: An estimate of enrichment, that is, the increased likelihood (versus random) of this sample being in the category.
Bayesian Score: The standard Laplacian-modified Bayesian score.
Mahalanobis Distance: The Mahalanobis distance (MD) is the distance to the center of the training data. The larger the MD, the less trustworthy the prediction.
Mahalanobis Distance p-value: The p-value gives the fraction of training data with an MD greater than or equal to the one for the given sample, assuming normally distributed data. The smaller the p-value, the less trustworthy the prediciton. For highly non-normal X properties (e.g., fingerprints), the MD p-value is wildly inaccurate.

Structural Similar Compounds

Name	Ganciclovir	Hydrochlorothiazide	Zalcitabine
Structure	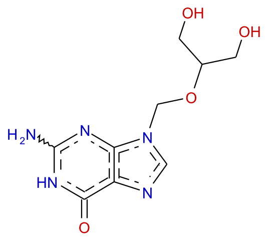	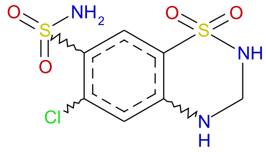	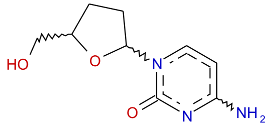
Actual Endpoint	Multiple-Carcinogen	Single-Carcinogen	Single-Carcinogen
Predicted Endpoint	Multiple-Carcinogen	Single-Carcinogen	Single-Carcinogen
Distance	0.670	0.768	0.807
Reference	US FDA (Centre for Drug Eval.& Res./Off. Testing & Res.) Sept. 1997	US FDA (Centre for Drug Eval.& Res./Off. Testing & Res.) Sept. 1997	US FDA (Centre for Drug Eval.& Res./Off. Testing & Res.) Sept. 1997

Model Applicability

Unknown features are fingerprint features in the query molecule, but not found in the training set.

1. All properties and OPS components are within expected ranges.

Feature Contribution

Top features for positive contribution

Fingerprint	Bit/Smiles	Feature Structure	Score	Multiple-Carcinogen in training set
FCFP_12	-1043310069	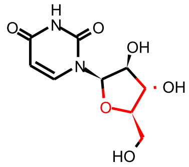 [*]C[C@H]1O[*][*][C@@H]1[*]	0.597	7 out of 10

FCFP_12	451847724	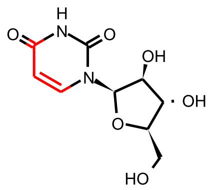<br>[*]\C=C/C(=[*])[*]	0.300	10 out of 21
FCFP_12	1561767715	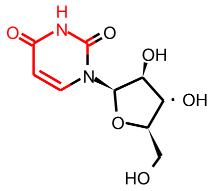<br>[*]=C1[*]C=CC(=O)N1	0.239	2 out of 4
**Top Features for negative contribution**				
**Fingerprint**	**Bit/Smiles**	**Feature Structure**	**Score**	**Multiple-Carcinogen in training set**
FCFP_12	451877515	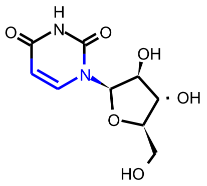<br>[*]N([*])C=C([*])[*]	-0.704	0 out of 3
FCFP_12	566058135	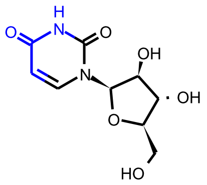<br>[*]NC(=O)C(=[*])[*]	-0.528	3 out of 17
FCFP_12	201733902	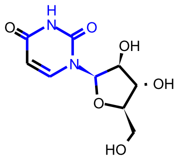<br>[*]C([*])N1C=[*]C(=[*])NC1=O	-0.519	0 out of 2

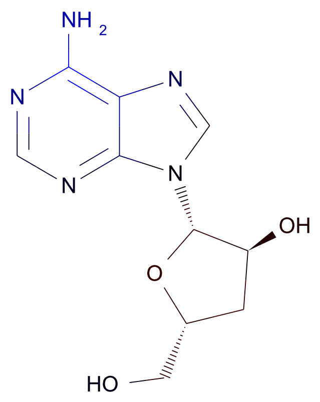

$C_{10}H_{13}N_5O_3$
Molecular Weight: 251.24191
ALogP: -1.308
Rotatable Bonds: 2
Acceptors: 7
Donors: 3

Model Prediction

Prediction: Multiple-Carcinogen
Probability: 0.172
Enrichment: 0.571
Bayesian Score: -4.515
Mahalanobis Distance: 10.631
Mahalanobis Distance p-value: 0.00656

Prediction: Positive if the Bayesian score is above the estimated best cutoff value from minimizing the false positive and false negative rate.
Probability: The esimated probability that the sample is in the positive category. This assumes that the Bayesian score follows a normal distribution and is different from the prediction using a cutoff.
Enrichment: An estimate of enrichment, that is, the increased likelihood (versus random) of this sample being in the category.
Bayesian Score: The standard Laplacian-modified Bayesian score.
Mahalanobis Distance: The Mahalanobis distance (MD) is the distance to the center of the training data. The larger the MD, the less trustworthy the prediction.
Mahalanobis Distance p-value: The p-value gives the fraction of training data with an MD greater than or equal to the one for the given sample, assuming normally distributed data. The smaller the p-value, the less trustworthy the prediciton. For highly non-normal X properties (e.g., fingerprints), the MD p-value is wildly inaccurate.

Structural Similar Compounds

Name	Triamterene	Ganciclovir	Hydrochlorothiazide
Structure	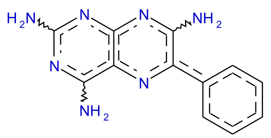	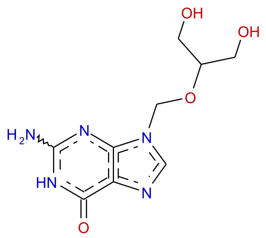	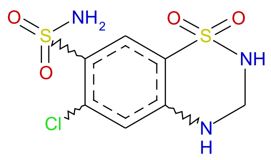
Actual Endpoint	Single-Carcinogen	Multiple-Carcinogen	Single-Carcinogen
Predicted Endpoint	Single-Carcinogen	Multiple-Carcinogen	Single-Carcinogen
Distance	0.655	0.707	0.734
Reference	US FDA (Centre for Drug Eval.& Res./Off. Testing & Res.) Sept. 1997	US FDA (Centre for Drug Eval.& Res./Off. Testing & Res.) Sept. 1997	US FDA (Centre for Drug Eval.& Res./Off. Testing & Res.) Sept. 1997

Model Applicability

Unknown features are fingerprint features in the query molecule, but not found in the training set.

1. All properties and OPS components are within expected ranges.
2. Unknown FCFP_2 feature: 1315416442: [*][C@H]1[*][*]O[C@@H]1n(:[*]):[*]

Feature Contribution

Top features for positive contribution

Fingerprint	Bit/Smiles	Feature Structure	Score	Multiple-Carcinogen in training set
FCFP_12	-1043310069	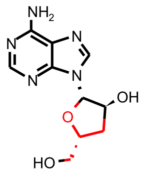 [*]C[C@H]1O[*][*][C@@H]1[*]	0.597	7 out of 10

FCFP_12	355654354	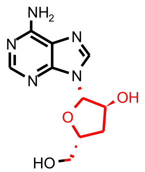 [*]C[C@H]1C[C@H](O)[C@@H]([*])O1	0.579	2 out of 2
FCFP_12	-124685461	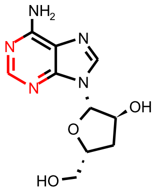 [*]:n:[cH]:n:[*]	0.400	1 out of 1
**Top Features for negative contribution**				
**Fingerprint**	**Bit/Smiles**	**Feature Structure**	**Score**	**Multiple-Carcinogen in training set**
FCFP_12	1069584379	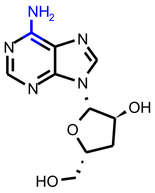 [*]:[c](:[*])N	-1.112	0 out of 6
FCFP_12	-1151884458	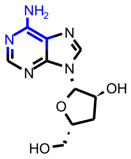 [*]:n:[c](N):[c](:[*]):[*]	-1.112	0 out of 6
FCFP_12	178336375	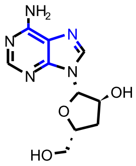 [*][c](:[*]):[c]1:n:[*]:[*]:[c]:1:[*]	-0.994	0 out of 5

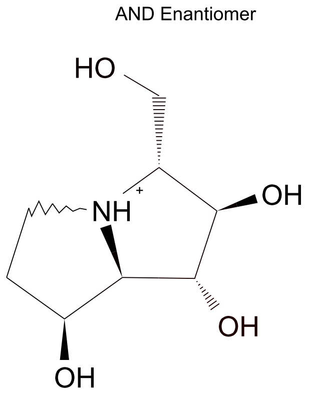

$C_8H_{16}NO_4$
Molecular Weight: 190.21693
ALogP: -3.556
Rotatable Bonds: 1
Acceptors: 4
Donors: 5

Structural Similar Compounds

Name	1;2-Ethanediamine; N-(2-aminoethyl)-N'-(2-((2-aminoethyl)amino)ethyl)-	TRIETHYLENETETRAMINE	DIETHYLENETRIAMINE
Structure	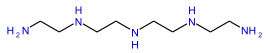	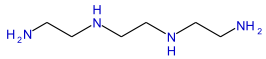	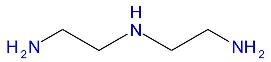
Actual Endpoint	Moderate_Severe	Moderate_Severe	Moderate_Severe
Predicted Endpoint	Moderate_Severe	Moderate_Severe	Moderate_Severe
Distance	0.776	0.786	0.966
Reference	Prehled Prumyslove Toxikologie; Organicke Latky; Marhold; J. -;446;86	UCDS**12/12/66	JIHTAB 31;60;49

Model Prediction

Prediction: Moderate_Severe
Probability: 0.835
Enrichment: 1.212
Bayesian Score: 0.895
Mahalanobis Distance: 11.858
Mahalanobis Distance p-value: 0.000128

Prediction: Positive if the Bayesian score is above the estimated best cutoff value from minimizing the false positive and false negative rate.
Probability: The esimated probability that the sample is in the positive category. This assumes that the Bayesian score follows a normal distribution and is different from the prediction using a cutoff.
Enrichment: An estimate of enrichment, that is, the increased likelihood (versus random) of this sample being in the category.
Bayesian Score: The standard Laplacian-modified Bayesian score.
Mahalanobis Distance: The Mahalanobis distance (MD) is the distance to the center of the training data. The larger the MD, the less trustworthy the prediction.
Mahalanobis Distance p-value: The p-value gives the fraction of training data with an MD greater than or equal to the one for the given sample, assuming normally distributed data. The smaller the p-value, the less trustworthy the prediciton. For highly non-normal X properties (e.g., fingerprints), the MD p-value is wildly inaccurate.

Model Applicability

Unknown features are fingerprint features in the query molecule, but not found in the training set.

1. All properties and OPS components are within expected ranges.
2. Unknown FCFP_2 feature: 10: [*][NH+]([*])[*]
3. Unknown FCFP_2 feature: -1853714334: [*][C@@H]1[*][*][C@H]2[*][*]C[NH+]12
4. Unknown FCFP_2 feature: -1817836174: [*][C@H]1[*][*][NH+]2[*][*][C@H]([*])C12
5. Unknown FCFP_2 feature: 1155241219: [*][NH+]1[*][*]CC1

Feature Contribution

Top features for positive contribution				
Fingerprint	Bit/Smiles	Feature Structure	Score	Moderate_Severe in training set
FCFP_10	-1272709286	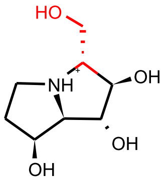 [*]C([*])CO	0.285	234 out of 266

FCFP_10	1070061035	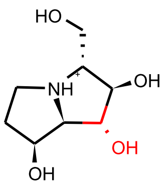<br>[*]C([*])O	0.239	284 out of 338
FCFP_10	-1043250487	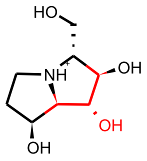<br>[*][C@@H]1[*][*][C@H]([*])[C@H]1O	0.220	62 out of 75

Top Features for negative contribution

Fingerprint	Bit/Smiles	Feature Structure	Score	Moderate_Severe in training set
FCFP_10	0	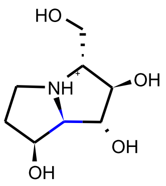<br>[*]C([*])[*]	0.000	811 out of 1184

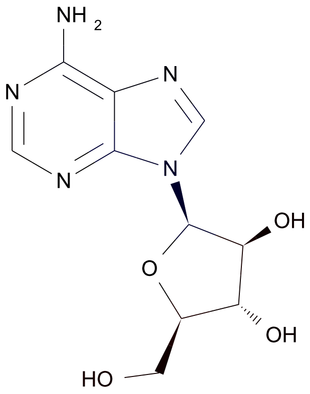

$C_{10}H_{13}N_5O_4$

Molecular Weight: 267.24132

ALogP: -1.881

Rotatable Bonds: 2

Acceptors: 8

Donors: 4

Structural Similar Compounds

Name	2;2'-Biphenyldisulfonic acid; 4;4'-diamino-	ANTHRAQUINONE;1;5-DIAMINO-4;8-DIHYDROXY-	ANTHRAQUINONE; 1;8-DIAMINO-4;5-DIHYDROXY-
Structure	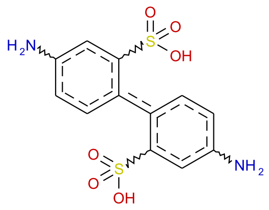	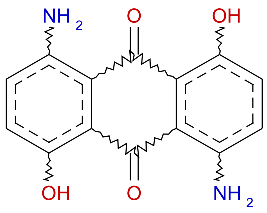	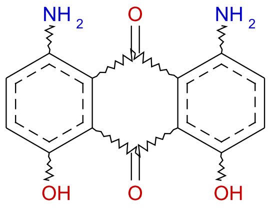
Actual Endpoint	Mild	Moderate_Severe	Moderate_Severe
Predicted Endpoint	Mild	Mild	Mild
Distance	0.747	0.810	0.810
Reference	Prehled Prumyslove Toxikologie; Organicke Latky; Marhold; J. pp 1061;86	28ZPAK-;103;72	28ZPAK-;103;72

Model Prediction

Prediction: Moderate_Severe

Probability: 0.816

Enrichment: 1.185

Bayesian Score: -0.298

Mahalanobis Distance: 10.671

Mahalanobis Distance p-value: 0.0147

Prediction: Positive if the Bayesian score is above the estimated best cutoff value from minimizing the false positive and false negative rate.
Probability: The esimated probability that the sample is in the positive category. This assumes that the Bayesian score follows a normal distribution and is different from the prediction using a cutoff.
Enrichment: An estimate of enrichment, that is, the increased likelihood (versus random) of this sample being in the category.
Bayesian Score: The standard Laplacian-modified Bayesian score.
Mahalanobis Distance: The Mahalanobis distance (MD) is the distance to the center of the training data. The larger the MD, the less trustworthy the prediction.
Mahalanobis Distance p-value: The p-value gives the fraction of training data with an MD greater than or equal to the one for the given sample, assuming normally distributed data. The smaller the p-value, the less trustworthy the prediciton. For highly non-normal X properties (e.g., fingerprints), the MD p-value is wildly inaccurate.

Model Applicability

Unknown features are fingerprint features in the query molecule, but not found in the training set.

1. All properties and OPS components are within expected ranges.
2. Unknown FCFP_2 feature: -124685461: [*]:n:c:n:[*]
3. Unknown FCFP_2 feature: -1564473960: [*]n1:[*]:[*]:[c](:[*]):[c]:1:n:[*]
4. Unknown FCFP_2 feature: -1151884458: [*]:n:[c](N):[c](:[*]):[*]
5. Unknown FCFP_2 feature: 1315416442: [*][C@H]1[*][*]O[C@H]1n(:[*]):[*]

Feature Contribution

Top features for positive contribution				
**Fingerprint**	**Bit/Smiles**	**Feature Structure**	**Score**	**Moderate_Severe in training set**

FCFP_10	-1272709286	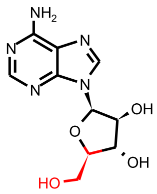 [*]C([*])CO	0.285	234 out of 266
FCFP_10	1070061035	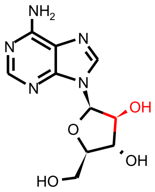 [*]C([*])O	0.239	284 out of 338
FCFP_10	-1043250487	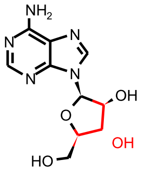 [*][C@@H]1[*][*][C@H]([*])[C@H]1O	0.220	62 out of 75

Top Features for negative contribution

Fingerprint	Bit/Smiles	Feature Structure	Score	Moderate_Severe in training set
FCFP_10	-306856457	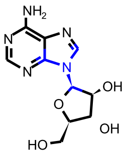 [*]C([*])n1:[cH]:[*]:[*]:[c]:1:[*]	-0.842	0 out of 2
FCFP_10	178336375	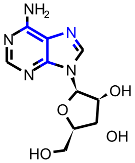 [*][c](:[*]):[c]1:n:[*]:[*]:[c]:1:[*]	-0.209	10 out of 19

FCFP_10	1069584379	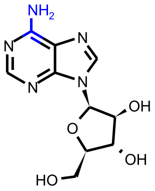 [*]:[c](:[*])N	-0.196	46 out of 85

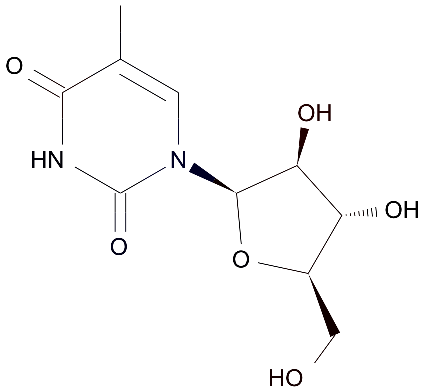

$C_{10}H_{14}N_2O_6$
Molecular Weight: 258.22795
ALogP: -2.005
Rotatable Bonds: 2
Acceptors: 6
Donors: 4

Model Prediction

Prediction: Moderate_Severe
Probability: 0.829
Enrichment: 1.204
Bayesian Score: 0.471
Mahalanobis Distance: 15.556
Mahalanobis Distance p-value: 1.42e-015

Prediction: Positive if the Bayesian score is above the estimated best cutoff value from minimizing the false positive and false negative rate.
Probability: The esimated probability that the sample is in the positive category. This assumes that the Bayesian score follows a normal distribution and is different from the prediction using a cutoff.
Enrichment: An estimate of enrichment, that is, the increased likelihood (versus random) of this sample being in the category.
Bayesian Score: The standard Laplacian-modified Bayesian score.
Mahalanobis Distance: The Mahalanobis distance (MD) is the distance to the center of the training data. The larger the MD, the less trustworthy the prediction.
Mahalanobis Distance p-value: The p-value gives the fraction of training data with an MD greater than or equal to the one for the given sample, assuming normally distributed data. The smaller the p-value, the less trustworthy the prediciton. For highly non-normal X properties (e.g., fingerprints), the MD p-value is wildly inaccurate.

Structural Similar Compounds

Name	ANTHRAQUINONE;1;5-DIAMINO-4;8-DIHYDROXY-	ANTHRAQUINONE; 1;8-DIAMINO-4;5-DIHYDROXY-	TRIETHYLENETETRAMINE
Structure	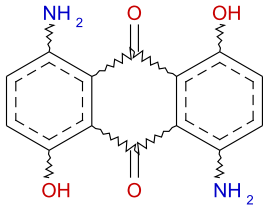	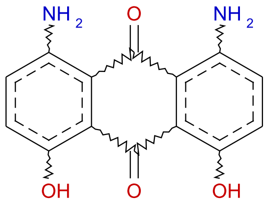	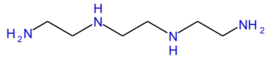
Actual Endpoint	Moderate_Severe	Moderate_Severe	Moderate_Severe
Predicted Endpoint	Mild	Mild	Moderate_Severe
Distance	0.770	0.771	0.781
Reference	28ZPAK-;103;72	28ZPAK-;103;72	UCDS**12/12/66

Model Applicability

Unknown features are fingerprint features in the query molecule, but not found in the training set.

1. All properties and OPS components are within expected ranges.

Feature Contribution

Top features for positive contribution

Fingerprint	Bit/Smiles	Feature Structure	Score	Moderate_Severe in training set
FCFP_10	-1272709286	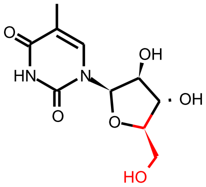 [*]C([*])CO	0.285	234 out of 266

FCFP_10	-885550502	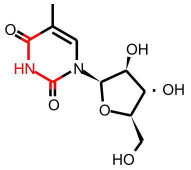 [*]C(=[*])NC(=[*])[*]	0.239	54 out of 64
FCFP_10	1070061035	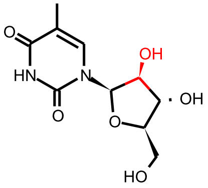 [*]C([*])O	0.239	284 out of 338

Top Features for negative contribution

Fingerprint	Bit/Smiles	Feature Structure	Score	Moderate_Severe in training set
FCFP_10	-1986098826	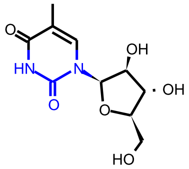 [*]NC(=O)N([*])[*]	-0.361	2 out of 5
FCFP_10	451877515	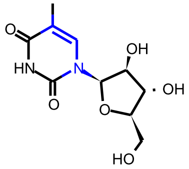 [*]N([*])C=C([*])[*]	-0.350	16 out of 35
FCFP_10	699500266	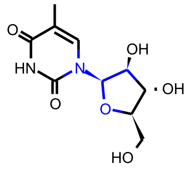 [*][C@H]1[*][*]O[C@H]1N([*])[*]	-0.103	16 out of 27

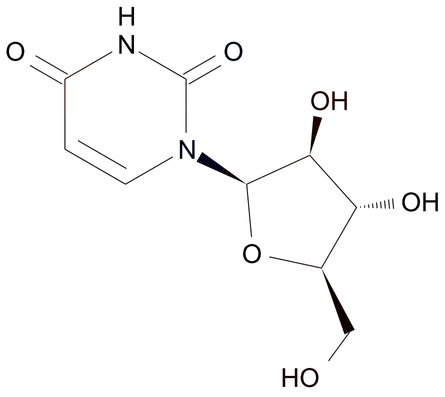

$C_9H_{12}N_2O_6$
Molecular Weight: 244.20137
ALogP: -2.451
Rotatable Bonds: 2
Acceptors: 6
Donors: 4

Model Prediction

Prediction: Moderate_Severe
Probability: 0.831
Enrichment: 1.206
Bayesian Score: 0.573
Mahalanobis Distance: 15.956
Mahalanobis Distance p-value: 4.12e-017

Prediction: Positive if the Bayesian score is above the estimated best cutoff value from minimizing the false positive and false negative rate.
Probability: The esimated probability that the sample is in the positive category. This assumes that the Bayesian score follows a normal distribution and is different from the prediction using a cutoff.
Enrichment: An estimate of enrichment, that is, the increased likelihood (versus random) of this sample being in the category.
Bayesian Score: The standard Laplacian-modified Bayesian score.
Mahalanobis Distance: The Mahalanobis distance (MD) is the distance to the center of the training data. The larger the MD, the less trustworthy the prediction.
Mahalanobis Distance p-value: The p-value gives the fraction of training data with an MD greater than or equal to the one for the given sample, assuming normally distributed data. The smaller the p-value, the less trustworthy the prediciton. For highly non-normal X properties (e.g., fingerprints), the MD p-value is wildly inaccurate.

Structural Similar Compounds

Name	TRIETHYLENETETRAMINE	ANTHRAQUINONE;1;5-DIAMINO-4;8-DIHYDROXY-	ANTHRAQUINONE; 1;8-DIAMINO-4;5-DIHYDROXY-
Structure	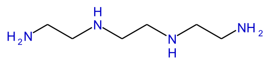	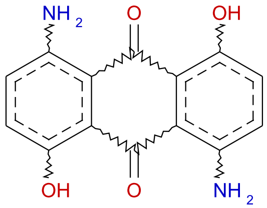	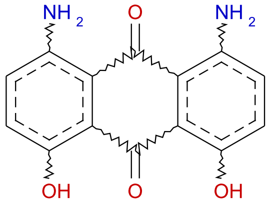
Actual Endpoint	Moderate_Severe	Moderate_Severe	Moderate_Severe
Predicted Endpoint	Moderate_Severe	Mild	Mild
Distance	0.794	0.815	0.816
Reference	UCDS**12/12/66	28ZPAK-;103;72	28ZPAK-;103;72

Model Applicability

Unknown features are fingerprint features in the query molecule, but not found in the training set.

1. All properties and OPS components are within expected ranges.

Feature Contribution

Top features for positive contribution

Fingerprint	Bit/Smiles	Feature Structure	Score	Moderate_Severe in training set
FCFP_10	-1272709286	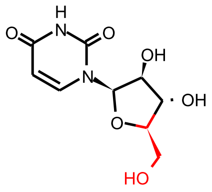 [*]C([*])CO	0.285	234 out of 266

FCFP_10	-885550502	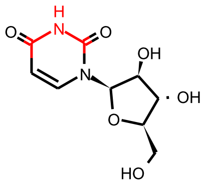<br>[*]C(=[*])NC(=[*])[*]	0.239	54 out of 64
FCFP_10	1070061035	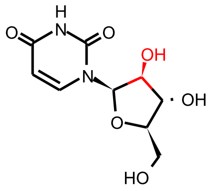<br>[*]C([*])O	0.239	284 out of 338
**Top Features for negative contribution**				
**Fingerprint**	**Bit/Smiles**	**Feature Structure**	**Score**	**Moderate_Severe in training set**
FCFP_10	-1986098826	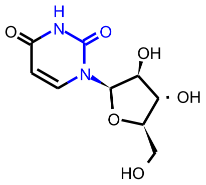<br>[*]NC(=O)N([*])[*]	-0.361	2 out of 5
FCFP_10	451877515	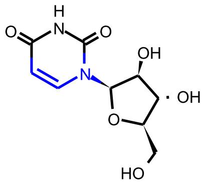<br>[*]N([*])C=C([*])[*]	-0.350	16 out of 35
FCFP_10	699500266	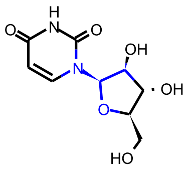<br>[*][C@H]1[*][*]O[C@H]<br>1N([*])[*]	-0.103	16 out of 27

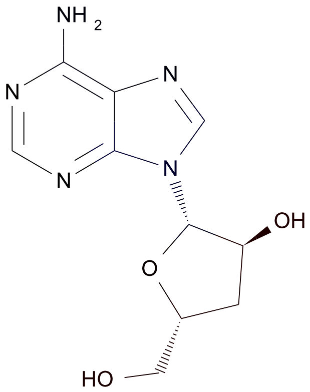

$C_{10}H_{13}N_5O_3$
Molecular Weight: 251.24191
ALogP: -1.308
Rotatable Bonds: 2
Acceptors: 7
Donors: 3

Structural Similar Compounds

Name	2-NAPHTHALENESULFONIC ACID; 6-AMINO-5-SULFOMETHYL-	1;5-NAPHTHALENEDISULFONIC ACID;2-AMINO-	1;5-NAPHTHALENEDISULFONIC ACID;3-AMINO-
Structure	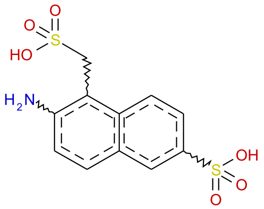	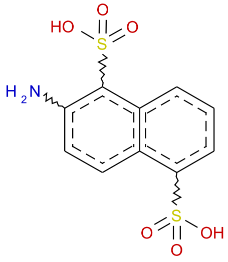	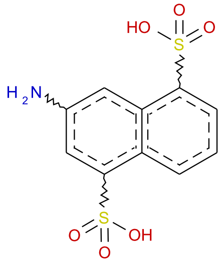
Actual Endpoint	Mild	Moderate_Severe	Moderate_Severe
Predicted Endpoint	Mild	Mild	Moderate_Severe
Distance	0.718	0.729	0.730
Reference	28ZPAK 190;72	28ZPAK-;188;72	28ZPAK-;189;72

Model Prediction

Prediction: Moderate_Severe
Probability: 0.820
Enrichment: 1.190
Bayesian Score: -0.093
Mahalanobis Distance: 9.811
Mahalanobis Distance p-value: 0.144

Prediction: Positive if the Bayesian score is above the estimated best cutoff value from minimizing the false positive and false negative rate.
Probability: The esimated probability that the sample is in the positive category. This assumes that the Bayesian score follows a normal distribution and is different from the prediction using a cutoff.
Enrichment: An estimate of enrichment, that is, the increased likelihood (versus random) of this sample being in the category.
Bayesian Score: The standard Laplacian-modified Bayesian score.
Mahalanobis Distance: The Mahalanobis distance (MD) is the distance to the center of the training data. The larger the MD, the less trustworthy the prediction.
Mahalanobis Distance p-value: The p-value gives the fraction of training data with an MD greater than or equal to the one for the given sample, assuming normally distributed data. The smaller the p-value, the less trustworthy the prediciton. For highly non-normal X properties (e.g., fingerprints), the MD p-value is wildly inaccurate.

Model Applicability

Unknown features are fingerprint features in the query molecule, but not found in the training set.

1. All properties and OPS components are within expected ranges.
2. Unknown FCFP_2 feature: -124685461: [*]:n:c:n:[*]
3. Unknown FCFP_2 feature: -1564473960: [*]n1:[*]:[*]:[c](:[*]):[c]:1:n:[*]
4. Unknown FCFP_2 feature: -1151884458: [*]:n:[c](N):[c](:[*]):[*]
5. Unknown FCFP_2 feature: 1315416442: [*][C@H]1[*][*]O[C@H]1n(:[*]):[*]

Feature Contribution

Top features for positive contribution				
**Fingerprint**	**Bit/Smiles**	**Feature Structure**	**Score**	**Moderate_Severe in training set**
FCFP_10	-1272709286	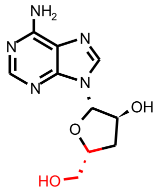 [*]C([*])CO	0.285	234 out of 266

FCFP_10	1070061035	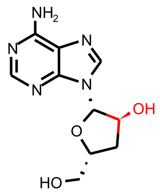 [*]C([*])O	0.239	284 out of 338
FCFP_10	-1043250487	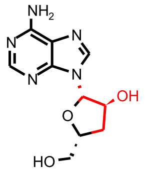 [*][C@@H]1[*][*][C@H]([*])[C@H]1O	0.220	62 out of 75

Top Features for negative contribution

Fingerprint	Bit/Smiles	Feature Structure	Score	Moderate_Severe in training set
FCFP_10	-306856457	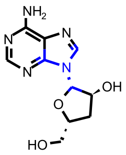 [*]C([*])n1:[cH]:[*]:[*]:[c]:1:[*]	-0.842	0 out of 2
FCFP_10	178336375	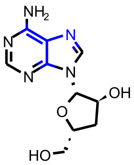 [*][c](:[*]):[c]1:n:[*]:[*]:[c]:1:[*]	-0.209	10 out of 19
FCFP_10	1069584379	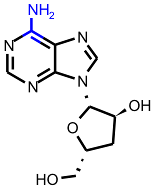 [*]:[c](:[*])N	-0.196	46 out of 85

Ribavirin

TOPKAT_Ocular_Irritancy_Mild_vs_Moderate_Severe

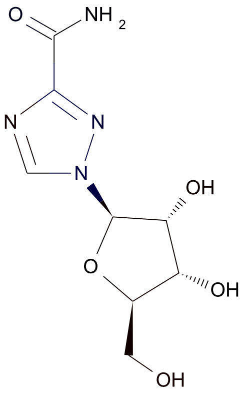

$C_8H_{12}N_4O_5$

Molecular Weight: 244.20467

ALogP: -2.745

Rotatable Bonds: 3

Acceptors: 7

Donors: 4

Structural Similar Compounds

Name	P-BENZENEDISULFONIC ACID;2-AMINO-	2;2'-Biphenyldisulfonic acid; 4;4'-diamino-	BENZENE-1;3-DICARBOXYLIC ACID;5-SULFO-
Structure	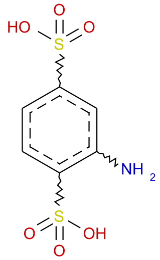	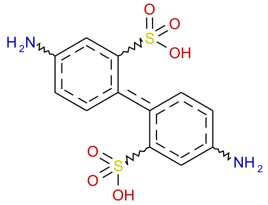	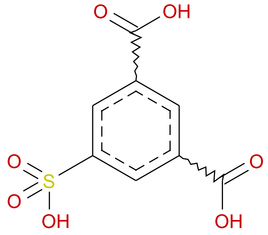
Actual Endpoint	Moderate_Severe	Mild	Moderate_Severe
Predicted Endpoint	Mild	Mild	Moderate_Severe
Distance	0.820	0.842	0.848
Reference	28ZPAK-;182;72	Prehled Prumyslove Toxikologie; Organicke Latky; Marhold; J. pp 1061;86	28ZPAK-;184;72

Model Prediction

Prediction: Mild

Probability: 0.807

Enrichment: 1.172

Bayesian Score: -0.711

Mahalanobis Distance: 10.957

Mahalanobis Distance p-value: 0.00555

Prediction: Positive if the Bayesian score is above the estimated best cutoff value from minimizing the false positive and false negative rate.
Probability: The esimated probability that the sample is in the positive category. This assumes that the Bayesian score follows a normal distribution and is different from the prediction using a cutoff.
Enrichment: An estimate of enrichment, that is, the increased likelihood (versus random) of this sample being in the category.
Bayesian Score: The standard Laplacian-modified Bayesian score.
Mahalanobis Distance: The Mahalanobis distance (MD) is the distance to the center of the training data. The larger the MD, the less trustworthy the prediction.
Mahalanobis Distance p-value: The p-value gives the fraction of training data with an MD greater than or equal to the one for the given sample, assuming normally distributed data. The smaller the p-value, the less trustworthy the prediciton. For highly non-normal X properties (e.g., fingerprints), the MD p-value is wildly inaccurate.

Model Applicability

Unknown features are fingerprint features in the query molecule, but not found in the training set.

1. All properties and OPS components are within expected ranges.
2. Unknown FCFP_2 feature: -2049666792: [*]C([*])n1:n:[*]:[*]:c:1
3. Unknown FCFP_2 feature: -124685461: [*]:n:c:n:[*]
4. Unknown FCFP_2 feature: 1315416442: [*][C@H]1[*][*]O[C@H]1n(:[*]):[*]

Feature Contribution

Top features for positive contribution

Fingerprint	Bit/Smiles	Feature Structure	Score	Moderate_Severe in training set
FCFP_10	-1539162406	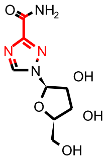 [*]C(=[*])[c]1:n:[*]:[*]:n:1	0.294	3 out of 3

FCFP_10	-1272709286	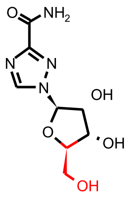 [*]C([*])CO	0.285	234 out of 266
FCFP_10	1070061035	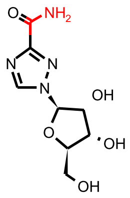 [*]C([*])O	0.239	284 out of 338

Top Features for negative contribution

Fingerprint	Bit/Smiles	Feature Structure	Score	Moderate_Severe in training set
FCFP_10	4427049	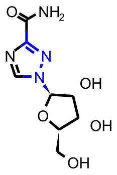 [*]n1:[*]:[*]:[c]([*]):n:1	-1.293	0 out of 4
FCFP_10	-1549103449	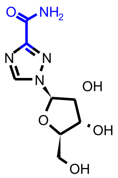 [*]:[c](:[*])C(=O)N	-0.504	2 out of 6
FCFP_10	1872154524	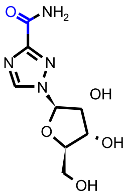 [*]C(=O)[*]	-0.078	344 out of 563

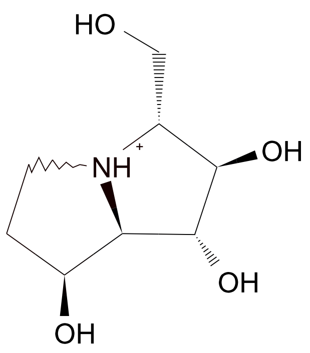

$C_8H_{16}NO_4$
Molecular Weight: 190.21693
ALogP: -3.556
Rotatable Bonds: 1
Acceptors: 4
Donors: 5

Structural Similar Compounds

Name	1;2-Ethanediamine; N-(2-aminoethyl)-N'-(2-((2-aminoethyl)amino)ethyl)-	TRIETHYLENETETRAMINE	ETHANOL; 2-((2-AMINOETHYL)AMINO)-
Structure	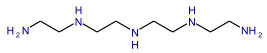	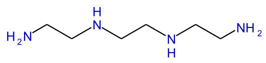	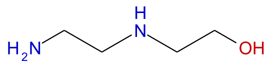
Actual Endpoint	Moderate	Moderate	Severe
Predicted Endpoint	Moderate	Moderate	Severe
Distance	0.801	0.829	1.004
Reference	Prehled Prumyslove Toxikologie; Organicke Latky; Marhold; J. -;446;86	UCDS**12/12/66	UCDS** 7/19/65

Model Prediction

Prediction: Severe
Probability: 0.693
Enrichment: 1.117
Bayesian Score: 0.037
Mahalanobis Distance: 10.310
Mahalanobis Distance p-value: 0.0173

Prediction: Positive if the Bayesian score is above the estimated best cutoff value from minimizing the false positive and false negative rate.
Probability: The esimated probability that the sample is in the positive category. This assumes that the Bayesian score follows a normal distribution and is different from the prediction using a cutoff.
Enrichment: An estimate of enrichment, that is, the increased likelihood (versus random) of this sample being in the category.
Bayesian Score: The standard Laplacian-modified Bayesian score.
Mahalanobis Distance: The Mahalanobis distance (MD) is the distance to the center of the training data. The larger the MD, the less trustworthy the prediction.
Mahalanobis Distance p-value: The p-value gives the fraction of training data with an MD greater than or equal to the one for the given sample, assuming normally distributed data. The smaller the p-value, the less trustworthy the prediciton. For highly non-normal X properties (e.g., fingerprints), the MD p-value is wildly inaccurate.

Model Applicability

Unknown features are fingerprint features in the query molecule, but not found in the training set.

1. All properties and OPS components are within expected ranges.

Feature Contribution

Top features for positive contribution				
**Fingerprint**	**Bit/Smiles**	**Feature Structure**	**Score**	**Severe in training set**
SCFP_12	11	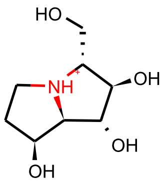 [*][NH+]([*])[*]	0.218	1 out of 1

SCFP_12	-1396915742	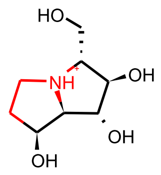 [*][NH+]1[*][*]CC1	0.218	1 out of 1
SCFP_12	-1272798659	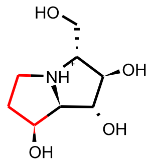 [*][C@@H]1[*][*]CC1	0.139	216 out of 309

Top Features for negative contribution

Fingerprint	Bit/Smiles	Feature Structure	Score	Severe in training set
SCFP_12	1702634808	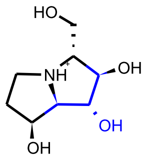 [*][C@@H]1[*][*][C@H]([*])[C@H]1O	-0.072	48 out of 85
SCFP_12	0	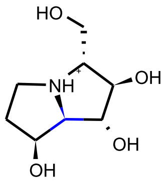 [*]C([*])[*]	0.000	463 out of 727
SCFP_12	-711686199	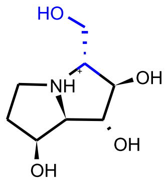 [*]C([*])CO	0.000	173 out of 293

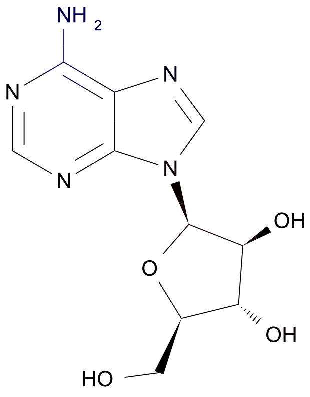

$C_{10}H_{13}N_5O_4$
Molecular Weight: 267.24132
ALogP: -1.881
Rotatable Bonds: 2
Acceptors: 8
Donors: 4

Model Prediction

Prediction: Moderate
Probability: 0.590
Enrichment: 0.952
Bayesian Score: -2.452
Mahalanobis Distance: 9.737
Mahalanobis Distance p-value: 0.0828

Prediction: Positive if the Bayesian score is above the estimated best cutoff value from minimizing the false positive and false negative rate.
Probability: The esimated probability that the sample is in the positive category. This assumes that the Bayesian score follows a normal distribution and is different from the prediction using a cutoff.
Enrichment: An estimate of enrichment, that is, the increased likelihood (versus random) of this sample being in the category.
Bayesian Score: The standard Laplacian-modified Bayesian score.
Mahalanobis Distance: The Mahalanobis distance (MD) is the distance to the center of the training data. The larger the MD, the less trustworthy the prediction.
Mahalanobis Distance p-value: The p-value gives the fraction of training data with an MD greater than or equal to the one for the given sample, assuming normally distributed data. The smaller the p-value, the less trustworthy the prediciton. For highly non-normal X properties (e.g., fingerprints), the MD p-value is wildly inaccurate.

Structural Similar Compounds

Name	ANTHRAQUINONE;1;5-DIAMINO-4;8-DIHYDROXY-	ANTHRAQUINONE; 1;8-DIAMINO-4;5-DIHYDROXY-	1;5-NAPHTHALENEDISULFONIC ACID;2-AMINO-
Structure	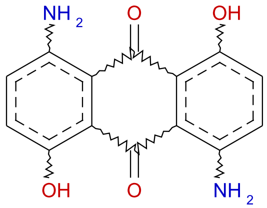	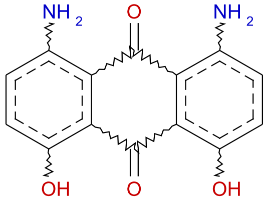	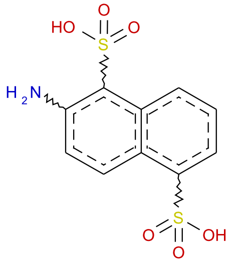
Actual Endpoint	Moderate	Moderate	Moderate
Predicted Endpoint	Moderate	Moderate	Moderate
Distance	0.826	0.827	0.860
Reference	28ZPAK-;103;72	28ZPAK-;103;72	28ZPAK-;188;72

Model Applicability

Unknown features are fingerprint features in the query molecule, but not found in the training set.

1. All properties and OPS components are within expected ranges.

Feature Contribution

Top features for positive contribution				
**Fingerprint**	**Bit/Smiles**	**Feature Structure**	**Score**	**Severe in training set**
SCFP_12	-443505090	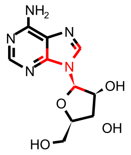 [*]C([*])n1:[cH]:[*]:[*]:[c]:1:[*]	0.218	1 out of 1

SCFP_12	-1715619483	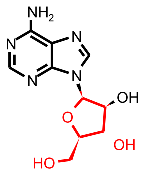 [*][C@@H]1O[C@H](CO)[C@@H](O)[C@@H]1[*]	0.218	1 out of 1
SCFP_12	-424515134	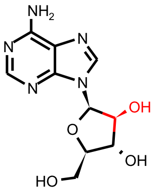 [*]C([*])O	0.099	141 out of 210
**Top Features for negative contribution**				
**Fingerprint**	**Bit/Smiles**	**Feature Structure**	**Score**	**Severe in training set**
SCFP_12	384920865	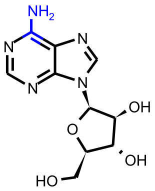 [*]:[c](:[*])N	-0.968	10 out of 46
SCFP_12	10	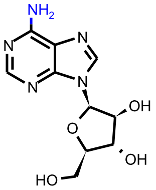 [*]N	-0.451	43 out of 112
SCFP_12	112554633	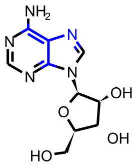 [*][c](:[*]):[c]1:n:[*]:[*]:[c]:1:[*]	-0.324	5 out of 12

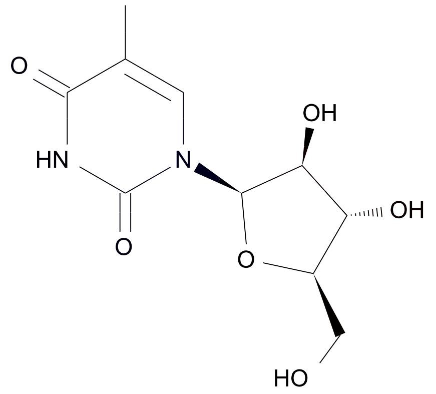

$C_{10}H_{14}N_2O_6$
Molecular Weight: 258.22795
ALogP: -2.005
Rotatable Bonds: 2
Acceptors: 6
Donors: 4

Model Prediction

Prediction: Moderate
Probability: 0.622
Enrichment: 1.004
Bayesian Score: -1.775
Mahalanobis Distance: 12.021
Mahalanobis Distance p-value: 1.5e-005

Prediction: Positive if the Bayesian score is above the estimated best cutoff value from minimizing the false positive and false negative rate.
Probability: The esimated probability that the sample is in the positive category. This assumes that the Bayesian score follows a normal distribution and is different from the prediction using a cutoff.
Enrichment: An estimate of enrichment, that is, the increased likelihood (versus random) of this sample being in the category.
Bayesian Score: The standard Laplacian-modified Bayesian score.
Mahalanobis Distance: The Mahalanobis distance (MD) is the distance to the center of the training data. The larger the MD, the less trustworthy the prediction.
Mahalanobis Distance p-value: The p-value gives the fraction of training data with an MD greater than or equal to the one for the given sample, assuming normally distributed data. The smaller the p-value, the less trustworthy the prediciton. For highly non-normal X properties (e.g., fingerprints), the MD p-value is wildly inaccurate.

Structural Similar Compounds

Name	ANTHRAQUINONE;1;5-DIAMINO-4;8-DIHYDROXY-	ANTHRAQUINONE; 1;8-DIAMINO-4;5-DIHYDROXY-	TRIETHYLENETETRAMINE
Structure	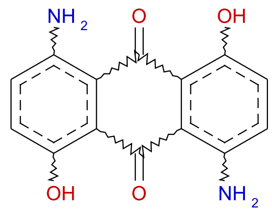	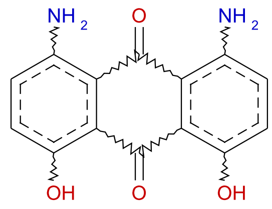	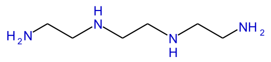
Actual Endpoint	Moderate	Moderate	Moderate
Predicted Endpoint	Moderate	Moderate	Moderate
Distance	0.785	0.786	0.813
Reference	28ZPAK-;103;72	28ZPAK-;103;72	UCDS**12/12/66

Model Applicability

Unknown features are fingerprint features in the query molecule, but not found in the training set.

1. All properties and OPS components are within expected ranges.

Feature Contribution

Top features for positive contribution

Fingerprint	Bit/Smiles	Feature Structure	Score	Severe in training set
SCFP_12	-1715619483	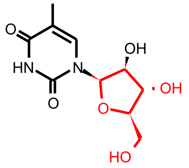 [*][C@@H]1O[C@H](CO)[C@@H](O)[C@@H]1[*]	0.218	1 out of 1

SCFP_12	1631785938	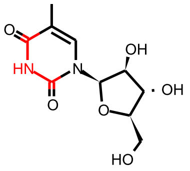<br>[*]C(=[*])NC(=[*])[*]	0.218	1 out of 1
SCFP_12	-1971435055	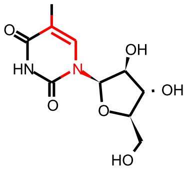<br>[*]N([*])C=C([*])[*]	0.153	3 out of 4
**Top Features for negative contribution**				
**Fingerprint**	**Bit/Smiles**	**Feature Structure**	**Score**	**Severe in training set**
SCFP_12	9	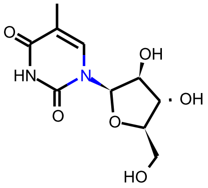<br>[*]N([*])[*]	-0.315	29 out of 66
SCFP_12	399659969	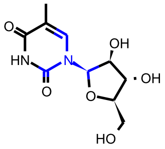<br>[*]C([*])N(C=[*])C(=[*])[*]	-0.298	2 out of 5
SCFP_12	1257024795	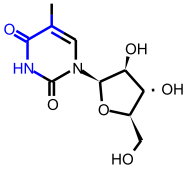<br>[*]NC(=O)C(=[*])[*]	-0.248	5 out of 11

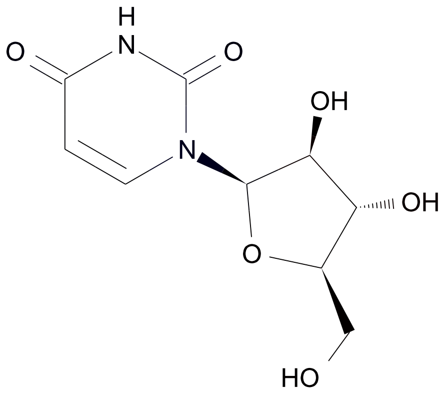

$C_9H_{12}N_2O_6$
Molecular Weight: 244.20137
ALogP: -2.451
Rotatable Bonds: 2
Acceptors: 6
Donors: 4

Model Prediction

Prediction: Moderate
Probability: 0.625
Enrichment: 1.009
Bayesian Score: -1.707
Mahalanobis Distance: 11.729
Mahalanobis Distance p-value: 6.38e-005

Prediction: Positive if the Bayesian score is above the estimated best cutoff value from minimizing the false positive and false negative rate.
Probability: The esimated probability that the sample is in the positive category. This assumes that the Bayesian score follows a normal distribution and is different from the prediction using a cutoff.
Enrichment: An estimate of enrichment, that is, the increased likelihood (versus random) of this sample being in the category.
Bayesian Score: The standard Laplacian-modified Bayesian score.
Mahalanobis Distance: The Mahalanobis distance (MD) is the distance to the center of the training data. The larger the MD, the less trustworthy the prediction.
Mahalanobis Distance p-value: The p-value gives the fraction of training data with an MD greater than or equal to the one for the given sample, assuming normally distributed data. The smaller the p-value, the less trustworthy the prediciton. For highly non-normal X properties (e.g., fingerprints), the MD p-value is wildly inaccurate.

Structural Similar Compounds

Name	TRIETHYLENETETRAMINE	ANTHRAQUINONE;1;5-DIAMINO-4;8-DIHYDROXY-	ANTHRAQUINONE; 1;8-DIAMINO-4;5-DIHYDROXY-
Structure	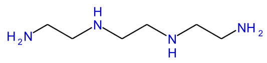	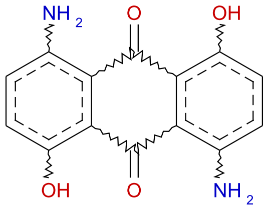	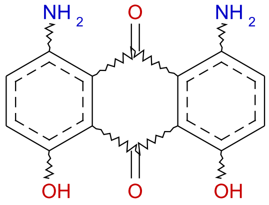
Actual Endpoint	Moderate	Moderate	Moderate
Predicted Endpoint	Moderate	Moderate	Moderate
Distance	0.830	0.833	0.833
Reference	UCDS**12/12/66	28ZPAK-;103;72	28ZPAK-;103;72

Model Applicability

Unknown features are fingerprint features in the query molecule, but not found in the training set.

1. All properties and OPS components are within expected ranges.

Feature Contribution

Top features for positive contribution

Fingerprint	Bit/Smiles	Feature Structure	Score	Severe in training set
SCFP_12	-1715619483	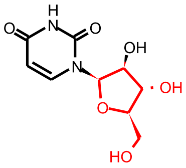 [*][C@@H]1O[C@H](CO)[C@@H](O)[C@@H]1[*]	0.218	1 out of 1

SCFP_12	1631785938	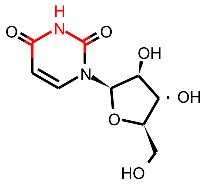 [*]C(=[*])NC(=[*])[*]	0.218	1 out of 1
SCFP_12	-1971435055	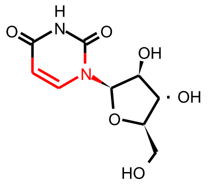 [*]N([*])C=C([*])[*]	0.153	3 out of 4

Top Features for negative contribution

Fingerprint	Bit/Smiles	Feature Structure	Score	Severe in training set
SCFP_12	9	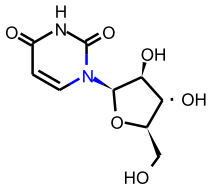 [*]N([*])[*]	-0.315	29 out of 66
SCFP_12	399659969	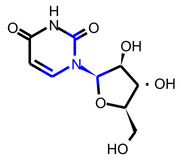 [*]C([*])N(C=[*])C(=[*])[*]	-0.298	2 out of 5
SCFP_12	1257024795	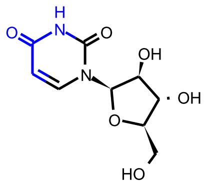 [*]NC(=O)C(=[*])[*]	-0.248	5 out of 11

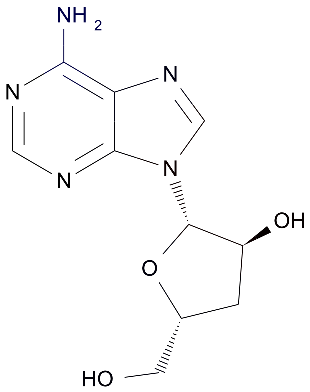

$C_{10}H_{13}N_5O_3$
Molecular Weight: 251.24191
ALogP: -1.308
Rotatable Bonds: 2
Acceptors: 7
Donors: 3

Model Prediction

Prediction: Moderate
Probability: 0.586
Enrichment: 0.946
Bayesian Score: -2.532
Mahalanobis Distance: 9.549
Mahalanobis Distance p-value: 0.126

Prediction: Positive if the Bayesian score is above the estimated best cutoff value from minimizing the false positive and false negative rate.
Probability: The esimated probability that the sample is in the positive category. This assumes that the Bayesian score follows a normal distribution and is different from the prediction using a cutoff.
Enrichment: An estimate of enrichment, that is, the increased likelihood (versus random) of this sample being in the category.
Bayesian Score: The standard Laplacian-modified Bayesian score.
Mahalanobis Distance: The Mahalanobis distance (MD) is the distance to the center of the training data. The larger the MD, the less trustworthy the prediction.
Mahalanobis Distance p-value: The p-value gives the fraction of training data with an MD greater than or equal to the one for the given sample, assuming normally distributed data. The smaller the p-value, the less trustworthy the prediciton. For highly non-normal X properties (e.g., fingerprints), the MD p-value is wildly inaccurate.

Structural Similar Compounds

Name	1;5-NAPHTHALENEDISULFONIC ACID;2-AMINO-	1;5-NAPHTHALENEDISULFONIC ACID;3-AMINO-	BENZENE-1;3-DICARBOXYLIC ACID;5-SULFO-
Structure	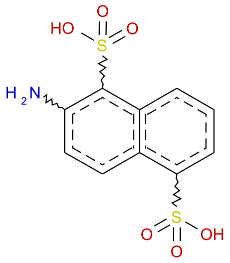	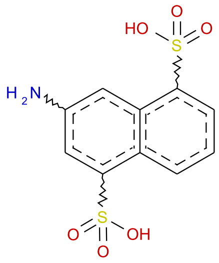	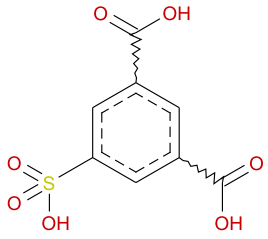
Actual Endpoint	Moderate	Moderate	Severe
Predicted Endpoint	Moderate	Moderate	Moderate
Distance	0.752	0.752	0.771
Reference	28ZPAK-;188;72	28ZPAK-;189;72	28ZPAK-;184;72

Model Applicability

Unknown features are fingerprint features in the query molecule, but not found in the training set.

1. All properties and OPS components are within expected ranges.

Feature Contribution

Top features for positive contribution

Fingerprint	Bit/Smiles	Feature Structure	Score	Severe in training set
SCFP_12	-443505090	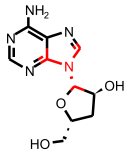 [*]C([*])n1:[cH]:[*]:[*]:[c]:1:[*]	0.218	1 out of 1

SCFP_12	-1272798659	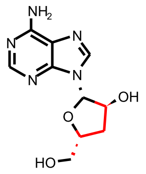 [*][C@@H]1[*][*]CC1	0.139	216 out of 309
SCFP_12	-424515134	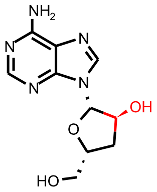 [*]C([*])O	0.099	141 out of 210

Top Features for negative contribution

Fingerprint	Bit/Smiles	Feature Structure	Score	Severe in training set
SCFP_12	384920865	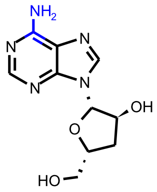 [*]:[c](:[*])N	-0.968	10 out of 46
SCFP_12	10	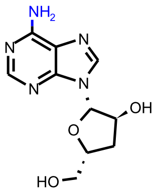 [*]N	-0.451	43 out of 112
SCFP_12	112554633	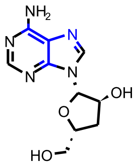 [*][c](:[*]):[c]1:n:[*]:[*]:[c]:1:[*]	-0.324	5 out of 12

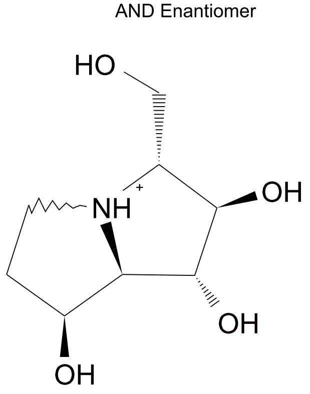

$C_8H_{16}NO_4$
Molecular Weight: 190.21693
ALogP: -3.556
Rotatable Bonds: 1
Acceptors: 4
Donors: 5

Structural Similar Compounds

Name	1;2-Ethanediamine; N-(2-aminoethyl)-N'-(2-((2-aminoethyl)amino)ethyl)-	TRIETHYLENETETRAMINE	Ethanol; 2;2';2'';2'''-silanetetrayltetrakis-
Structure	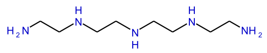	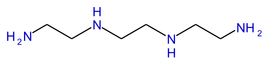	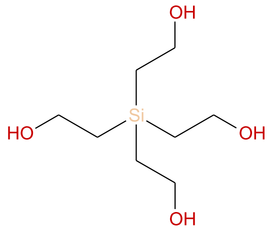
Actual Endpoint	Irritant	Irritant	Non-Irritant
Predicted Endpoint	Irritant	Irritant	Non-Irritant
Distance	0.755	0.771	0.874
Reference	Prehled Prumyslove Toxikologie; Organicke Latky; Marhold; J. -;446;86	UCDS**12/12/66	Prehled Prumyslove Toxikologie; Organicke Latky; Marhold; J. -;1232;86

Model Prediction

Prediction: Irritant

Probability: 0.976

Enrichment: 1.147

Bayesian Score: 0.277

Mahalanobis Distance: 10.118

Mahalanobis Distance p-value: 0.0704

Prediction: Positive if the Bayesian score is above the estimated best cutoff value from minimizing the false positive and false negative rate.
Probability: The esimated probability that the sample is in the positive category. This assumes that the Bayesian score follows a normal distribution and is different from the prediction using a cutoff.
Enrichment: An estimate of enrichment, that is, the increased likelihood (versus random) of this sample being in the category.
Bayesian Score: The standard Laplacian-modified Bayesian score.
Mahalanobis Distance: The Mahalanobis distance (MD) is the distance to the center of the training data. The larger the MD, the less trustworthy the prediction.
Mahalanobis Distance p-value: The p-value gives the fraction of training data with an MD greater than or equal to the one for the given sample, assuming normally distributed data. The smaller the p-value, the less trustworthy the prediciton. For highly non-normal X properties (e.g., fingerprints), the MD p-value is wildly inaccurate.

Model Applicability

Unknown features are fingerprint features in the query molecule, but not found in the training set.

1. All properties and OPS components are within expected ranges.
2. Unknown FCFP_2 feature: 10: [*][NH+]([*])[*]
3. Unknown FCFP_2 feature: -1853714334: [*][C@@H]1[*][*][C@H]2[*][*]C[NH+]12
4. Unknown FCFP_2 feature: -1817836174: [*][C@H]1[*][*][NH+]2[*][*][C@H]([*])C12
5. Unknown FCFP_2 feature: 1155241219: [*][NH+]1[*][*]CC1

Feature Contribution

Top features for positive contribution				
Fingerprint	Bit/Smiles	Feature Structure	Score	Irritant in training set

FCFP_12	1070061035	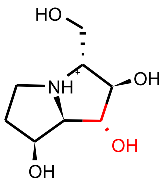 [*]C([*])O	0.107	338 out of 376
FCFP_12	3	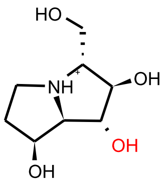 [*]O	0.105	491 out of 547
FCFP_12	-1272709286	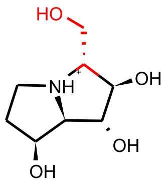 [*]C([*])CO	0.103	266 out of 297

Top Features for negative contribution

**Fingerprint**	**Bit/Smiles**	**Feature Structure**	**Score**	**Irritant in training set**
FCFP_12	0	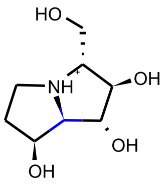 [*]C([*])[*]	0.000	1184 out of 1397
FCFP_12	-1272798659	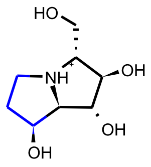 [*][C@@H]1[*][*]CC1	0.000	517 out of 643

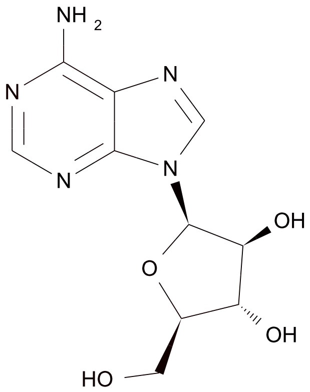

$C_{10}H_{13}N_5O_4$
Molecular Weight: 267.24132
ALogP: -1.881
Rotatable Bonds: 2
Acceptors: 8
Donors: 4

Structural Similar Compounds

Name	2;2'-Biphenyldisulfonic acid; 4;4'-diamino-	2;7-Naphthalenedisulfonic acid; 4-amino-5-hydroxy-	ANTHRAQUINONE;1;5-DIAMINO-4;8-DIHYDROXY-
Structure	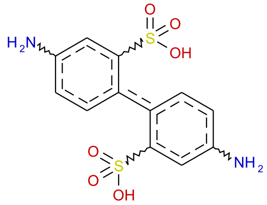	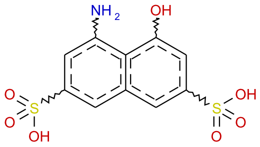	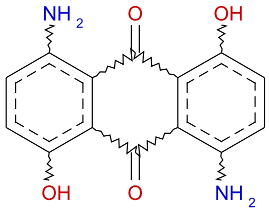
Actual Endpoint	Irritant	Non-Irritant	Irritant
Predicted Endpoint	Irritant	Non-Irritant	Irritant
Distance	0.718	0.729	0.777
Reference	Prehled Prumyslove Toxikologie; Organicke Latky; Marhold; J. pp 1061;86	Prehled Prumyslove Toxikologie; Organicke Latky; Marhold; J. pp 1058;86	28ZPAK-;103;72

Model Prediction

Prediction: Irritant
Probability: 1.000
Enrichment: 1.176
Bayesian Score: 1.388
Mahalanobis Distance: 9.789
Mahalanobis Distance p-value: 0.15

Prediction: Positive if the Bayesian score is above the estimated best cutoff value from minimizing the false positive and false negative rate.
Probability: The esimated probability that the sample is in the positive category. This assumes that the Bayesian score follows a normal distribution and is different from the prediction using a cutoff.
Enrichment: An estimate of enrichment, that is, the increased likelihood (versus random) of this sample being in the category.
Bayesian Score: The standard Laplacian-modified Bayesian score.
Mahalanobis Distance: The Mahalanobis distance (MD) is the distance to the center of the training data. The larger the MD, the less trustworthy the prediction.
Mahalanobis Distance p-value: The p-value gives the fraction of training data with an MD greater than or equal to the one for the given sample, assuming normally distributed data. The smaller the p-value, the less trustworthy the prediciton. For highly non-normal X properties (e.g., fingerprints), the MD p-value is wildly inaccurate.

Model Applicability

Unknown features are fingerprint features in the query molecule, but not found in the training set.

1. All properties and OPS components are within expected ranges.
2. Unknown FCFP_2 feature: -124685461: [*]:n:c:n:[*]
3. Unknown FCFP_2 feature: -1564473960: [*]n1:[*]:[*]:[c](:[*]):[c]:1:n:[*]
4. Unknown FCFP_2 feature: -1151884458: [*]:n:[c](N):[c](:[*]):[*]
5. Unknown FCFP_2 feature: 1315416442: [*][C@H]1[*][*]O[C@H]1n(:[*]):[*]

Feature Contribution

Top features for positive contribution				
**Fingerprint**	**Bit/Smiles**	**Feature Structure**	**Score**	**Irritant in training set**

FCFP_12	1747237384	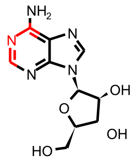 [*][c](:[*]):n:[cH]:[*]	0.208	44 out of 44
FCFP_12	178336375	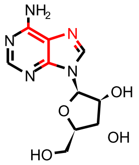 [*][c](:[*]):[c]1:n:[*]:[*]:[c]:1:[*]	0.202	19 out of 19
FCFP_12	17	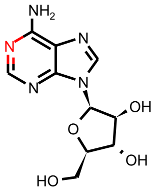 [*]:n:[*]	0.189	48 out of 49

Top Features for negative contribution

Fingerprint	Bit/Smiles	Feature Structure	Score	Irritant in training set
FCFP_12	-1143715940	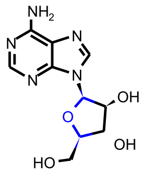 [*][C@@H]1[*][*][C@H] ([*])O1	0.000	454 out of 590
FCFP_12	1069584379	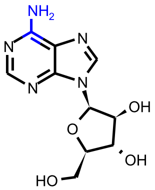 [*]:[c](:[*])N	0.000	85 out of 101

FCFP_12	1	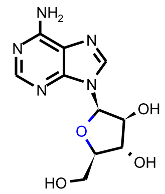 [*]O[*]	0.000	872 out of 1051

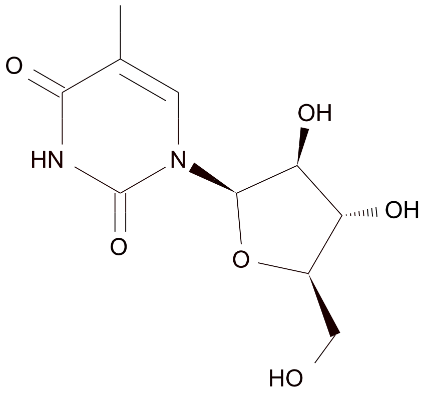

$C_{10}H_{14}N_2O_6$
Molecular Weight: 258.22795
ALogP: -2.005
Rotatable Bonds: 2
Acceptors: 6
Donors: 4

Model Prediction

Prediction: Irritant
Probability: 1.000
Enrichment: 1.176
Bayesian Score: 1.530
Mahalanobis Distance: 13.987
Mahalanobis Distance p-value: 2.54e-010

Prediction: Positive if the Bayesian score is above the estimated best cutoff value from minimizing the false positive and false negative rate.
Probability: The esimated probability that the sample is in the positive category. This assumes that the Bayesian score follows a normal distribution and is different from the prediction using a cutoff.
Enrichment: An estimate of enrichment, that is, the increased likelihood (versus random) of this sample being in the category.
Bayesian Score: The standard Laplacian-modified Bayesian score.
Mahalanobis Distance: The Mahalanobis distance (MD) is the distance to the center of the training data. The larger the MD, the less trustworthy the prediction.
Mahalanobis Distance p-value: The p-value gives the fraction of training data with an MD greater than or equal to the one for the given sample, assuming normally distributed data. The smaller the p-value, the less trustworthy the prediciton. For highly non-normal X properties (e.g., fingerprints), the MD p-value is wildly inaccurate.

Structural Similar Compounds

Name	ANTHRAQUINONE;1;5-DIAMINO-4;8-DIHYDROXY-	ANTHRAQUINONE; 1;8-DIAMINO-4;5-DIHYDROXY-	TRIETHYLENETETRAMINE
Structure	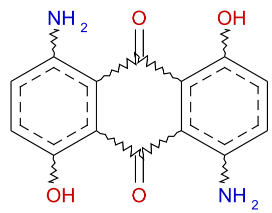	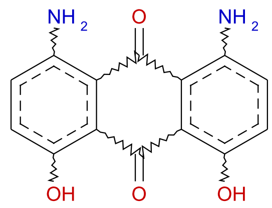	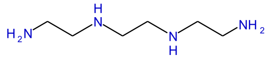
Actual Endpoint	Irritant	Irritant	Irritant
Predicted Endpoint	Irritant	Irritant	Irritant
Distance	0.733	0.733	0.765
Reference	28ZPAK-;103;72	28ZPAK-;103;72	UCDS**12/12/66

Model Applicability

Unknown features are fingerprint features in the query molecule, but not found in the training set.

1. All properties and OPS components are within expected ranges.

Feature Contribution

Top features for positive contribution

Fingerprint	Bit/Smiles	Feature Structure	Score	Irritant in training set
FCFP_12	-2091721556	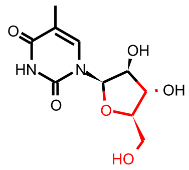 [*][C@H]1[*][*]O[C@@H]1CO	0.187	8 out of 8

FCFP_12	-885550502	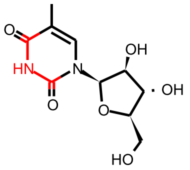 [*]C(=[*])NC(=[*])[*]	0.180	64 out of 66
FCFP_12	-1986098826	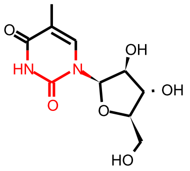 [*]NC(=O)N([*])[*]	0.175	5 out of 5

Top Features for negative contribution

Fingerprint	Bit/Smiles	Feature Structure	Score	Irritant in training set
FCFP_12	0	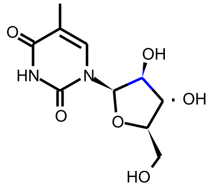 [*]C([*])[*]	0.000	1184 out of 1397
FCFP_12	-1143715940	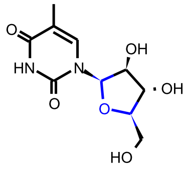 [*][C@@H]1[*][*][C@H]([*])O1	0.000	454 out of 590
FCFP_12	1872154524	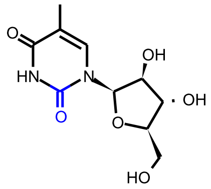 [*]C(=O)[*]	0.000	563 out of 690

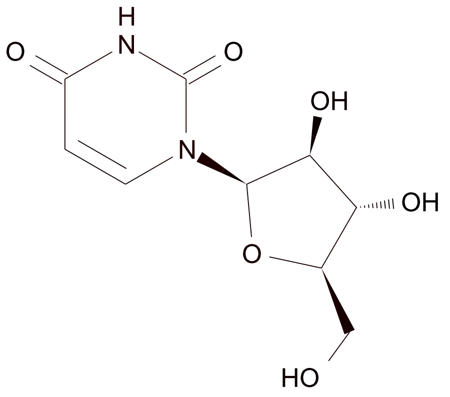

$C_9H_{12}N_2O_6$
Molecular Weight: 244.20137
ALogP: -2.451
Rotatable Bonds: 2
Acceptors: 6
Donors: 4

Model Prediction

Prediction: Irritant
Probability: 1.000
Enrichment: 1.176
Bayesian Score: 1.631
Mahalanobis Distance: 12.548
Mahalanobis Distance p-value: 3.04e-006

Prediction: Positive if the Bayesian score is above the estimated best cutoff value from minimizing the false positive and false negative rate.
Probability: The esimated probability that the sample is in the positive category. This assumes that the Bayesian score follows a normal distribution and is different from the prediction using a cutoff.
Enrichment: An estimate of enrichment, that is, the increased likelihood (versus random) of this sample being in the category.
Bayesian Score: The standard Laplacian-modified Bayesian score.
Mahalanobis Distance: The Mahalanobis distance (MD) is the distance to the center of the training data. The larger the MD, the less trustworthy the prediction.
Mahalanobis Distance p-value: The p-value gives the fraction of training data with an MD greater than or equal to the one for the given sample, assuming normally distributed data. The smaller the p-value, the less trustworthy the prediciton. For highly non-normal X properties (e.g., fingerprints), the MD p-value is wildly inaccurate.

Structural Similar Compounds

Name	ANTHRAQUINONE;1;5-DIAMINO-4;8-DIHYDROXY-	ANTHRAQUINONE; 1;8-DIAMINO-4;5-DIHYDROXY-	TRIETHYLENETETRAMINE
Structure	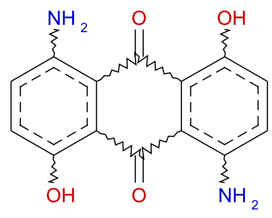	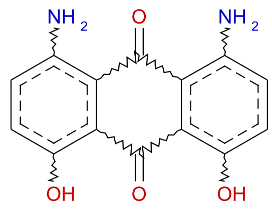	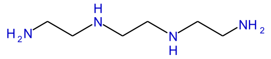
Actual Endpoint	Irritant	Irritant	Irritant
Predicted Endpoint	Irritant	Irritant	Irritant
Distance	0.772	0.773	0.781
Reference	28ZPAK-;103;72	28ZPAK-;103;72	UCDS**12/12/66

Model Applicability

Unknown features are fingerprint features in the query molecule, but not found in the training set.

1. All properties and OPS components are within expected ranges.

Feature Contribution

Top features for positive contribution				
**Fingerprint**	**Bit/Smiles**	**Feature Structure**	**Score**	**Irritant in training set**
FCFP_12	-2091721556	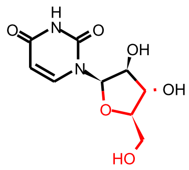 [*][C@H]1[*][*]O[C@@H]1CO	0.187	8 out of 8

FCFP_12	-885550502	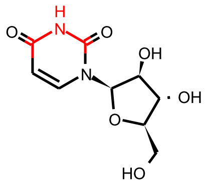 [*]C(=[*])NC(=[*])[*]	0.180	64 out of 66
FCFP_12	-1986098826	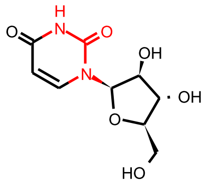 [*]NC(=O)N([*])[*]	0.175	5 out of 5
**Top Features for negative contribution**				
**Fingerprint**	**Bit/Smiles**	**Feature Structure**	**Score**	**Irritant in training set**
FCFP_12	1	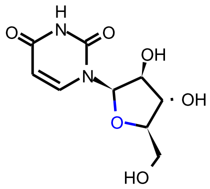 [*]O[*]	0.000	872 out of 1051
FCFP_12	0	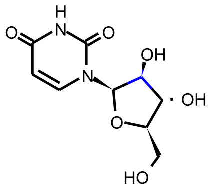 [*]C([*])[*]	0.000	1184 out of 1397
FCFP_12	-1143715940	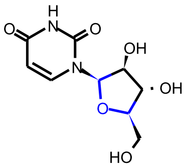 [*][C@@H]1[*][*][C@H]([*])O1	0.000	454 out of 590

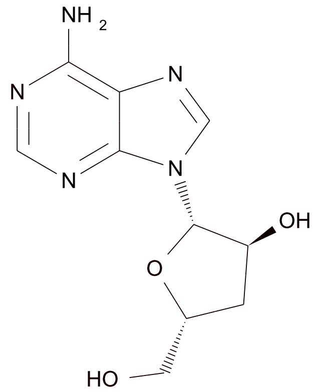

$C_{10}H_{13}N_5O_3$
Molecular Weight: 251.24191
ALogP: -1.308
Rotatable Bonds: 2
Acceptors: 7
Donors: 3

Structural Similar Compounds

Name	2-NAPHTHALENESULFONIC ACID; 6-AMINO-5-SULFOMETHYL-	HC Red #3	1;5-NAPHTHALENEDISULFONIC ACID;2-AMINO-
Structure	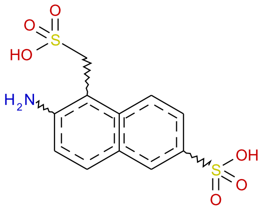	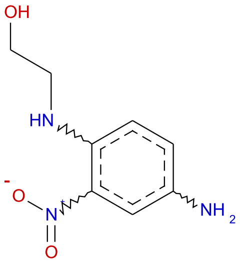	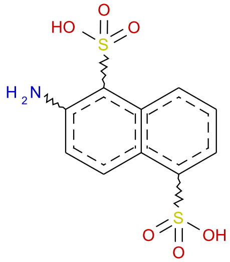
Actual Endpoint	Irritant	Non-Irritant	Irritant
Predicted Endpoint	Irritant	Non-Irritant	Irritant
Distance	0.695	0.705	0.710
Reference	28ZPAK 190;72	J. Am. Coll. Toxicol. 11(4):509;1992	28ZPAK-;188;72

Model Prediction

Prediction: Irritant
Probability: 1.000
Enrichment: 1.176
Bayesian Score: 1.663
Mahalanobis Distance: 9.406
Mahalanobis Distance p-value: 0.3

Prediction: Positive if the Bayesian score is above the estimated best cutoff value from minimizing the false positive and false negative rate.
Probability: The esimated probability that the sample is in the positive category. This assumes that the Bayesian score follows a normal distribution and is different from the prediction using a cutoff.
Enrichment: An estimate of enrichment, that is, the increased likelihood (versus random) of this sample being in the category.
Bayesian Score: The standard Laplacian-modified Bayesian score.
Mahalanobis Distance: The Mahalanobis distance (MD) is the distance to the center of the training data. The larger the MD, the less trustworthy the prediction.
Mahalanobis Distance p-value: The p-value gives the fraction of training data with an MD greater than or equal to the one for the given sample, assuming normally distributed data. The smaller the p-value, the less trustworthy the prediciton. For highly non-normal X properties (e.g., fingerprints), the MD p-value is wildly inaccurate.

Model Applicability

Unknown features are fingerprint features in the query molecule, but not found in the training set.

1. All properties and OPS components are within expected ranges.
2. Unknown FCFP_2 feature: -124685461: [*]:n:c:n:[*]
3. Unknown FCFP_2 feature: -1564473960: [*]n1:[*]:[*]:[c](:[*]):[c]:1:n:[*]
4. Unknown FCFP_2 feature: -1151884458: [*]:n:[c](N):[c](:[*]):[*]
5. Unknown FCFP_2 feature: 1315416442: [*][C@H]1[*][*]O[C@H]1n(:[*]):[*]

Feature Contribution

Top features for positive contribution				
Fingerprint	Bit/Smiles	Feature Structure	Score	Irritant in training set
FCFP_12	1747237384	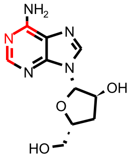 [*][c](:[*]):n:[cH]:[*]	0.208	44 out of 44

FCFP_12	178336375	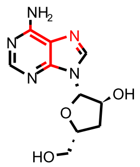 [*][c](:[*]):[c]1:n:[*]:[*]:[c]:1:[*]	0.202	19 out of 19
FCFP_12	17	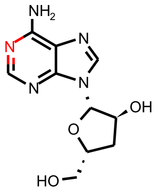 [*]:n:[*]	0.189	48 out of 49
**Top Features for negative contribution**				
**Fingerprint**	**Bit/Smiles**	**Feature Structure**	**Score**	**Irritant in training set**
FCFP_12	1069584379	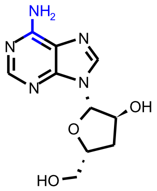 [*]:[c](:[*])N	0.000	85 out of 101
FCFP_12	0	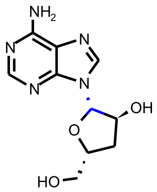 [*]C([*])[*]	0.000	1184 out of 1397
FCFP_12	1	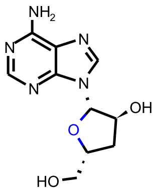 [*]O[*]	0.000	872 out of 1051

Ribavirin

TOPKAT_Ocular_Irritancy_None_vs_Irritant

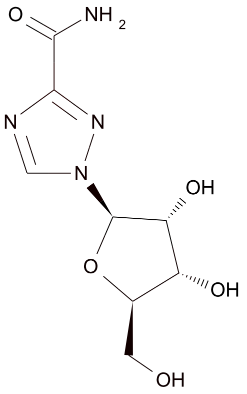

$C_8H_{12}N_4O_5$
Molecular Weight: 244.20467
ALogP: -2.745
Rotatable Bonds: 3
Acceptors: 7
Donors: 4

Structural Similar Compounds

Name	2;7-Naphthalenedisulfonic acid; 4-amino-5-hydroxy-	P-BENZENEDISULFONIC ACID;2-AMINO-	2;2'-Biphenyldisulfonic acid; 4;4'-diamino-
Structure	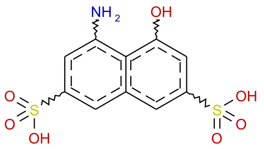	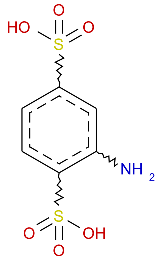	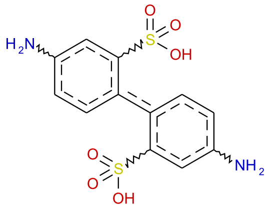
Actual Endpoint	Non-Irritant	Irritant	Irritant
Predicted Endpoint	Non-Irritant	Irritant	Irritant
Distance	0.786	0.799	0.801
Reference	Prehled Prumyslove Toxikologie; Organicke Latky; Marhold; J. pp 1058;86	28ZPAK-;182;72	Prehled Prumyslove Toxikologie; Organicke Latky; Marhold; J. pp 1061;86

Model Prediction

Prediction: Irritant

Probability: 1.000

Enrichment: 1.176

Bayesian Score: 1.535

Mahalanobis Distance: 9.704

Mahalanobis Distance p-value: 0.177

Prediction: Positive if the Bayesian score is above the estimated best cutoff value from minimizing the false positive and false negative rate.
Probability: The esimated probability that the sample is in the positive category. This assumes that the Bayesian score follows a normal distribution and is different from the prediction using a cutoff.
Enrichment: An estimate of enrichment, that is, the increased likelihood (versus random) of this sample being in the category.
Bayesian Score: The standard Laplacian-modified Bayesian score.
Mahalanobis Distance: The Mahalanobis distance (MD) is the distance to the center of the training data. The larger the MD, the less trustworthy the prediction.
Mahalanobis Distance p-value: The p-value gives the fraction of training data with an MD greater than or equal to the one for the given sample, assuming normally distributed data. The smaller the p-value, the less trustworthy the prediciton. For highly non-normal X properties (e.g., fingerprints), the MD p-value is wildly inaccurate.

Model Applicability

Unknown features are fingerprint features in the query molecule, but not found in the training set.

1. All properties and OPS components are within expected ranges.
2. Unknown FCFP_2 feature: -2049666792: [*]C([*])n1:n:[*]:[*]:c:1
3. Unknown FCFP_2 feature: -124685461: [*]:n:c:n:[*]
4. Unknown FCFP_2 feature: 1315416442: [*][C@H]1[*][*]O[C@H]1n(:[*]):[*]

Feature Contribution

Top features for positive contribution

Fingerprint	Bit/Smiles	Feature Structure	Score	Irritant in training set
FCFP_12	1747237384	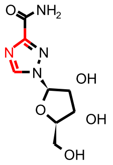 [*][c](:[*]):n:[cH]:[*]	0.208	44 out of 44

FCFP_12	17	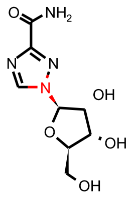 [*]:n:[*]	0.189	48 out of 49
FCFP_12	-2091721556	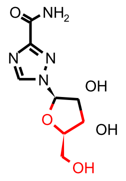 [*][C@H]1[*][*]O[C@@H]1CO	0.187	8 out of 8

Top Features for negative contribution

Fingerprint	Bit/Smiles	Feature Structure	Score	Irritant in training set
FCFP_12	-1143715940	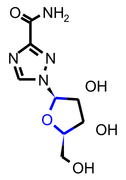 [*][C@@H]1[*][*][C@H] ([*])O1	0.000	454 out of 590
FCFP_12	1872154524	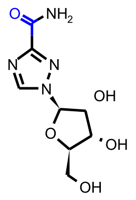 [*]C(=O)[*]	0.000	563 out of 690
FCFP_12	1	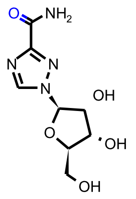 [*]O[*]	0.000	872 out of 1051

AND Enantiomer

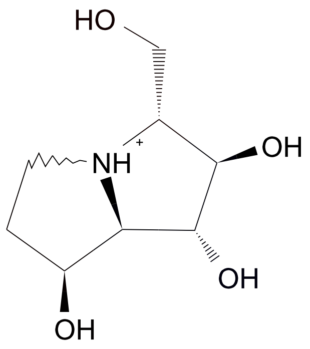

$C_8H_{16}NO_4$
Molecular Weight: 190.21693
ALogP: -3.556
Rotatable Bonds: 1
Acceptors: 4
Donors: 5

Model Prediction

Prediction: Carcinogen
Probability: 0.291
Enrichment: 0.904
Bayesian Score: -0.382
Mahalanobis Distance: 8.675
Mahalanobis Distance p-value: 0.909

Prediction: Positive if the Bayesian score is above the estimated best cutoff value from minimizing the false positive and false negative rate.
Probability: The esimated probability that the sample is in the positive category. This assumes that the Bayesian score follows a normal distribution and is different from the prediction using a cutoff.
Enrichment: An estimate of enrichment, that is, the increased likelihood (versus random) of this sample being in the category.
Bayesian Score: The standard Laplacian-modified Bayesian score.
Mahalanobis Distance: The Mahalanobis distance (MD) is the distance to the center of the training data. The larger the MD, the less trustworthy the prediction.
Mahalanobis Distance p-value: The p-value gives the fraction of training data with an MD greater than or equal to the one for the given sample, assuming normally distributed data. The smaller the p-value, the less trustworthy the prediciton. For highly non-normal X properties (e.g., fingerprints), the MD p-value is wildly inaccurate.

Structural Similar Compounds

Name	Methyldopa	Ribavirin	Ascorbic acid
Structure	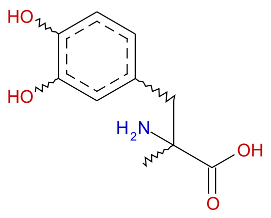	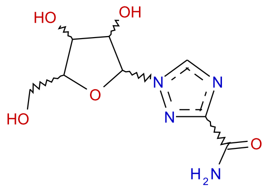	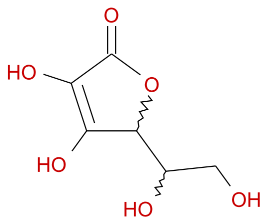
Actual Endpoint	Non-Carcinogen	Carcinogen	Non-Carcinogen
Predicted Endpoint	Non-Carcinogen	Carcinogen	Non-Carcinogen
Distance	0.755	0.845	0.855
Reference	US FDA (Centre for Drug Eval.& Res./Off. Testing & Res.) Sept. 1997	US FDA (Centre for Drug Eval.& Res./Off. Testing & Res.) Sept. 1997	US FDA (Centre for Drug Eval.& Res./Off. Testing & Res.) Sept. 1997

Model Applicability

Unknown features are fingerprint features in the query molecule, but not found in the training set.

1. All properties and OPS components are within expected ranges.
2. Unknown ECFP_2 feature: 1976330679: [*][NH+]([*])[*]
3. Unknown ECFP_2 feature: -1693770146: [*][C@@H]1[*][*][C@H]2[*][*]C[NH+]12
4. Unknown ECFP_2 feature: -1395316370: [*][C@H]1[*][*][NH+]2[*][*][C@H]([*])C12
5. Unknown ECFP_2 feature: -705668329: [*]C[C@@H]1[C@@H]([*])[*][*][NH+]1[*]
6. Unknown ECFP_2 feature: -244159614: [*][NH+]1[*][*]CC1

Feature Contribution

Top features for positive contribution

Fingerprint	Bit/Smiles	Feature Structure	Score	Carcinogen in training set
ECFP_12	-85480422	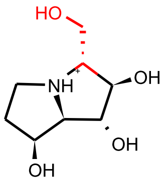 [*]C([*])CO	0.330	3 out of 6

ECFP_12	-1331450522	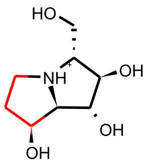 [*][C@@H]1[*][*]CC1	0.097	28 out of 81
ECFP_12	-329826665	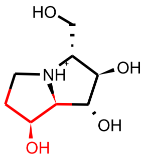 [*][C@@H]1[*][*]C[C@@H]1O	0.051	4 out of 12

Top Features for negative contribution

Fingerprint	Bit/Smiles	Feature Structure	Score	Carcinogen in training set
ECFP_12	305695353	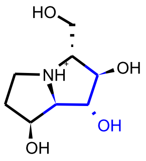 [*][C@@H]1[*][*][C@H]([*])[C@H]1O	-0.154	2 out of 8
ECFP_12	1559650422	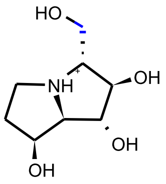 [*]C[*]	-0.112	63 out of 226
ECFP_12	-1884411803	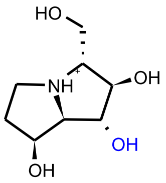 [*]O	-0.106	55 out of 196

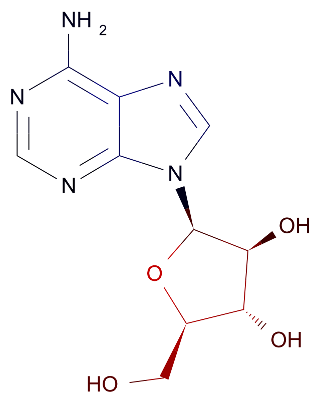

$C_{10}H_{13}N_5O_4$
Molecular Weight: 267.24132
ALogP: -1.881
Rotatable Bonds: 2
Acceptors: 8
Donors: 4

Model Prediction

Prediction: Carcinogen
Probability: 0.345
Enrichment: 1.072
Bayesian Score: 2.069
Mahalanobis Distance: 9.415
Mahalanobis Distance p-value: 0.649

Prediction: Positive if the Bayesian score is above the estimated best cutoff value from minimizing the false positive and false negative rate.
Probability: The esimated probability that the sample is in the positive category. This assumes that the Bayesian score follows a normal distribution and is different from the prediction using a cutoff.
Enrichment: An estimate of enrichment, that is, the increased likelihood (versus random) of this sample being in the category.
Bayesian Score: The standard Laplacian-modified Bayesian score.
Mahalanobis Distance: The Mahalanobis distance (MD) is the distance to the center of the training data. The larger the MD, the less trustworthy the prediction.
Mahalanobis Distance p-value: The p-value gives the fraction of training data with an MD greater than or equal to the one for the given sample, assuming normally distributed data. The smaller the p-value, the less trustworthy the prediciton. For highly non-normal X properties (e.g., fingerprints), the MD p-value is wildly inaccurate.

Structural Similar Compounds

Name	Ribavirin	Streptozocin	Acyclovir
Structure	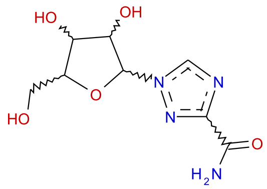	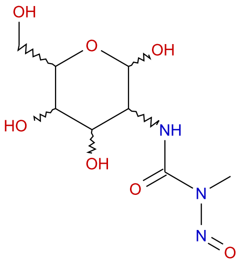	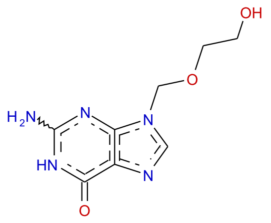
Actual Endpoint	Carcinogen	Carcinogen	Non-Carcinogen
Predicted Endpoint	Carcinogen	Carcinogen	Non-Carcinogen
Distance	0.526	0.603	0.684
Reference	US FDA (Centre for Drug Eval.& Res./Off. Testing & Res.) Sept. 1997	US FDA (Centre for Drug Eval.& Res./Off. Testing & Res.) Sept. 1997	US FDA (Centre for Drug Eval.& Res./Off. Testing & Res.) Sept. 1997

Model Applicability

Unknown features are fingerprint features in the query molecule, but not found in the training set.

1. All properties and OPS components are within expected ranges.

Feature Contribution

Top features for positive contribution

Fingerprint	Bit/Smiles	Feature Structure	Score	Carcinogen in training set
ECFP_12	-553149446	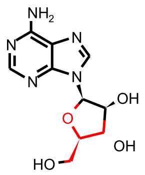 [*]C[C@H]1O[*][*][C@@H]1[*]	0.575	3 out of 4

ECFP_12	370123836	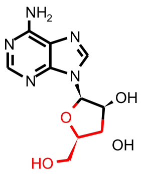 [*][C@H]1[*][*]O[C@@H]1CO	0.575	3 out of 4
ECFP_12	-763075316	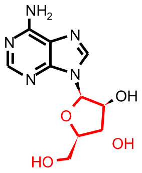 [*][C@@H]1O[C@H](CO)[C@@H](O)[C@@H]1[*]	0.437	2 out of 3

Top Features for negative contribution

Fingerprint	Bit/Smiles	Feature Structure	Score	Carcinogen in training set
ECFP_12	-1734834311	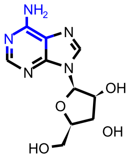 [*]:n:[c](N):[c](:[*]):[*]	-0.560	1 out of 8
ECFP_12	615197788	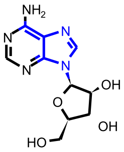 [*]n1:[cH]:n:[c](:[c]([*]):[*]):[c]:1:[*]	-0.485	0 out of 2
ECFP_12	-1219098860	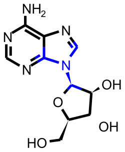 [*]C([*])n1:[cH]:[*]:[*]:[c]:1:[*]	-0.272	0 out of 1

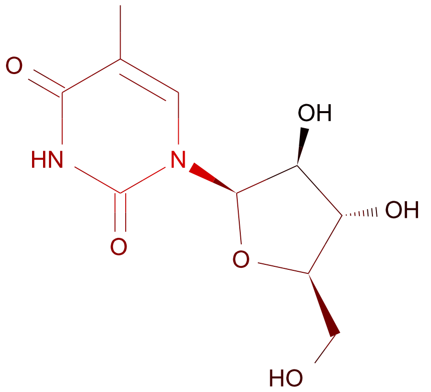

$C_{10}H_{14}N_2O_6$
Molecular Weight: 258.22795
ALogP: -2.005
Rotatable Bonds: 2
Acceptors: 6
Donors: 4

Model Prediction

Prediction: Carcinogen
Probability: 0.467
Enrichment: 1.451
Bayesian Score: 6.219
Mahalanobis Distance: 9.201
Mahalanobis Distance p-value: 0.743

Prediction: Positive if the Bayesian score is above the estimated best cutoff value from minimizing the false positive and false negative rate.
Probability: The esimated probability that the sample is in the positive category. This assumes that the Bayesian score follows a normal distribution and is different from the prediction using a cutoff.
Enrichment: An estimate of enrichment, that is, the increased likelihood (versus random) of this sample being in the category.
Bayesian Score: The standard Laplacian-modified Bayesian score.
Mahalanobis Distance: The Mahalanobis distance (MD) is the distance to the center of the training data. The larger the MD, the less trustworthy the prediction.
Mahalanobis Distance p-value: The p-value gives the fraction of training data with an MD greater than or equal to the one for the given sample, assuming normally distributed data. The smaller the p-value, the less trustworthy the prediciton. For highly non-normal X properties (e.g., fingerprints), the MD p-value is wildly inaccurate.

Structural Similar Compounds

Name	Zidovudine	Methyldopa	Ribavirin
Structure	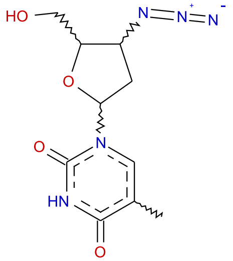	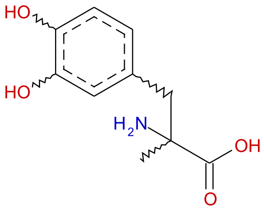	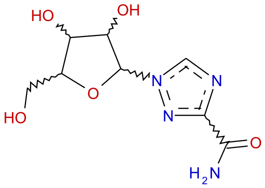
Actual Endpoint	Carcinogen	Non-Carcinogen	Carcinogen
Predicted Endpoint	Carcinogen	Non-Carcinogen	Carcinogen
Distance	0.566	0.605	0.617
Reference	US FDA (Centre for Drug Eval.& Res./Off. Testing & Res.) Sept. 1997	US FDA (Centre for Drug Eval.& Res./Off. Testing & Res.) Sept. 1997	US FDA (Centre for Drug Eval.& Res./Off. Testing & Res.) Sept. 1997

Model Applicability

Unknown features are fingerprint features in the query molecule, but not found in the training set.

1. All properties and OPS components are within expected ranges.
2. Unknown ECFP_2 feature: -627157165: [*][C@H]1[*][*]O[C@H]1N([*])[*]

Feature Contribution

Top features for positive contribution				
Fingerprint	Bit/Smiles	Feature Structure	Score	Carcinogen in training set
ECFP_12	1526392165	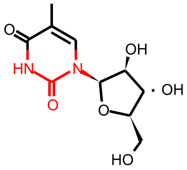 [*]NC(=O)N([*])[*]	0.851	5 out of 5

ECFP_12	370123836	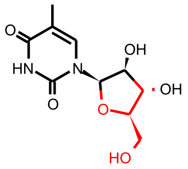 [*][C@H]1[*][*]O[C@@H]1CO	0.575	3 out of 4
ECFP_12	-553149446	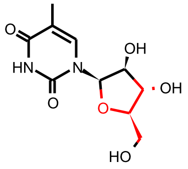 [*]C[C@H]1O[*][*][C@@ H]1[*]	0.575	3 out of 4

Top Features for negative contribution

**Fingerprint**	**Bit/Smiles**	**Feature Structure**	**Score**	**Carcinogen in training set**
ECFP_12	-3067141	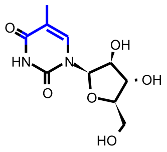 [*]\C=C(\C)/C(=[*])[*]	-0.248	1 out of 5
ECFP_12	-154530762	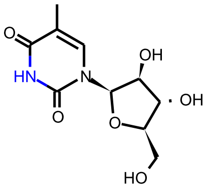 [*]N[*]	-0.203	9 out of 36
ECFP_12	305695353	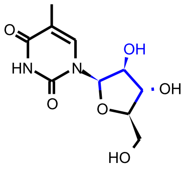 [*][C@@H]1[*][*][C@H] ([*])[C@H]1O	-0.154	2 out of 8

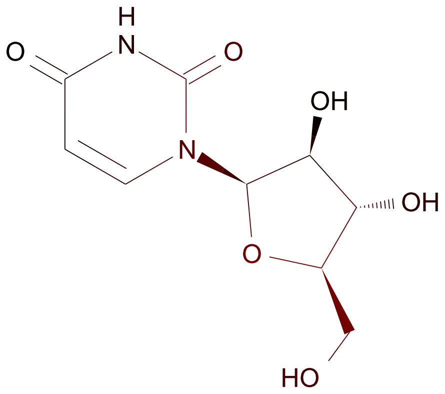

$C_9H_{12}N_2O_6$
Molecular Weight: 244.20137
ALogP: -2.451
Rotatable Bonds: 2
Acceptors: 6
Donors: 4

Model Prediction

Prediction: Carcinogen
Probability: 0.410
Enrichment: 1.274
Bayesian Score: 4.440
Mahalanobis Distance: 8.233
Mahalanobis Distance p-value: 0.973

Prediction: Positive if the Bayesian score is above the estimated best cutoff value from minimizing the false positive and false negative rate.
Probability: The esimated probability that the sample is in the positive category. This assumes that the Bayesian score follows a normal distribution and is different from the prediction using a cutoff.
Enrichment: An estimate of enrichment, that is, the increased likelihood (versus random) of this sample being in the category.
Bayesian Score: The standard Laplacian-modified Bayesian score.
Mahalanobis Distance: The Mahalanobis distance (MD) is the distance to the center of the training data. The larger the MD, the less trustworthy the prediction.
Mahalanobis Distance p-value: The p-value gives the fraction of training data with an MD greater than or equal to the one for the given sample, assuming normally distributed data. The smaller the p-value, the less trustworthy the prediciton. For highly non-normal X properties (e.g., fingerprints), the MD p-value is wildly inaccurate.

Structural Similar Compounds

Name	Ribavirin	Methyldopa	Ascorbic acid
Structure	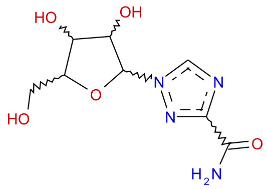	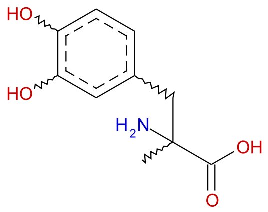	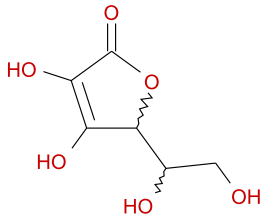
Actual Endpoint	Carcinogen	Non-Carcinogen	Non-Carcinogen
Predicted Endpoint	Carcinogen	Non-Carcinogen	Non-Carcinogen
Distance	0.557	0.633	0.664
Reference	US FDA (Centre for Drug Eval.& Res./Off. Testing & Res.) Sept. 1997	US FDA (Centre for Drug Eval.& Res./Off. Testing & Res.) Sept. 1997	US FDA (Centre for Drug Eval.& Res./Off. Testing & Res.) Sept. 1997

Model Applicability

Unknown features are fingerprint features in the query molecule, but not found in the training set.

1. All properties and OPS components are within expected ranges.
2. Unknown ECFP_2 feature: -627157165: [*][C@H]1[*][*]O[C@H]1N([*])[*]

Feature Contribution

Top features for positive contribution

Fingerprint	Bit/Smiles	Feature Structure	Score	Carcinogen in training set
ECFP_12	1526392165	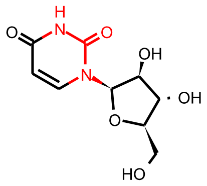 [*]NC(=O)N([*])[*]	0.851	5 out of 5

ECFP_12	-553149446	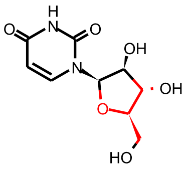 [*]C[C@H]1O[*][*][C@@H]1[*]	0.575	3 out of 4
ECFP_12	370123836	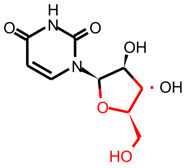 [*][C@H]1[*][*]O[C@@H]1CO	0.575	3 out of 4

Top Features for negative contribution

Fingerprint	Bit/Smiles	Feature Structure	Score	Carcinogen in training set
ECFP_12	-867415197	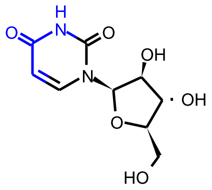 [*]NC(=O)C=[*]	-0.272	0 out of 1
ECFP_12	-154530762	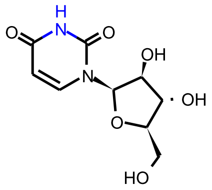 [*]N[*]	-0.203	9 out of 36
ECFP_12	305695353	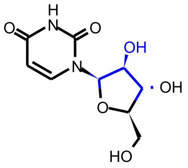 [*][C@@H]1[*][*][C@H]([*])[C@H]1O	-0.154	2 out of 8

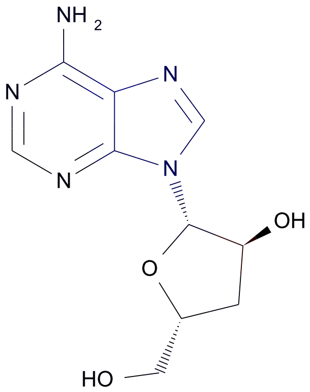

$C_{10}H_{13}N_5O_3$
Molecular Weight: 251.24191
ALogP: -1.308
Rotatable Bonds: 2
Acceptors: 7
Donors: 3

Model Prediction

Prediction: Non-Carcinogen
Probability: 0.256
Enrichment: 0.796
Bayesian Score: -2.359
Mahalanobis Distance: 9.986
Mahalanobis Distance p-value: 0.375

Prediction: Positive if the Bayesian score is above the estimated best cutoff value from minimizing the false positive and false negative rate.
Probability: The esimated probability that the sample is in the positive category. This assumes that the Bayesian score follows a normal distribution and is different from the prediction using a cutoff.
Enrichment: An estimate of enrichment, that is, the increased likelihood (versus random) of this sample being in the category.
Bayesian Score: The standard Laplacian-modified Bayesian score.
Mahalanobis Distance: The Mahalanobis distance (MD) is the distance to the center of the training data. The larger the MD, the less trustworthy the prediction.
Mahalanobis Distance p-value: The p-value gives the fraction of training data with an MD greater than or equal to the one for the given sample, assuming normally distributed data. The smaller the p-value, the less trustworthy the prediciton. For highly non-normal X properties (e.g., fingerprints), the MD p-value is wildly inaccurate.

Structural Similar Compounds

Name	Acyclovir	Didanosine	Zidovudine
Structure	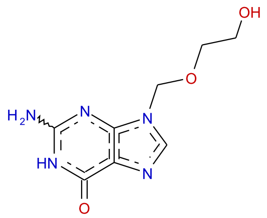	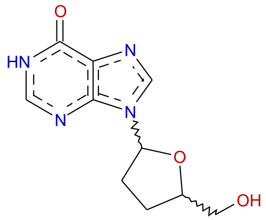	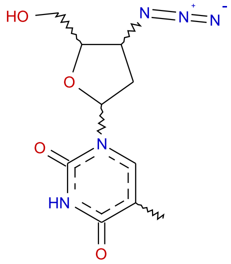
Actual Endpoint	Non-Carcinogen	Non-Carcinogen	Carcinogen
Predicted Endpoint	Non-Carcinogen	Non-Carcinogen	Carcinogen
Distance	0.580	0.595	0.617
Reference	US FDA (Centre for Drug Eval.& Res./Off. Testing & Res.) Sept. 1997	US FDA (Centre for Drug Eval.& Res./Off. Testing & Res.) Sept. 1997	US FDA (Centre for Drug Eval.& Res./Off. Testing & Res.) Sept. 1997

Model Applicability

Unknown features are fingerprint features in the query molecule, but not found in the training set.

1. All properties and OPS components are within expected ranges.

Feature Contribution

Top features for positive contribution				
**Fingerprint**	**Bit/Smiles**	**Feature Structure**	**Score**	**Carcinogen in training set**
ECFP_12	-1448786963	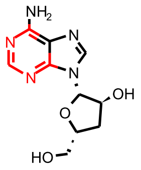 [*][c]1:[*]:[c](:[*]):n:[cH]:n:1	0.437	2 out of 3

ECFP_12	125442029	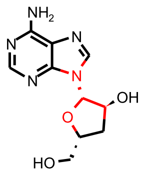 [*][C@H]1[*][*]O[C@H]1n(:[*]):[*]	0.421	1 out of 1
ECFP_12	-801490360	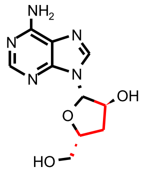 [*][C@@H]1[*][*][C@@H]([*])C1	0.339	14 out of 31

Top Features for negative contribution

**Fingerprint**	**Bit/Smiles**	**Feature Structure**	**Score**	**Carcinogen in training set**
ECFP_12	-1734834311	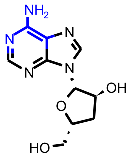 [*]:n:[c](N):[c](:[*]):[*]	-0.560	1 out of 8
ECFP_12	615197788	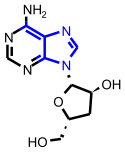 [*]n1:[cH]:n:[c](:[c]([*]):[*]):[c]:1:[*]	-0.485	0 out of 2
ECFP_12	376664744	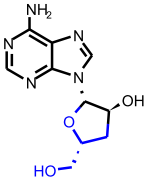 OC[C@H]1C[*][*]O1	-0.485	0 out of 2

Ribavirin

TOPKAT_Rat_Female_FDA_None_vs_Carcinogen

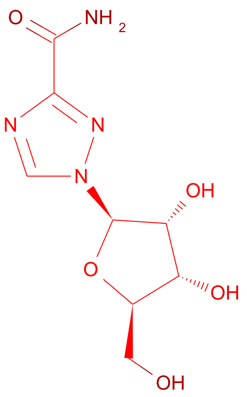

$C_8H_{12}N_4O_5$
Molecular Weight: 244.20467
ALogP: -2.745
Rotatable Bonds: 3
Acceptors: 7
Donors: 4

Model Prediction

Prediction: Carcinogen
Probability: 0.758
Enrichment: 2.353
Bayesian Score: 14.116
Mahalanobis Distance: 9.133
Mahalanobis Distance p-value: 0.77

Prediction: Positive if the Bayesian score is above the estimated best cutoff value from minimizing the false positive and false negative rate.
Probability: The esimated probability that the sample is in the positive category. This assumes that the Bayesian score follows a normal distribution and is different from the prediction using a cutoff.
Enrichment: An estimate of enrichment, that is, the increased likelihood (versus random) of this sample being in the category.
Bayesian Score: The standard Laplacian-modified Bayesian score.
Mahalanobis Distance: The Mahalanobis distance (MD) is the distance to the center of the training data. The larger the MD, the less trustworthy the prediction.
Mahalanobis Distance p-value: The p-value gives the fraction of training data with an MD greater than or equal to the one for the given sample, assuming normally distributed data. The smaller the p-value, the less trustworthy the prediciton. For highly non-normal X properties (e.g., fingerprints), the MD p-value is wildly inaccurate.

Structural Similar Compounds

Name	Ribavirin	Streptozocin	Ascorbic acid
Structure	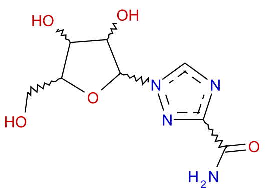	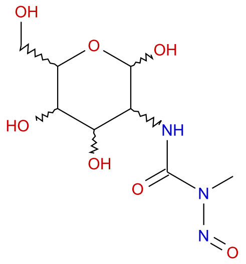	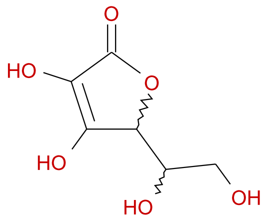
Actual Endpoint	Carcinogen	Carcinogen	Non-Carcinogen
Predicted Endpoint	Carcinogen	Carcinogen	Non-Carcinogen
Distance	0.000	0.590	0.665
Reference	US FDA (Centre for Drug Eval.& Res./Off. Testing & Res.) Sept. 1997	US FDA (Centre for Drug Eval.& Res./Off. Testing & Res.) Sept. 1997	US FDA (Centre for Drug Eval.& Res./Off. Testing & Res.) Sept. 1997

Model Applicability

Unknown features are fingerprint features in the query molecule, but not found in the training set.

1. All properties and OPS components are within expected ranges.

Feature Contribution

Top features for positive contribution

Fingerprint	Bit/Smiles	Feature Structure	Score	Carcinogen in training set
ECFP_12	-553149446	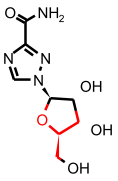 [*]C[C@H]1O[*][*][C@@H]1[*]	0.575	3 out of 4

ECFP_12	370123836	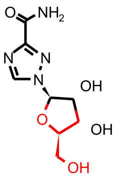 [*][C@H]1[*][*]O[C@@H]1CO	0.575	3 out of 4
ECFP_12	835630791	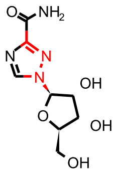 [*]n1:[*]:[*]:[c]([*]):n:1	0.437	2 out of 3
**Top Features for negative contribution**				
**Fingerprint**	**Bit/Smiles**	**Feature Structure**	**Score**	**Carcinogen in training set**
ECFP_12	672362763	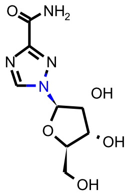 [*]n(:[*]):[*]	-0.198	7 out of 28
ECFP_12	305695353	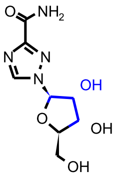 [*][C@@H]1[*][*][C@H]([*])[C@H]1O	-0.154	2 out of 8
ECFP_12	1559650422	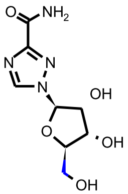 [*]C[*]	-0.112	63 out of 226

AND Enantiomer

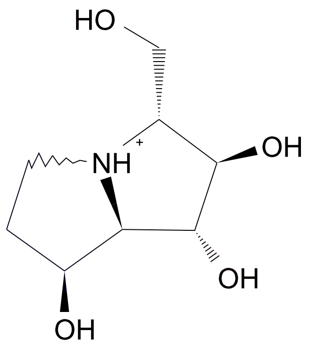

$C_8H_{16}NO_4$
Molecular Weight: 190.21693
ALogP: -3.556
Rotatable Bonds: 1
Acceptors: 4
Donors: 5

Model Prediction

Prediction: Multiple-Carcinogen
Probability: 0.512
Enrichment: 1.370
Bayesian Score: 0.156
Mahalanobis Distance: 13.368
Mahalanobis Distance p-value: 0.000104

Prediction: Positive if the Bayesian score is above the estimated best cutoff value from minimizing the false positive and false negative rate.
Probability: The esimated probability that the sample is in the positive category. This assumes that the Bayesian score follows a normal distribution and is different from the prediction using a cutoff.
Enrichment: An estimate of enrichment, that is, the increased likelihood (versus random) of this sample being in the category.
Bayesian Score: The standard Laplacian-modified Bayesian score.
Mahalanobis Distance: The Mahalanobis distance (MD) is the distance to the center of the training data. The larger the MD, the less trustworthy the prediction.
Mahalanobis Distance p-value: The p-value gives the fraction of training data with an MD greater than or equal to the one for the given sample, assuming normally distributed data. The smaller the p-value, the less trustworthy the prediciton. For highly non-normal X properties (e.g., fingerprints), the MD p-value is wildly inaccurate.

Structural Similar Compounds

Name	Ribavirin	Streptozocin	Zidovudine
Structure	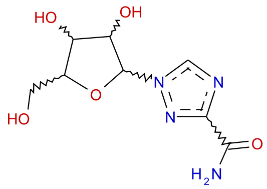	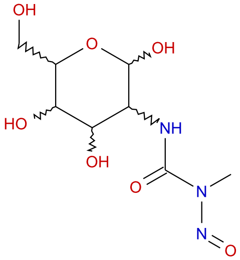	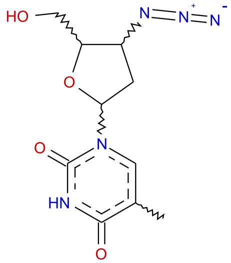
Actual Endpoint	Multiple-Carcinogen	Multiple-Carcinogen	Single-Carcinogen
Predicted Endpoint	Multiple-Carcinogen	Multiple-Carcinogen	Single-Carcinogen
Distance	0.868	0.914	0.991
Reference	US FDA (Centre for Drug Eval.& Res./Off. Testing & Res.) Sept. 1997	US FDA (Centre for Drug Eval.& Res./Off. Testing & Res.) Sept. 1997	US FDA (Centre for Drug Eval.& Res./Off. Testing & Res.) Sept. 1997

Model Applicability

Unknown features are fingerprint features in the query molecule, but not found in the training set.

1. All properties and OPS components are within expected ranges.

Feature Contribution

Top features for positive contribution

Fingerprint	Bit/Smiles	Feature Structure	Score	Multiple-Carcinogen in training set
SCFP_4	0	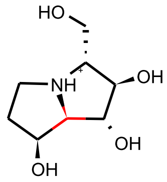 [*]C([*])[*]	0.155	41 out of 111

SCFP_4	-424515134	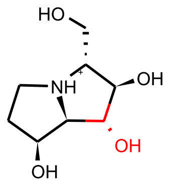 [*]C([*])O	0.131	12 out of 33
SCFP_4	-711686199	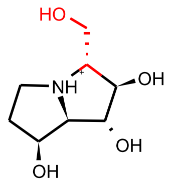 [*]C([*])CO	0.119	9 out of 25
**Top Features for negative contribution**				
**Fingerprint**	**Bit/Smiles**	**Feature Structure**	**Score**	**Multiple-Carcinogen in training set**
SCFP_4	-1272798659	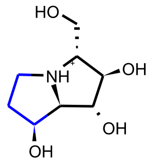 [*][C@@H]1[*][*]CC1	-0.421	10 out of 50
SCFP_4	1702634808	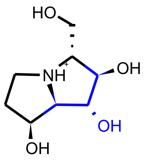 [*][C@@H]1[*][*][C@H]([*])[C@H]1O	0.000	7 out of 21

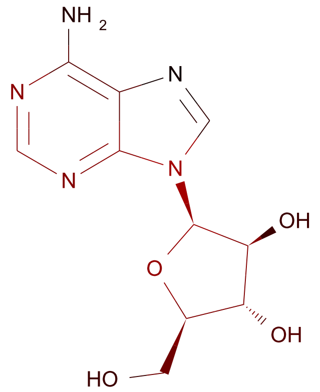

$C_{10}H_{13}N_5O_4$
Molecular Weight: 267.24132
ALogP: -1.881
Rotatable Bonds: 2
Acceptors: 8
Donors: 4

Model Prediction

Prediction: Multiple-Carcinogen
Probability: 0.785
Enrichment: 2.098
Bayesian Score: 7.794
Mahalanobis Distance: 10.001
Mahalanobis Distance p-value: 0.0611

Prediction: Positive if the Bayesian score is above the estimated best cutoff value from minimizing the false positive and false negative rate.
Probability: The esimated probability that the sample is in the positive category. This assumes that the Bayesian score follows a normal distribution and is different from the prediction using a cutoff.
Enrichment: An estimate of enrichment, that is, the increased likelihood (versus random) of this sample being in the category.
Bayesian Score: The standard Laplacian-modified Bayesian score.
Mahalanobis Distance: The Mahalanobis distance (MD) is the distance to the center of the training data. The larger the MD, the less trustworthy the prediction.
Mahalanobis Distance p-value: The p-value gives the fraction of training data with an MD greater than or equal to the one for the given sample, assuming normally distributed data. The smaller the p-value, the less trustworthy the prediciton. For highly non-normal X properties (e.g., fingerprints), the MD p-value is wildly inaccurate.

Structural Similar Compounds

Name	Ribavirin	Streptozocin	Zidovudine
Structure	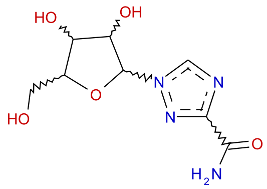	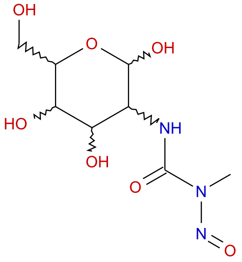	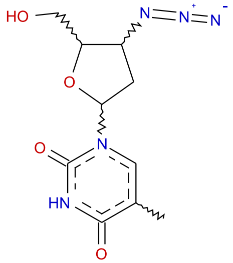
Actual Endpoint	Multiple-Carcinogen	Multiple-Carcinogen	Single-Carcinogen
Predicted Endpoint	Multiple-Carcinogen	Multiple-Carcinogen	Single-Carcinogen
Distance	0.500	0.614	0.775
Reference	US FDA (Centre for Drug Eval.& Res./Off. Testing & Res.) Sept. 1997	US FDA (Centre for Drug Eval.& Res./Off. Testing & Res.) Sept. 1997	US FDA (Centre for Drug Eval.& Res./Off. Testing & Res.) Sept. 1997

Model Applicability

Unknown features are fingerprint features in the query molecule, but not found in the training set.

1. All properties and OPS components are within expected ranges.

Feature Contribution

Top features for positive contribution

Fingerprint	Bit/Smiles	Feature Structure	Score	Multiple-Carcinogen in training set
SCFP_4	-1065373877	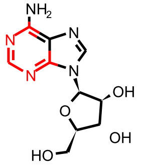 [*][c]1:[*]:[c](:[*]):n:[cH]:n:1	0.721	3 out of 3

SCFP_4	-1181430618	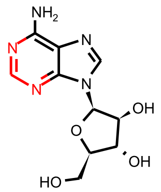 [*]:n:[cH]:n:[*]	0.663	4 out of 5
SCFP_4	2142983647	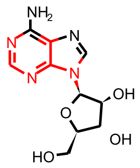 [*]n1:[*]:[*]:[c]2:[*]:n:[cH]:n:[c]:1:2	0.610	2 out of 2
**Top Features for negative contribution**				
**Fingerprint**	**Bit/Smiles**	**Feature Structure**	**Score**	**Multiple-Carcinogen in training set**
SCFP_4	1785884858	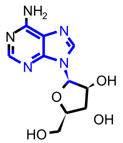 [*]C([*])n1:[cH]:n:[c]2:[c]([*]):[*]:[cH] :n:[c]:1:2	-0.274	0 out of 1
SCFP_4	276193969	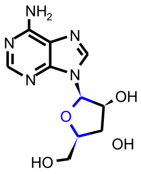 [*][C@@H]1[*][*][C@H] ([*])O1	-0.079	4 out of 14
SCFP_4	1334878018	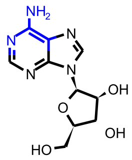 [*]:n:[c](N):[c](:[*]):[*]	-0.066	2 out of 7

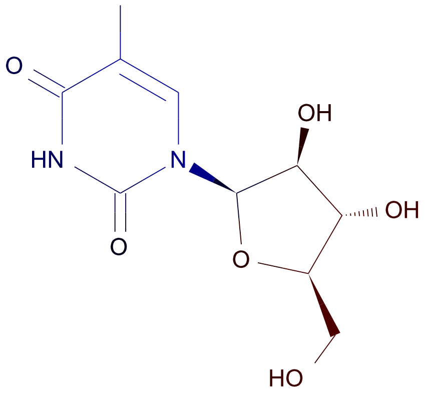

$C_{10}H_{14}N_2O_6$
Molecular Weight: 258.22795
ALogP: -2.005
Rotatable Bonds: 2
Acceptors: 6
Donors: 4

Model Prediction

Prediction: Single-Carcinogen
Probability: 0.432
Enrichment: 1.156
Bayesian Score: -2.416
Mahalanobis Distance: 10.191
Mahalanobis Distance p-value: 0.0455

Prediction: Positive if the Bayesian score is above the estimated best cutoff value from minimizing the false positive and false negative rate.
Probability: The esimated probability that the sample is in the positive category. This assumes that the Bayesian score follows a normal distribution and is different from the prediction using a cutoff.
Enrichment: An estimate of enrichment, that is, the increased likelihood (versus random) of this sample being in the category.
Bayesian Score: The standard Laplacian-modified Bayesian score.
Mahalanobis Distance: The Mahalanobis distance (MD) is the distance to the center of the training data. The larger the MD, the less trustworthy the prediction.
Mahalanobis Distance p-value: The p-value gives the fraction of training data with an MD greater than or equal to the one for the given sample, assuming normally distributed data. The smaller the p-value, the less trustworthy the prediciton. For highly non-normal X properties (e.g., fingerprints), the MD p-value is wildly inaccurate.

Structural Similar Compounds

Name	Zidovudine	Ribavirin	Streptozocin
Structure	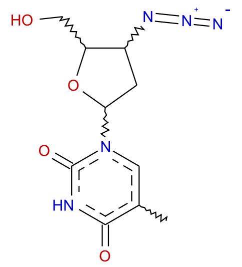	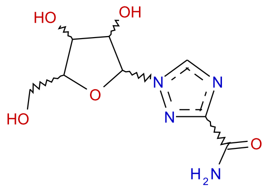	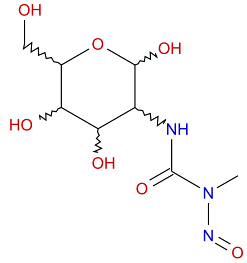
Actual Endpoint	Single-Carcinogen	Multiple-Carcinogen	Multiple-Carcinogen
Predicted Endpoint	Single-Carcinogen	Multiple-Carcinogen	Multiple-Carcinogen
Distance	0.458	0.573	0.650
Reference	US FDA (Centre for Drug Eval.& Res./Off. Testing & Res.) Sept. 1997	US FDA (Centre for Drug Eval.& Res./Off. Testing & Res.) Sept. 1997	US FDA (Centre for Drug Eval.& Res./Off. Testing & Res.) Sept. 1997

Model Applicability

Unknown features are fingerprint features in the query molecule, but not found in the training set.

1. All properties and OPS components are within expected ranges.

Feature Contribution

Top features for positive contribution				
**Fingerprint**	**Bit/Smiles**	**Feature Structure**	**Score**	**Multiple-Carcinogen in training set**
SCFP_4	-1486266146	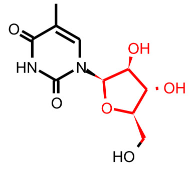 [*]C[C@H]1O[C@@H]([*])[C@@H](O)[C@@H]1O	0.610	2 out of 2

SCFP_4	-1715619483	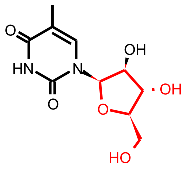<br>[*][C@@H]1O[C@H](CO)[C@@H](O)[C@@H]1[*]	0.610	2 out of 2
SCFP_4	-188384666	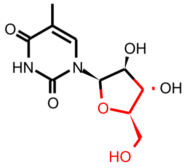<br>[*][C@H]1[*][*]O[C@@H]1CO	0.433	2 out of 3
**Top Features for negative contribution**				
**Fingerprint**	**Bit/Smiles**	**Feature Structure**	**Score**	**Multiple-Carcinogen in training set**
SCFP_4	1257024795	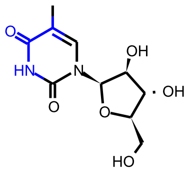<br>[*]NC(=O)C(=[*])[*]	-0.666	0 out of 3
SCFP_4	399659969	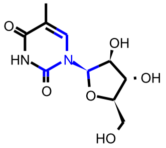<br>[*]C([*])N(C=[*])C(=[*])[*]	-0.666	0 out of 3
SCFP_4	-1971435055	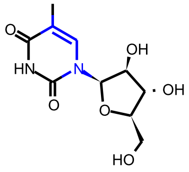<br>[*]N([*])C=C([*])[*]	-0.489	0 out of 2

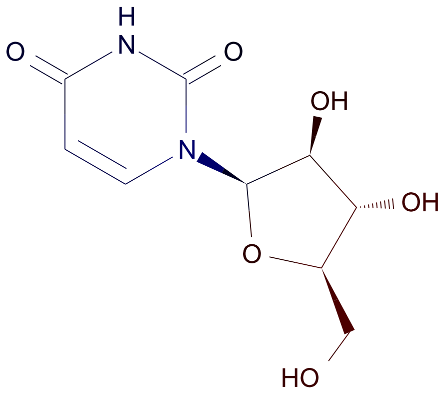

$C_9H_{12}N_2O_6$
Molecular Weight: 244.20137
ALogP: -2.451
Rotatable Bonds: 2
Acceptors: 6
Donors: 4

Structural Similar Compounds

Name	Ribavirin	Zidovudine	Streptozocin
Structure	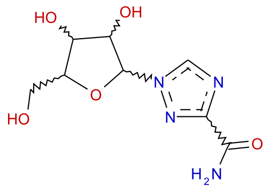	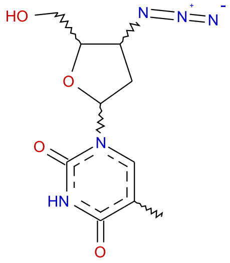	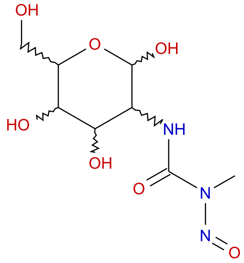
Actual Endpoint	Multiple-Carcinogen	Single-Carcinogen	Multiple-Carcinogen
Predicted Endpoint	Multiple-Carcinogen	Single-Carcinogen	Multiple-Carcinogen
Distance	0.519	0.573	0.630
Reference	US FDA (Centre for Drug Eval.& Res./Off. Testing & Res.) Sept. 1997	US FDA (Centre for Drug Eval.& Res./Off. Testing & Res.) Sept. 1997	US FDA (Centre for Drug Eval.& Res./Off. Testing & Res.) Sept. 1997

Model Prediction

Prediction: Single-Carcinogen
Probability: 0.486
Enrichment: 1.300
Bayesian Score: -0.901
Mahalanobis Distance: 9.758
Mahalanobis Distance p-value: 0.0874

Prediction: Positive if the Bayesian score is above the estimated best cutoff value from minimizing the false positive and false negative rate.
Probability: The esimated probability that the sample is in the positive category. This assumes that the Bayesian score follows a normal distribution and is different from the prediction using a cutoff.
Enrichment: An estimate of enrichment, that is, the increased likelihood (versus random) of this sample being in the category.
Bayesian Score: The standard Laplacian-modified Bayesian score.
Mahalanobis Distance: The Mahalanobis distance (MD) is the distance to the center of the training data. The larger the MD, the less trustworthy the prediction.
Mahalanobis Distance p-value: The p-value gives the fraction of training data with an MD greater than or equal to the one for the given sample, assuming normally distributed data. The smaller the p-value, the less trustworthy the prediciton. For highly non-normal X properties (e.g., fingerprints), the MD p-value is wildly inaccurate.

Model Applicability

Unknown features are fingerprint features in the query molecule, but not found in the training set.

1. All properties and OPS components are within expected ranges.

Feature Contribution

Top features for positive contribution

Fingerprint	Bit/Smiles	Feature Structure	Score	Multiple-Carcinogen in training set
SCFP_4	-1715619483	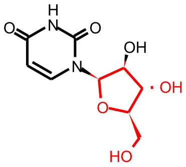 [*][C@@H]1O[C@H](CO)[C@@H](O)[C@@H]1[*]	0.610	2 out of 2

SCFP_4	-1486266146	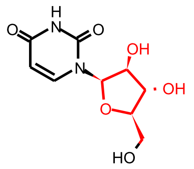 [*]C[C@H]1O[C@@H]([*])[C@@H](O)[C@@H]1O	0.610	2 out of 2
SCFP_4	-188384666	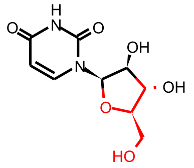 [*][C@H]1[*][*]O[C@@H]1CO	0.433	2 out of 3
**Top Features for negative contribution**				
**Fingerprint**	**Bit/Smiles**	**Feature Structure**	**Score**	**Multiple-Carcinogen in training set**
SCFP_4	399659969	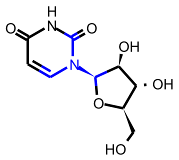 [*]C([*])N(C=[*])C(=[*])[*]	-0.666	0 out of 3
SCFP_4	1257024795	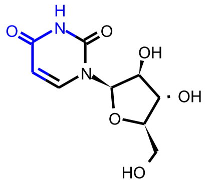 [*]NC(=O)C(=[*])[*]	-0.666	0 out of 3
SCFP_4	-1971435055	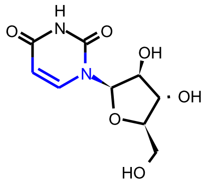 [*]N([*])C=C([*])[*]	-0.489	0 out of 2

Ribavirin

TOPKAT_Rat_Female_FDA_Single_vs_Multiple

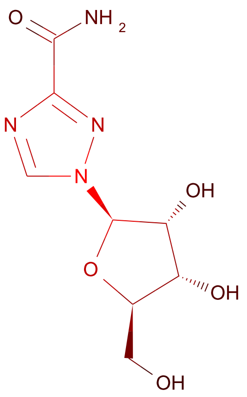

$C_8H_{12}N_4O_5$
Molecular Weight: 244.20467
ALogP: -2.745
Rotatable Bonds: 3
Acceptors: 7
Donors: 4

Model Prediction

Prediction: Multiple-Carcinogen
Probability: 0.836
Enrichment: 2.236
Bayesian Score: 9.443
Mahalanobis Distance: 7.485
Mahalanobis Distance p-value: 0.76

Prediction: Positive if the Bayesian score is above the estimated best cutoff value from minimizing the false positive and false negative rate.
Probability: The esimated probability that the sample is in the positive category. This assumes that the Bayesian score follows a normal distribution and is different from the prediction using a cutoff.
Enrichment: An estimate of enrichment, that is, the increased likelihood (versus random) of this sample being in the category.
Bayesian Score: The standard Laplacian-modified Bayesian score.
Mahalanobis Distance: The Mahalanobis distance (MD) is the distance to the center of the training data. The larger the MD, the less trustworthy the prediction.
Mahalanobis Distance p-value: The p-value gives the fraction of training data with an MD greater than or equal to the one for the given sample, assuming normally distributed data. The smaller the p-value, the less trustworthy the prediciton. For highly non-normal X properties (e.g., fingerprints), the MD p-value is wildly inaccurate.

Structural Similar Compounds

Name	Ribavirin	Streptozocin	Zidovudine
Structure	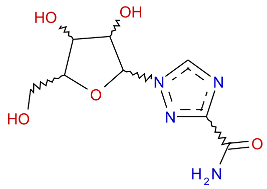	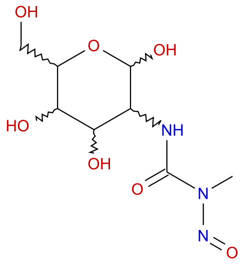	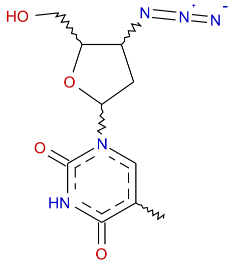
Actual Endpoint	Multiple-Carcinogen	Multiple-Carcinogen	Single-Carcinogen
Predicted Endpoint	Multiple-Carcinogen	Multiple-Carcinogen	Single-Carcinogen
Distance	0.000	0.582	0.765
Reference	US FDA (Centre for Drug Eval.& Res./Off. Testing & Res.) Sept. 1997	US FDA (Centre for Drug Eval.& Res./Off. Testing & Res.) Sept. 1997	US FDA (Centre for Drug Eval.& Res./Off. Testing & Res.) Sept. 1997

Model Applicability

Unknown features are fingerprint features in the query molecule, but not found in the training set.

1. All properties and OPS components are within expected ranges.

Feature Contribution

Top features for positive contribution

Fingerprint	Bit/Smiles	Feature Structure	Score	Multiple-Carcinogen in training set
SCFP_4	-1181430618	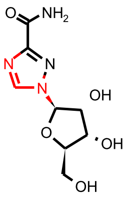 [*]:n:[cH]:n:[*]	0.663	4 out of 5

SCFP_4	-1486266146	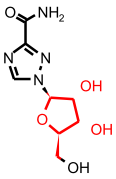<br>[*]C[C@H]1O[C@@H]([*]<br>)[C@@H](O)[C@@H]1O	0.610	2 out of 2
SCFP_4	-1715619483	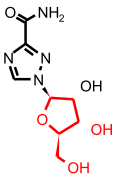<br>[*][C@@H]1O[C@H](CO)[<br>C@@H](O)[C@@H]1[*]	0.610	2 out of 2
**Top Features for negative contribution**				
**Fingerprint**	**Bit/Smiles**	**Feature Structure**	**Score**	**Multiple-Carcinogen in training set**
SCFP_4	276193969	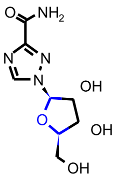<br>[*][C@@H]1[*][*][C@H]<br>([*])O1	-0.079	4 out of 14
SCFP_4	1702634808	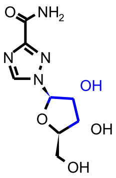<br>[*][C@@H]1[*][*][C@H]<br>([*])[C@H]1O	0.000	7 out of 21

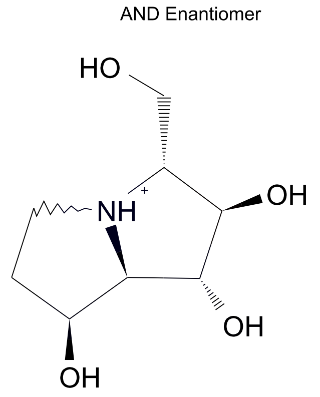

$C_8H_{16}NO_4$
Molecular Weight: 190.21693
ALogP: -3.556
Rotatable Bonds: 1
Acceptors: 4
Donors: 5

Model Prediction

Prediction: Non-Carcinogen
Probability: 0.320
Enrichment: 0.957
Bayesian Score: -1.335
Mahalanobis Distance: 8.333
Mahalanobis Distance p-value: 0.995

Prediction: Positive if the Bayesian score is above the estimated best cutoff value from minimizing the false positive and false negative rate.
Probability: The esimated probability that the sample is in the positive category. This assumes that the Bayesian score follows a normal distribution and is different from the prediction using a cutoff.
Enrichment: An estimate of enrichment, that is, the increased likelihood (versus random) of this sample being in the category.
Bayesian Score: The standard Laplacian-modified Bayesian score.
Mahalanobis Distance: The Mahalanobis distance (MD) is the distance to the center of the training data. The larger the MD, the less trustworthy the prediction.
Mahalanobis Distance p-value: The p-value gives the fraction of training data with an MD greater than or equal to the one for the given sample, assuming normally distributed data. The smaller the p-value, the less trustworthy the prediciton. For highly non-normal X properties (e.g., fingerprints), the MD p-value is wildly inaccurate.

Structural Similar Compounds

Name	Methyldopa	Mannitol	Ascorbic acid
Structure	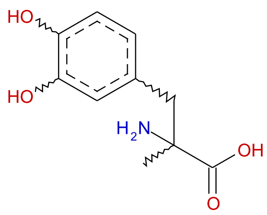	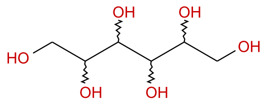	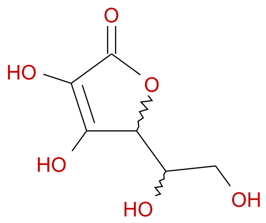
Actual Endpoint	Non-Carcinogen	Non-Carcinogen	Non-Carcinogen
Predicted Endpoint	Non-Carcinogen	Non-Carcinogen	Non-Carcinogen
Distance	0.749	0.811	0.816
Reference	US FDA (Centre for Drug Eval.& Res./Off. Testing & Res.) Sept. 1997	US FDA (Centre for Drug Eval.& Res./Off. Testing & Res.) Sept. 1997	US FDA (Centre for Drug Eval.& Res./Off. Testing & Res.) Sept. 1997

Model Applicability

Unknown features are fingerprint features in the query molecule, but not found in the training set.

1. All properties and OPS components are within expected ranges.

Feature Contribution

Top features for positive contribution

Fingerprint	Bit/Smiles	Feature Structure	Score	Carcinogen in training set
SCFP_6	1702634808	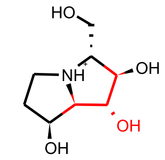 [*][C@@H]1[*][*][C@H]([*])[C@H]1O	0.117	25 out of 69

Top Features for negative contribution

Fingerprint	Bit/Smiles	Feature Structure	Score	Carcinogen in training set

SCFP_6	-1688753455	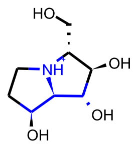 [*][C@H]1[*][*][NH+]2 [*][*][C@H]([*])C12	-0.278	0 out of 1
SCFP_6	11	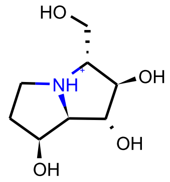 [*][NH+]([*])[*]	-0.278	0 out of 1
SCFP_6	-424515134	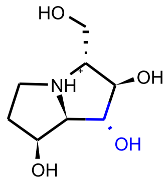 [*]C([*])O	-0.157	30 out of 110

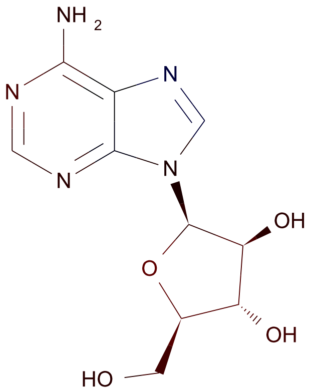

$C_{10}H_{13}N_5O_4$
Molecular Weight: 267.24132
ALogP: -1.881
Rotatable Bonds: 2
Acceptors: 8
Donors: 4

Model Prediction

Prediction: Carcinogen
Probability: 0.427
Enrichment: 1.277
Bayesian Score: 2.266
Mahalanobis Distance: 10.667
Mahalanobis Distance p-value: 0.342

Prediction: Positive if the Bayesian score is above the estimated best cutoff value from minimizing the false positive and false negative rate.
Probability: The esimated probability that the sample is in the positive category. This assumes that the Bayesian score follows a normal distribution and is different from the prediction using a cutoff.
Enrichment: An estimate of enrichment, that is, the increased likelihood (versus random) of this sample being in the category.
Bayesian Score: The standard Laplacian-modified Bayesian score.
Mahalanobis Distance: The Mahalanobis distance (MD) is the distance to the center of the training data. The larger the MD, the less trustworthy the prediction.
Mahalanobis Distance p-value: The p-value gives the fraction of training data with an MD greater than or equal to the one for the given sample, assuming normally distributed data. The smaller the p-value, the less trustworthy the prediciton. For highly non-normal X properties (e.g., fingerprints), the MD p-value is wildly inaccurate.

Structural Similar Compounds

Name	Ribavirin	Streptozocin	Acyclovir
Structure	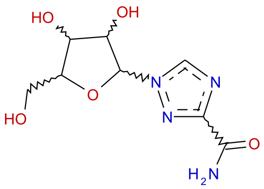	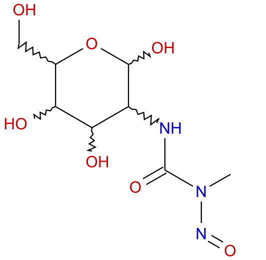	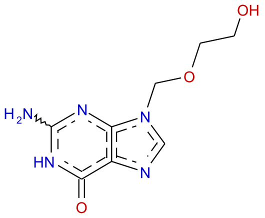
Actual Endpoint	Carcinogen	Carcinogen	Non-Carcinogen
Predicted Endpoint	Carcinogen	Carcinogen	Non-Carcinogen
Distance	0.539	0.606	0.708
Reference	US FDA (Centre for Drug Eval.& Res./Off. Testing & Res.) Sept. 1997	US FDA (Centre for Drug Eval.& Res./Off. Testing & Res.) Sept. 1997	US FDA (Centre for Drug Eval.& Res./Off. Testing & Res.) Sept. 1997

Model Applicability

Unknown features are fingerprint features in the query molecule, but not found in the training set.

1. All properties and OPS components are within expected ranges.

Feature Contribution

Top features for positive contribution				
**Fingerprint**	**Bit/Smiles**	**Feature Structure**	**Score**	**Carcinogen in training set**
SCFP_6	-1065373877	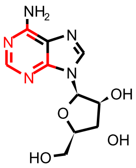 [*][c]1:[*]:[c](:[*]):n:[cH]:n:1	0.429	3 out of 5

SCFP_6	-1559337158	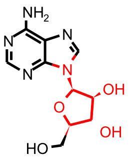 [*][C@H]1O[C@H]([C@@H](O)[C@@H]1O)n(:[*]):[*]	0.415	1 out of 1
SCFP_6	-2147171373	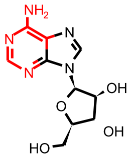 [*]:[c]1:[*]:n:[cH]:n:[c]:1N	0.415	1 out of 1
**Top Features for negative contribution**				
**Fingerprint**	**Bit/Smiles**	**Feature Structure**	**Score**	**Carcinogen in training set**
SCFP_6	-1519771906	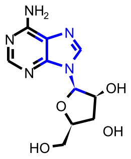 [*]C([*])n1:[cH]:n:[c](:[*]):[c]:1:[*]	-0.484	1 out of 7
SCFP_6	194135988	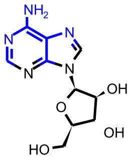 N[c]1:n:[cH]:[*]:[c]2:[*]:[*]:n:[c]:1:2	-0.278	0 out of 1
SCFP_6	-430442553	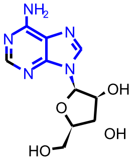 [*]n1:[cH]:n:[c]2:[c](N):n:[*]:n:[c]:1:2	-0.278	0 out of 1

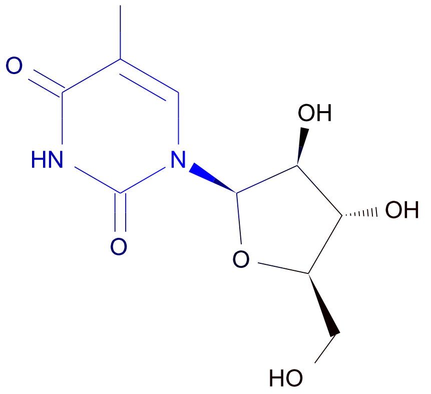

$C_{10}H_{14}N_2O_6$
Molecular Weight: 258.22795
ALogP: -2.005
Rotatable Bonds: 2
Acceptors: 6
Donors: 4

Model Prediction

Prediction: Non-Carcinogen
Probability: 0.202
Enrichment: 0.603
Bayesian Score: -7.231
Mahalanobis Distance: 10.401
Mahalanobis Distance p-value: 0.463

Prediction: Positive if the Bayesian score is above the estimated best cutoff value from minimizing the false positive and false negative rate.
Probability: The esimated probability that the sample is in the positive category. This assumes that the Bayesian score follows a normal distribution and is different from the prediction using a cutoff.
Enrichment: An estimate of enrichment, that is, the increased likelihood (versus random) of this sample being in the category.
Bayesian Score: The standard Laplacian-modified Bayesian score.
Mahalanobis Distance: The Mahalanobis distance (MD) is the distance to the center of the training data. The larger the MD, the less trustworthy the prediction.
Mahalanobis Distance p-value: The p-value gives the fraction of training data with an MD greater than or equal to the one for the given sample, assuming normally distributed data. The smaller the p-value, the less trustworthy the prediciton. For highly non-normal X properties (e.g., fingerprints), the MD p-value is wildly inaccurate.

Structural Similar Compounds

Name	Zidovudine	Methyldopa	Ribavirin
Structure	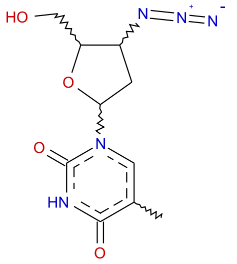	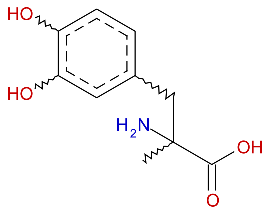	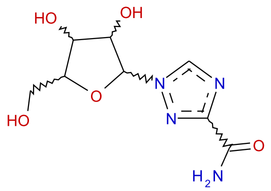
Actual Endpoint	Non-Carcinogen	Non-Carcinogen	Carcinogen
Predicted Endpoint	Non-Carcinogen	Non-Carcinogen	Carcinogen
Distance	0.481	0.602	0.611
Reference	US FDA (Centre for Drug Eval.& Res./Off. Testing & Res.) Sept. 1997	US FDA (Centre for Drug Eval.& Res./Off. Testing & Res.) Sept. 1997	US FDA (Centre for Drug Eval.& Res./Off. Testing & Res.) Sept. 1997

Model Applicability

Unknown features are fingerprint features in the query molecule, but not found in the training set.

1. All properties and OPS components are within expected ranges.

Feature Contribution

Top features for positive contribution				
**Fingerprint**	**Bit/Smiles**	**Feature Structure**	**Score**	**Carcinogen in training set**
SCFP_6	55464376	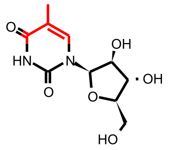 [*]\C=C(\C)/C(=[*])[*]	0.345	14 out of 30

SCFP_6	-1715619483	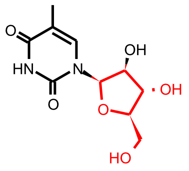<br>[*][C@@H]1O[C@H](CO)[C@@H](O)[C@@H]1[*]	0.273	2 out of 4
SCFP_6	136627117	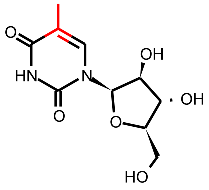<br>[*]C(=[*])C	0.167	18 out of 47

Top Features for negative contribution

Fingerprint	Bit/Smiles	Feature Structure	Score	Carcinogen in training set
SCFP_6	-1971435055	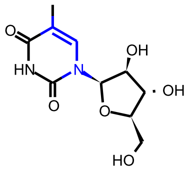<br>[*]N([*])C=C([*])[*]	-0.825	0 out of 4
SCFP_6	399659969	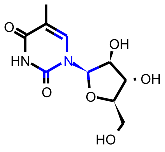<br>[*]C([*])N(C=[*])C(=[*])[*]	-0.578	1 out of 8
SCFP_6	-628337057	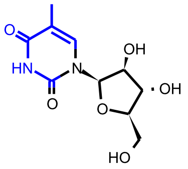<br>[*]=C1[*]C=C(C)C(=O)N1	-0.496	0 out of 2

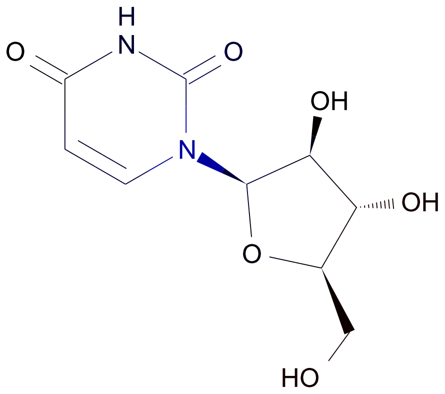

$C_9H_{12}N_2O_6$
Molecular Weight: 244.20137
ALogP: -2.451
Rotatable Bonds: 2
Acceptors: 6
Donors: 4

Structural Similar Compounds

Name	Ribavirin	Methyldopa	Ascorbic acid
Structure	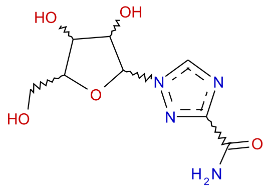	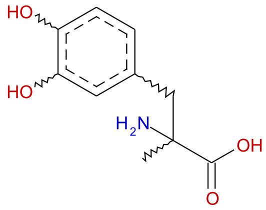	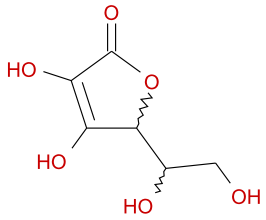
Actual Endpoint	Carcinogen	Non-Carcinogen	Non-Carcinogen
Predicted Endpoint	Carcinogen	Non-Carcinogen	Non-Carcinogen
Distance	0.551	0.623	0.625
Reference	US FDA (Centre for Drug Eval.& Res./Off. Testing & Res.) Sept. 1997	US FDA (Centre for Drug Eval.& Res./Off. Testing & Res.) Sept. 1997	US FDA (Centre for Drug Eval.& Res./Off. Testing & Res.) Sept. 1997

Model Prediction

Prediction: Non-Carcinogen
Probability: 0.257
Enrichment: 0.769
Bayesian Score: -4.047
Mahalanobis Distance: 9.470
Mahalanobis Distance p-value: 0.855

Prediction: Positive if the Bayesian score is above the estimated best cutoff value from minimizing the false positive and false negative rate.
Probability: The esimated probability that the sample is in the positive category. This assumes that the Bayesian score follows a normal distribution and is different from the prediction using a cutoff.
Enrichment: An estimate of enrichment, that is, the increased likelihood (versus random) of this sample being in the category.
Bayesian Score: The standard Laplacian-modified Bayesian score.
Mahalanobis Distance: The Mahalanobis distance (MD) is the distance to the center of the training data. The larger the MD, the less trustworthy the prediction.
Mahalanobis Distance p-value: The p-value gives the fraction of training data with an MD greater than or equal to the one for the given sample, assuming normally distributed data. The smaller the p-value, the less trustworthy the prediciton. For highly non-normal X properties (e.g., fingerprints), the MD p-value is wildly inaccurate.

Model Applicability

Unknown features are fingerprint features in the query molecule, but not found in the training set.

1. All properties and OPS components are within expected ranges.

Feature Contribution

Top features for positive contribution

Fingerprint	Bit/Smiles	Feature Structure	Score	Carcinogen in training set
SCFP_6	1225693839	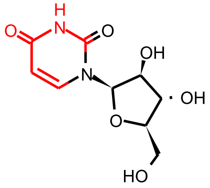 [*]=C1[*]C=CC(=O)N1	0.425	2 out of 3

SCFP_6	-1971196727	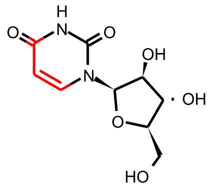 [*]\C=C/C(=[*])[*]	0.361	17 out of 36
SCFP_6	-1715619483	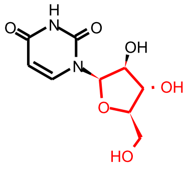 [*][C@@H]1O[C@H](CO)[C@@H](O)[C@@H]1[*]	0.273	2 out of 4
**Top Features for negative contribution**				
**Fingerprint**	**Bit/Smiles**	**Feature Structure**	**Score**	**Carcinogen in training set**
SCFP_6	-1971435055	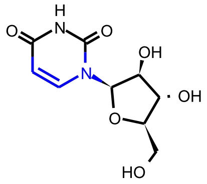 [*]N([*])C=C([*])[*]	-0.825	0 out of 4
SCFP_6	399659969	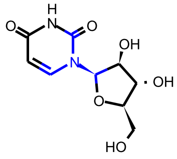 [*]C([*])N(C=[*])C(=[*])[*]	-0.578	1 out of 8
SCFP_6	1061310967	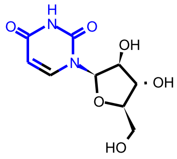 [*]N1[*]=C([*])C(=O)NC1=O	-0.496	0 out of 2

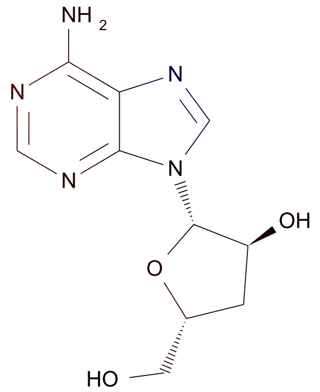

$C_{10}H_{13}N_5O_3$
Molecular Weight: 251.24191
ALogP: -1.308
Rotatable Bonds: 2
Acceptors: 7
Donors: 3

Model Prediction

Prediction: Carcinogen
Probability: 0.414
Enrichment: 1.239
Bayesian Score: 1.879
Mahalanobis Distance: 10.643
Mahalanobis Distance p-value: 0.352

Prediction: Positive if the Bayesian score is above the estimated best cutoff value from minimizing the false positive and false negative rate.
Probability: The esimated probability that the sample is in the positive category. This assumes that the Bayesian score follows a normal distribution and is different from the prediction using a cutoff.
Enrichment: An estimate of enrichment, that is, the increased likelihood (versus random) of this sample being in the category.
Bayesian Score: The standard Laplacian-modified Bayesian score.
Mahalanobis Distance: The Mahalanobis distance (MD) is the distance to the center of the training data. The larger the MD, the less trustworthy the prediction.
Mahalanobis Distance p-value: The p-value gives the fraction of training data with an MD greater than or equal to the one for the given sample, assuming normally distributed data. The smaller the p-value, the less trustworthy the prediciton. For highly non-normal X properties (e.g., fingerprints), the MD p-value is wildly inaccurate.

Structural Similar Compounds

Name	Acyclovir	Zidovudine	Didanosine
Structure	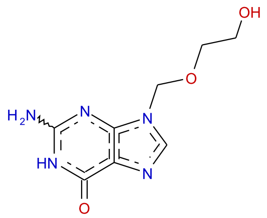	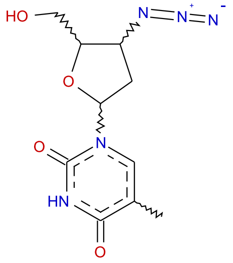	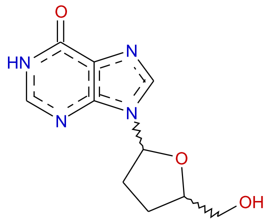
Actual Endpoint	Non-Carcinogen	Non-Carcinogen	Non-Carcinogen
Predicted Endpoint	Non-Carcinogen	Non-Carcinogen	Non-Carcinogen
Distance	0.592	0.617	0.652
Reference	US FDA (Centre for Drug Eval.& Res./Off. Testing & Res.) Sept. 1997	US FDA (Centre for Drug Eval.& Res./Off. Testing & Res.) Sept. 1997	US FDA (Centre for Drug Eval.& Res./Off. Testing & Res.) Sept. 1997

Model Applicability

Unknown features are fingerprint features in the query molecule, but not found in the training set.

1. All properties and OPS components are within expected ranges.

Feature Contribution

Top features for positive contribution

Fingerprint	Bit/Smiles	Feature Structure	Score	Carcinogen in training set
SCFP_6	-1905051945	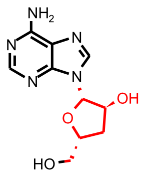 [*]C[C@H]1C[C@H](O)[C@@H]([*])O1	0.561	3 out of 4

SCFP_6	-1065373877	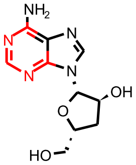 [*][c]1:[*]:[c](:[*]) :n:[cH]:n:1	0.429	3 out of 5
SCFP_6	-2147171373	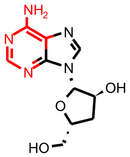 [*]:[c]1:[*]:n:[cH]:n :[c]:1N	0.415	1 out of 1

Top Features for negative contribution

Fingerprint	Bit/Smiles	Feature Structure	Score	Carcinogen in training set
SCFP_6	-1519771906	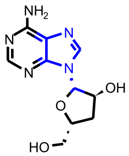 [*]C([*])n1:[cH]:n:[c](:[*]):[c]:1:[*]	-0.484	1 out of 7
SCFP_6	-1618236862	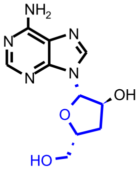 [*][C@H]1C[C@H](CO)O[C@@H]1[*]	-0.278	0 out of 1
SCFP_6	194135988	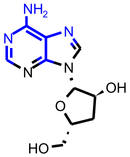 N[c]1:n:[cH]:[*]:[c]2 :[*]:[*]:n:[c]:1:2	-0.278	0 out of 1

Ribavirin

TOPKAT_Rat_Male_FDA_None_vs_Carcinogen

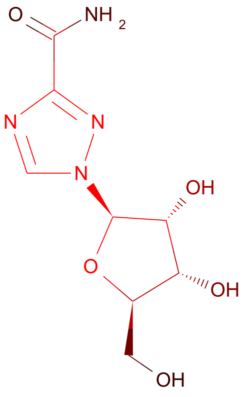

$C_8H_{12}N_4O_5$

Molecular Weight: 244.20467

ALogP: -2.745

Rotatable Bonds: 3

Acceptors: 7

Donors: 4

Model Prediction

Prediction: Carcinogen

Probability: 0.695

Enrichment: 2.080

Bayesian Score: 9.565

Mahalanobis Distance: 8.604

Mahalanobis Distance p-value: 0.986

Prediction: Positive if the Bayesian score is above the estimated best cutoff value from minimizing the false positive and false negative rate.
Probability: The esimated probability that the sample is in the positive category. This assumes that the Bayesian score follows a normal distribution and is different from the prediction using a cutoff.
Enrichment: An estimate of enrichment, that is, the increased likelihood (versus random) of this sample being in the category.
Bayesian Score: The standard Laplacian-modified Bayesian score.
Mahalanobis Distance: The Mahalanobis distance (MD) is the distance to the center of the training data. The larger the MD, the less trustworthy the prediction.
Mahalanobis Distance p-value: The p-value gives the fraction of training data with an MD greater than or equal to the one for the given sample, assuming normally distributed data. The smaller the p-value, the less trustworthy the prediciton. For highly non-normal X properties (e.g., fingerprints), the MD p-value is wildly inaccurate.

Structural Similar Compounds

Name	Ribavirin	Streptozocin	Ascorbic acid
Structure	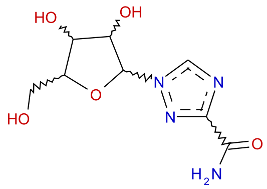	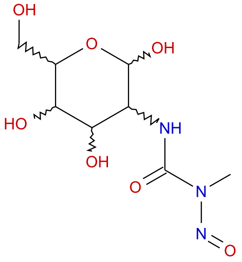	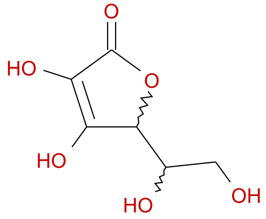
Actual Endpoint	Carcinogen	Carcinogen	Non-Carcinogen
Predicted Endpoint	Carcinogen	Carcinogen	Non-Carcinogen
Distance	0.000	0.578	0.614
Reference	US FDA (Centre for Drug Eval.& Res./Off. Testing & Res.) Sept. 1997	US FDA (Centre for Drug Eval.& Res./Off. Testing & Res.) Sept. 1997	US FDA (Centre for Drug Eval.& Res./Off. Testing & Res.) Sept. 1997

Model Applicability

Unknown features are fingerprint features in the query molecule, but not found in the training set.

1. All properties and OPS components are within expected ranges.

Feature Contribution

Top features for positive contribution

Fingerprint	Bit/Smiles	Feature Structure	Score	Carcinogen in training set
SCFP_6	599957850	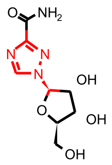 [*]C([*])n1:[cH]:n:[c]([*]):n:1	0.712	3 out of 3

SCFP_6	-443296553	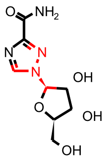 [*]C([*])n1:[cH]:[*]:[*]:n:1	0.653	4 out of 5
SCFP_6	149212520	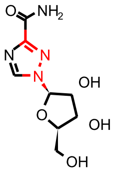 [*]n1:[*]:[*]:[c]([*]):n:1	0.543	9 out of 15

Top Features for negative contribution

Fingerprint	Bit/Smiles	Feature Structure	Score	Carcinogen in training set
SCFP_6	1257084377	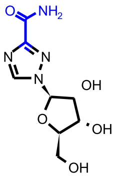 [*]:[c](:[*])C(=O)N	-0.436	4 out of 21
SCFP_6	-424515134	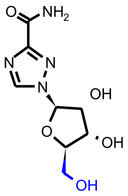 [*]C([*])O	-0.157	30 out of 110
SCFP_6	9	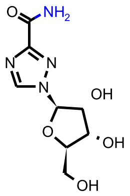 [*]N([*])[*]	-0.055	40 out of 132

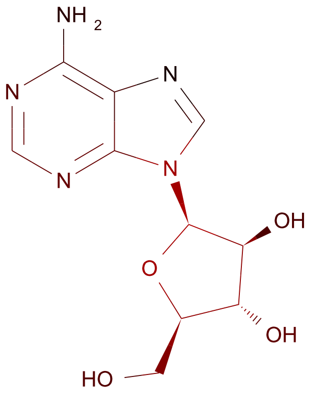

$C_{10}H_{13}N_5O_4$
Molecular Weight: 267.24132
ALogP: -1.881
Rotatable Bonds: 2
Acceptors: 8
Donors: 4

Model Prediction

Prediction: Multiple-Carcinogen
Probability: 0.647
Enrichment: 1.563
Bayesian Score: 7.633
Mahalanobis Distance: 13.760
Mahalanobis Distance p-value: 0.000142

Prediction: Positive if the Bayesian score is above the estimated best cutoff value from minimizing the false positive and false negative rate.
Probability: The esimated probability that the sample is in the positive category. This assumes that the Bayesian score follows a normal distribution and is different from the prediction using a cutoff.
Enrichment: An estimate of enrichment, that is, the increased likelihood (versus random) of this sample being in the category.
Bayesian Score: The standard Laplacian-modified Bayesian score.
Mahalanobis Distance: The Mahalanobis distance (MD) is the distance to the center of the training data. The larger the MD, the less trustworthy the prediction.
Mahalanobis Distance p-value: The p-value gives the fraction of training data with an MD greater than or equal to the one for the given sample, assuming normally distributed data. The smaller the p-value, the less trustworthy the prediciton. For highly non-normal X properties (e.g., fingerprints), the MD p-value is wildly inaccurate.

Structural Similar Compounds

Name	Ribavirin	Streptozocin	Cimetidine
Structure	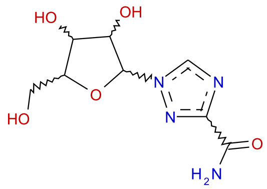	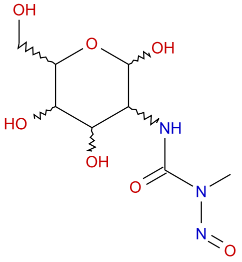	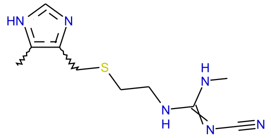
Actual Endpoint	Multiple-Carcinogen	Multiple-Carcinogen	Multiple-Carcinogen
Predicted Endpoint	Multiple-Carcinogen	Multiple-Carcinogen	Multiple-Carcinogen
Distance	0.562	0.654	0.966
Reference	US FDA (Centre for Drug Eval.& Res./Off. Testing & Res.) Sept. 1997	US FDA (Centre for Drug Eval.& Res./Off. Testing & Res.) Sept. 1997	US FDA (Centre for Drug Eval.& Res./Off. Testing & Res.) Sept. 1997

Model Applicability

Unknown features are fingerprint features in the query molecule, but not found in the training set.

1. All properties and OPS components are within expected ranges.

Feature Contribution

Top features for positive contribution

Fingerprint	Bit/Smiles	Feature Structure	Score	Multiple-Carcinogen in training set
SCFP_8	-188384666	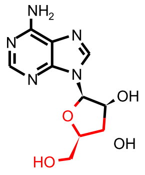 [*][C@H]1[*][*]O[C@@H]1CO	0.649	3 out of 3

SCFP_8	-1065373877	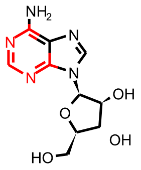 [*][c]1:[*]:[c](:[*]) :n:[cH]:n:1	0.649	3 out of 3
SCFP_8	-1715619483	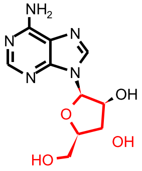 [*][C@@H]1O[C@H](CO)[C@@H](O)[C@@H]1[*]	0.553	2 out of 2
**Top Features for negative contribution**				
**Fingerprint**	**Bit/Smiles**	**Feature Structure**	**Score**	**Multiple-Carcinogen in training set**
SCFP_8	112554633	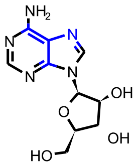 [*][c](:[*]):[c]1:n:[*]:[*]:[c]:1:[*]	-0.292	3 out of 12
SCFP_8	8	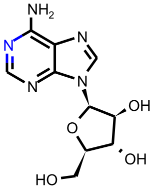 [*]:n:[*]	-0.154	12 out of 39
SCFP_8	276193969	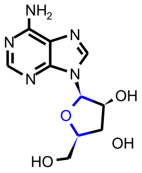 [*][C@@H]1[*][*][C@H] ([*])O1	-0.072	5 out of 15

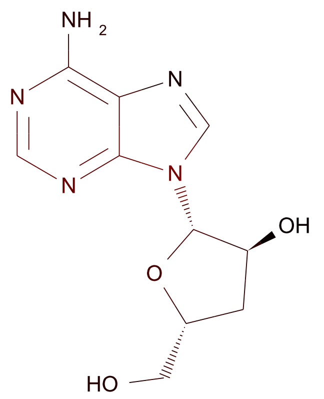

$C_{10}H_{13}N_5O_3$
Molecular Weight: 251.24191
ALogP: -1.308
Rotatable Bonds: 2
Acceptors: 7
Donors: 3

Model Prediction

Prediction: Multiple-Carcinogen
Probability: 0.596
Enrichment: 1.440
Bayesian Score: 5.549
Mahalanobis Distance: 15.349
Mahalanobis Distance p-value: 5.22e-006

Prediction: Positive if the Bayesian score is above the estimated best cutoff value from minimizing the false positive and false negative rate.
Probability: The esimated probability that the sample is in the positive category. This assumes that the Bayesian score follows a normal distribution and is different from the prediction using a cutoff.
Enrichment: An estimate of enrichment, that is, the increased likelihood (versus random) of this sample being in the category.
Bayesian Score: The standard Laplacian-modified Bayesian score.
Mahalanobis Distance: The Mahalanobis distance (MD) is the distance to the center of the training data. The larger the MD, the less trustworthy the prediction.
Mahalanobis Distance p-value: The p-value gives the fraction of training data with an MD greater than or equal to the one for the given sample, assuming normally distributed data. The smaller the p-value, the less trustworthy the prediciton. For highly non-normal X properties (e.g., fingerprints), the MD p-value is wildly inaccurate.

Structural Similar Compounds

Name	Ribavirin	Dacarbazine	Monocrotaline
Structure	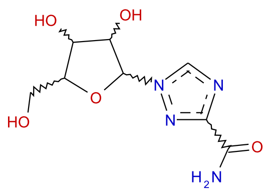	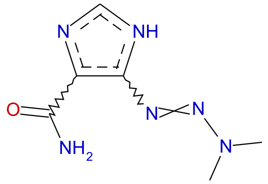	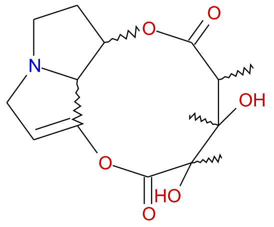
Actual Endpoint	Multiple-Carcinogen	Multiple-Carcinogen	Single-Carcinogen
Predicted Endpoint	Multiple-Carcinogen	Multiple-Carcinogen	Single-Carcinogen
Distance	0.709	0.777	0.822
Reference	US FDA (Centre for Drug Eval.& Res./Off. Testing & Res.) Sept. 1997	US FDA (Centre for Drug Eval.& Res./Off. Testing & Res.) Sept. 1997	US FDA (Centre for Drug Eval.& Res./Off. Testing & Res.) Sept. 1997

Model Applicability

Unknown features are fingerprint features in the query molecule, but not found in the training set.

1. All properties and OPS components are within expected ranges.

Feature Contribution

Top features for positive contribution

Fingerprint	Bit/Smiles	Feature Structure	Score	Multiple-Carcinogen in training set
SCFP_8	-188384666	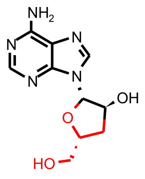 [*][C@H]1[*][*]O[C@@H]1CO	0.649	3 out of 3

SCFP_8	-1065373877	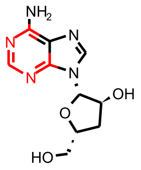 [*][c]1:[*]:[c](:[*]) :n:[cH]:n:1	0.649	3 out of 3
SCFP_8	-1181430618	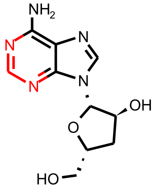 [*]:n:[cH]:n:[*]	0.453	4 out of 6
**Top Features for negative contribution**				
**Fingerprint**	**Bit/Smiles**	**Feature Structure**	**Score**	**Multiple-Carcinogen in training set**
SCFP_8	112554633	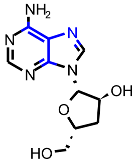 [*][c](:[*]):[c]1:n:[*]:[*]:[c]:1:[*]	-0.292	3 out of 12
SCFP_8	8	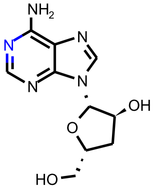 [*]:n:[*]	-0.154	12 out of 39
SCFP_8	-1272798659	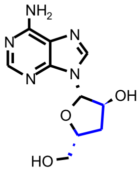 [*][C@@H]1[*][*][C@@H]([*])C1	-0.100	16 out of 49

Ribavirin

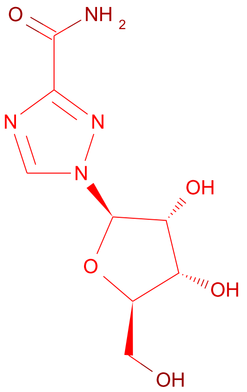

$C_8H_{12}N_4O_5$

Molecular Weight: 244.20467

ALogP: -2.745

Rotatable Bonds: 3

Acceptors: 7

Donors: 4

Model Prediction

Prediction: Multiple-Carcinogen

Probability: 0.733

Enrichment: 1.771

Bayesian Score: 12.770

Mahalanobis Distance: 8.323

Mahalanobis Distance p-value: 0.587

Prediction: Positive if the Bayesian score is above the estimated best cutoff value from minimizing the false positive and false negative rate.
Probability: The esimated probability that the sample is in the positive category. This assumes that the Bayesian score follows a normal distribution and is different from the prediction using a cutoff.
Enrichment: An estimate of enrichment, that is, the increased likelihood (versus random) of this sample being in the category.
Bayesian Score: The standard Laplacian-modified Bayesian score.
Mahalanobis Distance: The Mahalanobis distance (MD) is the distance to the center of the training data. The larger the MD, the less trustworthy the prediction.
Mahalanobis Distance p-value: The p-value gives the fraction of training data with an MD greater than or equal to the one for the given sample, assuming normally distributed data. The smaller the p-value, the less trustworthy the prediciton. For highly non-normal X properties (e.g., fingerprints), the MD p-value is wildly inaccurate.

Structural Similar Compounds

Name	Ribavirin	Streptozocin	Azaserine
Structure	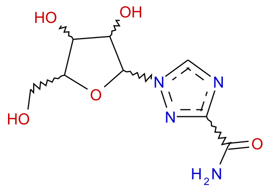	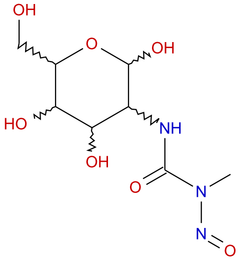	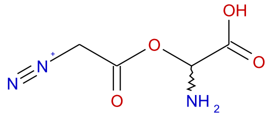
Actual Endpoint	Multiple-Carcinogen	Multiple-Carcinogen	Single-Carcinogen
Predicted Endpoint	Multiple-Carcinogen	Multiple-Carcinogen	Single-Carcinogen
Distance	0.000	0.631	0.902
Reference	US FDA (Centre for Drug Eval.& Res./Off. Testing & Res.) Sept. 1997	US FDA (Centre for Drug Eval.& Res./Off. Testing & Res.) Sept. 1997	US FDA (Centre for Drug Eval.& Res./Off. Testing & Res.) Sept. 1997

Model Applicability

Unknown features are fingerprint features in the query molecule, but not found in the training set.

1. All properties and OPS components are within expected ranges.

Feature Contribution

Top features for positive contribution				
**Fingerprint**	**Bit/Smiles**	**Feature Structure**	**Score**	**Multiple-Carcinogen in training set**
SCFP_8	-188384666	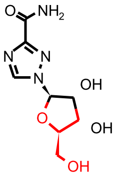 [*][C@H]1[*][*]O[C@@H]1CO	0.649	3 out of 3

SCFP_8	-1715619483	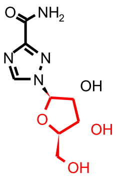 [*][C@@H]1O[C@H](CO)[C@@H](O)[C@@H]1[*]	0.553	2 out of 2
SCFP_8	-1486266146	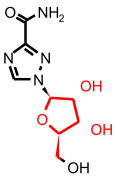 [*]C[C@H]1O[C@@H]([*])[C@@H](O)[C@@H]1O	0.553	2 out of 2
**Top Features for negative contribution**				
**Fingerprint**	**Bit/Smiles**	**Feature Structure**	**Score**	**Multiple-Carcinogen in training set**
SCFP_8	-443296553	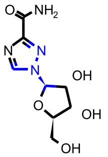 [*]C([*])n1:[cH]:[*]:[*]:n:1	-0.204	1 out of 4
SCFP_8	8	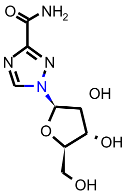 [*]:n:[*]	-0.154	12 out of 39
SCFP_8	276193969	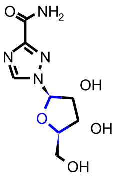 [*][C@@H]1[*][*][C@H]([*])O1	-0.072	5 out of 15

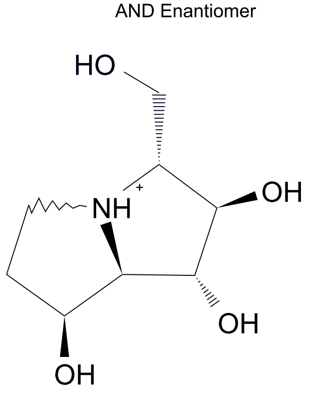

$C_8H_{16}NO_4$
Molecular Weight: 190.21693
ALogP: -3.556
Rotatable Bonds: 1
Acceptors: 4
Donors: 5

Model Prediction

Prediction: Mild
Probability: 0.225
Enrichment: 0.612
Bayesian Score: -4.500
Mahalanobis Distance: 10.706
Mahalanobis Distance p-value: 0.0029

Prediction: Positive if the Bayesian score is above the estimated best cutoff value from minimizing the false positive and false negative rate.
Probability: The esimated probability that the sample is in the positive category. This assumes that the Bayesian score follows a normal distribution and is different from the prediction using a cutoff.
Enrichment: An estimate of enrichment, that is, the increased likelihood (versus random) of this sample being in the category.
Bayesian Score: The standard Laplacian-modified Bayesian score.
Mahalanobis Distance: The Mahalanobis distance (MD) is the distance to the center of the training data. The larger the MD, the less trustworthy the prediction.
Mahalanobis Distance p-value: The p-value gives the fraction of training data with an MD greater than or equal to the one for the given sample, assuming normally distributed data. The smaller the p-value, the less trustworthy the prediciton. For highly non-normal X properties (e.g., fingerprints), the MD p-value is wildly inaccurate.

Structural Similar Compounds

Name	Ethanol, 2,2',2'',2'''-silanetetrayltetrakis-	Propionamidine, 2,2'-azobis(2-methyl-, dihydrochloride	Glycerol
Structure	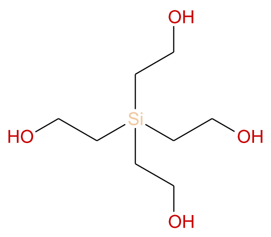	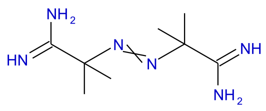	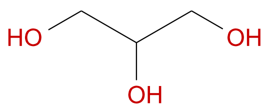
Actual Endpoint	Mild	Mild	Mild
Predicted Endpoint	Mild	Mild	Mild
Distance	0.930	0.945	0.958
Reference	85JCAE "Prehled Prumyslove Toxikologie; Organicke Latky," Marhold, J., Prague , Czechoslovakia, Avicenum, 1986 Volume(issue)/page/year: -,1232,1986	EPASR* United States Environmental Protection Agency, Office of Pesticides an d Toxic Substances. (U.S. Environmental Protection Agency, 401 M St., SW, Washin gton, DC 20460) History unknown. Volume(issue)/pag	85JCAE "Prehled Prumyslove Toxikologie; Organicke Latky," Marhold, J., Prague , Czechoslovakia, Avicenum, 1986 Volume(issue)/page/year: -,207,1986

Model Applicability

Unknown features are fingerprint features in the query molecule, but not found in the training set.

1. OPS PC1 out of range. Value: -3.8188. Training min, max, SD, explained variance: -3.7938, 19.107, 3.41, 0.1570.
2. Unknown FCFP_2 feature: 10: [*][NH+]([*])[*]
3. Unknown FCFP_2 feature: -1853714334: [*][C@@H]1[*][*][C@H]2[*][*]C[NH+]12
4. Unknown FCFP_2 feature: -1817836174: [*][C@H]1[*][*][NH+]2[*][*][C@H]([*])C12
5. Unknown FCFP_2 feature: 1155241219: [*][NH+]1[*][*]CC1

Feature Contribution

Top features for positive contribution

Fingerprint	Bit/Smiles	Feature Structure	Score	Moderate_Severe in training set

FCFP_12	-1272798659	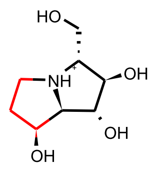 [*][C@@H]1[*][*]CC1	0.204	227 out of 513
FCFP_12	-1043250487	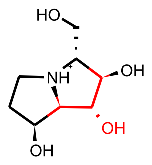 [*][C@@H]1[*][*][C@H]([*])[C@H]1O	0.060	15 out of 39

Top Features for negative contribution

**Fingerprint**	**Bit/Smiles**	**Feature Structure**	**Score**	**Moderate_Severe in training set**
FCFP_12	-1272709286	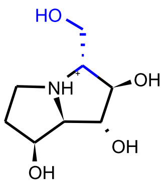 [*]C([*])CO	-0.475	18 out of 82
FCFP_12	3	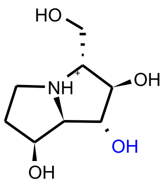 [*]O	-0.234	58 out of 204
FCFP_12	1070061035	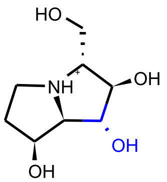 [*]C([*])O	-0.144	47 out of 151

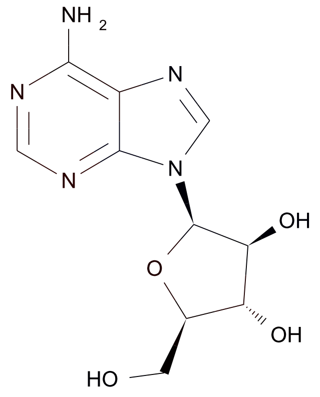

$C_{10}H_{13}N_5O_4$
Molecular Weight: 267.24132
ALogP: -1.881
Rotatable Bonds: 2
Acceptors: 8
Donors: 4

Structural Similar Compounds

Name	Citric acid	Benzene-1,3-dicarboxylic acid, 5-sulfo-	1,5-Naphthalenedisulfonic acid, 2-amino-
Structure	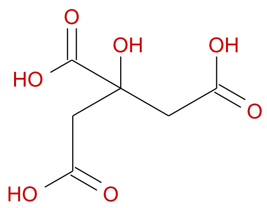	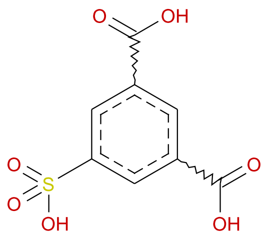	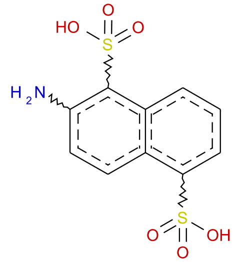
Actual Endpoint	Mild	Mild	Mild
Predicted Endpoint	Mild	Mild	Mild
Distance	0.767	0.807	0.810
Reference	85JCAE "Prehled Prumyslove Toxikologie; Organicke Latky," Marhold, J., Prague , Czechoslovakia, Avicenum, 1986 Volume(issue)/page/year: -,658,1986	85JCAE "Prehled Prumyslove Toxikologie; Organicke Latky," Marhold, J., Prague , Czechoslovakia, Avicenum, 1986 Volume(issue)/page/year: -,1056,1986	85JCAE "Prehled Prumyslove Toxikologie; Organicke Latky," Marhold, J., Prague , Czechoslovakia, Avicenum, 1986 Volume(issue)/page/year: -,1058,1986

Model Prediction

Prediction: Mild
Probability: 0.201
Enrichment: 0.545
Bayesian Score: -5.137
Mahalanobis Distance: 10.534
Mahalanobis Distance p-value: 0.00545

Prediction: Positive if the Bayesian score is above the estimated best cutoff value from minimizing the false positive and false negative rate.
Probability: The esimated probability that the sample is in the positive category. This assumes that the Bayesian score follows a normal distribution and is different from the prediction using a cutoff.
Enrichment: An estimate of enrichment, that is, the increased likelihood (versus random) of this sample being in the category.
Bayesian Score: The standard Laplacian-modified Bayesian score.
Mahalanobis Distance: The Mahalanobis distance (MD) is the distance to the center of the training data. The larger the MD, the less trustworthy the prediction.
Mahalanobis Distance p-value: The p-value gives the fraction of training data with an MD greater than or equal to the one for the given sample, assuming normally distributed data. The smaller the p-value, the less trustworthy the prediciton. For highly non-normal X properties (e.g., fingerprints), the MD p-value is wildly inaccurate.

Model Applicability

Unknown features are fingerprint features in the query molecule, but not found in the training set.

1. All properties and OPS components are within expected ranges.
2. Unknown FCFP_2 feature: -1564473960: [*]n1:[*]:[*]:[c](:[*]):[c]:1:n:[*]
3. Unknown FCFP_2 feature: 1315416442: [*][C@H]1[*][*]O[C@H]1n(:[*]):[*]

Feature Contribution

Top features for positive contribution

Fingerprint	Bit/Smiles	Feature Structure	Score	Moderate_Severe in training set

FCFP_12	-1151884458	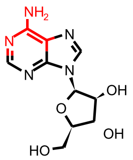<br>[*]:n:[c](N):[c](:[*]):[*]	0.385	1 out of 1
FCFP_12	76292238	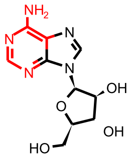<br>[*]:[c]1:[*]:n:[cH]:n:[c]:1N	0.385	1 out of 1
FCFP_12	-2091721556	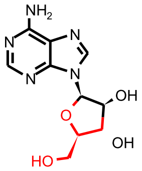<br>[*][C@H]1[*][*]O[C@@H]1CO	0.365	2 out of 3

Top Features for negative contribution

Fingerprint	Bit/Smiles	Feature Structure	Score	Moderate_Severe in training set
FCFP_12	-1272709286	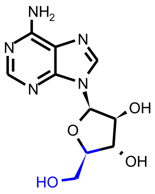<br>[*]C([*])CO	-0.475	18 out of 82
FCFP_12	178336375	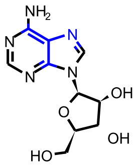<br>[*][c](:[*]):[c]1:n:[*]:[*]:[c]:1:[*]	-0.413	3 out of 14

FCFP_12	1069584379	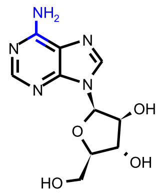 [*]:[c](:[*])N	-0.360	8 out of 33

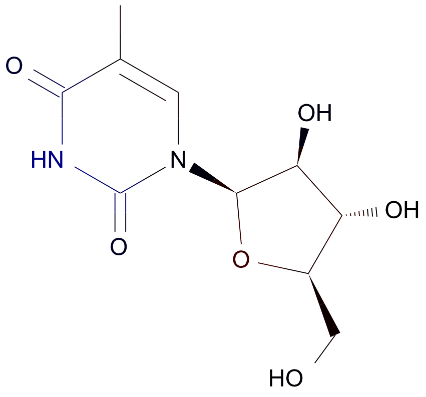

$C_{10}H_{14}N_2O_6$
Molecular Weight: 258.22795
ALogP: -2.005
Rotatable Bonds: 2
Acceptors: 6
Donors: 4

Model Prediction

Prediction: Mild
Probability: 0.165
Enrichment: 0.448
Bayesian Score: -6.135
Mahalanobis Distance: 14.296
Mahalanobis Distance p-value: 3.17e-012

Prediction: Positive if the Bayesian score is above the estimated best cutoff value from minimizing the false positive and false negative rate.
Probability: The esimated probability that the sample is in the positive category. This assumes that the Bayesian score follows a normal distribution and is different from the prediction using a cutoff.
Enrichment: An estimate of enrichment, that is, the increased likelihood (versus random) of this sample being in the category.
Bayesian Score: The standard Laplacian-modified Bayesian score.
Mahalanobis Distance: The Mahalanobis distance (MD) is the distance to the center of the training data. The larger the MD, the less trustworthy the prediction.
Mahalanobis Distance p-value: The p-value gives the fraction of training data with an MD greater than or equal to the one for the given sample, assuming normally distributed data. The smaller the p-value, the less trustworthy the prediciton. For highly non-normal X properties (e.g., fingerprints), the MD p-value is wildly inaccurate.

Structural Similar Compounds

Name	Propionamidine, 2,2'-azobis(2-methyl-, dihydrochloride	Glutamic acid, N-(p-(methylamino)benzoyl)-, sodium salt	Citric acid
Structure	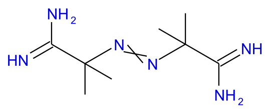	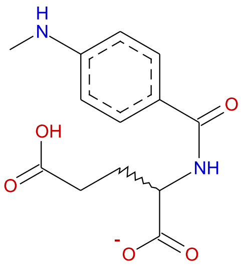	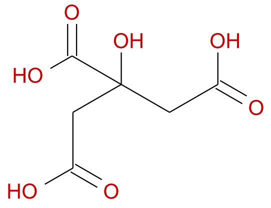
Actual Endpoint	Mild	Mild	Mild
Predicted Endpoint	Mild	Mild	Mild
Distance	0.780	0.805	0.808
Reference	EPASR* United States Environmental Protection Agency, Office of Pesticides an d Toxic Substances. (U.S. Environmental Protection Agency, 401 M St., SW, Washin gton, DC 20460) History unknown. Volume(issue)/pag	FCTOD7 Food and Chemical Toxicology. (Pergamon Press Inc., Maxwell House, Fa irview Park, Elmsford, NY 10523) V.20- 1982- Volume(issue)/page/year: 20,563 ,1982	85JCAE "Prehled Prumyslove Toxikologie; Organicke Latky," Marhold, J., Prague , Czechoslovakia, Avicenum, 1986 Volume(issue)/page/year: -,658,1986

Model Applicability

Unknown features are fingerprint features in the query molecule, but not found in the training set.

1. All properties and OPS components are within expected ranges.

Feature Contribution

Top features for positive contribution				
Fingerprint	Bit/Smiles	Feature Structure	Score	Moderate_Severe in training set

FCFP_12	436886043	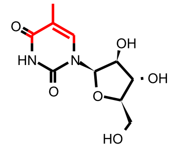 [*]\C=C(\C)/C(=[*])[*]	0.503	68 out of 113
FCFP_12	699500266	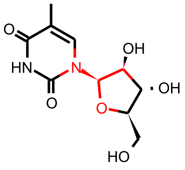 [*][C@H]1[*][*]O[C@H] 1N([*])[*]	0.441	14 out of 24
FCFP_12	-2091721556	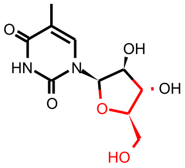 [*][C@H]1[*][*]O[C@@H]1CO	0.365	2 out of 3

Top Features for negative contribution

Fingerprint	Bit/Smiles	Feature Structure	Score	Moderate_Severe in training set
FCFP_12	-885550502	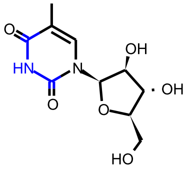 [*]C(=[*])NC(=[*])[*]	-1.050	2 out of 21
FCFP_12	566058135	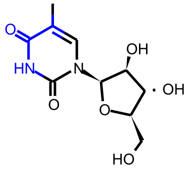 [*]NC(=O)C(=[*])[*]	-0.909	1 out of 11

FCFP_12	-1986098826	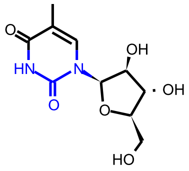 [*]NC(=O)N([*])[*]	-0.733	0 out of 3

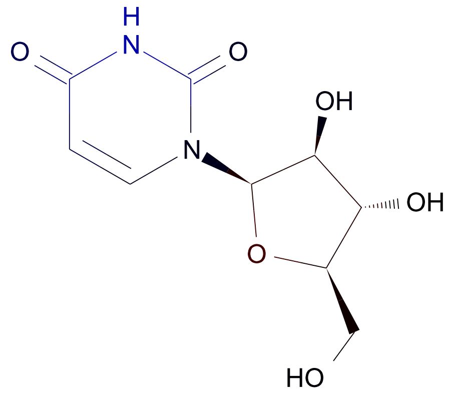

$C_9H_{12}N_2O_6$
Molecular Weight: 244.20137
ALogP: -2.451
Rotatable Bonds: 2
Acceptors: 6
Donors: 4

Structural Similar Compounds

Name	Citric acid	Propionamidine, 2,2'-azobis(2-methyl-, dihydrochloride	Benzenesulfonic acid, 2,4-diamino-
Structure	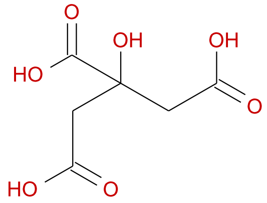	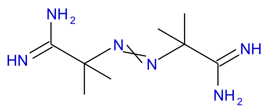	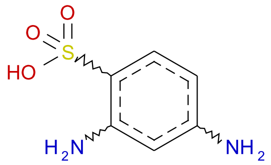
Actual Endpoint	Mild	Mild	Moderate_Severe
Predicted Endpoint	Mild	Mild	Mild
Distance	0.762	0.818	0.821
Reference	85JCAE "Prehled Prumyslove Toxikologie; Organicke Latky," Marhold, J., Prague , Czechoslovakia, Avicenum, 1986 Volume(issue)/page/year: -,658,1986	EPASR* United States Environmental Protection Agency, Office of Pesticides an d Toxic Substances. (U.S. Environmental Protection Agency, 401 M St., SW, Washin gton, DC 20460) History unknown. Volume(issue)/pag	28ZPAK "Sbornik Vysledku Toxixologickeho Vysetreni Latek A Pripravku," Marhol d, J.V., Institut Pro Vychovu Vedoucicn Pracovniku Chemickeho Prumyclu Praha, Cz echoslovakia, 1972 Volume(issue)/page/year: -,180,1

Model Prediction

Prediction: Mild
Probability: 0.131
Enrichment: 0.356
Bayesian Score: -7.203
Mahalanobis Distance: 13.737
Mahalanobis Distance p-value: 1.84e-010

Prediction: Positive if the Bayesian score is above the estimated best cutoff value from minimizing the false positive and false negative rate.
Probability: The esimated probability that the sample is in the positive category. This assumes that the Bayesian score follows a normal distribution and is different from the prediction using a cutoff.
Enrichment: An estimate of enrichment, that is, the increased likelihood (versus random) of this sample being in the category.
Bayesian Score: The standard Laplacian-modified Bayesian score.
Mahalanobis Distance: The Mahalanobis distance (MD) is the distance to the center of the training data. The larger the MD, the less trustworthy the prediction.
Mahalanobis Distance p-value: The p-value gives the fraction of training data with an MD greater than or equal to the one for the given sample, assuming normally distributed data. The smaller the p-value, the less trustworthy the prediciton. For highly non-normal X properties (e.g., fingerprints), the MD p-value is wildly inaccurate.

Model Applicability

Unknown features are fingerprint features in the query molecule, but not found in the training set.

1. All properties and OPS components are within expected ranges.

Feature Contribution

Top features for positive contribution				
Fingerprint	Bit/Smiles	Feature Structure	Score	Moderate_Severe in training set

FCFP_12	699500266	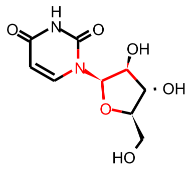 [*][C@H]1[*][*]O[C@H]1N([*])[*]	0.441	14 out of 24
FCFP_12	-2091721556	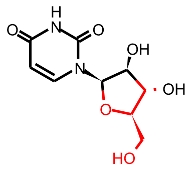 [*][C@H]1[*][*]O[C@@H]1CO	0.365	2 out of 3
FCFP_12	451847724	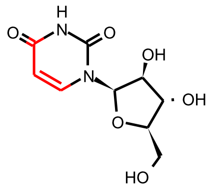 [*]\C=C/C(=[*])[*]	0.257	101 out of 216

Top Features for negative contribution

Fingerprint	Bit/Smiles	Feature Structure	Score	Moderate_Severe in training set
FCFP_12	-885550502	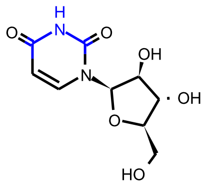 [*]C(=[*])NC(=[*])[*]	-1.050	2 out of 21
FCFP_12	566058135	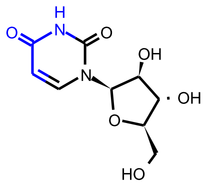 [*]NC(=O)C(=[*])[*]	-0.909	1 out of 11

FCFP_12	-1986098826	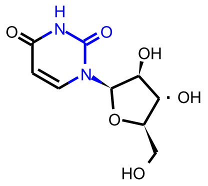[*]NC(=O)N([*])[*]	-0.733	0 out of 3

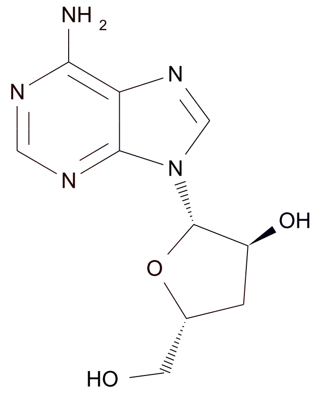

$C_{10}H_{13}N_5O_3$
Molecular Weight: 251.24191
ALogP: -1.308
Rotatable Bonds: 2
Acceptors: 7
Donors: 3

Model Prediction

Prediction: Mild
Probability: 0.208
Enrichment: 0.566
Bayesian Score: -4.933
Mahalanobis Distance: 9.932
Mahalanobis Distance p-value: 0.0366

Prediction: Positive if the Bayesian score is above the estimated best cutoff value from minimizing the false positive and false negative rate.
Probability: The esimated probability that the sample is in the positive category. This assumes that the Bayesian score follows a normal distribution and is different from the prediction using a cutoff.
Enrichment: An estimate of enrichment, that is, the increased likelihood (versus random) of this sample being in the category.
Bayesian Score: The standard Laplacian-modified Bayesian score.
Mahalanobis Distance: The Mahalanobis distance (MD) is the distance to the center of the training data. The larger the MD, the less trustworthy the prediction.
Mahalanobis Distance p-value: The p-value gives the fraction of training data with an MD greater than or equal to the one for the given sample, assuming normally distributed data. The smaller the p-value, the less trustworthy the prediciton. For highly non-normal X properties (e.g., fingerprints), the MD p-value is wildly inaccurate.

Structural Similar Compounds

Name	C.I. Fluorescent Brightening Agent 24	Glutamic acid, N-(p-(methylamino)benzoyl)-, sodium salt	Benzene-1,3-dicarboxylic acid, 5-sulfo-
Structure	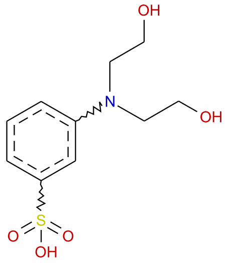	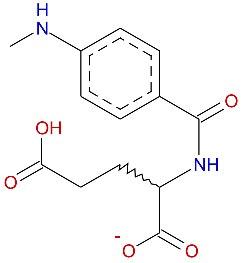	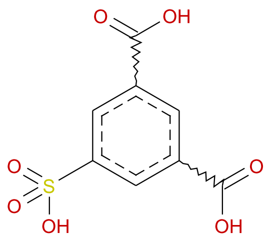
Actual Endpoint	Mild	Mild	Mild
Predicted Endpoint	Mild	Mild	Mild
Distance	0.734	0.738	0.741
Reference	MVCRB3 MVC-Report. (Stockholm, Sweden) No.1-2, 1972-73. Discontinued. Volu me(issue)/page/year: 2,193,1973	FCTOD7 Food and Chemical Toxicology. (Pergamon Press Inc., Maxwell House, Fa irview Park, Elmsford, NY 10523) V.20- 1982- Volume(issue)/page/year: 20,563 ,1982	85JCAE "Prehled Prumyslove Toxikologie; Organicke Latky," Marhold, J., Prague , Czechoslovakia, Avicenum, 1986 Volume(issue)/page/year: -,1056,1986

Model Applicability

Unknown features are fingerprint features in the query molecule, but not found in the training set.

1. All properties and OPS components are within expected ranges.
2. Unknown FCFP_2 feature: -1564473960: [*]n1:[*]:[*]:[c](:[*]):[c]:1:n:[*]
3. Unknown FCFP_2 feature: 1315416442: [*][C@H]1[*][*]O[C@H]1n(:[*]):[*]

Feature Contribution

Top features for positive contribution

Fingerprint	Bit/Smiles	Feature Structure	Score	Moderate_Severe in training set

FCFP_12	76292238	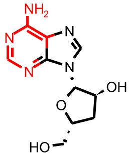 [*]:[c]1:[*]:n:[cH]:n:[c]:1N	0.385	1 out of 1
FCFP_12	-1151884458	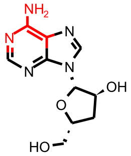 [*]:n:[c](N):[c](:[*]):[*]	0.385	1 out of 1
FCFP_12	-2091721556	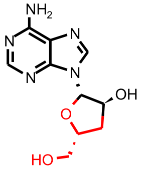 [*][C@H]1[*][*]O[C@@H]1CO	0.365	2 out of 3

Top Features for negative contribution

Fingerprint	Bit/Smiles	Feature Structure	Score	Moderate_Severe in training set
FCFP_12	-1272709286	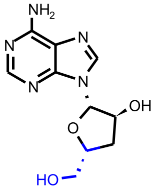 [*]C([*])CO	-0.475	18 out of 82
FCFP_12	178336375	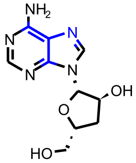 [*][c](:[*]):[c]1:n:[*]:[*]:[c]:1:[*]	-0.413	3 out of 14

FCFP_12	1069584379	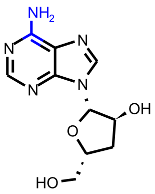[*]:[c](:[*])N	-0.360	8 out of 33

Ribavirin

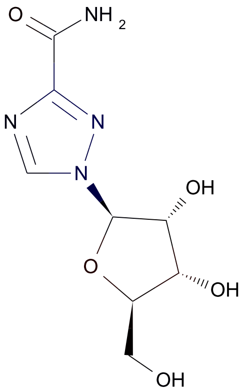

$C_8H_{12}N_4O_5$
Molecular Weight: 244.20467
ALogP: -2.745
Rotatable Bonds: 3
Acceptors: 7
Donors: 4

Structural Similar Compounds

Name	Citric acid	p-Benzenedisulfonic acid, 2-amino-	Benzene-1,3-dicarboxylic acid, 5-sulfo-
Structure	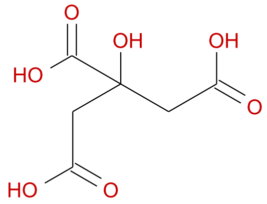	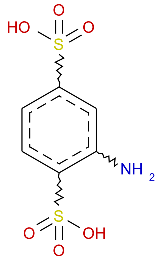	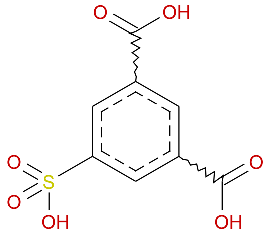
Actual Endpoint	Mild	Mild	Mild
Predicted Endpoint	Mild	Mild	Mild
Distance	0.675	0.793	0.811
Reference	85JCAE "Prehled Prumyslove Toxikologie; Organicke Latky," Marhold, J., Prague , Czechoslovakia, Avicenum, 1986 Volume(issue)/page/year: -,658,1986	85JCAE "Prehled Prumyslove Toxikologie; Organicke Latky," Marhold, J., Prague , Czechoslovakia, Avicenum, 1986 Volume(issue)/page/year: -,1052,1986	85JCAE "Prehled Prumyslove Toxikologie; Organicke Latky," Marhold, J., Prague , Czechoslovakia, Avicenum, 1986 Volume(issue)/page/year: -,1056,1986

Model Prediction

Prediction: Mild
Probability: 0.157
Enrichment: 0.425
Bayesian Score: -6.393
Mahalanobis Distance: 10.379
Mahalanobis Distance p-value: 0.00931

Prediction: Positive if the Bayesian score is above the estimated best cutoff value from minimizing the false positive and false negative rate.
Probability: The esimated probability that the sample is in the positive category. This assumes that the Bayesian score follows a normal distribution and is different from the prediction using a cutoff.
Enrichment: An estimate of enrichment, that is, the increased likelihood (versus random) of this sample being in the category.
Bayesian Score: The standard Laplacian-modified Bayesian score.
Mahalanobis Distance: The Mahalanobis distance (MD) is the distance to the center of the training data. The larger the MD, the less trustworthy the prediction.
Mahalanobis Distance p-value: The p-value gives the fraction of training data with an MD greater than or equal to the one for the given sample, assuming normally distributed data. The smaller the p-value, the less trustworthy the prediciton. For highly non-normal X properties (e.g., fingerprints), the MD p-value is wildly inaccurate.

Model Applicability

Unknown features are fingerprint features in the query molecule, but not found in the training set.

1. All properties and OPS components are within expected ranges.
2. Unknown FCFP_2 feature: 1315416442: [*][C@H]1[*][*]O[C@H]1n(:[*]):[*]

Feature Contribution

Top features for positive contribution

Fingerprint	Bit/Smiles	Feature Structure	Score	Moderate_Severe in training set
FCFP_12	-2091721556	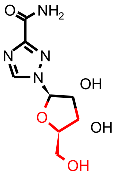 [*][C@H]1[*][*]O[C@@H]1CO	0.365	2 out of 3

FCFP_12	-1549103449	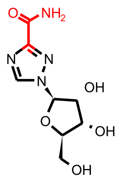<br>[*]:[c](:[*])C(=O)N	0.206	2 out of 4
FCFP_12	-124685461	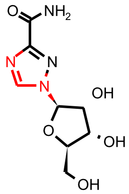<br>[*]:n:[cH]:n:[*]	0.206	2 out of 4
**Top Features for negative contribution**				
**Fingerprint**	**Bit/Smiles**	**Feature Structure**	**Score**	**Moderate_Severe in training set**
FCFP_12	4427049	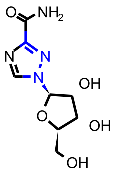<br>[*]n1:[*]:[*]:[c]([*]<br>):n:1	-0.893	0 out of 4
FCFP_12	-1272709286	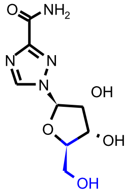<br>[*]C([*])CO	-0.475	18 out of 82
FCFP_12	17	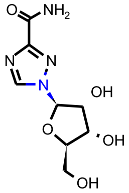<br>[*]:n:[*]	-0.332	8 out of 32

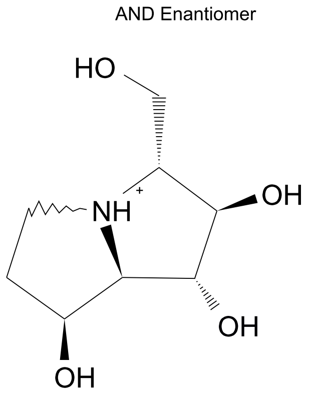

$C_8H_{16}NO_4$
Molecular Weight: 190.21693
ALogP: -3.556
Rotatable Bonds: 1
Acceptors: 4
Donors: 5

Model Prediction

Prediction: Irritant
Probability: 0.973
Enrichment: 1.057
Bayesian Score: -0.731
Mahalanobis Distance: 10.663
Mahalanobis Distance p-value: 0.00854

Prediction: Positive if the Bayesian score is above the estimated best cutoff value from minimizing the false positive and false negative rate.
Probability: The esimated probability that the sample is in the positive category. This assumes that the Bayesian score follows a normal distribution and is different from the prediction using a cutoff.
Enrichment: An estimate of enrichment, that is, the increased likelihood (versus random) of this sample being in the category.
Bayesian Score: The standard Laplacian-modified Bayesian score.
Mahalanobis Distance: The Mahalanobis distance (MD) is the distance to the center of the training data. The larger the MD, the less trustworthy the prediction.
Mahalanobis Distance p-value: The p-value gives the fraction of training data with an MD greater than or equal to the one for the given sample, assuming normally distributed data. The smaller the p-value, the less trustworthy the prediciton. For highly non-normal X properties (e.g., fingerprints), the MD p-value is wildly inaccurate.

Structural Similar Compounds

Name	2-Imidazolidinone, 1,3-bis(hydroxymethyl)-4,5-dihydroxy-	Triethylenetetramine	1,2-Ethanediamine, N-(2-aminoethyl)-N'-(2-((2-aminoethyl)amino)ethy l)-
Structure	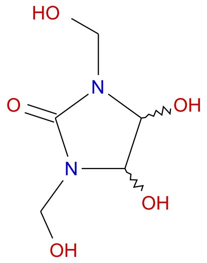	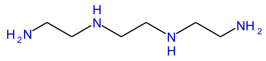	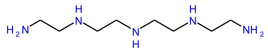
Actual Endpoint	Irritant	Irritant	Irritant
Predicted Endpoint	Irritant	Irritant	Irritant
Distance	0.771	0.828	0.895
Reference	28ZPAK "Sbornik Vysledku Toxixologickeho Vysetreni Latek A Pripravku," Marhol d, J.V., Institut Pro Vychovu Vedoucicn Pracovniku Chemickeho Prumyclu Praha, Cz echoslovakia, 1972 Volume(issue)/page/year: -,269,1	85JCAE "Prehled Prumyslove Toxikologie; Organicke Latky," Marhold, J., Prague , Czechoslovakia, Avicenum, 1986 Volume(issue)/page/year: -,443,1986	85JCAE "Prehled Prumyslove Toxikologie; Organicke Latky," Marhold, J., Prague , Czechoslovakia, Avicenum, 1986 Volume(issue)/page/year: -,446,1986

Model Applicability

Unknown features are fingerprint features in the query molecule, but not found in the training set.

1. All properties and OPS components are within expected ranges.
2. Unknown FCFP_2 feature: -1853714334: [*][C@@H]1[*][*][C@H]2[*][*]C[NH+]12
3. Unknown FCFP_2 feature: -1817836174: [*][C@H]1[*][*][NH+]2[*][*][C@H]([*])C12
4. Unknown FCFP_2 feature: 1155241219: [*][NH+]1[*][*]CC1

Feature Contribution

Top Features for negative contribution				
Fingerprint	Bit/Smiles	Feature Structure	Score	Irritant in training set

FCFP_12	3	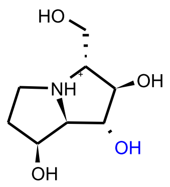<br>[*]O	-0.081	291 out of 345
FCFP_12	-1272798659	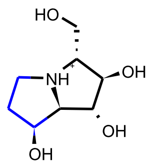<br>[*][C@@H]1[*][*]CC1	0.000	630 out of 658
FCFP_12	-1043250487	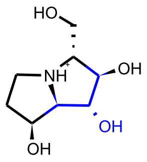<br>[*][C@@H]1[*][*][C@H]<br>([*])[C@H]1O	0.000	55 out of 61

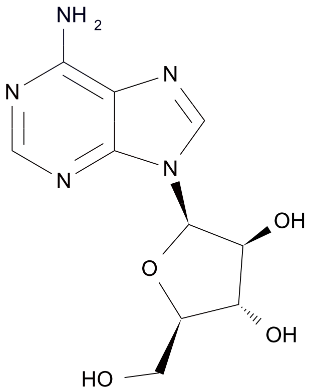

$C_{10}H_{13}N_5O_4$
Molecular Weight: 267.24132
ALogP: -1.881
Rotatable Bonds: 2
Acceptors: 8
Donors: 4

Model Prediction

Prediction: Irritant
Probability: 0.974
Enrichment: 1.058
Bayesian Score: -0.690
Mahalanobis Distance: 10.365
Mahalanobis Distance p-value: 0.0225

Prediction: Positive if the Bayesian score is above the estimated best cutoff value from minimizing the false positive and false negative rate.
Probability: The esimated probability that the sample is in the positive category. This assumes that the Bayesian score follows a normal distribution and is different from the prediction using a cutoff.
Enrichment: An estimate of enrichment, that is, the increased likelihood (versus random) of this sample being in the category.
Bayesian Score: The standard Laplacian-modified Bayesian score.
Mahalanobis Distance: The Mahalanobis distance (MD) is the distance to the center of the training data. The larger the MD, the less trustworthy the prediction.
Mahalanobis Distance p-value: The p-value gives the fraction of training data with an MD greater than or equal to the one for the given sample, assuming normally distributed data. The smaller the p-value, the less trustworthy the prediciton. For highly non-normal X properties (e.g., fingerprints), the MD p-value is wildly inaccurate.

Structural Similar Compounds

Name	Citric acid	Benzene-1,3-dicarboxylic acid, 5-sulfo-	2,2'-Benzidine disulfonic acid
Structure	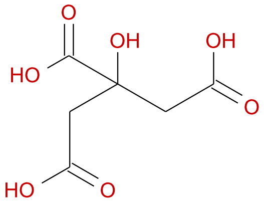	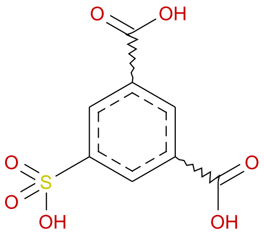	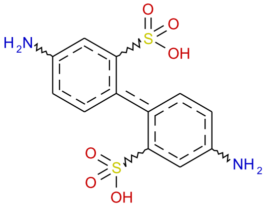
Actual Endpoint	Irritant	Irritant	Non-Irritant
Predicted Endpoint	Irritant	Non-Irritant	Non-Irritant
Distance	0.771	0.804	0.808
Reference	85JCAE "Prehled Prumyslove Toxikologie; Organicke Latky," Marhold, J., Prague , Czechoslovakia, Avicenum, 1986 Volume(issue)/page/year: -,658,1986	85JCAE "Prehled Prumyslove Toxikologie; Organicke Latky," Marhold, J., Prague , Czechoslovakia, Avicenum, 1986 Volume(issue)/page/year: -,1056,1986	28ZPAK -,191,72

Model Applicability

Unknown features are fingerprint features in the query molecule, but not found in the training set.

1. All properties and OPS components are within expected ranges.

Feature Contribution

Top features for positive contribution				
Fingerprint	Bit/Smiles	Feature Structure	Score	Irritant in training set
FCFP_12	-124685461	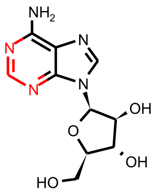 [*]:n:[cH]:n:[*]	0.073	5 out of 5

FCFP_12	-475316933	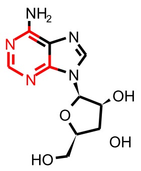 [*][c]1:[*]:[c](:[*]) :n:[cH]:n:1	0.070	4 out of 4
FCFP_12	-2091721556	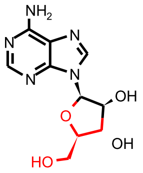 [*][C@H]1[*][*]O[C@@H]1CO	0.070	4 out of 4

Top Features for negative contribution

Fingerprint	Bit/Smiles	Feature Structure	Score	Irritant in training set
FCFP_12	1069584379	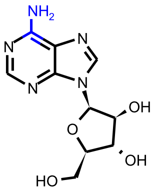 [*]:[c](:[*])N	-0.439	38 out of 65
FCFP_12	16	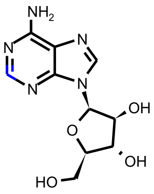 [*]:[cH]:[*]	-0.084	423 out of 503
FCFP_12	3	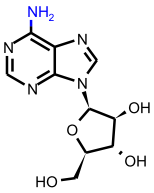 [*]O	-0.081	291 out of 345

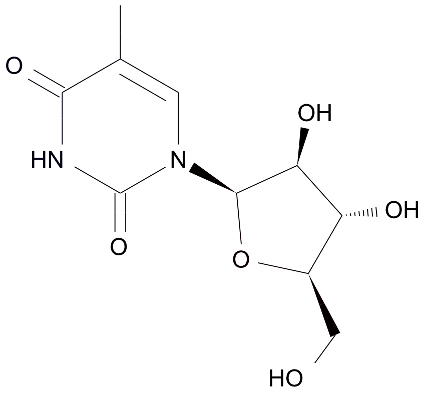

$C_{10}H_{14}N_2O_6$
Molecular Weight: 258.22795
ALogP: -2.005
Rotatable Bonds: 2
Acceptors: 6
Donors: 4

Model Prediction

Prediction: Irritant
Probability: 0.975
Enrichment: 1.058
Bayesian Score: -0.618
Mahalanobis Distance: 14.458
Mahalanobis Distance p-value: 2.7e-012

Prediction: Positive if the Bayesian score is above the estimated best cutoff value from minimizing the false positive and false negative rate.
Probability: The esimated probability that the sample is in the positive category. This assumes that the Bayesian score follows a normal distribution and is different from the prediction using a cutoff.
Enrichment: An estimate of enrichment, that is, the increased likelihood (versus random) of this sample being in the category.
Bayesian Score: The standard Laplacian-modified Bayesian score.
Mahalanobis Distance: The Mahalanobis distance (MD) is the distance to the center of the training data. The larger the MD, the less trustworthy the prediction.
Mahalanobis Distance p-value: The p-value gives the fraction of training data with an MD greater than or equal to the one for the given sample, assuming normally distributed data. The smaller the p-value, the less trustworthy the prediciton. For highly non-normal X properties (e.g., fingerprints), the MD p-value is wildly inaccurate.

Structural Similar Compounds

Name	2-Imidazolidinone, 1,3-bis(hydroxymethyl)-4,5-dihydroxy-	Anthraquinone, 1,5-diamino-4,8-dihydroxy-	Propionamidine, 2,2'-azobis(2-methyl-, dihydrochloride
Structure	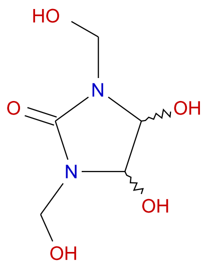	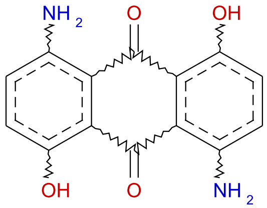	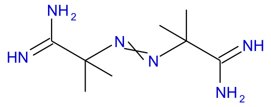
Actual Endpoint	Irritant	Non-Irritant	Irritant
Predicted Endpoint	Irritant	Non-Irritant	Irritant
Distance	0.680	0.755	0.792
Reference	28ZPAK "Sbornik Vysledku Toxixologickeho Vysetreni Latek A Pripravku," Marhol d, J.V., Institut Pro Vychovu Vedoucicn Pracovniku Chemickeho Prumyclu Praha, Cz echoslovakia, 1972 Volume(issue)/page/year: -,269,1	28ZPAK -,103,72	EPASR* United States Environmental Protection Agency, Office of Pesticides an d Toxic Substances. (U.S. Environmental Protection Agency, 401 M St., SW, Washin gton, DC 20460) History unknown. Volume(issue)/pag

Model Applicability

Unknown features are fingerprint features in the query molecule, but not found in the training set.

1. All properties and OPS components are within expected ranges.

Feature Contribution

Top features for positive contribution				
**Fingerprint**	**Bit/Smiles**	**Feature Structure**	**Score**	**Irritant in training set**

FCFP_12	699500266	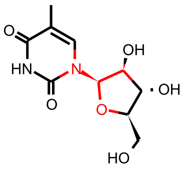 [*][C@H]1[*][*]O[C@H]1N([*])[*]	0.085	25 out of 25
FCFP_12	451877515	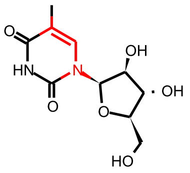 [*]N([*])C=C([*])[*]	0.082	13 out of 13
FCFP_12	436886043	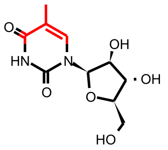 [*]\C=C(\C)/C(=[*])[*]	0.080	129 out of 130
**Top Features for negative contribution**				
**Fingerprint**	**Bit/Smiles**	**Feature Structure**	**Score**	**Irritant in training set**
FCFP_12	566058135	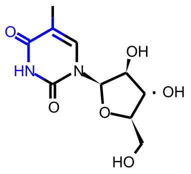 [*]NC(=O)C(=[*])[*]	-0.367	13 out of 21
FCFP_12	3	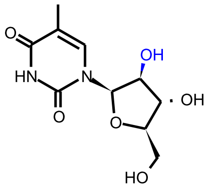 [*]O	-0.081	291 out of 345

FCFP_12	-1986098826	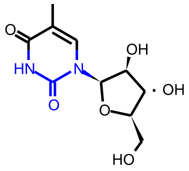 [*]NC(=O)N([*])[*]	-0.079	5 out of 6

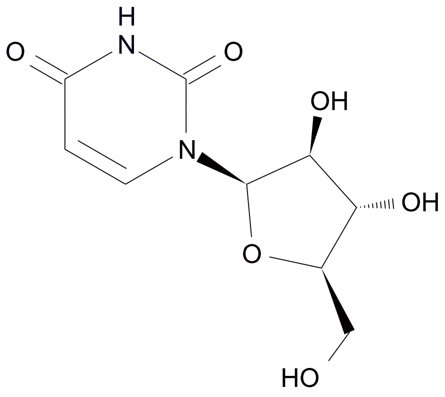

$C_9H_{12}N_2O_6$
Molecular Weight: 244.20137
ALogP: -2.451
Rotatable Bonds: 2
Acceptors: 6
Donors: 4

Model Prediction

Prediction: Irritant
Probability: 0.974
Enrichment: 1.058
Bayesian Score: -0.671
Mahalanobis Distance: 13.834
Mahalanobis Distance p-value: 2.98e-010

Prediction: Positive if the Bayesian score is above the estimated best cutoff value from minimizing the false positive and false negative rate.
Probability: The esimated probability that the sample is in the positive category. This assumes that the Bayesian score follows a normal distribution and is different from the prediction using a cutoff.
Enrichment: An estimate of enrichment, that is, the increased likelihood (versus random) of this sample being in the category.
Bayesian Score: The standard Laplacian-modified Bayesian score.
Mahalanobis Distance: The Mahalanobis distance (MD) is the distance to the center of the training data. The larger the MD, the less trustworthy the prediction.
Mahalanobis Distance p-value: The p-value gives the fraction of training data with an MD greater than or equal to the one for the given sample, assuming normally distributed data. The smaller the p-value, the less trustworthy the prediciton. For highly non-normal X properties (e.g., fingerprints), the MD p-value is wildly inaccurate.

Structural Similar Compounds

Name	2-Imidazolidinone, 1,3-bis(hydroxymethyl)-4,5-dihydroxy-	Citric acid	Anthraquinone, 1,5-diamino-4,8-dihydroxy-
Structure	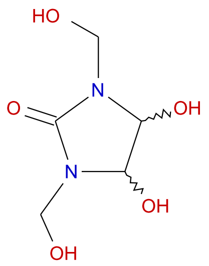	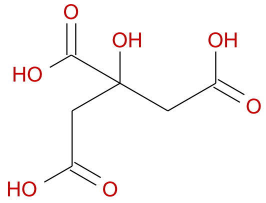	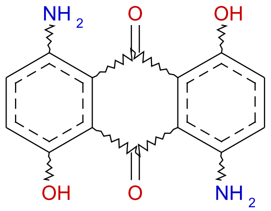
Actual Endpoint	Irritant	Irritant	Non-Irritant
Predicted Endpoint	Irritant	Irritant	Non-Irritant
Distance	0.636	0.765	0.795
Reference	28ZPAK "Sbornik Vysledku Toxixologickeho Vysetreni Latek A Pripravku," Marhol d, J.V., Institut Pro Vychovu Vedoucicn Pracovniku Chemickeho Prumyclu Praha, Cz echoslovakia, 1972 Volume(issue)/page/year: -,269,1	85JCAE "Prehled Prumyslove Toxikologie; Organicke Latky," Marhold, J., Prague , Czechoslovakia, Avicenum, 1986 Volume(issue)/page/year: -,658,1986	28ZPAK -,103,72

Model Applicability

Unknown features are fingerprint features in the query molecule, but not found in the training set.

1. All properties and OPS components are within expected ranges.

Feature Contribution

Top features for positive contribution				
Fingerprint	Bit/Smiles	Feature Structure	Score	Irritant in training set

FCFP_12	699500266	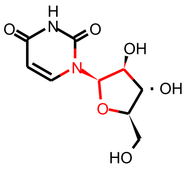 [*][C@H]1[*][*]O[C@H]1N([*])[*]	0.085	25 out of 25
FCFP_12	451877515	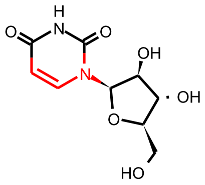 [*]N([*])C=C([*])[*]	0.082	13 out of 13
FCFP_12	451847724	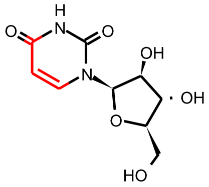 [*]\C=C/C(=[*])[*]	0.074	270 out of 274
**Top Features for negative contribution**				
**Fingerprint**	**Bit/Smiles**	**Feature Structure**	**Score**	**Irritant in training set**
FCFP_12	566058135	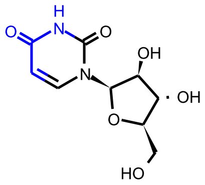 [*]NC(=O)C(=[*])[*]	-0.367	13 out of 21
FCFP_12	3	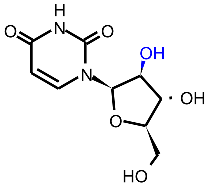 [*]O	-0.081	291 out of 345

FCFP_12	-1986098826	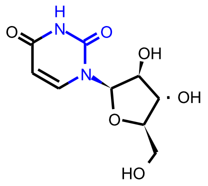 [*]NC(=O)N([*])[*]	-0.079	5 out of 6

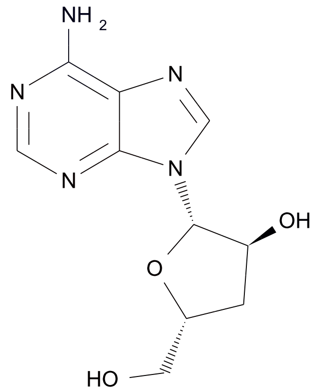

$C_{10}H_{13}N_5O_3$
Molecular Weight: 251.24191
ALogP: -1.308
Rotatable Bonds: 2
Acceptors: 7
Donors: 3

Model Prediction

Prediction: Irritant
Probability: 0.974
Enrichment: 1.058
Bayesian Score: -0.690
Mahalanobis Distance: 9.741
Mahalanobis Distance p-value: 0.117

Prediction: Positive if the Bayesian score is above the estimated best cutoff value from minimizing the false positive and false negative rate.
Probability: The esimated probability that the sample is in the positive category. This assumes that the Bayesian score follows a normal distribution and is different from the prediction using a cutoff.
Enrichment: An estimate of enrichment, that is, the increased likelihood (versus random) of this sample being in the category.
Bayesian Score: The standard Laplacian-modified Bayesian score.
Mahalanobis Distance: The Mahalanobis distance (MD) is the distance to the center of the training data. The larger the MD, the less trustworthy the prediction.
Mahalanobis Distance p-value: The p-value gives the fraction of training data with an MD greater than or equal to the one for the given sample, assuming normally distributed data. The smaller the p-value, the less trustworthy the prediciton. For highly non-normal X properties (e.g., fingerprints), the MD p-value is wildly inaccurate.

Structural Similar Compounds

Name	C.I. Fluorescent Brightening Agent 24	Benzene-1,3-dicarboxylic acid, 5-sulfo-	Glutamic acid, N-(p-(methylamino)benzoyl)-, sodium salt
Structure	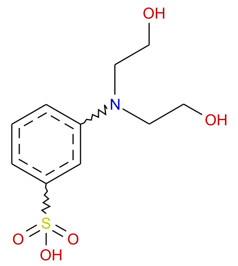	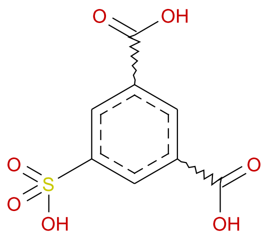	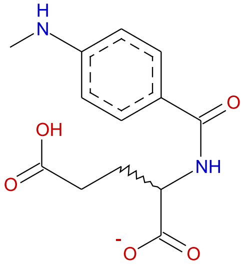
Actual Endpoint	Irritant	Irritant	Irritant
Predicted Endpoint	Non-Irritant	Non-Irritant	Non-Irritant
Distance	0.740	0.741	0.744
Reference	MVCRB3 MVC-Report. (Stockholm, Sweden) No.1-2, 1972-73. Discontinued. Volu me(issue)/page/year: 2,193,1973	85JCAE "Prehled Prumyslove Toxikologie; Organicke Latky," Marhold, J., Prague , Czechoslovakia, Avicenum, 1986 Volume(issue)/page/year: -,1056,1986	FCTOD7 Food and Chemical Toxicology. (Pergamon Press Inc., Maxwell House, Fa irview Park, Elmsford, NY 10523) V.20- 1982- Volume(issue)/page/year: 20,563 ,1982

Model Applicability

Unknown features are fingerprint features in the query molecule, but not found in the training set.

1. All properties and OPS components are within expected ranges.

Feature Contribution

Top features for positive contribution

Fingerprint	Bit/Smiles	Feature Structure	Score	Irritant in training set
FCFP_12	-124685461	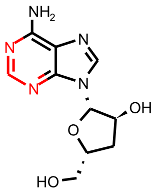 [*]:n:[cH]:n:[*]	0.073	5 out of 5

FCFP_12	-2091721556	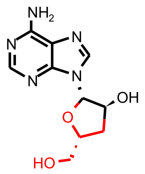 [*][C@H]1[*][*]O[C@@H]1CO	0.070	4 out of 4
FCFP_12	-475316933	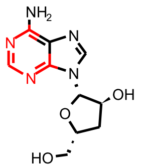 [*][c]1:[*]:[c](:[*]) :n:[cH]:n:1	0.070	4 out of 4
**Top Features for negative contribution**				
**Fingerprint**	**Bit/Smiles**	**Feature Structure**	**Score**	**Irritant in training set**
FCFP_12	1069584379	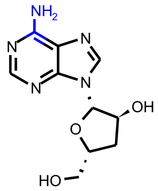 [*]:[c](:[*])N	-0.439	38 out of 65
FCFP_12	16	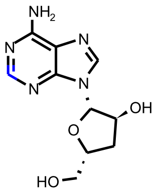 [*]:[cH]:[*]	-0.084	423 out of 503
FCFP_12	3	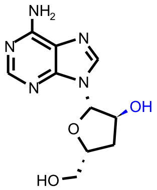 [*]O	-0.081	291 out of 345

Ribavirin

TOPKAT_Skin_Irritancy_None_vs_Irritant

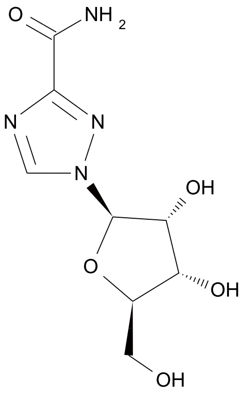

$C_8H_{12}N_4O_5$

Molecular Weight: 244.20467

ALogP: -2.745

Rotatable Bonds: 3

Acceptors: 7

Donors: 4

Model Prediction

Prediction: Irritant

Probability: 0.977

Enrichment: 1.061

Bayesian Score: -0.345

Mahalanobis Distance: 10.393

Mahalanobis Distance p-value: 0.0206

Prediction: Positive if the Bayesian score is above the estimated best cutoff value from minimizing the false positive and false negative rate.
Probability: The esimated probability that the sample is in the positive category. This assumes that the Bayesian score follows a normal distribution and is different from the prediction using a cutoff.
Enrichment: An estimate of enrichment, that is, the increased likelihood (versus random) of this sample being in the category.
Bayesian Score: The standard Laplacian-modified Bayesian score.
Mahalanobis Distance: The Mahalanobis distance (MD) is the distance to the center of the training data. The larger the MD, the less trustworthy the prediction.
Mahalanobis Distance p-value: The p-value gives the fraction of training data with an MD greater than or equal to the one for the given sample, assuming normally distributed data. The smaller the p-value, the less trustworthy the prediciton. For highly non-normal X properties (e.g., fingerprints), the MD p-value is wildly inaccurate.

Structural Similar Compounds

**Name**	**Citric acid**	**2-Imidazolidinone, 1,3-bis(hydroxymethyl)-4,5-dihydroxy-**	**p-Benzenedisulfonic acid, 2-amino-**
Structure	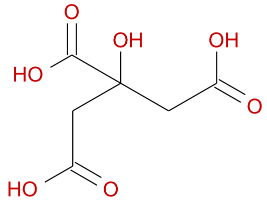	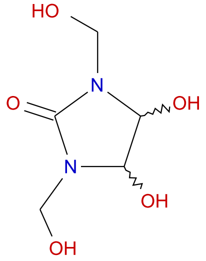	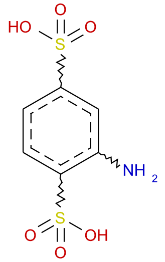
Actual Endpoint	Irritant	Irritant	Irritant
Predicted Endpoint	Irritant	Irritant	Non-Irritant
Distance	0.679	0.782	0.789
Reference	85JCAE "Prehled Prumyslove Toxikologie; Organicke Latky," Marhold, J., Prague , Czechoslovakia, Avicenum, 1986 Volume(issue)/page/year: -,658,1986	28ZPAK "Sbornik Vysledku Toxixologickeho Vysetreni Latek A Pripravku," Marhol d, J.V., Institut Pro Vychovu Vedoucicn Pracovniku Chemickeho Prumyclu Praha, Cz echoslovakia, 1972 Volume(issue)/page/year: -,269,1	85JCAE "Prehled Prumyslove Toxikologie; Organicke Latky," Marhold, J., Prague , Czechoslovakia, Avicenum, 1986 Volume(issue)/page/year: -,1052,1986

Model Applicability

Unknown features are fingerprint features in the query molecule, but not found in the training set.

1. All properties and OPS components are within expected ranges.

Feature Contribution

Top features for positive contribution

Fingerprint	Bit/Smiles	Feature Structure	Score	Irritant in training set

FCFP_12	-1549103449	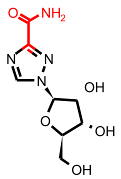 [*]:[c](:[*])C(=O)N	0.073	5 out of 5
FCFP_12	-124685461	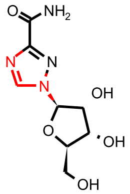 [*]:n:[cH]:n:[*]	0.073	5 out of 5
FCFP_12	4427049	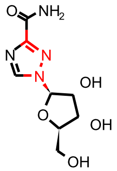 [*]n1:[*]:[*]:[c]([*]):n:1	0.073	5 out of 5
**Top Features for negative contribution**				
**Fingerprint**	**Bit/Smiles**	**Feature Structure**	**Score**	**Irritant in training set**
FCFP_12	16	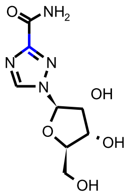 [*]:[cH]:[*]	-0.084	423 out of 503
FCFP_12	3	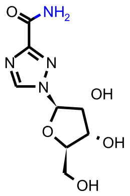 [*]O	-0.081	291 out of 345

FCFP_12	1070061035	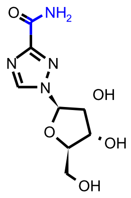 [*]C([*])O	0.000	215 out of 237

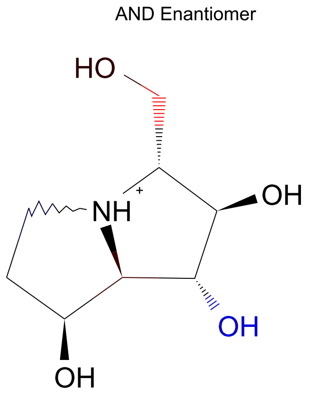

$C_8H_{16}NO_4$
Molecular Weight: 190.21693
ALogP: -3.556
Rotatable Bonds: 1
Acceptors: 4
Donors: 5

Model Prediction

Prediction: 9.294
Unit: mg/kg_body_weight/day
Mahalanobis Distance: 8.835
Mahalanobis Distance p-value: 0.365

Mahalanobis Distance: The Mahalanobis distance (MD) is a generalization of the Euclidean distance that accounts for correlations among the X properties. It is calculated as the distance to the center of the training data. The larger the MD, the less trustworthy the prediction.
Mahalanobis Distance p-value: The p-value gives the fraction of training data with an MD greater than or equal to the one for the given sample, assuming normally distributed data. The smaller the p-value, the less trustworthy the prediciton. For highly non-normal X properties (e.g., fingerprints), the MD p-value is wildly inaccurate.

Structural Similar Compounds

Name	5-Azacytidine	720	2-Hydroxyethylhydrazine s
Structure	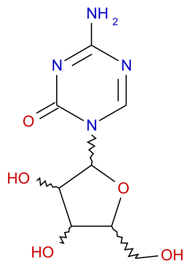	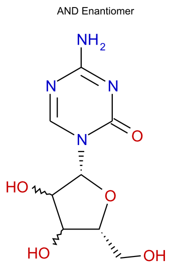	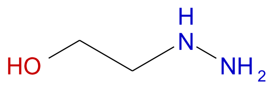
Actual Endpoint (-log C)	6.49901	6.49901	5.28258
Predicted Endpoint (-log C)	4.14504	4.14504	3.70898
Distance	0.846	0.846	0.874
Reference	CPDB	CPDB	CPDB

Model Applicability

Unknown features are fingerprint features in the query molecule, but not found in the training set.

1. All properties and OPS components are within expected ranges.
2. Unknown ECFP_2 feature: 1976330679: [*][NH+]([*])[*]
3. Unknown ECFP_2 feature: -1693770146: [*][C@@H]1[*][*][C@H]2[*][*]C[NH+]12
4. Unknown ECFP_2 feature: -1395316370: [*][C@H]1[*][*][NH+]2[*][*][C@H]([*])C12
5. Unknown ECFP_2 feature: -705668329: [*]C[C@@H]1[C@@H]([*])[*][*][NH+]1[*]
6. Unknown ECFP_2 feature: -244159614: [*][NH+]1[*][*]CC1

Feature Contribution

Top features for positive contribution			
Fingerprint	Bit/Smiles	Feature Structure	Score
ECFP_6	1559650422	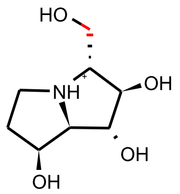 [*]C[*]	0.203

ECFP_6	-167460056	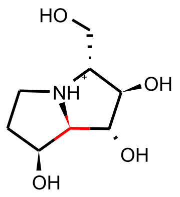<br>[*]C([*])[*]	0.060
ECFP_6	2022454958	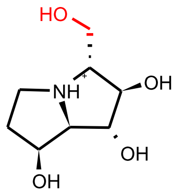<br>[*]CO	0.045
**Top Features for negative contribution**			
**Fingerprint**	**Bit/Smiles**	**Feature Structure**	**Score**
ECFP_6	-1884411803	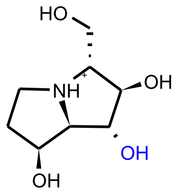<br>[*]O	-0.217
ECFP_6	-992506539	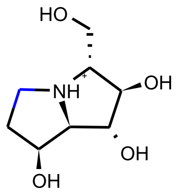<br>[*]C[*]	-0.085

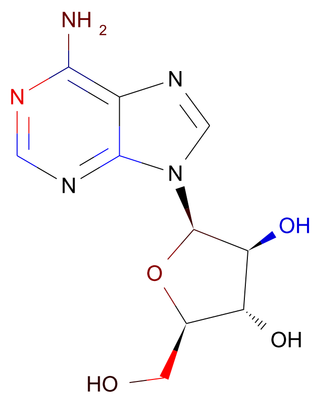

$C_{10}H_{13}N_5O_4$

Molecular Weight: 267.24132

ALogP: -1.881

Rotatable Bonds: 2

Acceptors: 8

Donors: 4

Model Prediction

Prediction: 4.245

Unit: mg/kg_body_weight/day

Mahalanobis Distance: 8.944

Mahalanobis Distance p-value: 0.314

Mahalanobis Distance: The Mahalanobis distance (MD) is a generalization of the Euclidean distance that accounts for correlations among the X properties. It is calculated as the distance to the center of the training data. The larger the MD, the less trustworthy the prediction.
Mahalanobis Distance p-value: The p-value gives the fraction of training data with an MD greater than or equal to the one for the given sample, assuming normally distributed data. The smaller the p-value, the less trustworthy the prediciton. For highly non-normal X properties (e.g., fingerprints), the MD p-value is wildly inaccurate.

Structural Similar Compounds

Name	(N-6)-(Methylnitroso)adenosine	377	720
Structure	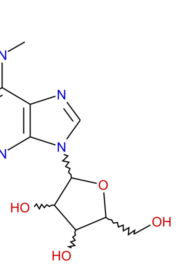	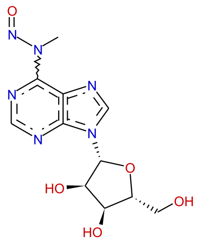 AND Enantiomer	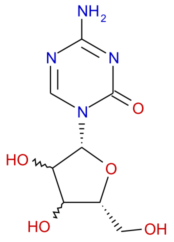 AND Enantiomer
Actual Endpoint (-log C)	4.22928	4.22928	6.49901
Predicted Endpoint (-log C)	5.36013	5.36013	4.14504
Distance	0.413	0.413	0.726
Reference	CPDB	CPDB	CPDB

Model Applicability

Unknown features are fingerprint features in the query molecule, but not found in the training set.

1. OPS PC13 out of range. Value: -3.2678. Training min, max, SD, explained variance: -3.068, 3.6909, 1.329, 0.0220.

Feature Contribution

Top features for positive contribution			
**Fingerprint**	**Bit/Smiles**	**Feature Structure**	**Score**
ECFP_6	655739385	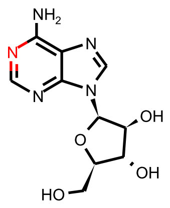 [*]:n:[*]	0.229

ECFP_6	1572579716	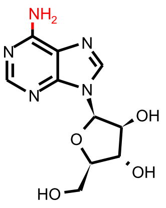 [*]N	0.225
ECFP_6	1559650422	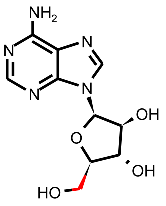 [*]C[*]	0.203

Top Features for negative contribution

Fingerprint	Bit/Smiles	Feature Structure	Score
ECFP_6	642810091	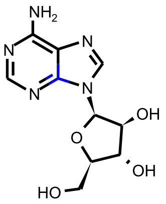 [*]:[c](:[*]):[*]	-0.247
ECFP_6	-182236392	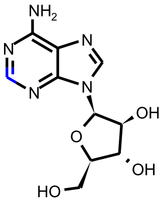 [*]:[cH]:[*]	-0.232
ECFP_6	-1884411803	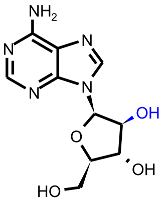 [*]O	-0.217

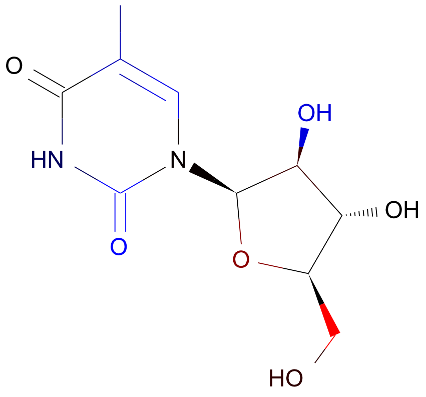

$C_{10}H_{14}N_2O_6$
Molecular Weight: 258.22795
ALogP: -2.005
Rotatable Bonds: 2
Acceptors: 6
Donors: 4

Model Prediction

Prediction: 67.851
Unit: mg/kg_body_weight/day
Mahalanobis Distance: 11.000
Mahalanobis Distance p-value: 0.00144

Mahalanobis Distance: The Mahalanobis distance (MD) is a generalization of the Euclidean distance that accounts for correlations among the X properties. It is calculated as the distance to the center of the training data. The larger the MD, the less trustworthy the prediction.
Mahalanobis Distance p-value: The p-value gives the fraction of training data with an MD greater than or equal to the one for the given sample, assuming normally distributed data. The smaller the p-value, the less trustworthy the prediciton. For highly non-normal X properties (e.g., fingerprints), the MD p-value is wildly inaccurate.

Structural Similar Compounds

Name	720	5-Azacytidine	AZT
Structure	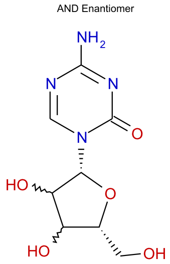	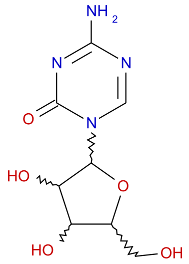	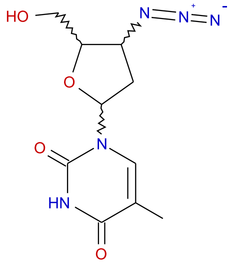
Actual Endpoint (-log C)	6.49901	6.49901	2.95561
Predicted Endpoint (-log C)	4.14504	4.14504	3.51843
Distance	0.470	0.470	0.607
Reference	CPDB	CPDB	CPDB

Model Applicability

Unknown features are fingerprint features in the query molecule, but not found in the training set.

1. All properties and OPS components are within expected ranges.

Feature Contribution

Top features for positive contribution			
**Fingerprint**	**Bit/Smiles**	**Feature Structure**	**Score**
ECFP_6	1559650422	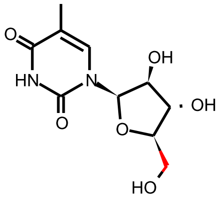 [*]C[*]	0.203

ECFP_6	683445015	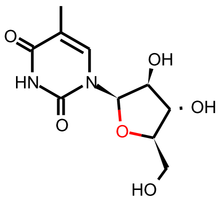 [*]O[*]	0.136
ECFP_6	-167460056	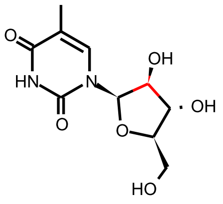 [*]C([*])[*]	0.060

Top Features for negative contribution

Fingerprint	Bit/Smiles	Feature Structure	Score
ECFP_6	2106656448	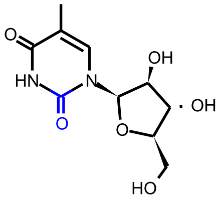 [*]C(=O)[*]	-0.275
ECFP_6	642810091	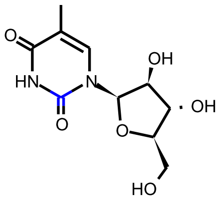 [*]:[c](:[*]):[*]	-0.247
ECFP_6	-182236392	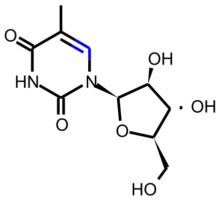 [*]:[cH]:[*]	-0.232

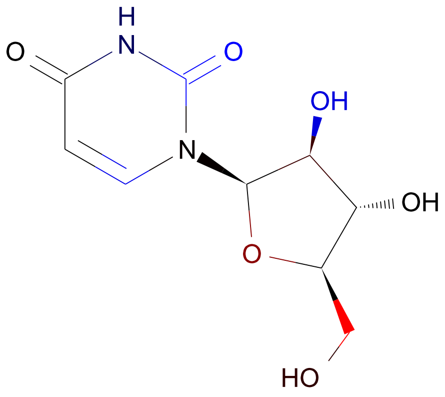

$C_9H_{12}N_2O_6$
Molecular Weight: 244.20137
ALogP: -2.451
Rotatable Bonds: 2
Acceptors: 6
Donors: 4

Model Prediction

Prediction: 55.437
Unit: mg/kg_body_weight/day
Mahalanobis Distance: 10.082
Mahalanobis Distance p-value: 0.0287

Mahalanobis Distance: The Mahalanobis distance (MD) is a generalization of the Euclidean distance that accounts for correlations among the X properties. It is calculated as the distance to the center of the training data. The larger the MD, the less trustworthy the prediction.
Mahalanobis Distance p-value: The p-value gives the fraction of training data with an MD greater than or equal to the one for the given sample, assuming normally distributed data. The smaller the p-value, the less trustworthy the prediciton. For highly non-normal X properties (e.g., fingerprints), the MD p-value is wildly inaccurate.

Structural Similar Compounds

Name	5-Azacytidine	720	534
Structure	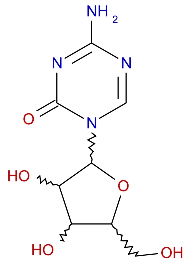	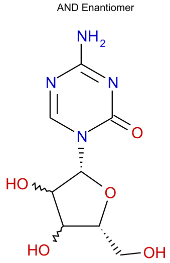	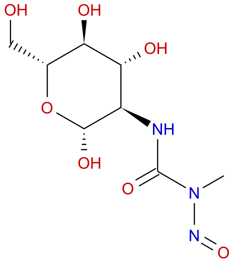
Actual Endpoint (-log C)	6.49901	6.49901	5.98904
Predicted Endpoint (-log C)	4.14504	4.14504	5.04171
Distance	0.465	0.465	0.668
Reference	CPDB	CPDB	CPDB

Model Applicability

Unknown features are fingerprint features in the query molecule, but not found in the training set.

1. All properties and OPS components are within expected ranges.
2. Unknown ECFP_2 feature: 935510419: [*]\C=C/N([*])[*]

Feature Contribution

Top features for positive contribution			
**Fingerprint**	**Bit/Smiles**	**Feature Structure**	**Score**
ECFP_6	1559650422	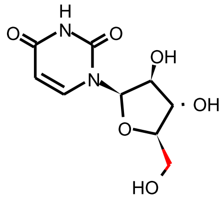 [*]C[*]	0.203

ECFP_6	683445015	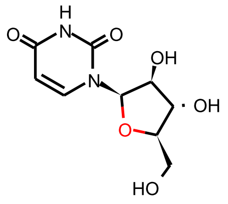 [*]O[*]	0.136
ECFP_6	-167460056	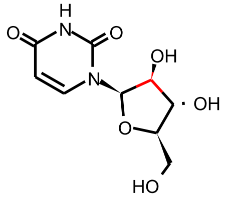 [*]C([*])[*]	0.060

Top Features for negative contribution

Fingerprint	Bit/Smiles	Feature Structure	Score
ECFP_6	2106656448	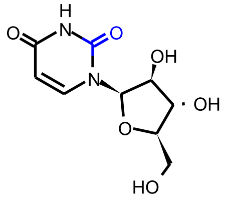 [*]C(=O)[*]	-0.275
ECFP_6	642810091	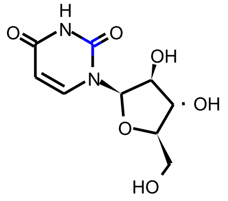 [*]:[c](:[*]):[*]	-0.247
ECFP_6	-182236392	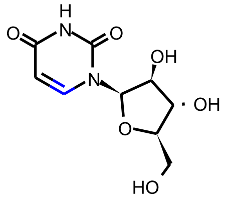 [*]:[cH]:[*]	-0.232

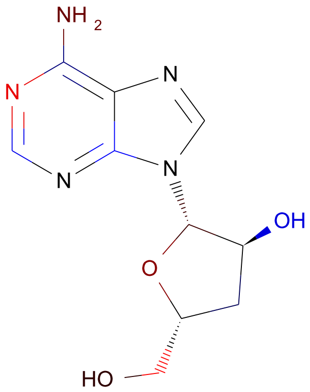

$C_{10}H_{13}N_5O_3$
Molecular Weight: 251.24191
ALogP: -1.308
Rotatable Bonds: 2
Acceptors: 7
Donors: 3

Model Prediction

Prediction: 6.402
Unit: mg/kg_body_weight/day
Mahalanobis Distance: 9.516
Mahalanobis Distance p-value: 0.113

Mahalanobis Distance: The Mahalanobis distance (MD) is a generalization of the Euclidean distance that accounts for correlations among the X properties. It is calculated as the distance to the center of the training data. The larger the MD, the less trustworthy the prediction.
Mahalanobis Distance p-value: The p-value gives the fraction of training data with an MD greater than or equal to the one for the given sample, assuming normally distributed data. The smaller the p-value, the less trustworthy the prediciton. For highly non-normal X properties (e.g., fingerprints), the MD p-value is wildly inaccurate.

Structural Similar Compounds

Name	(N-6)-(Methylnitroso)adenosine	377	Triamterene
Structure	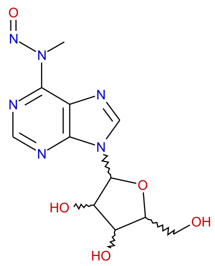	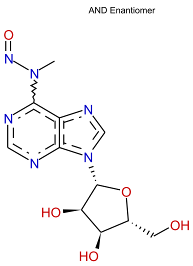	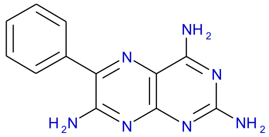
Actual Endpoint (-log C)	4.22928	4.22928	3.62397
Predicted Endpoint (-log C)	5.36013	5.36013	4.35116
Distance	0.546	0.546	0.702
Reference	CPDB	CPDB	CPDB

Model Applicability

Unknown features are fingerprint features in the query molecule, but not found in the training set.

1. All properties and OPS components are within expected ranges.

Feature Contribution

Top features for positive contribution

Fingerprint	Bit/Smiles	Feature Structure	Score
ECFP_6	655739385	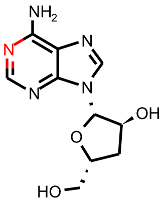 [*]:n:[*]	0.229

ECFP_6	1572579716	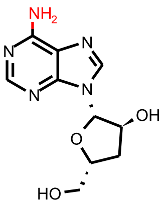 [*]N	0.225
ECFP_6	1559650422	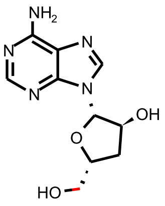 [*]C[*]	0.203

Top Features for negative contribution

Fingerprint	Bit/Smiles	Feature Structure	Score
ECFP_6	642810091	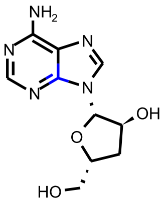 [*]:[c](:[*]):[*]	-0.247
ECFP_6	-182236392	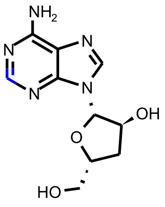 [*]:[cH]:[*]	-0.232
ECFP_6	-1884411803	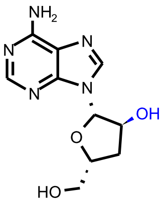 [*]O	-0.217

Ribavirin

TOPKAT_Carcinogenic_Potency_TD50_Mouse

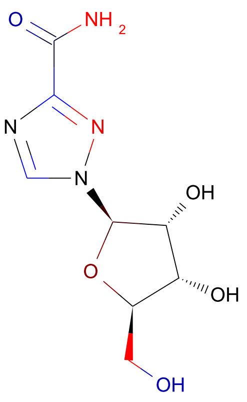

$C_8H_{12}N_4O_5$
Molecular Weight: 244.20467
ALogP: -2.745
Rotatable Bonds: 3
Acceptors: 7
Donors: 4

Structural Similar Compounds

Name	5-Azacytidine	720	534
Structure	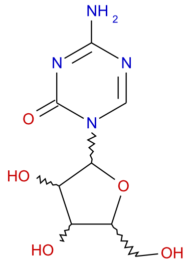	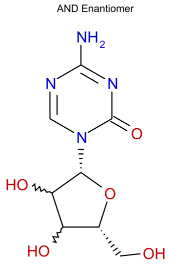	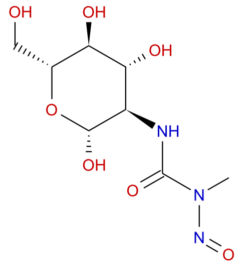
Actual Endpoint (-log C)	6.49901	6.49901	5.98904
Predicted Endpoint (-log C)	4.14504	4.14504	5.04171
Distance	0.577	0.577	0.638
Reference	CPDB	CPDB	CPDB

Model Prediction

Prediction: 13.111
Unit: mg/kg_body_weight/day
Mahalanobis Distance: 9.209
Mahalanobis Distance p-value: 0.205

Mahalanobis Distance: The Mahalanobis distance (MD) is a generalization of the Euclidean distance that accounts for correlations among the X properties. It is calculated as the distance to the center of the training data. The larger the MD, the less trustworthy the prediction.
Mahalanobis Distance p-value: The p-value gives the fraction of training data with an MD greater than or equal to the one for the given sample, assuming normally distributed data. The smaller the p-value, the less trustworthy the prediciton. For highly non-normal X properties (e.g., fingerprints), the MD p-value is wildly inaccurate.

Model Applicability

Unknown features are fingerprint features in the query molecule, but not found in the training set.

1. All properties and OPS components are within expected ranges.
2. Unknown ECFP_2 feature: -1142718401: [*]C([*])n1:n:[*]:[*]:c:1
3. Unknown ECFP_2 feature: 1128080580: [*]C(=[*])[c]1:n:[*]:[*]:n:1

Feature Contribution

Top features for positive contribution			
Fingerprint	Bit/Smiles	Feature Structure	Score
ECFP_6	655739385	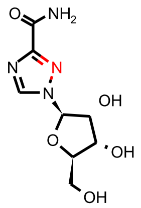 [*]:n:[*]	0.229

ECFP_6	1572579716	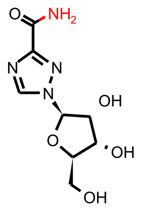 [*]N	0.225
ECFP_6	1559650422	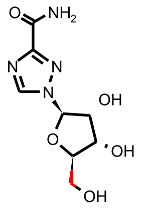 [*]C[*]	0.203
**Top Features for negative contribution**			
**Fingerprint**	**Bit/Smiles**	**Feature Structure**	**Score**
ECFP_6	642810091	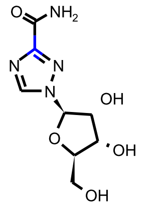 [*]:[c](:[*]):[*]	-0.247
ECFP_6	-182236392	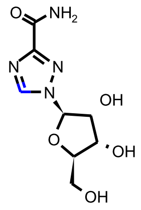 [*]:[cH]:[*]	-0.232
ECFP_6	-1884411803	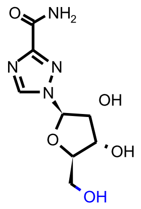 [*]O	-0.217

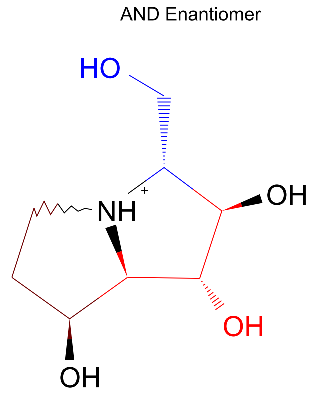

$C_8H_{16}NO_4$
Molecular Weight: 190.21693
ALogP: -3.556
Rotatable Bonds: 1
Acceptors: 4
Donors: 5

Model Prediction

Prediction: 2.818
Unit: mg/kg_body_weight/day
Mahalanobis Distance: 9.390
Mahalanobis Distance p-value: 0.479

Mahalanobis Distance: The Mahalanobis distance (MD) is a generalization of the Euclidean distance that accounts for correlations among the X properties. It is calculated as the distance to the center of the training data. The larger the MD, the less trustworthy the prediction.
Mahalanobis Distance p-value: The p-value gives the fraction of training data with an MD greater than or equal to the one for the given sample, assuming normally distributed data. The smaller the p-value, the less trustworthy the prediciton. For highly non-normal X properties (e.g., fingerprints), the MD p-value is wildly inaccurate.

Structural Similar Compounds

Name	Dibromodulcitol	5-Azacytidine	720
Structure	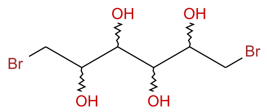	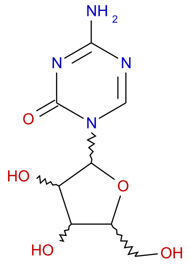	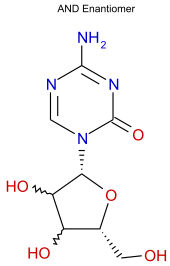
Actual Endpoint (-log C)	4.56578	6.15731	6.15731
Predicted Endpoint (-log C)	5.42021	5.04824	5.04824
Distance	0.823	0.831	0.831
Reference	CPDB	CPDB	CPDB

Model Applicability

Unknown features are fingerprint features in the query molecule, but not found in the training set.

1. All properties and OPS components are within expected ranges.

Feature Contribution

Top features for positive contribution

Fingerprint	Bit/Smiles	Feature Structure	Score
FCFP_6	-1043250487	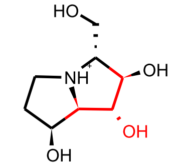 [*][C@@H]1[*][*][C@H]([*])[C@H]1O	1.153

FCFP_6	-1272798659	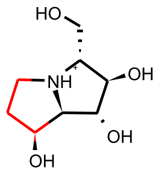 [*][C@@H]1[*][*]CC1	0.110
FCFP_6	3	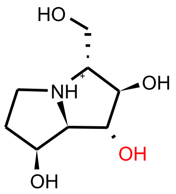 [*]O	0.064

Top Features for negative contribution

Fingerprint	Bit/Smiles	Feature Structure	Score
FCFP_6	-1272709286	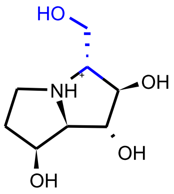 [*]C([*])CO	-0.526
FCFP_6	0	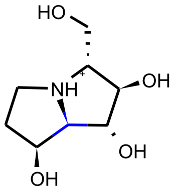 [*]C([*])[*]	-0.115

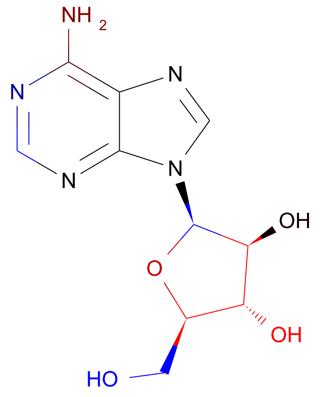

$C_{10}H_{13}N_5O_4$
Molecular Weight: 267.24132
ALogP: -1.881
Rotatable Bonds: 2
Acceptors: 8
Donors: 4

Model Prediction

Prediction: 3.280
Unit: mg/kg_body_weight/day
Mahalanobis Distance: 11.535
Mahalanobis Distance p-value: 0.0032

Mahalanobis Distance: The Mahalanobis distance (MD) is a generalization of the Euclidean distance that accounts for correlations among the X properties. It is calculated as the distance to the center of the training data. The larger the MD, the less trustworthy the prediction.
Mahalanobis Distance p-value: The p-value gives the fraction of training data with an MD greater than or equal to the one for the given sample, assuming normally distributed data. The smaller the p-value, the less trustworthy the prediciton. For highly non-normal X properties (e.g., fingerprints), the MD p-value is wildly inaccurate.

Structural Similar Compounds

Name	b-Thioguanine deoxyriboside	604	C.I. disperse blue 1
Structure	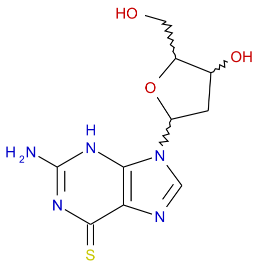	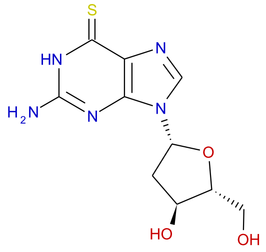	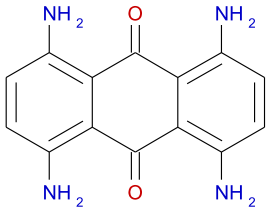
Actual Endpoint (-log C)	5.13004	5.13004	3.23545
Predicted Endpoint (-log C)	4.82552	4.96687	4.14295
Distance	0.540	0.555	0.690
Reference	CPDB	CPDB	CPDB

Model Applicability

Unknown features are fingerprint features in the query molecule, but not found in the training set.

1. All properties and OPS components are within expected ranges.

Feature Contribution

Top features for positive contribution

Fingerprint	Bit/Smiles	Feature Structure	Score
FCFP_6	-1043250487	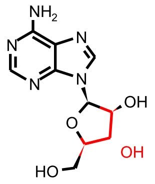 [*][C@@H]1[*][*][C@H]([*])[C@H]1O	1.153

FCFP_6	1	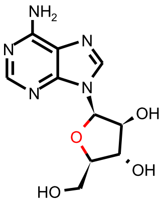 [*]O[*]	0.234
FCFP_6	-1043310069	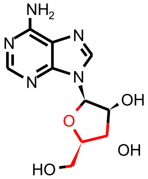 [*]C[C@H]1O[*][*][C@@H]1[*]	0.085
**Top Features for negative contribution**			
**Fingerprint**	**Bit/Smiles**	**Feature Structure**	**Score**
FCFP_6	-1272709286	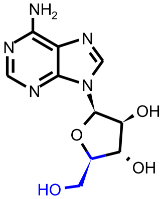 [*]C([*])CO	-0.526
FCFP_6	16	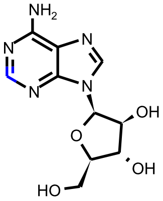 [*]:[cH]:[*]	-0.354
FCFP_6	17	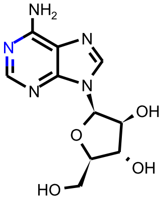 [*]:n:[*]	-0.149

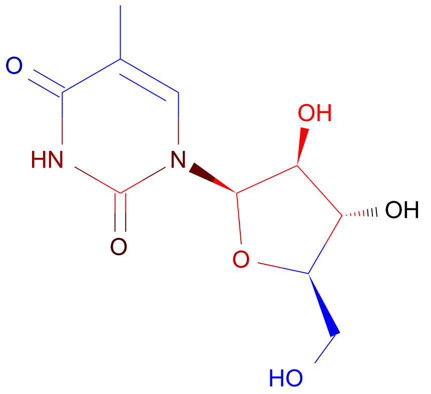

$C_{10}H_{14}N_2O_6$
Molecular Weight: 258.22795
ALogP: -2.005
Rotatable Bonds: 2
Acceptors: 6
Donors: 4

Structural Similar Compounds

Name	720	5-Azacytidine	AZT
Structure	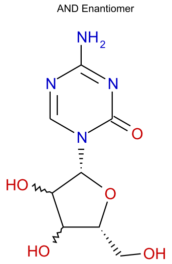	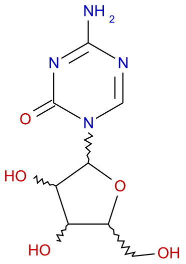	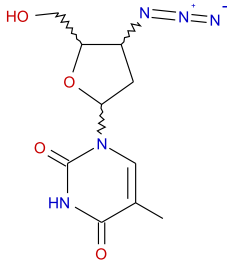
Actual Endpoint (-log C)	6.15731	6.15731	1.36245
Predicted Endpoint (-log C)	5.04824	5.04824	4.51955
Distance	0.442	0.442	0.559
Reference	CPDB	CPDB	CPDB

Model Prediction

Prediction: 4.844
Unit: mg/kg_body_weight/day
Mahalanobis Distance: 10.874
Mahalanobis Distance p-value: 0.0266

Mahalanobis Distance: The Mahalanobis distance (MD) is a generalization of the Euclidean distance that accounts for correlations among the X properties. It is calculated as the distance to the center of the training data. The larger the MD, the less trustworthy the prediction.
Mahalanobis Distance p-value: The p-value gives the fraction of training data with an MD greater than or equal to the one for the given sample, assuming normally distributed data. The smaller the p-value, the less trustworthy the prediciton. For highly non-normal X properties (e.g., fingerprints), the MD p-value is wildly inaccurate.

Model Applicability

Unknown features are fingerprint features in the query molecule, but not found in the training set.

1. All properties and OPS components are within expected ranges.

Feature Contribution

Top features for positive contribution			
**Fingerprint**	**Bit/Smiles**	**Feature Structure**	**Score**
FCFP_6	-1043250487	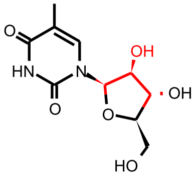 [*][C@@H]1[*][*][C@H]([*])[C@H]1O	1.153

FCFP_6	1	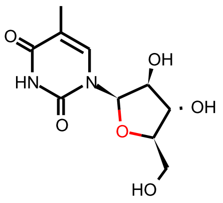 [*]O[*]	0.234
FCFP_6	-885550502	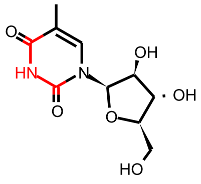 [*]C(=[*])NC(=[*])[*]	0.229

Top Features for negative contribution

Fingerprint	Bit/Smiles	Feature Structure	Score
FCFP_6	-1272709286	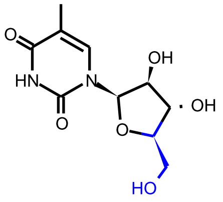 [*]C([*])CO	-0.526
FCFP_6	436886043	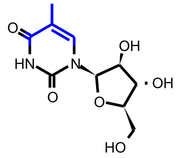 [*]\C=C(\C)/C(=[*])[*]<br>]	-0.383
FCFP_6	566058135	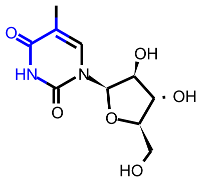 [*]NC(=O)C(=[*])[*]	-0.182

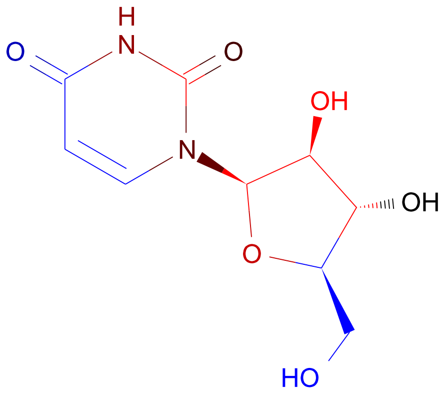

$C_9H_{12}N_2O_6$

Molecular Weight: 244.20137

ALogP: -2.451

Rotatable Bonds: 2

Acceptors: 6

Donors: 4

Model Prediction

Prediction: 6.735

Unit: mg/kg_body_weight/day

Mahalanobis Distance: 10.784

Mahalanobis Distance p-value: 0.0343

Mahalanobis Distance: The Mahalanobis distance (MD) is a generalization of the Euclidean distance that accounts for correlations among the X properties. It is calculated as the distance to the center of the training data. The larger the MD, the less trustworthy the prediction.
Mahalanobis Distance p-value: The p-value gives the fraction of training data with an MD greater than or equal to the one for the given sample, assuming normally distributed data. The smaller the p-value, the less trustworthy the prediciton. For highly non-normal X properties (e.g., fingerprints), the MD p-value is wildly inaccurate.

Structural Similar Compounds

Name	5-Azacytidine	720	534
Structure	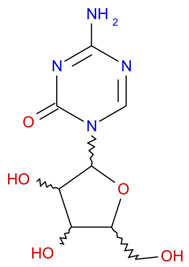	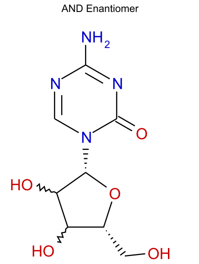	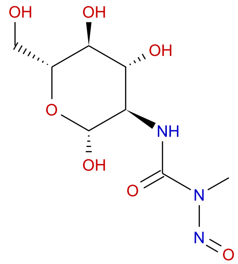
Actual Endpoint (-log C)	6.15731	6.15731	5.43998
Predicted Endpoint (-log C)	5.04824	5.04824	6.92268
Distance	0.438	0.438	0.622
Reference	CPDB	CPDB	CPDB

Model Applicability

Unknown features are fingerprint features in the query molecule, but not found in the training set.

1. All properties and OPS components are within expected ranges.

Feature Contribution

Top features for positive contribution

Fingerprint	Bit/Smiles	Feature Structure	Score
FCFP_6	-1043250487	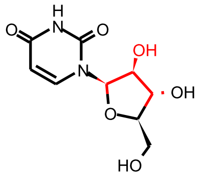 [*][C@@H]1[*][*][C@H]([*])[C@H]1O	1.153

FCFP_6	1	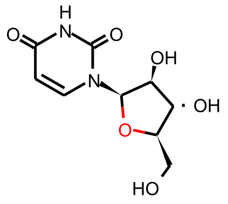<br>[*]O[*]	0.234
FCFP_6	-885550502	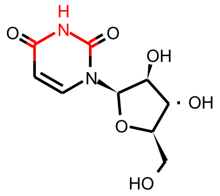<br>[*]C(=[*])NC(=[*])[*]	0.229
**Top Features for negative contribution**			
**Fingerprint**	**Bit/Smiles**	**Feature Structure**	**Score**
FCFP_6	-1272709286	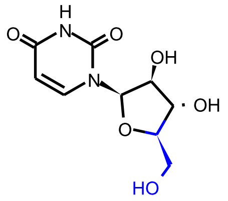<br>[*]C([*])CO	-0.526
FCFP_6	451847724	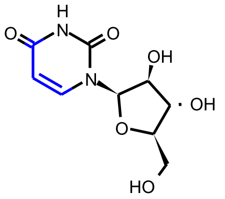<br>[*]\C=C/C(=[*])[*]	-0.436
FCFP_6	566058135	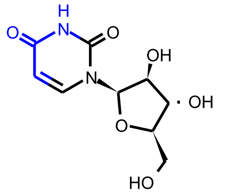<br>[*]NC(=O)C(=[*])[*]	-0.182

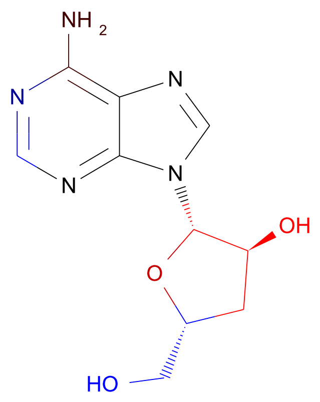

$C_{10}H_{13}N_5O_3$
Molecular Weight: 251.24191
ALogP: -1.308
Rotatable Bonds: 2
Acceptors: 7
Donors: 3

Model Prediction

Prediction: 2.651
Unit: mg/kg_body_weight/day
Mahalanobis Distance: 11.898
Mahalanobis Distance p-value: 0.000812

Mahalanobis Distance: The Mahalanobis distance (MD) is a generalization of the Euclidean distance that accounts for correlations among the X properties. It is calculated as the distance to the center of the training data. The larger the MD, the less trustworthy the prediction.
Mahalanobis Distance p-value: The p-value gives the fraction of training data with an MD greater than or equal to the one for the given sample, assuming normally distributed data. The smaller the p-value, the less trustworthy the prediciton. For highly non-normal X properties (e.g., fingerprints), the MD p-value is wildly inaccurate.

Structural Similar Compounds

Name	b-Thioguanine deoxyriboside	604	4,6-Diamino-2-(5-nitro-2-furyl)-S-triazine
Structure	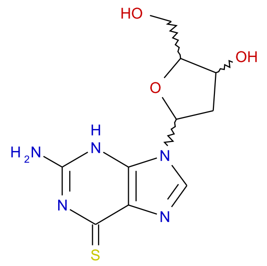	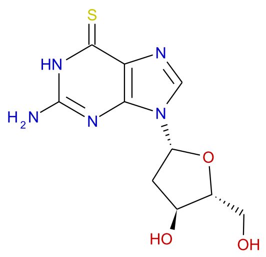	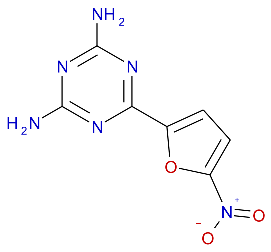
Actual Endpoint (-log C)	5.13004	5.13004	5.11367
Predicted Endpoint (-log C)	4.82552	4.96687	4.92048
Distance	0.605	0.607	0.696
Reference	CPDB	CPDB	CPDB

Model Applicability

Unknown features are fingerprint features in the query molecule, but not found in the training set.

1. All properties and OPS components are within expected ranges.

Feature Contribution

Top features for positive contribution

Fingerprint	Bit/Smiles	Feature Structure	Score
FCFP_6	-1043250487	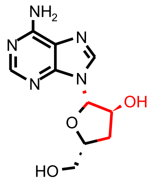 [*][C@@H]1[*][*][C@H]([*])[C@H]1O	1.153

FCFP_6	1	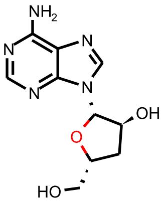<br>[*]O[*]	0.234
FCFP_6	-1272798659	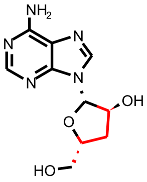<br>[*][C@@H]1[*][*]CC1	0.110
**Top Features for negative contribution**			
**Fingerprint**	**Bit/Smiles**	**Feature Structure**	**Score**
FCFP_6	-1272709286	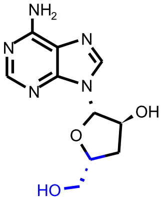<br>[*]C([*])CO	-0.526
FCFP_6	16	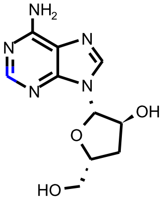<br>[*]:[cH]:[*]	-0.354
FCFP_6	17	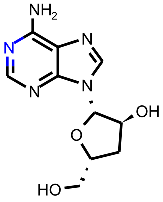<br>[*]:n:[*]	-0.149

Ribavirin

TOPKAT_Carcinogenic_Potency_TD50_Rat

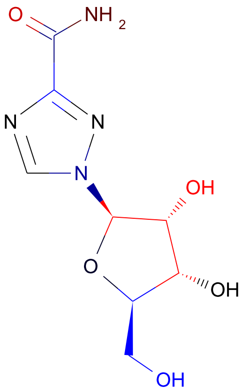

$C_8H_{12}N_4O_5$

Molecular Weight: 244.20467

ALogP: -2.745

Rotatable Bonds: 3

Acceptors: 7

Donors: 4

Model Prediction

Prediction: 16.625

Unit: mg/kg_body_weight/day

Mahalanobis Distance: 10.582

Mahalanobis Distance p-value: 0.0578

Mahalanobis Distance: The Mahalanobis distance (MD) is a generalization of the Euclidean distance that accounts for correlations among the X properties. It is calculated as the distance to the center of the training data. The larger the MD, the less trustworthy the prediction.
Mahalanobis Distance p-value: The p-value gives the fraction of training data with an MD greater than or equal to the one for the given sample, assuming normally distributed data. The smaller the p-value, the less trustworthy the prediciton. For highly non-normal X properties (e.g., fingerprints), the MD p-value is wildly inaccurate.

Structural Similar Compounds

Name	604	5-Azacytidine	720
Structure	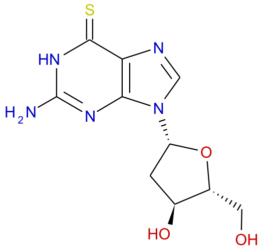	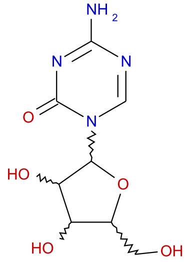	AND Enantiomer 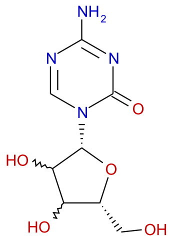
Actual Endpoint (-log C)	5.13004	6.15731	6.15731
Predicted Endpoint (-log C)	4.96687	5.04824	5.04824
Distance	0.557	0.566	0.566
Reference	CPDB	CPDB	CPDB

Model Applicability

Unknown features are fingerprint features in the query molecule, but not found in the training set.

1. All properties and OPS components are within expected ranges.

Feature Contribution

Top features for positive contribution

Fingerprint	Bit/Smiles	Feature Structure	Score
FCFP_6	-1043250487	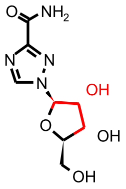 [*][C@@H]1[*][*][C@H]([*])[C@H]1O	1.153

FCFP_6	1	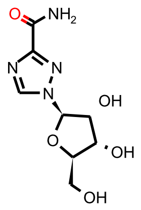 [*]O[*]	0.234
FCFP_6	-1043310069	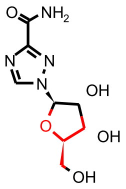 [*]C[C@H]1O[*][*][C@@H]1[*]	0.085
**Top Features for negative contribution**			
**Fingerprint**	**Bit/Smiles**	**Feature Structure**	**Score**
FCFP_6	-1272709286	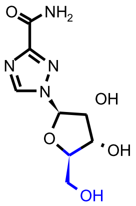 [*]C([*])CO	-0.526
FCFP_6	16	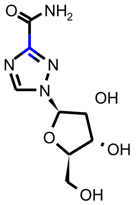 [*]:[cH]:[*]	-0.354
FCFP_6	17	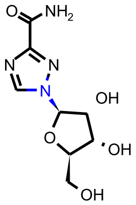 [*]:n:[*]	-0.149

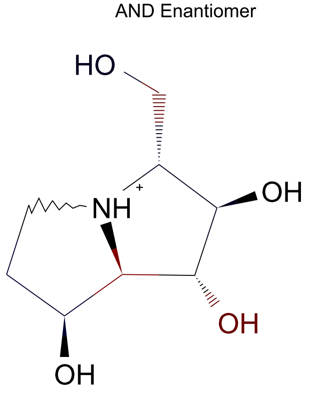

$C_8H_{16}NO_4$

Molecular Weight: 190.21693

ALogP: -3.556

Rotatable Bonds: 1

Acceptors: 4

Donors: 5

Model Prediction

Prediction: 0.018

Unit: g/kg_body_weight

Mahalanobis Distance: 29.463

Mahalanobis Distance p-value: 1.1e-023

Mahalanobis Distance: The Mahalanobis distance (MD) is a generalization of the Euclidean distance that accounts for correlations among the X properties. It is calculated as the distance to the center of the training data. The larger the MD, the less trustworthy the prediction.
Mahalanobis Distance p-value: The p-value gives the fraction of training data with an MD greater than or equal to the one for the given sample, assuming normally distributed data. The smaller the p-value, the less trustworthy the prediciton. For highly non-normal X properties (e.g., fingerprints), the MD p-value is wildly inaccurate.

Structural Similar Compounds

Name	L-ASCORBIC ACID	GLYPHOSATE	D-MANNITOL
Structure	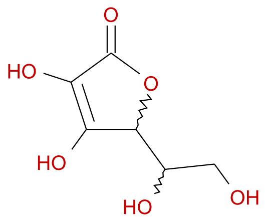	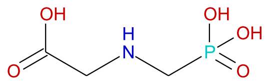	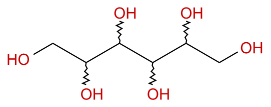
Actual Endpoint (-log C)	1.84788	3.75094	2.16357
Predicted Endpoint (-log C)	4.02723	3.64652	2.8029
Distance	0.656	0.708	0.731
Reference	NTP 247 115	EPA COVER SHEET 0057;891001;(1)	NTP REPORT # 236

Model Applicability

Unknown features are fingerprint features in the query molecule, but not found in the training set.

1. All properties and OPS components are within expected ranges.
2. Unknown FCFP_2 feature: 10: [*][NH+]([*])[*]
3. Unknown FCFP_2 feature: -1853714334: [*][C@@H]1[*][*][C@H]2[*][*]C[NH+]12
4. Unknown FCFP_2 feature: -1817836174: [*][C@H]1[*][*][NH+]2[*][*][C@H]([*])C12
5. Unknown FCFP_2 feature: 1155241219: [*][NH+]1[*][*]CC1
6. Unknown ECFP_6 feature: 1976330679: [*][NH+]([*])[*]
7. Unknown ECFP_6 feature: 2024749573: [*]C([*])O
8. Unknown ECFP_6 feature: 2022454958: [*]CO
9. Unknown ECFP_6 feature: -1693770146: [*][C@@H]1[*][*][C@H]2[*][*]C[NH+]12
10. Unknown ECFP_6 feature: -1395316370: [*][C@H]1[*][*][NH+]2[*][*][C@H]([*])C12
11. Unknown ECFP_6 feature: -705668329: [*]C[C@@H]1[C@@H]([*])[*][*][NH+]1[*]
12. Unknown ECFP_6 feature: 305695353: [*][C@@H]1[*][*][C@H]([*])[C@H]1O
13. Unknown ECFP_6 feature: -329826665: [*][C@@H]1[*][*]C[C@@H]1O
14. Unknown ECFP_6 feature: -244159614: [*][NH+]1[*][*]CC1
15. Unknown ECFP_6 feature: -85480422: [*]C([*])CO

Feature Contribution

Top features for positive contribution

Fingerprint	Bit/Smiles	Feature Structure	Score

ECFP_6	-167460056	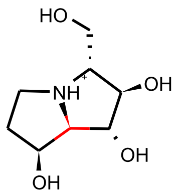 [*]C([*])[*]	0.136
ECFP_6	1559650422	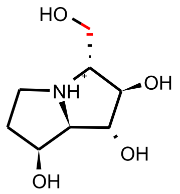 [*]C[*]	0.129
FCFP_6	3	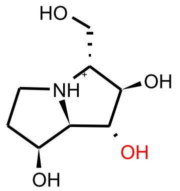 [*]O	0.092
**Top Features for negative contribution**			
**Fingerprint**	**Bit/Smiles**	**Feature Structure**	**Score**
ECFP_6	-1331450522	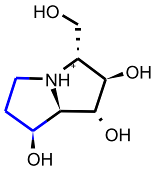 [*][C@@H]1[*][*]CC1	-0.054
FCFP_6	-1272709286	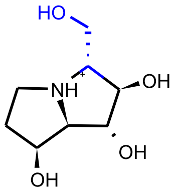 [*]C([*])CO	-0.031

FCFP_6	-1043250487	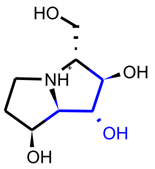<br>[*][C@@H]1[*][*][C@H]([*])[C@H]1O	-0.027

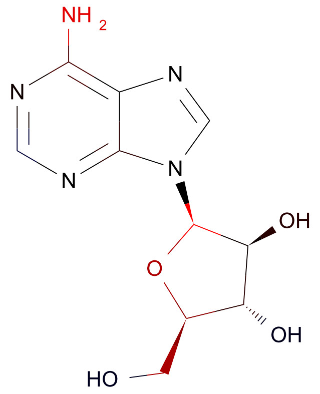

$C_{10}H_{13}N_5O_4$
Molecular Weight: 267.24132
ALogP: -1.881
Rotatable Bonds: 2
Acceptors: 8
Donors: 4

Model Prediction

Prediction: 0.010
Unit: g/kg_body_weight
Mahalanobis Distance: 44.555
Mahalanobis Distance p-value: 1.76e-049

Mahalanobis Distance: The Mahalanobis distance (MD) is a generalization of the Euclidean distance that accounts for correlations among the X properties. It is calculated as the distance to the center of the training data. The larger the MD, the less trustworthy the prediction.
Mahalanobis Distance p-value: The p-value gives the fraction of training data with an MD greater than or equal to the one for the given sample, assuming normally distributed data. The smaller the p-value, the less trustworthy the prediciton. For highly non-normal X properties (e.g., fingerprints), the MD p-value is wildly inaccurate.

Structural Similar Compounds

Name	C.I. DISPERSE BLUE I	AMPICILLIN TRIHYDRATE	AMILORIDE
Structure	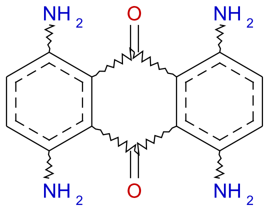	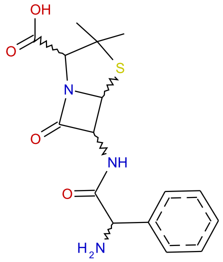	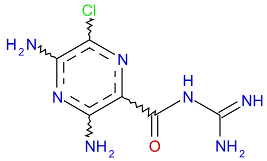
Actual Endpoint (-log C)	3.6327	2.81416	4.75896
Predicted Endpoint (-log C)	3.26657	3.59519	3.54686
Distance	0.658	0.703	0.703
Reference	NTP REPORT # 299	NTP REPORT # 318	NDA-18201

Model Applicability

Unknown features are fingerprint features in the query molecule, but not found in the training set.

1. All properties and OPS components are within expected ranges.
2. Unknown ECFP_6 feature: 672362763: [*]n(:[*]):[*]
3. Unknown ECFP_6 feature: -677309799: [*][c](:[*]):n:[cH]:[*]
4. Unknown ECFP_6 feature: -710237522: [*]:n:[cH]:n:[*]
5. Unknown ECFP_6 feature: 1049075205: [*]n1:[*]:[*]:[c](:[*]):[c]:1:n:[*]
6. Unknown ECFP_6 feature: 1048320787: [*][c](:[*]):[c]1:n:[*]:[*]:[c]:1:[*]
7. Unknown ECFP_6 feature: -1734834311: [*]:n:[c](N):[c](:[*]):[*]
8. Unknown ECFP_6 feature: -938530932: [*]:[c](:[*])N
9. Unknown ECFP_6 feature: -1219098860: [*]C([*])n1:[cH]:[*]:[*]:[c]:1:[*]
10. Unknown ECFP_6 feature: -708878603: [*]n1:[*]:[*]:n:[cH]:1
11. Unknown ECFP_6 feature: 125442029: [*][C@H]1[*][*]O[C@H]1n(:[*]):[*]
12. Unknown ECFP_6 feature: -1409796893: [*][C@@H]1[*][*][C@H]([*])O1
13. Unknown ECFP_6 feature: -553149446: [*]C[C@H]1O[*][*][C@@H]1[*]
14. Unknown ECFP_6 feature: 305695353: [*][C@@H]1[*][*][C@H]([*])[C@H]1O
15. Unknown ECFP_6 feature: 2024749573: [*]C([*])O
16. Unknown ECFP_6 feature: -85480422: [*]C([*])CO
17. Unknown ECFP_6 feature: 2022454958: [*]CO

Feature Contribution

Top features for positive contribution

Fingerprint	Bit/Smiles	Feature Structure	Score
ECFP_6	-167460056	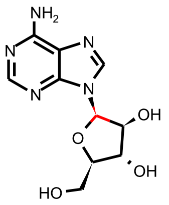 [*]C([*])[*]	0.136
FCFP_6	-1143715940	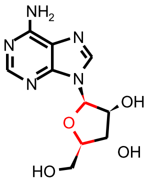 [*][C@@H]1[*][*][C@H]([*])O1	0.130
ECFP_6	1559650422	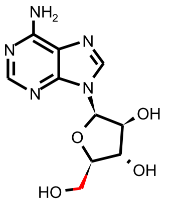 [*]C[*]	0.129
**Top Features for negative contribution**			
**Fingerprint**	**Bit/Smiles**	**Feature Structure**	**Score**
FCFP_6	1	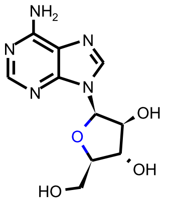 [*]O[*]	-0.102

FCFP_6	16	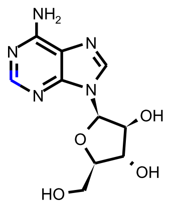 [*]:[cH]:[*]	-0.046
FCFP_6	-1272709286	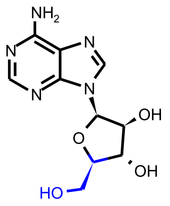 [*]C([*])CO	-0.031

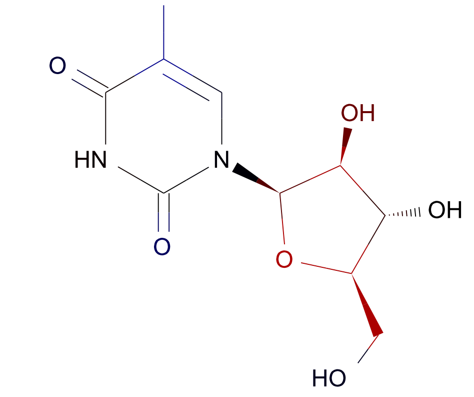

$C_{10}H_{14}N_2O_6$
Molecular Weight: 258.22795
ALogP: -2.005
Rotatable Bonds: 2
Acceptors: 6
Donors: 4

Model Prediction

Prediction: 0.009
Unit: g/kg_body_weight
Mahalanobis Distance: 38.315
Mahalanobis Distance p-value: 2.4e-039

Mahalanobis Distance: The Mahalanobis distance (MD) is a generalization of the Euclidean distance that accounts for correlations among the X properties. It is calculated as the distance to the center of the training data. The larger the MD, the less trustworthy the prediction.
Mahalanobis Distance p-value: The p-value gives the fraction of training data with an MD greater than or equal to the one for the given sample, assuming normally distributed data. The smaller the p-value, the less trustworthy the prediciton. For highly non-normal X properties (e.g., fingerprints), the MD p-value is wildly inaccurate.

Structural Similar Compounds

Name	L-ASCORBIC ACID	AMPICILLIN TRIHYDRATE	GLYPHOSATE
Structure	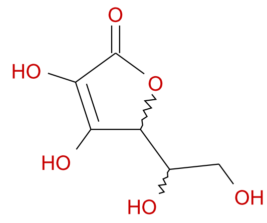	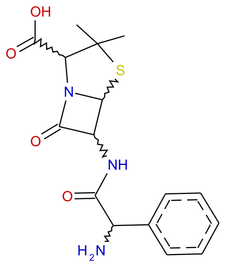	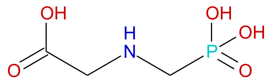
Actual Endpoint (-log C)	1.84788	2.81416	3.75094
Predicted Endpoint (-log C)	4.02723	3.59519	3.64652
Distance	0.544	0.682	0.705
Reference	NTP 247 115	NTP REPORT # 318	EPA COVER SHEET 0057;891001;(1)

Model Applicability

Unknown features are fingerprint features in the query molecule, but not found in the training set.

1. All properties and OPS components are within expected ranges.
2. Unknown ECFP_6 feature: -154530762: [*]N[*]
3. Unknown ECFP_6 feature: -1409796893: [*][C@@H]1[*][*][C@H]([*])O1
4. Unknown ECFP_6 feature: 2024749573: [*]C([*])O
5. Unknown ECFP_6 feature: 2022454958: [*]CO
6. Unknown ECFP_6 feature: -1811420270: [*]C([*])N(C=[*])C(=[*])[*]
7. Unknown ECFP_6 feature: -1699286547: [*]C(=[*])NC(=[*])[*]
8. Unknown ECFP_6 feature: 305695353: [*][C@@H]1[*][*][C@H]([*])[C@H]1O
9. Unknown ECFP_6 feature: -627157165: [*][C@H]1[*][*]O[C@H]1N([*])[*]
10. Unknown ECFP_6 feature: -553149446: [*]C[C@H]1O[*][*][C@@H]1[*]
11. Unknown ECFP_6 feature: -85480422: [*]C([*])CO
12. Unknown ECFP_6 feature: 471356069: [*]N([*])C=C([*])[*]
13. Unknown ECFP_6 feature: 1526392165: [*]NC(=O)N([*])[*]
14. Unknown ECFP_6 feature: -3067141: [*]\C=C(\C)/C(=[*])[*]
15. Unknown ECFP_6 feature: 1298725959: [*]NC(=O)C(=[*])[*]

Feature Contribution

Top features for positive contribution			
Fingerprint	Bit/Smiles	Feature Structure	Score

ECFP_6	-167460056	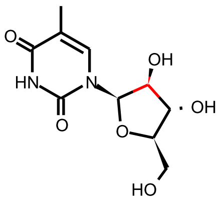 [*]C([*])[*]	0.136
FCFP_6	-1143715940	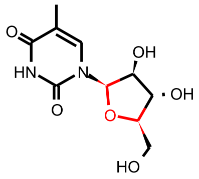 [*][C@@H]1[*][*][C@H]([*])O1	0.130
ECFP_6	1559650422	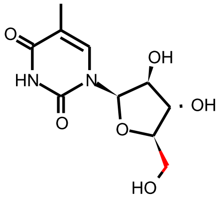 [*]C[*]	0.129

Top Features for negative contribution

Fingerprint	Bit/Smiles	Feature Structure	Score
ECFP_6	2106656448	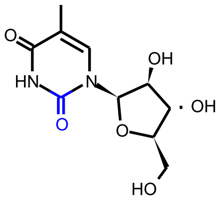 [*]C(=O)[*]	-0.110
FCFP_6	1	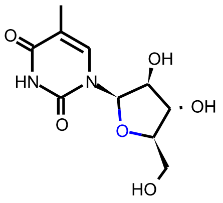 [*]O[*]	-0.102

FCFP_6	136597326	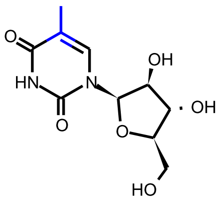 [*]C(=[*])C	-0.081

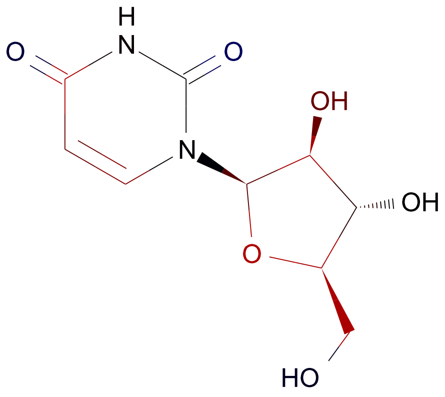

$C_9H_{12}N_2O_6$

Molecular Weight: 244.20137

ALogP: -2.451

Rotatable Bonds: 2

Acceptors: 6

Donors: 4

Structural Similar Compounds

Name	L-ASCORBIC ACID	GLYPHOSATE	AMPICILLIN TRIHYDRATE
Structure	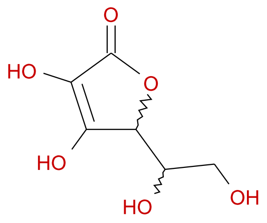	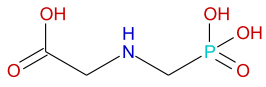	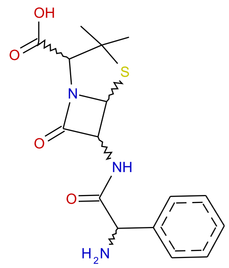
Actual Endpoint (-log C)	1.84788	3.75094	2.81416
Predicted Endpoint (-log C)	4.02723	3.64652	3.59519
Distance	0.527	0.660	0.701
Reference	NTP 247 115	EPA COVER SHEET 0057;891001;(1)	NTP REPORT # 318

Model Prediction

Prediction: 0.006

Unit: g/kg_body_weight

Mahalanobis Distance: 35.673

Mahalanobis Distance p-value: 8.25e-035

Mahalanobis Distance: The Mahalanobis distance (MD) is a generalization of the Euclidean distance that accounts for correlations among the X properties. It is calculated as the distance to the center of the training data. The larger the MD, the less trustworthy the prediction.
Mahalanobis Distance p-value: The p-value gives the fraction of training data with an MD greater than or equal to the one for the given sample, assuming normally distributed data. The smaller the p-value, the less trustworthy the prediciton. For highly non-normal X properties (e.g., fingerprints), the MD p-value is wildly inaccurate.

Model Applicability

Unknown features are fingerprint features in the query molecule, but not found in the training set.

1. All properties and OPS components are within expected ranges.
2. Unknown ECFP_6 feature: -154530762: [*]N[*]
3. Unknown ECFP_6 feature: -1409796893: [*][C@@H]1[*][*][C@H]([*])O1
4. Unknown ECFP_6 feature: 2024749573: [*]C([*])O
5. Unknown ECFP_6 feature: 2022454958: [*]CO
6. Unknown ECFP_6 feature: -1811420270: [*]C([*])N(C=[*])C(=[*])[*]
7. Unknown ECFP_6 feature: -1699286547: [*]C(=[*])NC(=[*])[*]
8. Unknown ECFP_6 feature: 305695353: [*][C@@H]1[*][*][C@H]([*])[C@H]1O
9. Unknown ECFP_6 feature: -627157165: [*][C@H]1[*][*]O[C@H]1N([*])[*]
10. Unknown ECFP_6 feature: -553149446: [*]C[C@H]1O[*][*][C@@H]1[*]
11. Unknown ECFP_6 feature: -85480422: [*]C([*])CO
12. Unknown ECFP_6 feature: 1526392165: [*]NC(=O)N([*])[*]
13. Unknown ECFP_6 feature: 935510419: [*]\C=C/N([*])[*]
14. Unknown ECFP_6 feature: 1745066357: [*]\C=C/C(=[*])[*]
15. Unknown ECFP_6 feature: -867415197: [*]NC(=O)C=[*]

Feature Contribution

Top features for positive contribution			
Fingerprint	Bit/Smiles	Feature Structure	Score

FCFP_6	451847724	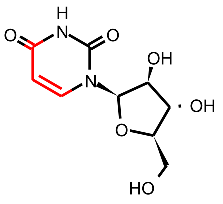<br>[*]\C=C/C(=[*])[*]	0.160
ECFP_6	-167460056	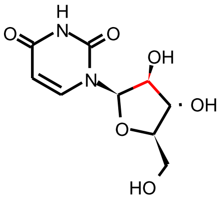<br>[*]C([*])[*]	0.136
FCFP_6	-1143715940	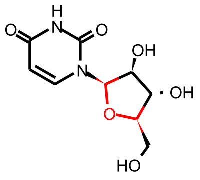<br>[*][C@@H]1[*][*][C@H]([*])O1	0.130

Top Features for negative contribution

Fingerprint	Bit/Smiles	Feature Structure	Score
ECFP_6	2106656448	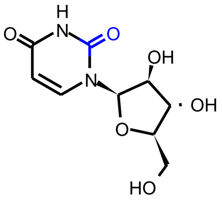<br>[*]C(=O)[*]	-0.110
FCFP_6	1	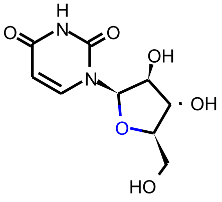<br>[*]O[*]	-0.102

FCFP_6	566058135	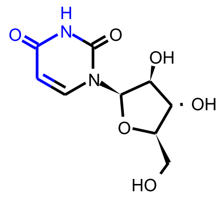 [*]NC(=O)C(=[*])[*]	-0.056

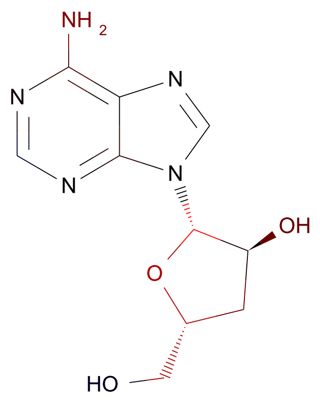

$C_{10}H_{13}N_5O_3$
Molecular Weight: 251.24191
ALogP: -1.308
Rotatable Bonds: 2
Acceptors: 7
Donors: 3

Model Prediction

Prediction: 0.004
Unit: g/kg_body_weight
Mahalanobis Distance: 45.467
Mahalanobis Distance p-value: 6.83e-051

Mahalanobis Distance: The Mahalanobis distance (MD) is a generalization of the Euclidean distance that accounts for correlations among the X properties. It is calculated as the distance to the center of the training data. The larger the MD, the less trustworthy the prediction.
Mahalanobis Distance p-value: The p-value gives the fraction of training data with an MD greater than or equal to the one for the given sample, assuming normally distributed data. The smaller the p-value, the less trustworthy the prediciton. For highly non-normal X properties (e.g., fingerprints), the MD p-value is wildly inaccurate.

Structural Similar Compounds

Name	TRIAMTERENE	HYDROCHLOROTHIAZIDE	PIROXICAM
Structure	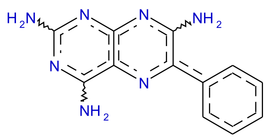	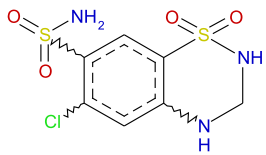	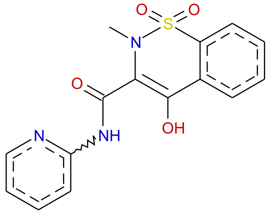
Actual Endpoint (-log C)	4.40358	4.37691	5.52028
Predicted Endpoint (-log C)	3.6657	3.84479	4.06087
Distance	0.660	0.683	0.690
Reference	NTP REPORT # 420	NTP REPORT # 357	NDA-18147

Model Applicability

Unknown features are fingerprint features in the query molecule, but not found in the training set.

1. All properties and OPS components are within expected ranges.
2. Unknown ECFP_6 feature: 672362763: [*]n(:[*]):[*]
3. Unknown ECFP_6 feature: -677309799: [*][c](:[*]):n:[cH]:[*]
4. Unknown ECFP_6 feature: -710237522: [*]:n:[cH]:n:[*]
5. Unknown ECFP_6 feature: 1049075205: [*]n1:[*]:[*]:[c](:[*]):[c]:1:n:[*]
6. Unknown ECFP_6 feature: 1048320787: [*][c](:[*]):[c]1:n:[*]:[*]:[c]:1:[*]
7. Unknown ECFP_6 feature: -1734834311: [*]:n:[c](N):[c](:[*]):[*]
8. Unknown ECFP_6 feature: -1219098860: [*]C([*])n1:[cH]:[*]:[*]:[c]:1:[*]
9. Unknown ECFP_6 feature: -708878603: [*]n1:[*]:[*]:n:[cH]:1
10. Unknown ECFP_6 feature: 125442029: [*][C@H]1[*][*]O[C@H]1n(:[*]):[*]
11. Unknown ECFP_6 feature: -1409796893: [*][C@@H]1[*][*][C@H]([*])O1
12. Unknown ECFP_6 feature: -554480104: [*]C[C@H]1C[*][*]O1
13. Unknown ECFP_6 feature: -801490360: [*][C@@H]1[*][*][C@@H]([*])C1
14. Unknown ECFP_6 feature: -329826665: [*][C@@H]1[*][*]C[C@@H]1O
15. Unknown ECFP_6 feature: 2024749573: [*]C([*])O
16. Unknown ECFP_6 feature: -85480422: [*]C([*])CO
17. Unknown ECFP_6 feature: 2022454958: [*]CO
18. Unknown ECFP_6 feature: -938530932: [*]:[c](:[*])N

Feature Contribution

Top features for positive contribution

Fingerprint	Bit/Smiles	Feature Structure	Score
ECFP_6	-167460056	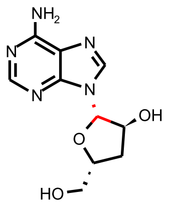 [*]C([*])[*]	0.136
FCFP_6	-1143715940	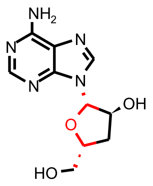 [*][C@@H]1[*][*][C@H]([*])O1	0.130
ECFP_6	1559650422	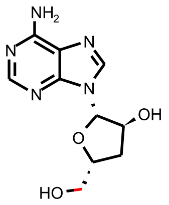 [*]C[*]	0.129

Top Features for negative contribution

Fingerprint	Bit/Smiles	Feature Structure	Score
FCFP_6	1	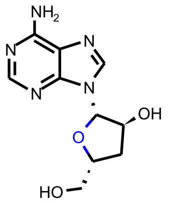 [*]O[*]	-0.102

FCFP_6	16	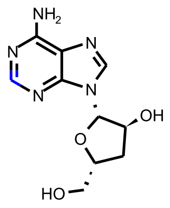 [*]:[cH]:[*]	-0.046
FCFP_6	-1272709286	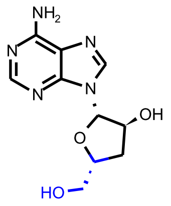 [*]C([*])CO	-0.031

Ribavirin

TOPKAT_Chronic_LOAEL

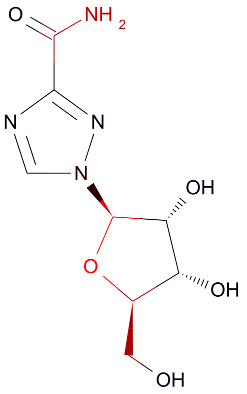

$C_8H_{12}N_4O_5$

Molecular Weight: 244.20467

ALogP: -2.745

Rotatable Bonds: 3

Acceptors: 7

Donors: 4

Model Prediction

Prediction: 0.013

Unit: g/kg_body_weight

Mahalanobis Distance: 42.536

Mahalanobis Distance p-value: 2.71e-046

Mahalanobis Distance: The Mahalanobis distance (MD) is a generalization of the Euclidean distance that accounts for correlations among the X properties. It is calculated as the distance to the center of the training data. The larger the MD, the less trustworthy the prediction.

Mahalanobis Distance p-value: The p-value gives the fraction of training data with an MD greater than or equal to the one for the given sample, assuming normally distributed data. The smaller the p-value, the less trustworthy the prediciton. For highly non-normal X properties (e.g., fingerprints), the MD p-value is wildly inaccurate.

Structural Similar Compounds

Name	AMILORIDE	AMPICILLIN TRIHYDRATE	L-ASCORBIC ACID
Structure	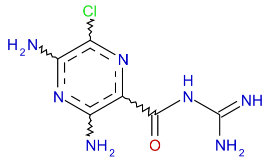	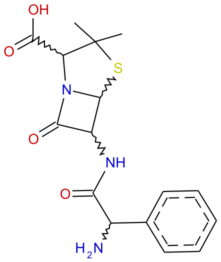	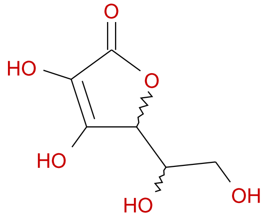
Actual Endpoint (-log C)	4.75896	2.81416	1.84788
Predicted Endpoint (-log C)	3.54686	3.59519	4.02723
Distance	0.638	0.648	0.688
Reference	NDA-18201	NTP REPORT # 318	NTP 247 115

Model Applicability

Unknown features are fingerprint features in the query molecule, but not found in the training set.

1. All properties and OPS components are within expected ranges.
2. Unknown ECFP_6 feature: 672362763: [*]n(:[*]):[*]
3. Unknown ECFP_6 feature: -1142718401: [*]C([*])n1:[cH]:[*]:[*]:n:1
4. Unknown ECFP_6 feature: 835630791: [*]n1:[*]:[*]:[c]([*]):n:1
5. Unknown ECFP_6 feature: 1128080580: [*]C(=[*])[c]1:n:[*]:[*]:n:1
6. Unknown ECFP_6 feature: -677309799: [*][c](:[*]):n:[cH]:[*]
7. Unknown ECFP_6 feature: -708878603: [*]n1:[*]:[*]:n:[cH]:1
8. Unknown ECFP_6 feature: 852414842: [*]:[c](:[*])C(=O)N
9. Unknown ECFP_6 feature: -932108170: [*]C(=[*])N
10. Unknown ECFP_6 feature: -1409796893: [*][C@@H]1[*][*][C@H]([*])O1
11. Unknown ECFP_6 feature: 125442029: [*][C@H]1[*][*]O[C@H]1n(:[*]):[*]
12. Unknown ECFP_6 feature: 305695353: [*][C@@H]1[*][*][C@H]([*])[C@H]1O
13. Unknown ECFP_6 feature: -553149446: [*]C[C@H]1O[*][*][C@@H]1[*]
14. Unknown ECFP_6 feature: -85480422: [*]C([*])CO
15. Unknown ECFP_6 feature: 2022454958: [*]CO
16. Unknown ECFP_6 feature: 2024749573: [*]C([*])O

Feature Contribution

Top features for positive contribution			
**Fingerprint**	**Bit/Smiles**	**Feature Structure**	**Score**

ECFP_6	-167460056	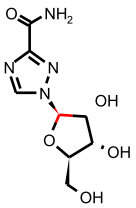 [*]C([*])[*]	0.136
FCFP_6	-1143715940	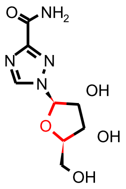 [*][C@@H]1[*][*][C@H]([*])O1	0.130
ECFP_6	1559650422	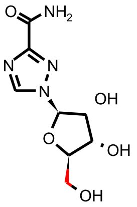 [*]C[*]	0.129

Top Features for negative contribution

Fingerprint	Bit/Smiles	Feature Structure	Score
FCFP_6	1	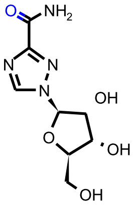 [*]O[*]	-0.102
FCFP_6	16	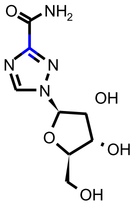 [*]:[cH]:[*]	-0.046

FCFP_6	-1272709286	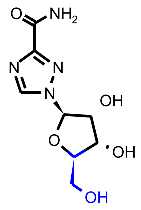 [*]C([*])CO	-0.031

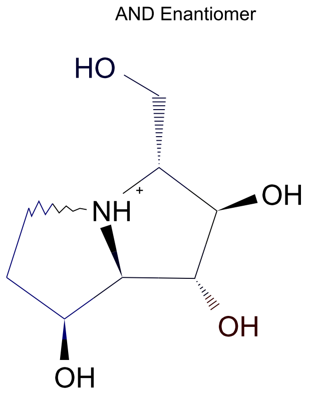

$C_8H_{16}NO_4$

Molecular Weight: 190.21693

ALogP: -3.556

Rotatable Bonds: 1

Acceptors: 4

Donors: 5

Model Prediction

Prediction: 0.191

Unit: g/kg_body_weight

Mahalanobis Distance: 8.310

Mahalanobis Distance p-value: 0.0109

Mahalanobis Distance: The Mahalanobis distance (MD) is a generalization of the Euclidean distance that accounts for correlations among the X properties. It is calculated as the distance to the center of the training data. The larger the MD, the less trustworthy the prediction.
Mahalanobis Distance p-value: The p-value gives the fraction of training data with an MD greater than or equal to the one for the given sample, assuming normally distributed data. The smaller the p-value, the less trustworthy the prediciton. For highly non-normal X properties (e.g., fingerprints), the MD p-value is wildly inaccurate.

Structural Similar Compounds

Name	D-MANNITOL	L-ASORBIC ACID	DIETHANOLAMINE
Structure	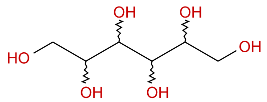	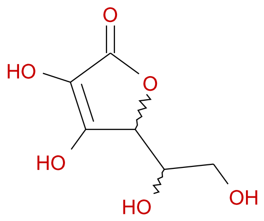	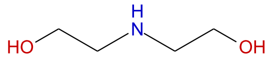
Actual Endpoint (-log C)	1.9083	1.89364	3.32279
Predicted Endpoint (-log C)	2.52388	2.56611	2.92091
Distance	0.651	0.670	0.791
Reference	NCI/NTP TR-236	NCI/NTP TR-247	NCI/NTP TR-478

Model Applicability

Unknown features are fingerprint features in the query molecule, but not found in the training set.

1. OPS PC3 out of range. Value: -5.7487. Training min, max, SD, explained variance: -5.5951, 5.5124, 2.075, 0.0973.
2. Unknown FCFP_2 feature: 10: [*][NH+]([*])[*]
3. Unknown FCFP_2 feature: -1853714334: [*][C@@H]1[*][*][C@H]2[*][*]C[NH+]12
4. Unknown FCFP_2 feature: -1817836174: [*][C@H]1[*][*][NH+]2[*][*][C@H]([*])C12
5. Unknown FCFP_2 feature: 1155241219: [*][NH+]1[*][*]CC1

Feature Contribution

Top features for positive contribution

Fingerprint	Bit/Smiles	Feature Structure	Score
FCFP_2	3	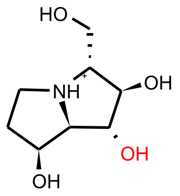 [*]O	0.074

Top Features for negative contribution

Fingerprint	Bit/Smiles	Feature Structure	Score
FCFP_2	-1272798659	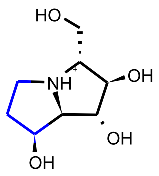<br>[*][C@@H]1[*][*]CC1	-0.111
FCFP_2	-1272709286	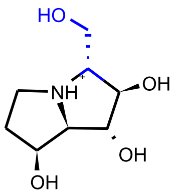<br>[*]C([*])CO	-0.049
FCFP_2	0	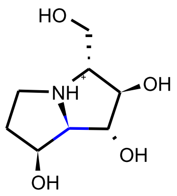<br>[*]C([*])[*]	-0.031

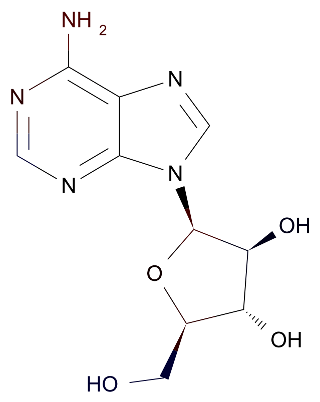

$C_{10}H_{13}N_5O_4$
Molecular Weight: 267.24132
ALogP: -1.881
Rotatable Bonds: 2
Acceptors: 8
Donors: 4

Structural Similar Compounds

Name	50%1,4,5,8-TETRAAMINOANTHRAQUIONONE + DERIVATIVES	TRIAMTERENE	MELAMINE
Structure	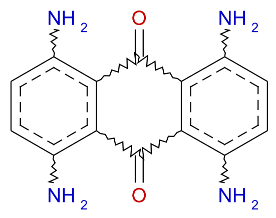	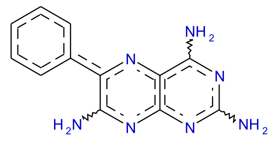	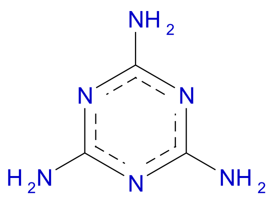
Actual Endpoint (-log C)	3.0764	4.00564	2.40182
Predicted Endpoint (-log C)	3.08142	3.1992	3.01288
Distance	0.664	0.699	0.796
Reference	NCI/NTP TR-299	NCI/NTP TR-420	NCI/NTP TR-245

Model Prediction

Prediction: 0.175
Unit: g/kg_body_weight
Mahalanobis Distance: 10.106
Mahalanobis Distance p-value: 2.99e-005

Mahalanobis Distance: The Mahalanobis distance (MD) is a generalization of the Euclidean distance that accounts for correlations among the X properties. It is calculated as the distance to the center of the training data. The larger the MD, the less trustworthy the prediction.
Mahalanobis Distance p-value: The p-value gives the fraction of training data with an MD greater than or equal to the one for the given sample, assuming normally distributed data. The smaller the p-value, the less trustworthy the prediciton. For highly non-normal X properties (e.g., fingerprints), the MD p-value is wildly inaccurate.

Model Applicability

Unknown features are fingerprint features in the query molecule, but not found in the training set.

1. All properties and OPS components are within expected ranges.
2. Unknown FCFP_2 feature: -124685461: [*]:n:c:n:[*]
3. Unknown FCFP_2 feature: -306856457: [*]C([*])n1:[c](:[*]):[*]:[*]:c:1
4. Unknown FCFP_2 feature: 1315416442: [*][C@H]1[*][*]O[C@H]1n(:[*]):[*]

Feature Contribution

Top features for positive contribution

Fingerprint	Bit/Smiles	Feature Structure	Score
FCFP_2	-1143715940	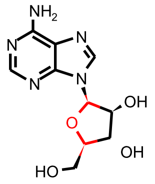 [*][C@@H]1[*][*][C@H]([*])O1	0.095

FCFP_2	3	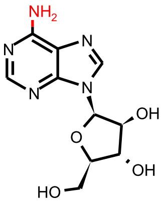 [*]O	0.074
FCFP_2	17	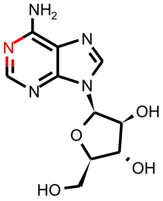 [*]:n:[*]	0.044
**Top Features for negative contribution**			
**Fingerprint**	**Bit/Smiles**	**Feature Structure**	**Score**
FCFP_2	1	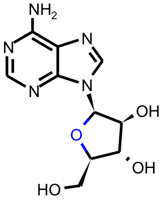 [*]O[*]	-0.080
FCFP_2	16	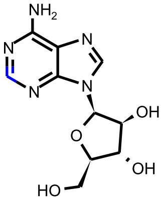 [*]:[cH]:[*]	-0.051
FCFP_2	-1272709286	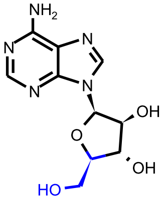 [*]C([*])CO	-0.049

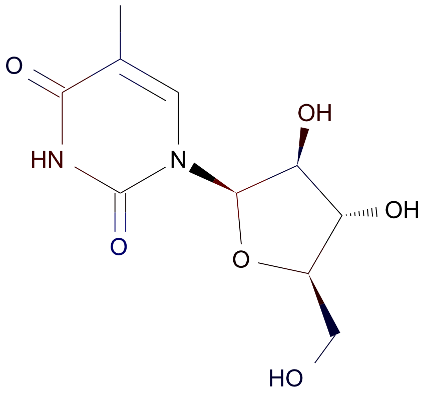

$C_{10}H_{14}N_2O_6$
Molecular Weight: 258.22795
ALogP: -2.005
Rotatable Bonds: 2
Acceptors: 6
Donors: 4

Model Prediction

Prediction: 0.095
Unit: g/kg_body_weight
Mahalanobis Distance: 8.873
Mahalanobis Distance p-value: 0.00211

Mahalanobis Distance: The Mahalanobis distance (MD) is a generalization of the Euclidean distance that accounts for correlations among the X properties. It is calculated as the distance to the center of the training data. The larger the MD, the less trustworthy the prediction.
Mahalanobis Distance p-value: The p-value gives the fraction of training data with an MD greater than or equal to the one for the given sample, assuming normally distributed data. The smaller the p-value, the less trustworthy the prediciton. For highly non-normal X properties (e.g., fingerprints), the MD p-value is wildly inaccurate.

Structural Similar Compounds

Name	L-ASORBIC ACID	METHYLDOPA SESQUIHYDRATE	HYDROCHLOROTHIAZIDE
Structure	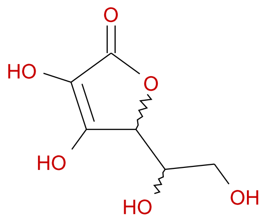	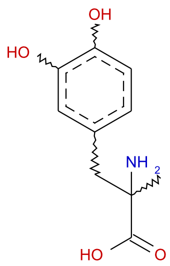	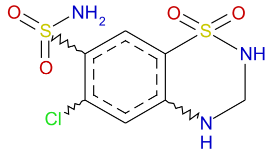
Actual Endpoint (-log C)	1.89364	2.94452	3.56001
Predicted Endpoint (-log C)	2.56611	2.32114	3.55045
Distance	0.435	0.644	0.733
Reference	NCI/NTP TR-247	NCI/NTP TR-348	NCI/NTP TR-357

Model Applicability

Unknown features are fingerprint features in the query molecule, but not found in the training set.

1. All properties and OPS components are within expected ranges.

Feature Contribution

Top features for positive contribution

Fingerprint	Bit/Smiles	Feature Structure	Score
FCFP_2	-885550502	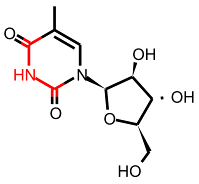 [*]C(=[*])NC(=[*])[*]	0.115

FCFP_2	-1143715940	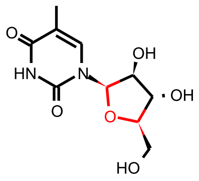 [*][C@@H]1[*][*][C@H]([*])O1	0.095
FCFP_2	3	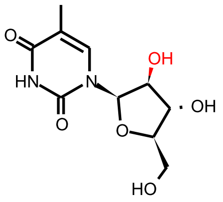 [*]O	0.074

Top Features for negative contribution

Fingerprint	Bit/Smiles	Feature Structure	Score
FCFP_2	1872154524	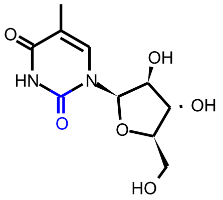 [*]C(=O)[*]	-0.105
FCFP_2	1	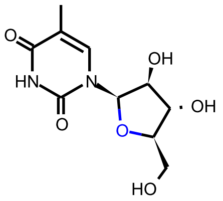 [*]O[*]	-0.080
FCFP_2	-1272709286	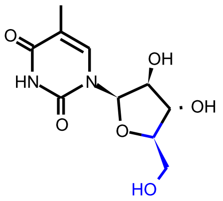 [*]C([*])CO	-0.049

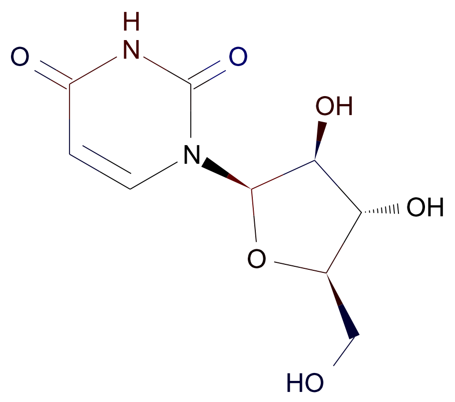

$C_9H_{12}N_2O_6$
Molecular Weight: 244.20137
ALogP: -2.451
Rotatable Bonds: 2
Acceptors: 6
Donors: 4

Model Prediction

Prediction: 0.094
Unit: g/kg_body_weight
Mahalanobis Distance: 8.623
Mahalanobis Distance p-value: 0.00448

Mahalanobis Distance: The Mahalanobis distance (MD) is a generalization of the Euclidean distance that accounts for correlations among the X properties. It is calculated as the distance to the center of the training data. The larger the MD, the less trustworthy the prediction.
Mahalanobis Distance p-value: The p-value gives the fraction of training data with an MD greater than or equal to the one for the given sample, assuming normally distributed data. The smaller the p-value, the less trustworthy the prediciton. For highly non-normal X properties (e.g., fingerprints), the MD p-value is wildly inaccurate.

Structural Similar Compounds

Name	L-ASORBIC ACID	METHYLDOPA SESQUIHYDRATE	D-MANNITOL
Structure	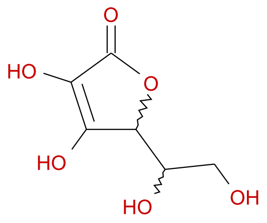	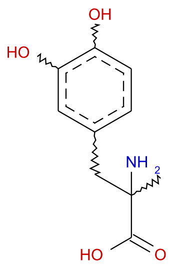	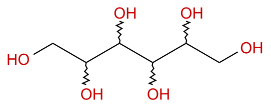
Actual Endpoint (-log C)	1.89364	2.94452	1.9083
Predicted Endpoint (-log C)	2.56611	2.32114	2.52388
Distance	0.417	0.663	0.711
Reference	NCI/NTP TR-247	NCI/NTP TR-348	NCI/NTP TR-236

Model Applicability

Unknown features are fingerprint features in the query molecule, but not found in the training set.

1. All properties and OPS components are within expected ranges.

Feature Contribution

Top features for positive contribution

Fingerprint	Bit/Smiles	Feature Structure	Score
FCFP_2	-885550502	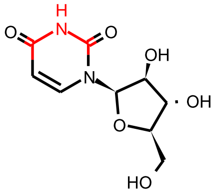 [*]C(=[*])NC(=[*])[*]	0.115

FCFP_2	-1143715940	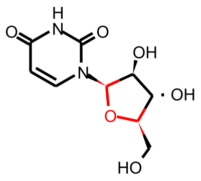 [*][C@@H]1[*][*][C@H] ([*])O1	0.095
FCFP_2	3	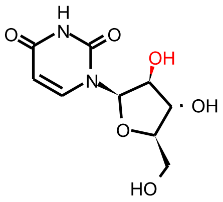 [*]O	0.074

Top Features for negative contribution

Fingerprint	Bit/Smiles	Feature Structure	Score
FCFP_2	1872154524	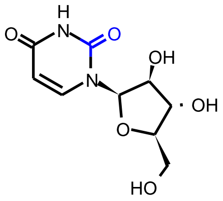 [*]C(=O)[*]	-0.105
FCFP_2	1	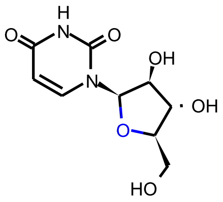 [*]O[*]	-0.080
FCFP_2	-1272709286	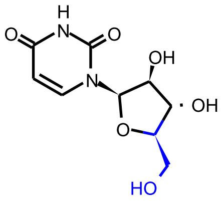 [*]C([*])CO	-0.049

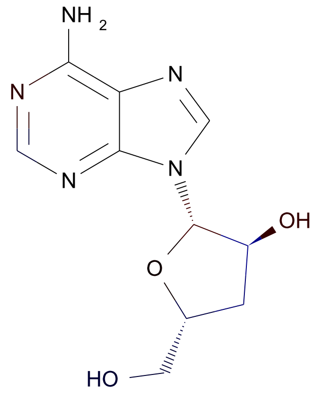

$C_{10}H_{13}N_5O_3$
Molecular Weight: 251.24191
ALogP: -1.308
Rotatable Bonds: 2
Acceptors: 7
Donors: 3

Model Prediction

Prediction: 0.155
Unit: g/kg_body_weight
Mahalanobis Distance: 11.019
Mahalanobis Distance p-value: 8.17e-007

Mahalanobis Distance: The Mahalanobis distance (MD) is a generalization of the Euclidean distance that accounts for correlations among the X properties. It is calculated as the distance to the center of the training data. The larger the MD, the less trustworthy the prediction.
Mahalanobis Distance p-value: The p-value gives the fraction of training data with an MD greater than or equal to the one for the given sample, assuming normally distributed data. The smaller the p-value, the less trustworthy the prediciton. For highly non-normal X properties (e.g., fingerprints), the MD p-value is wildly inaccurate.

Structural Similar Compounds

Name	TRIAMTERENE	MELAMINE	50%1,4,5,8-TETRAAMINOANTHRAQUIONONE + DERIVATIVES
Structure	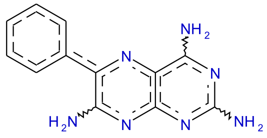	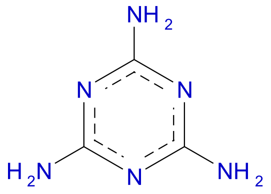	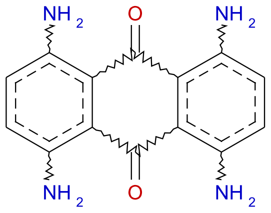
Actual Endpoint (-log C)	4.00564	2.40182	3.0764
Predicted Endpoint (-log C)	3.1992	3.01288	3.08142
Distance	0.639	0.707	0.715
Reference	NCI/NTP TR-420	NCI/NTP TR-245	NCI/NTP TR-299

Model Applicability

Unknown features are fingerprint features in the query molecule, but not found in the training set.

1. All properties and OPS components are within expected ranges.
2. Unknown FCFP_2 feature: -124685461: [*]:n:c:n:[*]
3. Unknown FCFP_2 feature: -306856457: [*]C([*])n1:[c](:[*]):[*]:[*]:c:1
4. Unknown FCFP_2 feature: 1315416442: [*][C@H]1[*][*]O[C@H]1n(:[*]):[*]

Feature Contribution

Top features for positive contribution

Fingerprint	Bit/Smiles	Feature Structure	Score
FCFP_2	-1143715940	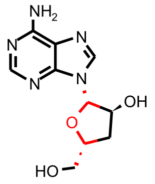 [*][C@@H]1[*][*][C@H]([*])O1	0.095

FCFP_2	3	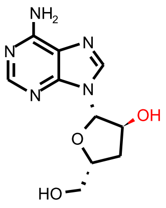 [*]O	0.074
FCFP_2	17	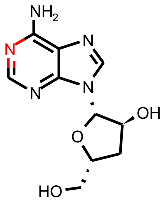 [*]:n:[*]	0.044

Top Features for negative contribution

Fingerprint	Bit/Smiles	Feature Structure	Score
FCFP_2	-1272798659	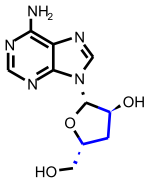 [*][C@@H]1[*][*]CC1	-0.111
FCFP_2	1	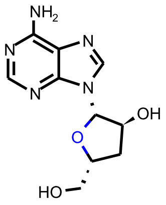 [*]O[*]	-0.080
FCFP_2	16	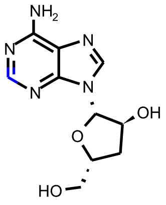 [*]:[cH]:[*]	-0.051

Ribavirin

TOPKAT_Rat_Maximum_Tolerated_Dose_Feed

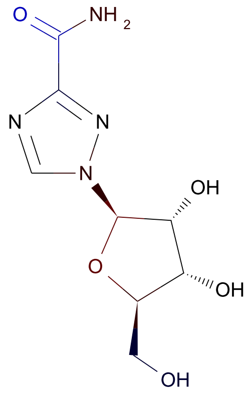

$C_8H_{12}N_4O_5$

Molecular Weight: 244.20467

ALogP: -2.745

Rotatable Bonds: 3

Acceptors: 7

Donors: 4

Model Prediction

Prediction: 0.154

Unit: g/kg_body_weight

Mahalanobis Distance: 9.872

Mahalanobis Distance p-value: 7.13e-005

Mahalanobis Distance: The Mahalanobis distance (MD) is a generalization of the Euclidean distance that accounts for correlations among the X properties. It is calculated as the distance to the center of the training data. The larger the MD, the less trustworthy the prediction.

Mahalanobis Distance p-value: The p-value gives the fraction of training data with an MD greater than or equal to the one for the given sample, assuming normally distributed data. The smaller the p-value, the less trustworthy the prediciton. For highly non-normal X properties (e.g., fingerprints), the MD p-value is wildly inaccurate.

Structural Similar Compounds

Name	L-ASORBIC ACID	METHYLDOPA SESQUIHYDRATE	HYDROCHLOROTHIAZIDE
Structure	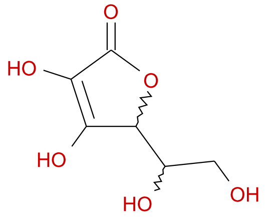	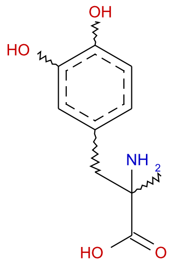	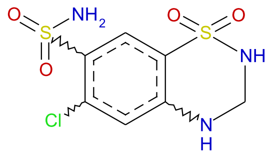
Actual Endpoint (-log C)	1.89364	2.94452	3.56001
Predicted Endpoint (-log C)	2.56611	2.32114	3.55045
Distance	0.651	0.683	0.740
Reference	NCI/NTP TR-247	NCI/NTP TR-348	NCI/NTP TR-357

Model Applicability

Unknown features are fingerprint features in the query molecule, but not found in the training set.

1. All properties and OPS components are within expected ranges.
2. Unknown FCFP_2 feature: -2049666792: [*]C([*])n1:n:[*]:[*]:c:1
3. Unknown FCFP_2 feature: -124685461: [*]:n:c:n:[*]
4. Unknown FCFP_2 feature: 1315416442: [*][C@H]1[*][*]O[C@H]1n(:[*]):[*]

Feature Contribution

Top features for positive contribution			
**Fingerprint**	**Bit/Smiles**	**Feature Structure**	**Score**
FCFP_2	-1143715940	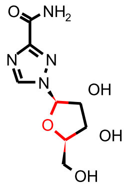 [*][C@@H]1[*][*][C@H]([*])O1	0.095

FCFP_2	3	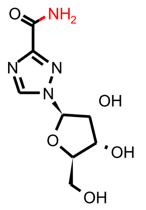 [*]O	0.074
FCFP_2	17	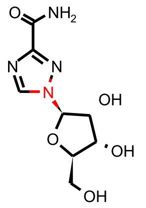 [*]:n:[*]	0.044
**Top Features for negative contribution**			
**Fingerprint**	**Bit/Smiles**	**Feature Structure**	**Score**
FCFP_2	1872154524	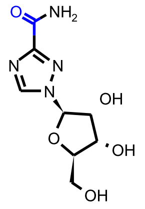 [*]C(=O)[*]	-0.105
FCFP_2	1	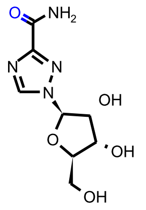 [*]O[*]	-0.080
FCFP_2	16	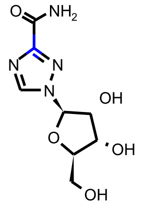 [*]:[cH]:[*]	-0.051

AND Enantiomer

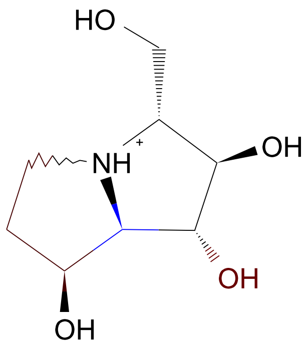

$C_8H_{16}NO_4$

Molecular Weight: 190.21693

ALogP: -3.556

Rotatable Bonds: 1

Acceptors: 4

Donors: 5

Model Prediction

Prediction: 0.261

Unit: g/kg_body_weight

Mahalanobis Distance: 15.946

Mahalanobis Distance p-value: 2.66e-014

Mahalanobis Distance: The Mahalanobis distance (MD) is a generalization of the Euclidean distance that accounts for correlations among the X properties. It is calculated as the distance to the center of the training data. The larger the MD, the less trustworthy the prediction.
Mahalanobis Distance p-value: The p-value gives the fraction of training data with an MD greater than or equal to the one for the given sample, assuming normally distributed data. The smaller the p-value, the less trustworthy the prediciton. For highly non-normal X properties (e.g., fingerprints), the MD p-value is wildly inaccurate.

Structural Similar Compounds

Name	HC RED 3	2,4-DIAMINOPHENOL DIHYDROCHLORIDE	AMPICILLIN TRIHYDRATE
Structure	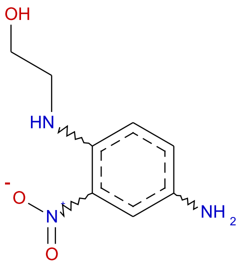	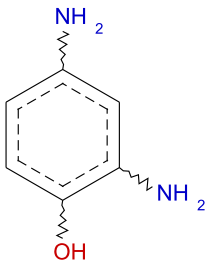	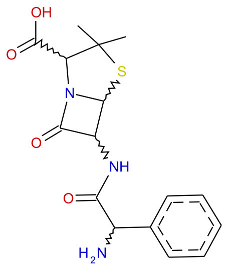
Actual Endpoint (-log C)	2.59592	3.69598	2.36724
Predicted Endpoint (-log C)	3.285	3.37438	2.27651
Distance	1.095	1.138	1.197
Reference	NCI/NTP TR-281	NCI/NTP TR-401	NCI/NTP TR-318

Model Applicability

Unknown features are fingerprint features in the query molecule, but not found in the training set.

1. ALogP out of range. Value: -3.556. Training min, max, mean, SD: -2.297, 8.698, 1.9556, 1.637.
2. Num_H_Donors out of range. Value: 5. Training min, max, mean, SD: 0, 3, 0.4375, 0.8311.
3. OPS PC9 out of range. Value: -2.7771. Training min, max, SD, explained variance: -2.7086, 2.9267, 1.019, 0.0321.
4. Unknown FCFP_2 feature: 10: [*][NH+]([*])[*]
5. Unknown FCFP_2 feature: -1853714334: [*][C@@H]1[*][*][C@H]2[*][*]C[NH+]12
6. Unknown FCFP_2 feature: -1817836174: [*][C@H]1[*][*][NH+]2[*][*][C@H]([*])C12
7. Unknown FCFP_2 feature: 1155241219: [*][NH+]1[*][*]CC1

Feature Contribution

Top features for positive contribution			
**Fingerprint**	**Bit/Smiles**	**Feature Structure**	**Score**

FCFP_2	3	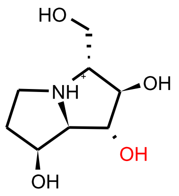 [*]O	0.104
FCFP_2	-1272798659	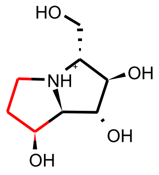 [*][C@@H]1[*][*]CC1	0.070

Top Features for negative contribution

Fingerprint	Bit/Smiles	Feature Structure	Score
FCFP_2	0	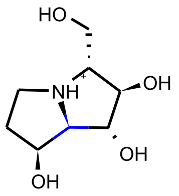 [*]C([*])[*]	-0.290

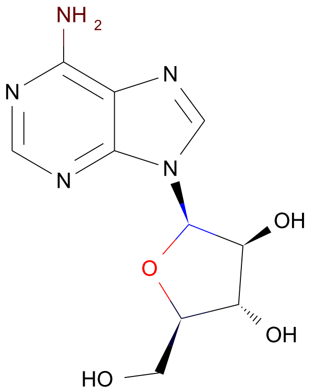

$C_{10}H_{13}N_5O_4$
Molecular Weight: 267.24132
ALogP: -1.881
Rotatable Bonds: 2
Acceptors: 8
Donors: 4

Model Prediction

Prediction: 0.001
Unit: g/kg_body_weight
Mahalanobis Distance: 13.179
Mahalanobis Distance p-value: 1.86e-010

Mahalanobis Distance: The Mahalanobis distance (MD) is a generalization of the Euclidean distance that accounts for correlations among the X properties. It is calculated as the distance to the center of the training data. The larger the MD, the less trustworthy the prediction.
Mahalanobis Distance p-value: The p-value gives the fraction of training data with an MD greater than or equal to the one for the given sample, assuming normally distributed data. The smaller the p-value, the less trustworthy the prediciton. For highly non-normal X properties (e.g., fingerprints), the MD p-value is wildly inaccurate.

Structural Similar Compounds

Name	AMPICILLIN TRIHYDRATE	HC RED 3	SULFISOOXAZOLE
Structure	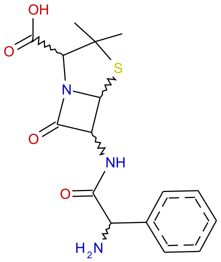	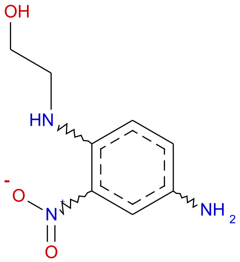	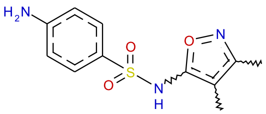
Actual Endpoint (-log C)	2.36724	2.59592	2.82494
Predicted Endpoint (-log C)	2.27651	3.285	3.0705
Distance	0.831	0.961	1.101
Reference	NCI/NTP TR-318	NCI/NTP TR-281	NCI/NTP TR-138

Model Applicability

Unknown features are fingerprint features in the query molecule, but not found in the training set.

1. Num_H_Donors out of range. Value: 4. Training min, max, mean, SD: 0, 3, 0.4375, 0.8311.
2. Num_H_Acceptors out of range. Value: 8. Training min, max, mean, SD: 0, 6, 1.6146, 1.644.
3. Molecular_PolarSASA out of range. Value: 238.62. Training min, max, mean, SD: 0, 223.97, 50.816, 55.15.
4. Molecular_PolarSurfaceArea out of range. Value: 139.54. Training min, max, mean, SD: 0, 138.03, 28.978, 32.1.
5. Unknown FCFP_2 feature: -124685461: [*]:n:c:n:[*]
6. Unknown FCFP_2 feature: -1564473960: [*]n1:[*]:[*]:[c](:[*]):[c]:1:n:[*]
7. Unknown FCFP_2 feature: -306856457: [*]C([*])n1:[c](:[*]):[*]:[*]:c:1
8. Unknown FCFP_2 feature: 1315416442: [*][C@H]1[*][*]O[C@H]1n(:[*]):[*]

Feature Contribution

Top features for positive contribution

Fingerprint	Bit/Smiles	Feature Structure	Score

FCFP_2	1	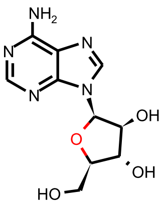<br>[*]O[*]	0.511
FCFP_2	3	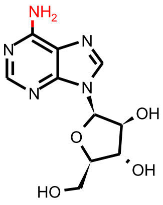<br>[*]O	0.104
**Top Features for negative contribution**			
**Fingerprint**	**Bit/Smiles**	**Feature Structure**	**Score**
FCFP_2	0	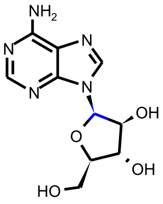<br>[*]C([*])[*]	-0.290
FCFP_2	-1143715940	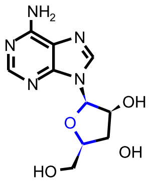<br>[*][C@@H]1[*][*][C@H]([*])O1	-0.053

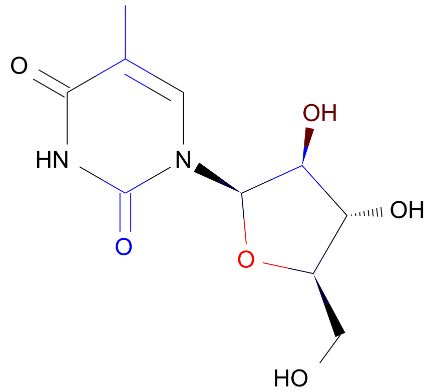

$C_{10}H_{14}N_2O_6$
Molecular Weight: 258.22795
ALogP: -2.005
Rotatable Bonds: 2
Acceptors: 6
Donors: 4

Model Prediction

Prediction: 0.060
Unit: g/kg_body_weight
Mahalanobis Distance: 12.293
Mahalanobis Distance p-value: 3.39e-009

Mahalanobis Distance: The Mahalanobis distance (MD) is a generalization of the Euclidean distance that accounts for correlations among the X properties. It is calculated as the distance to the center of the training data. The larger the MD, the less trustworthy the prediction.
Mahalanobis Distance p-value: The p-value gives the fraction of training data with an MD greater than or equal to the one for the given sample, assuming normally distributed data. The smaller the p-value, the less trustworthy the prediciton. For highly non-normal X properties (e.g., fingerprints), the MD p-value is wildly inaccurate.

Structural Similar Compounds

Name	AMPICILLIN TRIHYDRATE	HC RED 3	PENICILLIN VK
Structure	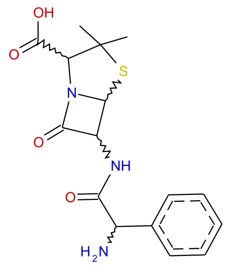	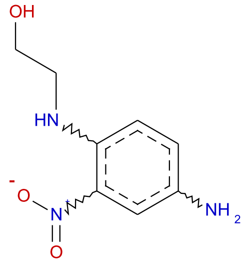	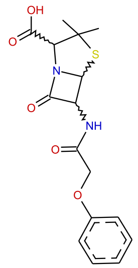
Actual Endpoint (-log C)	2.36724	2.59592	2.54455
Predicted Endpoint (-log C)	2.27651	3.285	3.9702
Distance	0.681	0.847	0.930
Reference	NCI/NTP TR-318	NCI/NTP TR-281	NCI/NTP TR-336

Model Applicability

Unknown features are fingerprint features in the query molecule, but not found in the training set.

1. Num_H_Donors out of range. Value: 4. Training min, max, mean, SD: 0, 3, 0.4375, 0.8311.

Feature Contribution

Top features for positive contribution

Fingerprint	Bit/Smiles	Feature Structure	Score
FCFP_2	1	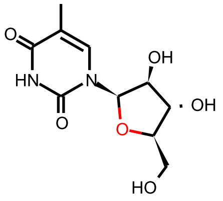 [*]O[*]	0.511

FCFP_2	3	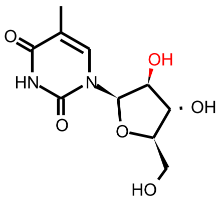 [*]O	0.104
**Top Features for negative contribution**			
**Fingerprint**	**Bit/Smiles**	**Feature Structure**	**Score**
FCFP_2	136597326	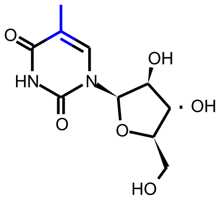 [*]C(=[*])C	-0.489
FCFP_2	1872154524	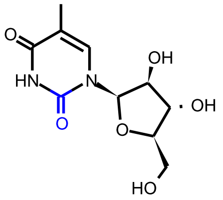 [*]C(=O)[*]	-0.307
FCFP_2	0	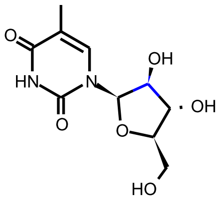 [*]C([*])[*]	-0.290

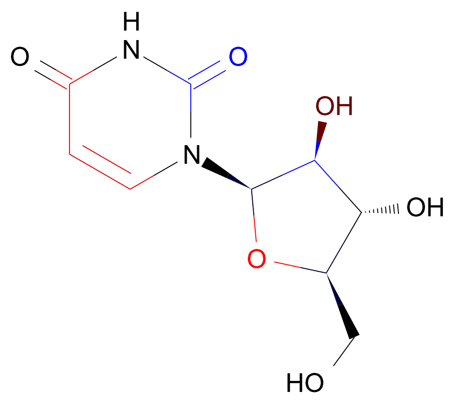

$C_9H_{12}N_2O_6$

Molecular Weight: 244.20137

ALogP: -2.451

Rotatable Bonds: 2

Acceptors: 6

Donors: 4

Model Prediction

Prediction: 0.014

Unit: g/kg_body_weight

Mahalanobis Distance: 12.800

Mahalanobis Distance p-value: 6.44e-010

Mahalanobis Distance: The Mahalanobis distance (MD) is a generalization of the Euclidean distance that accounts for correlations among the X properties. It is calculated as the distance to the center of the training data. The larger the MD, the less trustworthy the prediction.
Mahalanobis Distance p-value: The p-value gives the fraction of training data with an MD greater than or equal to the one for the given sample, assuming normally distributed data. The smaller the p-value, the less trustworthy the prediciton. For highly non-normal X properties (e.g., fingerprints), the MD p-value is wildly inaccurate.

Structural Similar Compounds

Name	AMPICILLIN TRIHYDRATE	HC RED 3	PENICILLIN VK
Structure	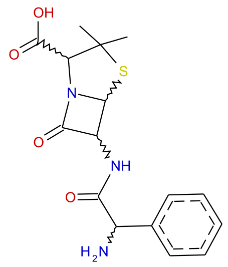	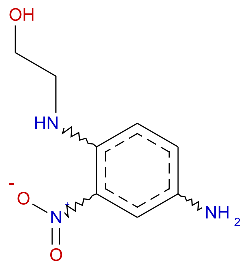	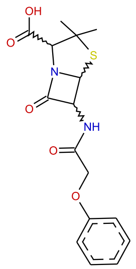
Actual Endpoint (-log C)	2.36724	2.59592	2.54455
Predicted Endpoint (-log C)	2.27651	3.285	3.9702
Distance	0.713	0.861	0.983
Reference	NCI/NTP TR-318	NCI/NTP TR-281	NCI/NTP TR-336

Model Applicability

Unknown features are fingerprint features in the query molecule, but not found in the training set.

1. ALogP out of range. Value: -2.451. Training min, max, mean, SD: -2.297, 8.698, 1.9556, 1.637.
2. Num_H_Donors out of range. Value: 4. Training min, max, mean, SD: 0, 3, 0.4375, 0.8311.

Feature Contribution

Top features for positive contribution

Fingerprint	Bit/Smiles	Feature Structure	Score
FCFP_2	1	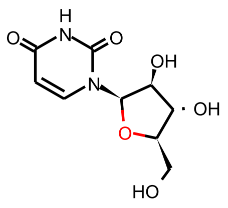 [*]O[*]	0.511

FCFP_2	451847724	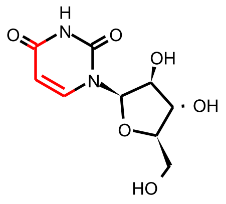 [*]\C=C/C(=[*])[*]	0.225
FCFP_2	3	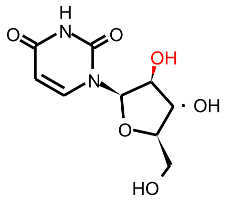 [*]O	0.104

Top Features for negative contribution

**Fingerprint**	**Bit/Smiles**	**Feature Structure**	**Score**
FCFP_2	1872154524	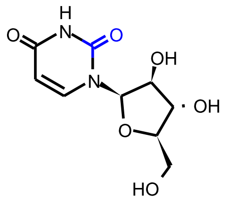 [*]C(=O)[*]	-0.307
FCFP_2	0	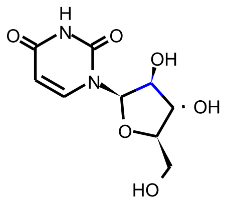 [*]C([*])[*]	-0.290
FCFP_2	-1143715940	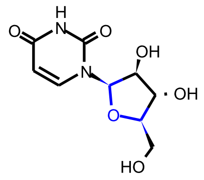 [*][C@@H]1[*][*][C@H]([*])O1	-0.053

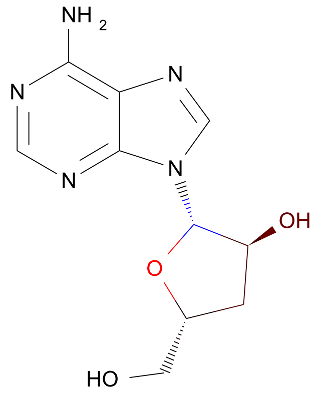

$C_{10}H_{13}N_5O_3$
Molecular Weight: 251.24191
ALogP: -1.308
Rotatable Bonds: 2
Acceptors: 7
Donors: 3

Structural Similar Compounds

Name	AMPICILLIN TRIHYDRATE	HC RED 3	SULFISOOXAZOLE
Structure	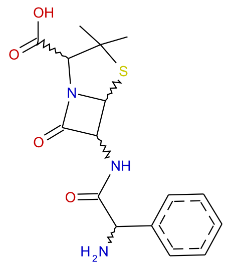	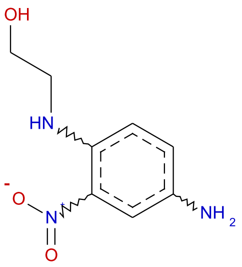	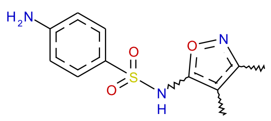
Actual Endpoint (-log C)	2.36724	2.59592	2.82494
Predicted Endpoint (-log C)	2.27651	3.285	3.0705
Distance	0.782	0.782	0.849
Reference	NCI/NTP TR-318	NCI/NTP TR-281	NCI/NTP TR-138

Model Prediction

Prediction: 0.002
Unit: g/kg_body_weight
Mahalanobis Distance: 11.838
Mahalanobis Distance p-value: 1.51e-008

Mahalanobis Distance: The Mahalanobis distance (MD) is a generalization of the Euclidean distance that accounts for correlations among the X properties. It is calculated as the distance to the center of the training data. The larger the MD, the less trustworthy the prediction.
Mahalanobis Distance p-value: The p-value gives the fraction of training data with an MD greater than or equal to the one for the given sample, assuming normally distributed data. The smaller the p-value, the less trustworthy the prediciton. For highly non-normal X properties (e.g., fingerprints), the MD p-value is wildly inaccurate.

Model Applicability

Unknown features are fingerprint features in the query molecule, but not found in the training set.

1. Num_H_Acceptors out of range. Value: 7. Training min, max, mean, SD: 0, 6, 1.6146, 1.644.
2. Unknown FCFP_2 feature: -124685461: [*]:n:c:n:[*]
3. Unknown FCFP_2 feature: -1564473960: [*]n1:[*]:[*]:[c](:[*]):[c]:1:n:[*]
4. Unknown FCFP_2 feature: -306856457: [*]C([*])n1:[c](:[*]):[*]:[*]:c:1
5. Unknown FCFP_2 feature: 1315416442: [*][C@H]1[*][*]O[C@H]1n(:[*]):[*]

Feature Contribution

Top features for positive contribution

Fingerprint	Bit/Smiles	Feature Structure	Score
FCFP_2	1	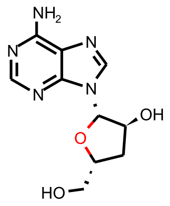 [*]O[*]	0.511

FCFP_2	3	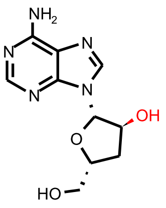 [*]O	0.104
FCFP_2	-1272798659	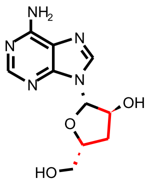 [*][C@@H]1[*][*]CC1	0.070
**Top Features for negative contribution**			
**Fingerprint**	**Bit/Smiles**	**Feature Structure**	**Score**
FCFP_2	0	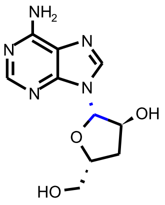 [*]C([*])[*]	-0.290
FCFP_2	-1143715940	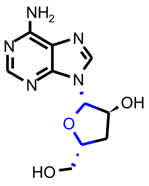 [*][C@@H]1[*][*][C@H]([*])O1	-0.053

Ribavirin

TOPKAT_Rat_Maximum_Tolerated_Dose_Gavage

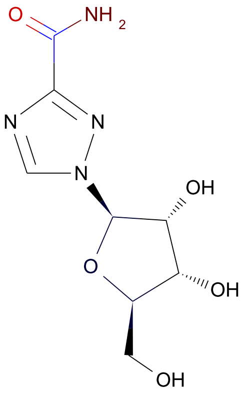

$C_8H_{12}N_4O_5$
Molecular Weight: 244.20467
ALogP: -2.745
Rotatable Bonds: 3
Acceptors: 7
Donors: 4

Structural Similar Compounds

Name	AMPICILLIN TRIHYDRATE	HC RED 3	PENICILLIN VK
Structure	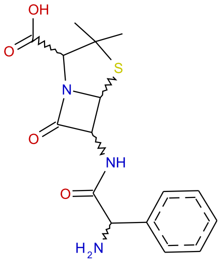	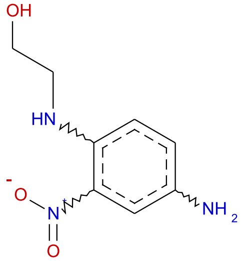	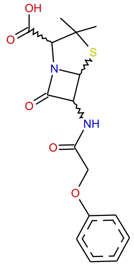
Actual Endpoint (-log C)	2.36724	2.59592	2.54455
Predicted Endpoint (-log C)	2.27651	3.285	3.9702
Distance	0.702	0.898	1.075
Reference	NCI/NTP TR-318	NCI/NTP TR-281	NCI/NTP TR-336

Model Prediction

Prediction: 0.024
Unit: g/kg_body_weight
Mahalanobis Distance: 12.098
Mahalanobis Distance p-value: 6.42e-009

Mahalanobis Distance: The Mahalanobis distance (MD) is a generalization of the Euclidean distance that accounts for correlations among the X properties. It is calculated as the distance to the center of the training data. The larger the MD, the less trustworthy the prediction.
Mahalanobis Distance p-value: The p-value gives the fraction of training data with an MD greater than or equal to the one for the given sample, assuming normally distributed data. The smaller the p-value, the less trustworthy the prediciton. For highly non-normal X properties (e.g., fingerprints), the MD p-value is wildly inaccurate.

Model Applicability

Unknown features are fingerprint features in the query molecule, but not found in the training set.

1. ALogP out of range. Value: -2.745. Training min, max, mean, SD: -2.297, 8.698, 1.9556, 1.637.
2. Num_H_Donors out of range. Value: 4. Training min, max, mean, SD: 0, 3, 0.4375, 0.8311.
3. Num_H_Acceptors out of range. Value: 7. Training min, max, mean, SD: 0, 6, 1.6146, 1.644.
4. Molecular_PolarSASA out of range. Value: 251.23. Training min, max, mean, SD: 0, 223.97, 50.816, 55.15.
5. Molecular_PolarSurfaceArea out of range. Value: 143.72. Training min, max, mean, SD: 0, 138.03, 28.978, 32.1.
6. Unknown FCFP_2 feature: -2049666792: [*]C([*])n1:n:[*]:[*]:c:1
7. Unknown FCFP_2 feature: -1539162406: [*]C(=[*])[c]1:n:[*]:[*]:n:1
8. Unknown FCFP_2 feature: -124685461: [*]:n:c:n:[*]
9. Unknown FCFP_2 feature: 1315416442: [*][C@H]1[*][*]O[C@H]1n(:[*]):[*]

Feature Contribution

Top features for positive contribution			
Fingerprint	Bit/Smiles	Feature Structure	Score

FCFP_2	1	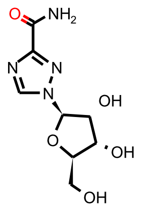 [*]O[*]	0.511
FCFP_2	3	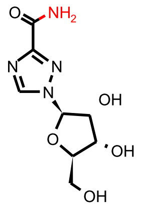 [*]O	0.104

Top Features for negative contribution

Fingerprint	Bit/Smiles	Feature Structure	Score
FCFP_2	1872154524	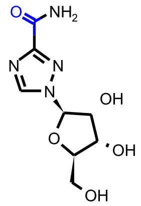 [*]C(=O)[*]	-0.307
FCFP_2	0	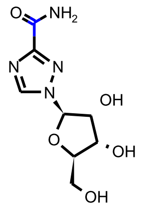 [*]C([*])[*]	-0.290
FCFP_2	-1143715940	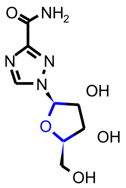 [*][C@@H]1[*][*][C@H]([*])O1	-0.053

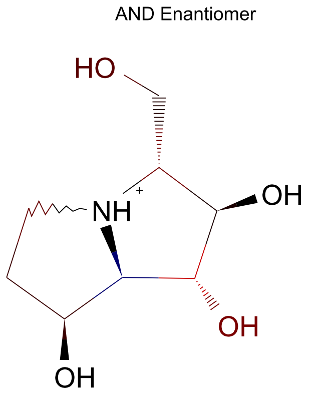

$C_8H_{16}NO_4$

Molecular Weight: 190.21693

ALogP: -3.556

Rotatable Bonds: 1

Acceptors: 4

Donors: 5

Model Prediction

Prediction: 0.778

Unit: g/kg_body_weight

Mahalanobis Distance: 22.451

Mahalanobis Distance p-value: 3.06e-018

Mahalanobis Distance: The Mahalanobis distance (MD) is a generalization of the Euclidean distance that accounts for correlations among the X properties. It is calculated as the distance to the center of the training data. The larger the MD, the less trustworthy the prediction.
Mahalanobis Distance p-value: The p-value gives the fraction of training data with an MD greater than or equal to the one for the given sample, assuming normally distributed data. The smaller the p-value, the less trustworthy the prediciton. For highly non-normal X properties (e.g., fingerprints), the MD p-value is wildly inaccurate.

Structural Similar Compounds

Name	GLUCOSE	2-AMINO-2-(HYDROXYMETHYL)-1;3-PROPANEDIOL	L-ASCORBIC ACID
Structure	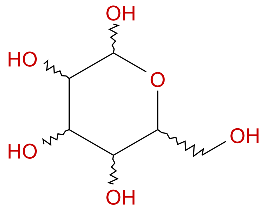	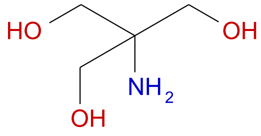	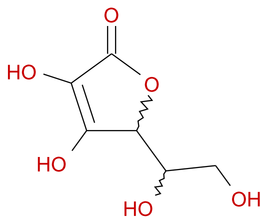
Actual Endpoint (-log C)	0.844	1.312	1.17
Predicted Endpoint (-log C)	1.68269	2.10886	2.00353
Distance	0.652	0.663	0.738
Reference	85AIAL -;39;73	BCFAAI 110;653;71	OYYAA2 19;323;80

Model Applicability

Unknown features are fingerprint features in the query molecule, but not found in the training set.

1. All properties and OPS components are within expected ranges.
2. Unknown ECFP_2 feature: 1976330679: [*][NH+]([*])[*]
3. Unknown ECFP_2 feature: -1693770146: [*][C@@H]1[*][*][C@H]2[*][*]C[NH+]12
4. Unknown ECFP_2 feature: -1395316370: [*][C@H]1[*][*][NH+]2[*][*][C@H]([*])C12
5. Unknown ECFP_2 feature: -705668329: [*]C[C@@H]1[C@@H]([*])[*][*][NH+]1[*]
6. Unknown ECFP_2 feature: -244159614: [*][NH+]1[*][*]CC1
7. Unknown FCFP_6 feature: 10: [*][NH+]([*])[*]
8. Unknown FCFP_6 feature: -1853714334: [*][C@@H]1[*][*][C@H]2[*][*]C[NH+]12
9. Unknown FCFP_6 feature: -1817836174: [*][C@H]1[*][*][NH+]2[*][*][C@H]([*])C12
10. Unknown FCFP_6 feature: 1155241219: [*][NH+]1[*][*]CC1

Feature Contribution

Top features for positive contribution

Fingerprint	Bit/Smiles	Feature Structure	Score

FCFP_6	-1272709286	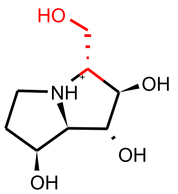 [*]C([*])CO	0.115
FCFP_6	1070061035	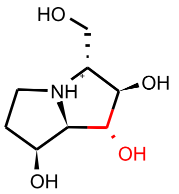 [*]C([*])O	0.100
ECFP_6	-1331450522	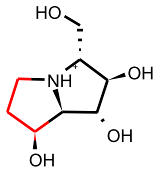 [*][C@@H]1[*][*]CC1	0.086
**Top Features for negative contribution**			
**Fingerprint**	**Bit/Smiles**	**Feature Structure**	**Score**
FCFP_6	3	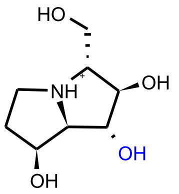 [*]O	-0.107
FCFP_6	0	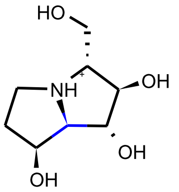 [*]C([*])[*]	-0.079

ECFP_6	-167460056	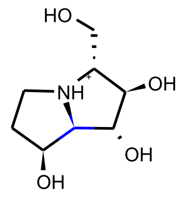<br>[*]C([*])[*]	-0.057

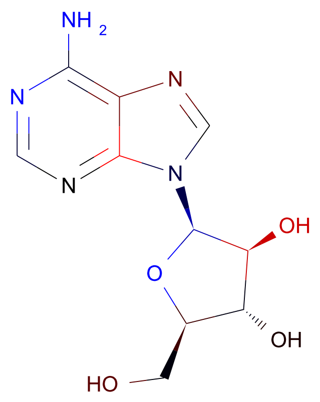

$C_{10}H_{13}N_5O_4$
Molecular Weight: 267.24132
ALogP: -1.881
Rotatable Bonds: 2
Acceptors: 8
Donors: 4

Structural Similar Compounds

Name	6-MERCAPTOPURINE RIBOSIDE	TUBERCIDIN	7H-PYRROLO[2;3-d]PYRIMIDIN-4-OL; 7-.beta.-d-RIBOFURANOSYL-
Structure	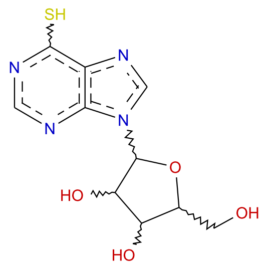	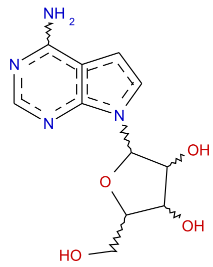	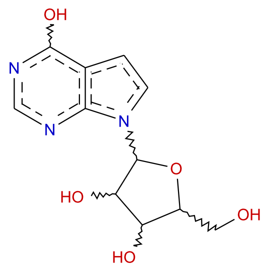
Actual Endpoint (-log C)	2.5	4.221	4.012
Predicted Endpoint (-log C)	2.54632	2.53511	3.00148
Distance	0.286	0.316	0.422
Reference	NIIRDN 6;853;82	CNREA8 29;116;69	CNREA8 29;116;69

Model Prediction

Prediction: 1.119
Unit: g/kg_body_weight
Mahalanobis Distance: 17.894
Mahalanobis Distance p-value: 0.00118

Mahalanobis Distance: The Mahalanobis distance (MD) is a generalization of the Euclidean distance that accounts for correlations among the X properties. It is calculated as the distance to the center of the training data. The larger the MD, the less trustworthy the prediction.
Mahalanobis Distance p-value: The p-value gives the fraction of training data with an MD greater than or equal to the one for the given sample, assuming normally distributed data. The smaller the p-value, the less trustworthy the prediciton. For highly non-normal X properties (e.g., fingerprints), the MD p-value is wildly inaccurate.

Model Applicability

Unknown features are fingerprint features in the query molecule, but not found in the training set.

1. All properties and OPS components are within expected ranges.
2. Unknown FCFP_6 feature: 16: [*]:[cH]:[*]
3. Unknown FCFP_6 feature: 1747237384: [*][c](:[*]):n:[cH]:[*]
4. Unknown FCFP_6 feature: -124685461: [*]:n:[cH]:n:[*]
5. Unknown FCFP_6 feature: -1564473960: [*]n1:[*]:[*]:[c](:[*]):[c]:1:n:[*]
6. Unknown FCFP_6 feature: -1151884458: [*]:n:[c](N):[c](:[*]):[*]
7. Unknown FCFP_6 feature: 1069584379: [*]:[c](:[*])N
8. Unknown FCFP_6 feature: -306856457: [*]C([*])n1:[cH]:[*]:[*]:[c]:1:[*]
9. Unknown FCFP_6 feature: 1315416442: [*][C@H]1[*][*]O[C@H]1n(:[*]):[*]

Feature Contribution

Top features for positive contribution			
**Fingerprint**	**Bit/Smiles**	**Feature Structure**	**Score**

ECFP_6	642810091	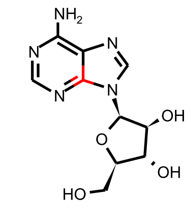 [*]:[c](:[*]):[*]	0.281
FCFP_6	-1272709286	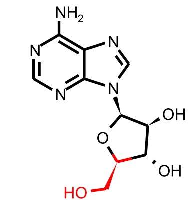 [*]C([*])CO	0.115
FCFP_6	178336375	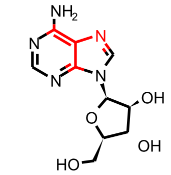 [*][c](:[*]):[c]1:n:[*]:[*]:[c]:1:[*]	0.109

Top Features for negative contribution

**Fingerprint**	**Bit/Smiles**	**Feature Structure**	**Score**
ECFP_6	683445015	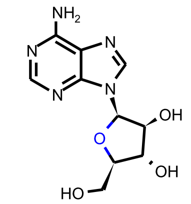 [*]O[*]	-0.266
ECFP_6	655739385	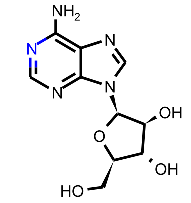 [*]:n:[*]	-0.239

FCFP_6	3	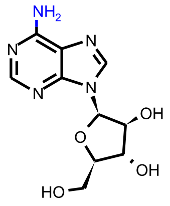 [*]O	-0.107

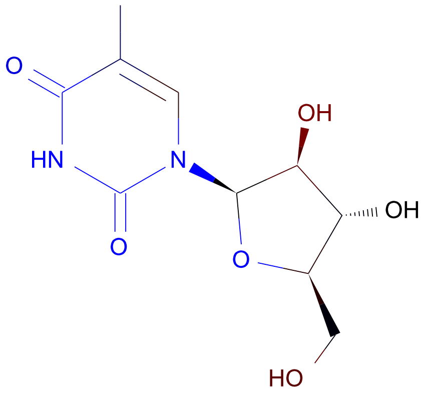

$C_{10}H_{14}N_2O_6$
Molecular Weight: 258.22795
ALogP: -2.005
Rotatable Bonds: 2
Acceptors: 6
Donors: 4

Model Prediction

Prediction: 6.173
Unit: g/kg_body_weight
Mahalanobis Distance: 22.213
Mahalanobis Distance p-value: 3.41e-017

Mahalanobis Distance: The Mahalanobis distance (MD) is a generalization of the Euclidean distance that accounts for correlations among the X properties. It is calculated as the distance to the center of the training data. The larger the MD, the less trustworthy the prediction.
Mahalanobis Distance p-value: The p-value gives the fraction of training data with an MD greater than or equal to the one for the given sample, assuming normally distributed data. The smaller the p-value, the less trustworthy the prediciton. For highly non-normal X properties (e.g., fingerprints), the MD p-value is wildly inaccurate.

Structural Similar Compounds

Name	2'-DEOXY-5-FLUOROURIDINE	5-BROMODEOXYURIDINE	L-ASCORBIC ACID
Structure	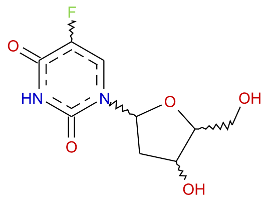	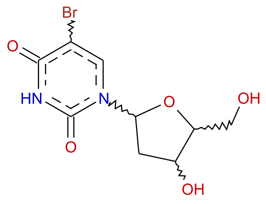	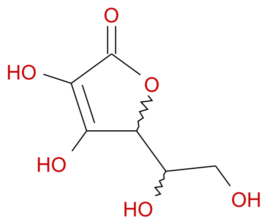
Actual Endpoint (-log C)	3.059	1.563	1.17
Predicted Endpoint (-log C)	2.42812	2.06634	2.00353
Distance	0.495	0.520	0.535
Reference	NCILB* NCI-E-C-72-3252;73	IYKEDH 4;467;73	OYYAA2 19;323;80

Model Applicability

Unknown features are fingerprint features in the query molecule, but not found in the training set.

1. All properties and OPS components are within expected ranges.

Feature Contribution

Top features for positive contribution			
Fingerprint	Bit/Smiles	Feature Structure	Score
ECFP_6	642810091	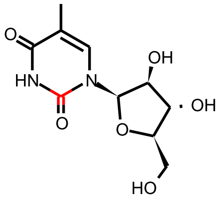 [*]:[c](:[*]):[*]	0.281

ECFP_6	-1074141656	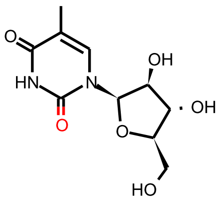 [*]=O	0.142
FCFP_6	-1272709286	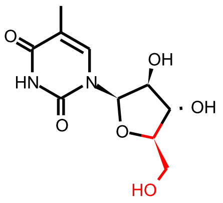 [*]C([*])CO	0.115
**Top Features for negative contribution**			
**Fingerprint**	**Bit/Smiles**	**Feature Structure**	**Score**
ECFP_6	2106656448	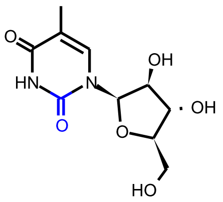 [*]C(=O)[*]	-0.352
ECFP_6	683445015	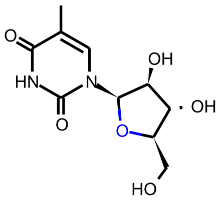 [*]O[*]	-0.266
FCFP_6	566058135	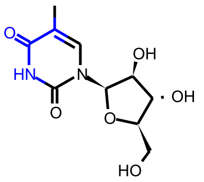 [*]NC(=O)C(=[*])[*]	-0.216

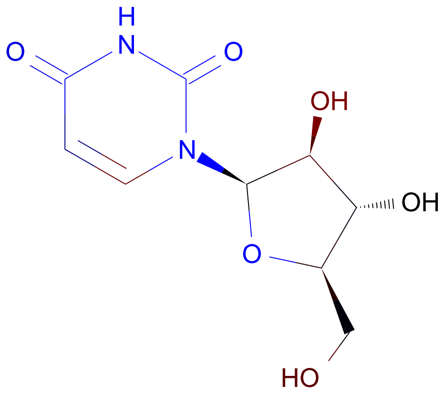

$C_9H_{12}N_2O_6$
Molecular Weight: 244.20137
ALogP: -2.451
Rotatable Bonds: 2
Acceptors: 6
Donors: 4

Structural Similar Compounds

Name	2'-DEOXY-5-FLUOROURIDINE	L-ASCORBIC ACID	5-BROMODEOXYURIDINE
Structure	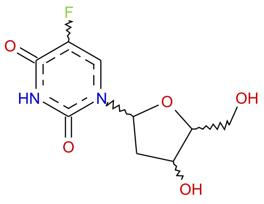	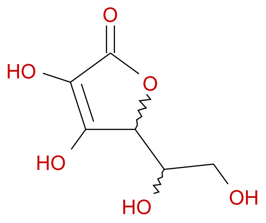	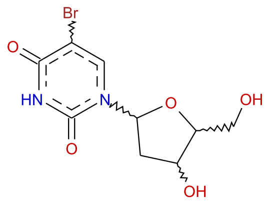
Actual Endpoint (-log C)	3.059	1.17	1.563
Predicted Endpoint (-log C)	2.42812	2.00353	2.06634
Distance	0.509	0.526	0.551
Reference	NCILB* NCI-E-C-72-3252;73	OYYAA2 19;323;80	IYKEDH 4;467;73

Model Prediction

Prediction: 4.343
Unit: g/kg_body_weight
Mahalanobis Distance: 21.674
Mahalanobis Distance p-value: 6.08e-015

Mahalanobis Distance: The Mahalanobis distance (MD) is a generalization of the Euclidean distance that accounts for correlations among the X properties. It is calculated as the distance to the center of the training data. The larger the MD, the less trustworthy the prediction.
Mahalanobis Distance p-value: The p-value gives the fraction of training data with an MD greater than or equal to the one for the given sample, assuming normally distributed data. The smaller the p-value, the less trustworthy the prediciton. For highly non-normal X properties (e.g., fingerprints), the MD p-value is wildly inaccurate.

Model Applicability

Unknown features are fingerprint features in the query molecule, but not found in the training set.

1. OPS PC52 out of range. Value: -4.4898. Training min, max, SD, explained variance: -4.4185, 5.4839, 1.093, 0.0043.

Feature Contribution

Top features for positive contribution			
Fingerprint	Bit/Smiles	Feature Structure	Score
ECFP_6	642810091	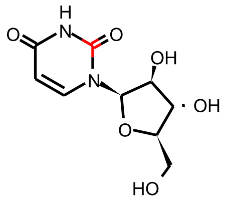 [*]:[c](:[*]):[*]	0.281

ECFP_6	-1074141656	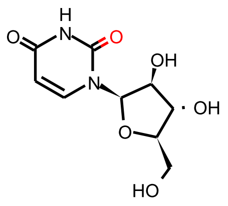 [*]=O	0.142
FCFP_6	-1272709286	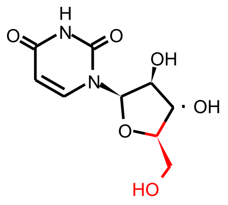 [*]C([*])CO	0.115

Top Features for negative contribution

Fingerprint	Bit/Smiles	Feature Structure	Score
ECFP_6	2106656448	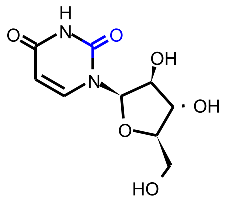 [*]C(=O)[*]	-0.352
ECFP_6	683445015	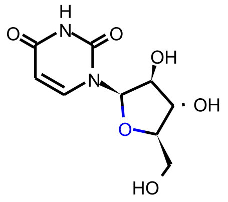 [*]O[*]	-0.266
FCFP_6	566058135	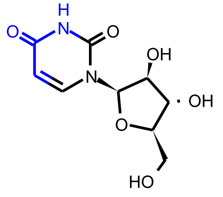 [*]NC(=O)C(=[*])[*]	-0.216

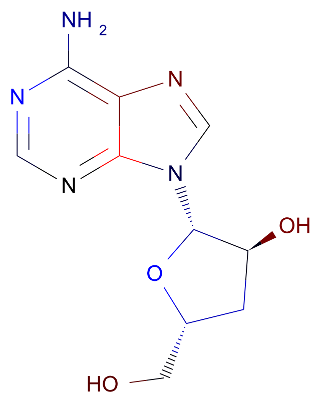

$C_{10}H_{13}N_5O_3$
Molecular Weight: 251.24191
ALogP: -1.308
Rotatable Bonds: 2
Acceptors: 7
Donors: 3

Structural Similar Compounds

Name	TUBERCIDIN	7H-PYRROLO[2;3-d]PYRIMIDIN-4-OL; 7-.beta.-d-RIBOFURANOSYL-	6-MERCAPTOPURINE RIBOSIDE
Structure	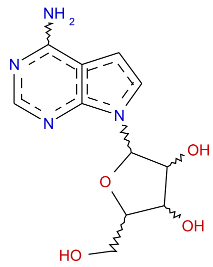	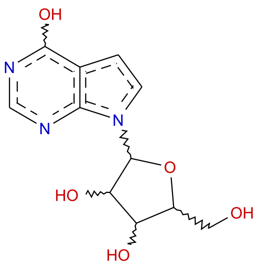	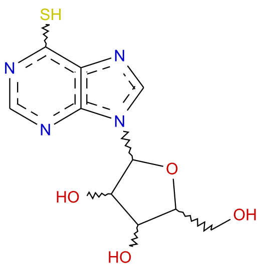
Actual Endpoint (-log C)	4.221	4.012	2.5
Predicted Endpoint (-log C)	2.53511	3.00148	2.54632
Distance	0.434	0.467	0.541
Reference	CNREA8 29;116;69	CNREA8 29;116;69	NIIRDN 6;853;82

Model Prediction

Prediction: 1.213

Unit: g/kg_body_weight

Mahalanobis Distance: 20.095

Mahalanobis Distance p-value: 3.16e-009

Mahalanobis Distance: The Mahalanobis distance (MD) is a generalization of the Euclidean distance that accounts for correlations among the X properties. It is calculated as the distance to the center of the training data. The larger the MD, the less trustworthy the prediction.
Mahalanobis Distance p-value: The p-value gives the fraction of training data with an MD greater than or equal to the one for the given sample, assuming normally distributed data. The smaller the p-value, the less trustworthy the prediciton. For highly non-normal X properties (e.g., fingerprints), the MD p-value is wildly inaccurate.

Model Applicability

Unknown features are fingerprint features in the query molecule, but not found in the training set.

1. All properties and OPS components are within expected ranges.
2. Unknown FCFP_6 feature: 16: [*]:[cH]:[*]
3. Unknown FCFP_6 feature: 1747237384: [*][c](:[*]):n:[cH]:[*]
4. Unknown FCFP_6 feature: -124685461: [*]:n:[cH]:n:[*]
5. Unknown FCFP_6 feature: -1564473960: [*]n1:[*]:[*]:[c](:[*]):[c]:1:n:[*]
6. Unknown FCFP_6 feature: -1151884458: [*]:n:[c](N):[c](:[*]):[*]
7. Unknown FCFP_6 feature: -306856457: [*]C([*])n1:[cH]:[*]:[*]:[c]:1:[*]
8. Unknown FCFP_6 feature: 1315416442: [*][C@H]1[*][*]O[C@H]1n(:[*]):[*]
9. Unknown FCFP_6 feature: 1069584379: [*]:[c](:[*])N

Feature Contribution

Top features for positive contribution			
Fingerprint	Bit/Smiles	Feature Structure	Score

ECFP_6	642810091	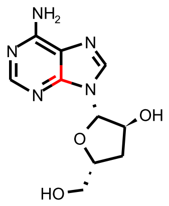 [*]:[c](:[*]):[*]	0.281
FCFP_6	-1272709286	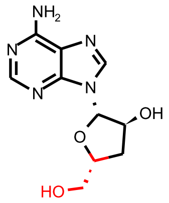 [*]C([*])CO	0.115
FCFP_6	178336375	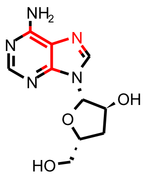 [*][c](:[*]):[c]1:n:[*]:[*]:[c]:1:[*]	0.109
**Top Features for negative contribution**			
**Fingerprint**	**Bit/Smiles**	**Feature Structure**	**Score**
ECFP_6	683445015	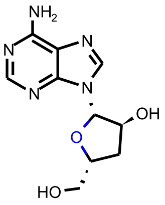 [*]O[*]	-0.266
ECFP_6	655739385	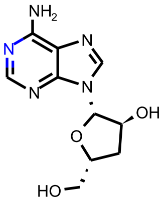 [*]:n:[*]	-0.239

ECFP_6	-801490360	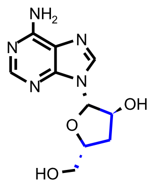<br>[*][C@@H]1[*][*][C@@H]([*])C1	-0.189

Ribavirin

TOPKAT_Rat_Oral_LD50

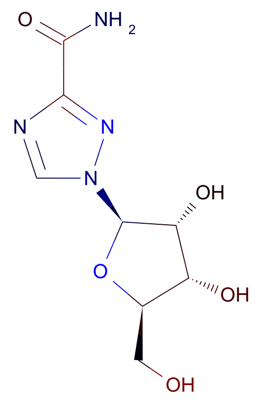

$C_8H_{12}N_4O_5$

Molecular Weight: 244.20467

ALogP: -2.745

Rotatable Bonds: 3

Acceptors: 7

Donors: 4

Model Prediction

Prediction: 0.750

Unit: g/kg_body_weight

Mahalanobis Distance: 19.779

Mahalanobis Distance p-value: 3e-008

Mahalanobis Distance: The Mahalanobis distance (MD) is a generalization of the Euclidean distance that accounts for correlations among the X properties. It is calculated as the distance to the center of the training data. The larger the MD, the less trustworthy the prediction.
Mahalanobis Distance p-value: The p-value gives the fraction of training data with an MD greater than or equal to the one for the given sample, assuming normally distributed data. The smaller the p-value, the less trustworthy the prediciton. For highly non-normal X properties (e.g., fingerprints), the MD p-value is wildly inaccurate.

Structural Similar Compounds

Name	RIBAVIRIN	BREDININ	TUBERCIDIN
Structure	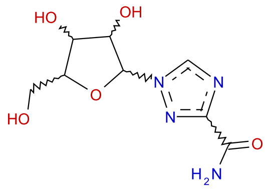	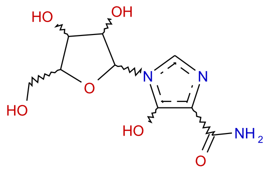	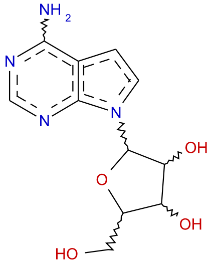
Actual Endpoint (-log C)	1.956	1.922	4.221
Predicted Endpoint (-log C)	2.51269	2.11795	2.53511
Distance	0.000	0.414	0.570
Reference	PCJOAU 18;667;84	IYKEDH 15;688;84	CNREA8 29;116;69

Model Applicability

Unknown features are fingerprint features in the query molecule, but not found in the training set.

1. All properties and OPS components are within expected ranges.
2. Unknown FCFP_6 feature: 16: [*]:[cH]:[*]
3. Unknown FCFP_6 feature: -2049666792: [*]C([*])n1:[cH]:[*]:[*]:n:1
4. Unknown FCFP_6 feature: 4427049: [*]n1:[*]:[*]:[c]([*]):n:1
5. Unknown FCFP_6 feature: -1539162406: [*]C(=[*])[c]1:n:[*]:[*]:n:1
6. Unknown FCFP_6 feature: 1747237384: [*][c](:[*]):n:[cH]:[*]
7. Unknown FCFP_6 feature: -124685461: [*]:n:[cH]:n:[*]
8. Unknown FCFP_6 feature: 1315416442: [*][C@H]1[*][*]O[C@H]1n(:[*]):[*]

Feature Contribution

Top features for positive contribution			
Fingerprint	Bit/Smiles	Feature Structure	Score

ECFP_6	642810091	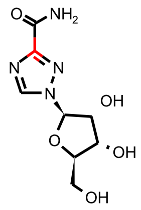 [*]:[c](:[*]):[*]	0.281
ECFP_6	-1074141656	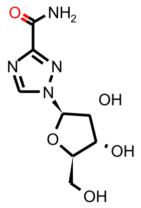 [*]=O	0.142
ECFP_6	-932108170	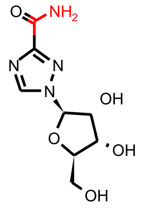 [*]C(=[*])N	0.126

Top Features for negative contribution

**Fingerprint**	**Bit/Smiles**	**Feature Structure**	**Score**
ECFP_6	683445015	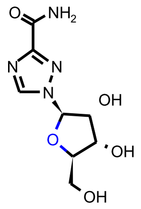 [*]O[*]	-0.266
ECFP_6	655739385	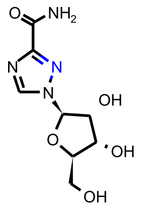 [*]:n:[*]	-0.239

FCFP_6	-1549103449	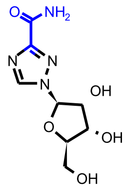 [*]:[c](:[*])C(=O)N	-0.117
